# Expansion and evolution of insect GMC oxidoreductases

**DOI:** 10.1186/1471-2148-7-75

**Published:** 2007-05-11

**Authors:** Kaori Iida, Diana L Cox-Foster, Xiaolong Yang, Wen-Ya Ko, Douglas R Cavener

**Affiliations:** 1 Department of Biology, 208 Mueller Laboratory, The Pennsylvania State University, University Park, PA 16802, USA; 2 Department of Entomology, 501 ASI Building, The Pennsylvania State University, University Park, PA 16802, USA

## Abstract

**Background:**

The GMC oxidoreductases comprise a large family of diverse FAD enzymes that share a homologous backbone. The relationship and origin of the GMC oxidoreductase genes, however, was unknown. Recent sequencing of entire genomes has allowed for the evolutionary analysis of the GMC oxidoreductase family.

**Results:**

Although genes that encode enzyme families are rarely linked in higher eukaryotes, we discovered that the majority of the GMC oxidoreductase genes in the fruit fly (*D. melanogaster*), mosquito (*A. gambiae*), honeybee (*A. mellifera*), and flour beetle (*T. castaneum*) are located in a highly conserved cluster contained within a large intron of the *flotillin-2 *(*Flo-2*) gene. In contrast, the genomes of vertebrates and the nematode *C. elegans *contain few GMC genes and lack a GMC cluster, suggesting that the GMC cluster and the function of its resident genes are unique to insects or arthropods. We found that the development patterns of expression of the GMC cluster genes are highly complex. Among the GMC oxidoreductases located outside of the GMC gene cluster, the identities of two related enzymes, glucose dehydrogenase (GLD) and glucose oxidase (GOX), are known, and they play major roles in development and immunity. We have discovered that several additional GLD and GOX homologues exist in insects but are remotely similar to fungal GOX.

**Conclusion:**

We speculate that the GMC oxidoreductase cluster has been conserved to coordinately regulate these genes for a common developmental or physiological function related to ecdysteroid metabolism. Furthermore, we propose that the GMC gene cluster may be the birthplace of the insect GMC oxidoreductase genes. Through tandem duplication and divergence within the cluster, new GMC genes evolved. Some of the GMC genes have been retained in the cluster for hundreds of millions of years while others might have transposed to other regions of the genome. Consistent with this hypothesis, our analysis indicates that insect GOX and GLD arose from a different ancestral GMC gene than that of fungal GOX.

## Background

Underlying biosynthesis and metabolism in all organisms is a large array of enzymes that catalyze a vast number of chemical reactions. Among these, oxidation-reduction reactions are the most prevalent and fundamental. Oxido-reductases typically entail electron transfer between the primary substrate and a co-factor such as NAD(P), FAD, or a cytochrome. Although similar structural domains are found in these enzymes, their primary amino acid sequences are generally not similar and therefore it is difficult to discern if they share a common evolutionary ancestor. An exceptional group in this regard is the family of GMC-FAD oxidoreductases [1] that shares an evolutionary conserved ca. 30 amino acid sequence comprising a beta-alpha-beta motif of the ADP-binding subdomain of FAD. Moreover, the GMC oxidoreductases contain five other blocks of conserved sequences dispersed throughout their primary sequence [2], supporting the hypothesis that they are evolutionarily homologous throughout.

Since the discovery of the GMC oxidoreductase family, several new enzymes have been added to this family [3,4]. Some of the unusual additions are hydroxynitrile lyase, which does not appear to catalyze an oxidation-reduction reaction [5], and celliobiose dehydrogenase, which contains an additional heme domain, not present in the archetypal GMC oxidoreductases [3]. In this study, we have searched the newly sequenced genomes of prokaryotic and eukaryotic organisms and have discovered a large number of previously unidentified GMC oxidoreductase genes in insects. Surprisingly, most of these newly identified genes are clustered in a conserved order and orientation, and are located in a large intron of the *flotillin-2 *gene in four distantly related insect species: *Drosophila melanogaster, Anopheles gambiae, Apis mellifera*, and *Tribolium castaneum*. We speculate that this insect GMC gene cluster may function in ecdysteroid metabolism. In addition, we report that the two glucose-metabolizing GMC enzymes in insects, GOX and GLD, are evolutionarily distinct from GOX in fungi and likely arose from a different ancestral GMC gene.

## Results and discussion

### The identification of a GMC oxidoreductase gene cluster in *Drosophila*

Prior to the sequence determination of the *D. melanogaster *genome, glucose dehydrogenase (*Gld*) located on the 3^rd ^chromosome (3R, 84D1-2) was the only known GMC oxidoreductase family member in Drosophila. Upon completion of the genome sequencing [6], we surveyed the entire genome for genes that belong to the GMC family based on the amino acid sequence characteristics. In addition to *Gld*, two GMC homologues, *NinaG *[7] and *CG6142*, are located on the 3^rd ^chromosome (3R, 97A1 and 86E7, respectively) and 12 other GMC homologues are located on the X-chromosome (12F5-13A1). These genes had been tentatively annotated as putative homologues of either choline dehydrogenase or *Gld *by the Berkeley Drosophila Genome Project [8].

However, the functions of the 12 GMC genes located on the X-chromosome are unknown except CG9504, which was recently identified by H. Takeuchi and coworkers as ecdysone oxidase (EO) [9]. Although these twelve genes share sequence similarity with choline dehydrogenase and GLD, their sequence similarity to these two enzymes is not significantly greater than to any other GMC oxidoreductases. This indicates that they are unlikely to encode either choline dehydrogenase or GLD, arguing against the initial annotation of these genes by the Berkeley Drosophila Genome Project. Moreover, choline dehydrogenase has not been reported in insects and we have not been able detect this enzyme in Drosophila by biochemical assays (D. R. Cavener, unpublished data).

**Figure 1:**
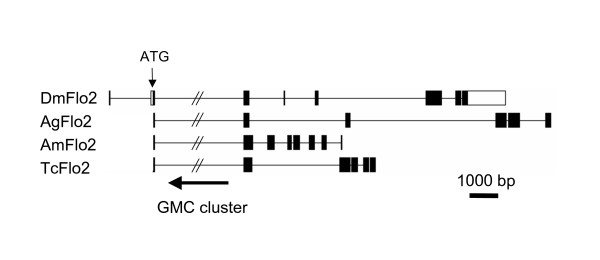
**The predicted gene structure of *Flo-2***. *D. melanogaster Flo-2 *structure is from GenBank. Coding regions are shown in black, and non-coding in white. In the other species, only predicted coding regions are shown. All genes are in scale, except the large intron that contains GMC cluster. The genes are positioned to align at the start codon and at the beginning of the exon immediately 5' of GMC cluster. The coding sequence information of this gene can be found in Additional File 4.

**Figure 2:**
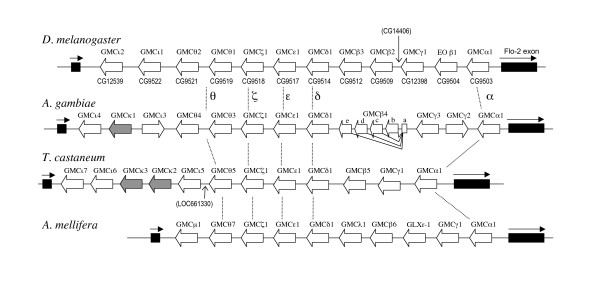
**Comparison of the structure of the GMC cluster in *D. melanogaster*, *A. gambiae*, *A. mellifera*, *T. castaneum***. The GMC cluster is located within the *Flo-2 *gene in opposite transcriptional orientation in all species. The coding regions of *Flo-2*, adjacent to the cluster, are shown in solid black. The transcriptional orientation of each GMC gene is shown by an open arrow that also shows the position of each gene. Only for *A. gambiae *GMCβ4, the gene structure is shown to indicate alternative splicing (also see Figure 5). The other genes may or may not contain introns. The highly conserved gene subfamilies, GMCα, δ, ε, ζ, and θ are indicated by dotted lines. Positions of non-GMC genes present in *D. melanogaster *and *T. castaneum *are indicated by vertical arrows. The sequences of these non-GMC genes are not homologous to each other. Figures are not drawn to scale.

The twelve X-chromosome GMC genes in *D. melanogaster *are in the same transcriptional orientation comprising a gene cluster encompassing 80.9 kb without any interruptions by non-GMC genes, with the exception of CG14406 located between CG9509 and CG12398. Pairwise comparisons of amino acid sequences of these genes revealed a varied degree of similarity to each other (27–69% amino acid identity). Surprisingly, this GMC cluster is entirely within the second intron of *flotillin-2 *(*Flo-2*), a non-GMC gene that is transcribed in the opposite direction to that of the GMC genes (Figures 1 and 2). The *Flo-2 *intron containing the GMC cluster is large, spanning over 83.2 kb, and exclusively contains the GMC gene cluster and CG14406. The first exon (45 bp) and most of the second exon (146 bp) of *Flo-2 *are non-coding, with only the first 16 amino acid residues of *Flo-2 *encoded by the 3' end of the second exon (Figure 1). The genome of *D. pseudoobscura *has recently been sequenced [10], and we found that *D. pseudoobscura *has an orthologous GMC cluster with an identical gene composition and order to that of *D. melanogaster *(data not shown).

### Evolutionary conservation of the GMC cluster in insect genomes

To examine the evolution of the GMC genes in insects, we searched for the GMC homologues in three other insect species including *Anopheles gambiae*, *Apis mellifera*, and *Tribolium castaneum *for which entire genomic sequences were available [11-13]. We performed TBLASTN against each genome using the *D. melanogaster *GLD protein sequence and identified multiple GMC genes in all species (Table 1). We discovered that 10–12 GMC genes in *A. gambiae*, *A. mellifera*, and *T. castaneum *were clustered in a tandem array as seen in Drosophila. In *A. mellifera*, and *T. castaneum*, more GMC genes exist outside the gene cluster, as compared to *D. melanogaster *and *A. gambiae *(Table 1).

**Table 1:** The predicted number of GMC genes in the insect genomes

Species	Inside cluster	Outside cluster	Total	(GLD/GOX related)^a^
*D. melanogaster*	12	3	15	(1)
*A. gambiae*	12	3	15	(1)
*A. mellifera*	10	8	18	(4)
*T. castaneum*	12	11	23	(3)

^a^The number of GLD/GOX-related genes is included in the other columns.

In order to identify orthologs of the Drosophila GMC genes in the other three insect genomes, all amino acid sequences of the newly discovered GMC genes were aligned together with several outgroup GMC enzymes, including choline dehydrogenase from *E. coli*, *C. elegans*, and humans, two *NinaG *genes from Drosophila and beetles, and fungal glucose oxidase. A considerable proportion of the alignment contained gaps or highly diverged residues due to the long divergence time among these distantly related species. However, the average sequence distance across all pairwise comparisons (0.63) was below the saturated distance (~0.94) estimated from the average amino acid frequencies across all sequences analyzed (see Additional File 1 for the sequence alignment).

**Figure 3:**
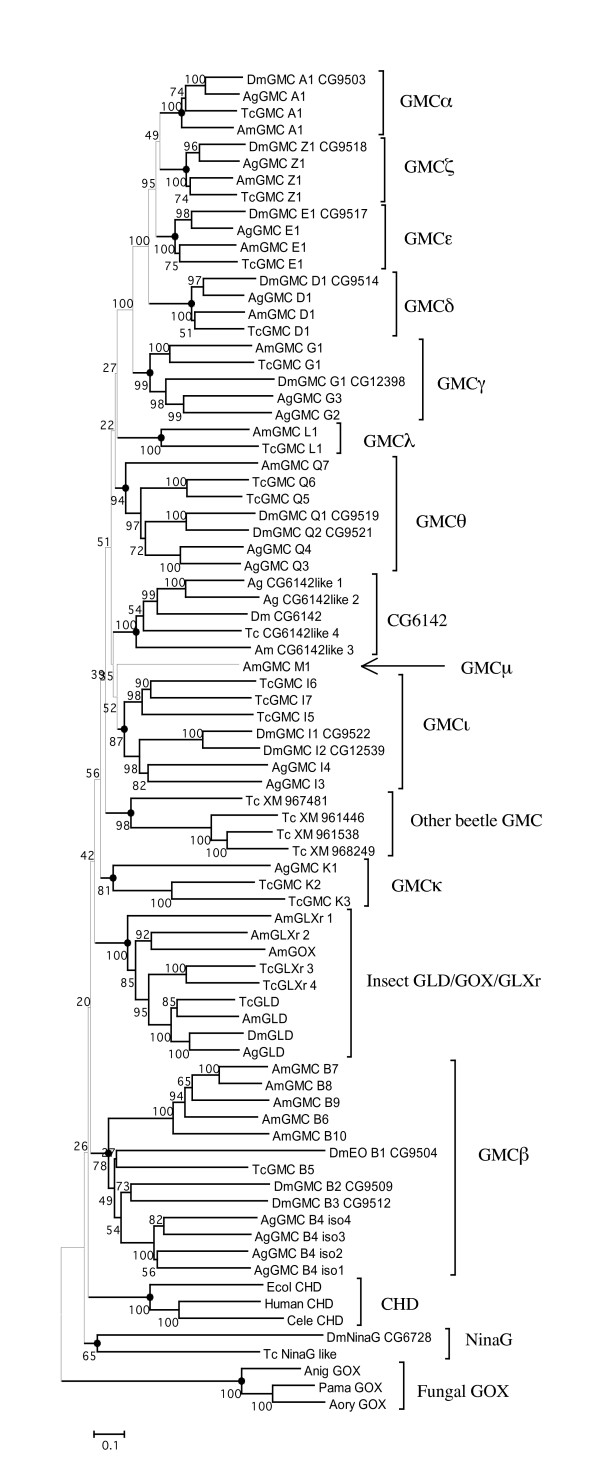
**Phylogeny of insect GMC oxidoreductases**. The phylogeny was generated using the neighbor-joining method with Poisson correction and bootstrap replicates 500 times. Bootstrap values are shown at nodes. Based on the phylogeny, genes were classified into gene subfamilies (see text). A Greek letter in a sequence name is shown in a Roman alphabet in this figure (e.g., A for α). Abbreviations: Dm (*D. melanogaster*), Ag (*A. gambiae*) Am (*A. mellifera*), Tc (*T. castaneum*), Ecol (*Escherichia coli*), Cele (*Caenorhabditis elegans*), Anig (*Aspergillus niger*), Aory (*Aspergillus oryzae*), Pama (*Penicillium amagasakiense*), EO (ecdysone oxidase), and CHD (choline dehydrogenase). "DmEO_B1" (CG9504) belongs to GMCβ, but it is indicated as EO instead of GMC because its functional identity is known. The number with 4 or more digits (e.g., CG9503, XM961446) indicates GenBank accession number of the sequence (detailed sequence information is in Additional File 7). "iso1-4" of GMCβ4 indicates four isoforms of this gene (see Figure 5). All protein sequences used in this study are in Additional File 8.

Next we reconstructed a neighbor-joining tree with Poisson correction for sequence distances in which orthologous genes from different species are expected to cluster together [14,15]. The results from our phylogenetic analysis provide reliable evidence on identifying GMC orthologues (Figure 3). The tree showed 13 major monophyletic clades (excluding outgroup sequences), and sequences clustered in each clade were classified as a subfamily. Alignments within subfamilies were less ambiguous with relatively lower average pairwise sequence differences (Additional Files 1 and 2). Results from the bootstrap re-sampling analysis indicated that the clustering of these subfamilies is reliable. While the bootstrap scores supporting three subfamilies (GMC β, ι, and κ) are 78, 87, and 81, respectively, all other subfamilies were supported by a high bootstrap value (= 94) (Figure 3). For genes that reside within the cluster, the identified subfamilies were designated with different Greek letters (e.g., GMCα). Within each subfamily, numbers were assigned to identify individual genes. When a subfamily contains only one gene from each species, we assumed that these were orthologous and assigned the same number ("1") for all species (e.g., "Dm GMCα1" and "Ag GMCα1"). In cases of apparent paralogues in some species, individual members of different species were given different numbers (e.g., "Dm GMCγ1", "Ag GMCγ2", and "Ag GMCγ3") because orthologues could not be identified.

These subfamilies were also identified when a different phylogenetic algorithm (maximum parsimony method) or a different sequence substitution model (Jones-Taylor-Thornton amino acid substitution model [16]) was used for phylogenetic reconstruction (Additional File 3). The phylogenetic tree reconstructed by the maximum parsimony method showed that each of the 13 subfamilies remained clustered as a monophyletic clade except for GMCβ and κ subfamilies although the bootstrap scores were not high. It has been known that the maximum parsimony method becomes unreliable when the extent of homoplasy (backward and parallel substitutions) is high, a problem often found when sequences are diverged considerably [15]. To take into account the problem of multiple substitutions, we also built a neighbor-joining tree by assuming a more complex substitution model (the Jones-Taylor-Thornton model). The results showed that each subfamily was clustered as a monophyletic group with a good bootstrap support (>88 for all subfamilies except for the GMC κ, which had the bootstrap score, 70).

The classification of these subfamilies is further supported by the striking evolutionary conservation in their order and orientation within the GMC gene cluster among the four distantly related species (Figure 2). The most readily identifiable are GMCα found at the 5' end of the cluster and four tandemly-arrayed families, GMCδ, ε, ζ, and θ, in the middle of the cluster. These genes have a single copy in the same orientation, except GMCθ, which has two copies in some species. GMCγ has also retained a well-conserved position between GMCα and GMCβ genes. Although *A. gambiae *has two GMCγ genes in opposing directions, the other three species contain a single copy in the same orientation. The other subfamilies also show conserved positions among different species despite that they contain the varied number of gene copies (0–3) in different species. Two copies of *D. melanogaster *GMCβ genes are located in the relative position conserved among the other species (between GMCγ and GMCδ) though EO-β1 is located between GMCα and GMCγ genes. The GMCι subfamily is missing from *A. mellifera*, but in the other species, it is always located at the 3' end of the cluster. The GMCκ subfamily is present only in *A. gambiae *and *T. castaneum*, and in both species, the GMCκ genes are located between the GMCι genes.

### Evolution of the GMC cluster

The overall conservation of the GMC cluster among insect species is striking, as microsynteny is typically not conserved among these highly diverged species. For example, only 30% of *A. gambiae *genes that are homologous to *D. melanogaster *genes in the *Adh *region retain microsynteny, where each syntenic region includes only two or three genes [17]. The conservation of the cluster region is highly specific to the cluster, and does not extend to the flanking regions. We examined the location of some *A. gambiae *genes that are apparently homologous to *D. melanogaster *genes to see if any of the genes surrounding the *D. melanogaster *cluster maintained microsynteny in Anopheles. Examined genes included *Rut*, *CG14411*, *CG14411*, and *CG14407 *in the 3' direction of the cluster and *CG9009*, *Eag*, *Hiw*, *CG5530*, *CG5560*, and *CG15027 *in the 5' direction of the cluster, which covered about 300 kb in the area surrounding the cluster. We found that the only gene that maintained microsynteny with the cluster was *Flo-2*, which contains the cluster within one of its introns. In fact, in all four species, the cluster locates within the homologous intron of *Flo-2*, and the first 16 amino acids of FLO-2 are encoded by an exon located at the 3' of the cluster (Figure 1 and Additional File 4). *Flo-2 *is transcriptionally oriented in the opposite direction to almost all of the GMC genes within the cluster (Figure 2). Sequences of FLO-2 homologues in these three insect species are highly conserved with *D. melanogaster *FLO-2 (77–88% pairwise amino acid identity).

The fact that four core genes (GMCδ, ε, ζ, and θ) in the middle of the GMC cluster have remained in tandem and in the same orientation over hundreds of millions of years strongly suggests that this cluster, partly or entirely, has been maintained by natural selection. None of the four core GMC cluster genes is a close homologue to any of the other GMC oxidoreductases for which enzyme substrate specificity has been determined, and therefore their catalytic activities remain to be determined.

**Figure 4:**
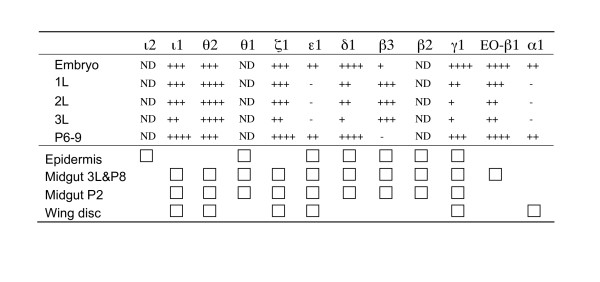
**Expression patterns of GMC genes in the *D. melanogaster *cluster**. The upper half shows developmental stage-specific expression patterns based on RT-PCR analyses on whole fly samples. The higher number of "+" means a higher level of expression. "-," undetectable; "ND," not determined. The bottom half shows a summary of tissue-specific expression patterns obtained from a previously published article [9]. The presence of each block indicates a high level of expression. First to third larval instar (1L-3L) and pupal (P) developmental stages are indicated.

Other similar examples of highly conserved gene clusters include the bithorax and Antennapedia homeobox complexes of Drosophila [18] and the β-globin cluster in vertebrates [19]. The genes in these clusters are coordinately regulated by cis-acting elements that require maintenance of a specific order and transcriptional orientation in the cluster. We examined the expression patterns in different developmental stages of nine *D. melanogaster *GMC genes in the cluster (EO-β1, GMCα1, γ1, β3, δ1, ε1, ζ1, θ2, and ι1). We found that these genes exhibit varying patterns of temporal expression; EO-β1, GMCα1, γ1, δ1, and ε1 highly expressed during embryonic and metamorphic development whereas GMCβ3 and θ2 were more highly expressed during larval growth (Figure 4). In addition, the Takeuchi and colleagues showed that the Drosophila GMC genes exhibit distinct patterns of tissue-specific expression during the critical transition from larval to metamorphic development [9] (summarized in Figure 4).

The presence of ecdysone oxidase (EO-β1) gene in the cluster suggests that the cluster may encode a series of enzymes that are involved in ecdysone metabolism. Ecdysone oxidase catalyzes the oxidation of ecdysone to dehydroecdysone within pathways involved in degradation of edysone and/or generation of unique ecdysteroids [9]. A diversity of ecdysteroids is produced in insects, and their tissue- and developmental stage-specific modification and degradation is important in the orchestration of insect development [20-22]. We speculate that the GMC cluster comprises a network of coordinately regulated suite of genes that act to modify developmental and physiological processes in tissue and spatially distinct patterns. By maintaining these genes in a cluster, combinatorial regulatory elements can efficiently coordinate their regulation.

Why then is GMC cluster located in a large intron of the *Flo-2 *gene? The parsimonious hypothesis is that the ancestral GMC gene or cluster was accidentally transposed into the *Flo-2 *gene and has never had the opportunity to leave without destroying itself or the *Flo-2 *gene. A more compelling possibility is that the transcriptional regulation of the GMC complex and *Flo-2 *are intimately tied together. Conservation of other gene clusters, including the vertebrate globin gene cluster and the insect homeobox gene clusters, appears to be due to a requirement of these genes to be coordinately regulated by local cis-acting mechanisms. *Flo-2 *encodes the lipid raft protein *flotillin-2*. As lipid rafts contain cholesterol and their derivatives including steroids [23] and steroid binding proteins have been detected in lipid rafts [24], *Flo-2 *and the GMC genes, including ecdysone oxidase, may be coordinately regulated in support of a common developmental or physiological function. We speculate that cis-acting control elements may exist in the GMC cluster and act to coordinately regulate the expression of the GMC genes, and perhaps the *Flo-2 *gene as well.

### Duplication of genes and exons

Homologous genes typically arise from tandem duplication events that result in two or more homologues tandemly arrayed. The GMC cluster has retained much of its history of gene duplication events that gave rise to the cluster. At a subfamily level, this is most apparent for the GMCα, γ, δ, ε, and ζ subfamilies. As these genes are tandemly arrayed (Figure 2) and phylogenetically form a distinct group of subfamilies (bootstrap value, 100; Figure 3), we postulate that these genes arose from the same ancestral gene prior to the divergence of the major insect subfamilies.

Duplication events are also seen within a subfamily. Four of the subfamilies in the GMC cluster, GMCα, δ, ε, and ζ, have remained as single-copy genes in the genome, whereas GMCβ, γ, θ, ι, and κ, are multi-copy genes. Particularly, each pair of GMCγ genes (*A. gambiae*), GMCθ genes (*D. melanogaster *and *A. gambiae*), GMCκ genes (*T. castaneum*), and GMCι genes (*D. melanogaster*) is supported by a very high bootstrap value (99–100) and is arrayed tandemly. This indicates that duplication events of the GMC genes had occurred after the emergence of the four insect species from a common ancestor.

**Figure 5:**
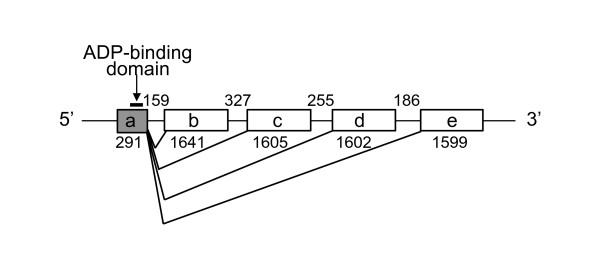
**Alternative splicing of *A. gambiae *GMCβ4 gene**. The figure is shown in a conventional direction of 5' to 3' from left to right (opposite to the direction in Figure 2). The length of exons and introns are shown in base pairs. Exon *a *(in gray color) contains the highly conserved ADP-binding domain (arrow) and is predicted as a shared exon in all isoforms. Exons *b-e *are predicted to be alternatively spliced C-terminal exons. The splicing patterns of isoforms indicated in the phylogeny (Figure 3) are as follows: "iso1", exons *a*/*b*; "iso2", exons *a*/*c*, "iso3", exons *a*/*d*; "iso4", exons *a*/*e*. The detailed sequence information of this gene can be found in Additional File 5.

In addition, some exons of one gene show evidence for multiple duplication events. The C-terminus exons of the *A. gambiae *GMCβ gene has undergone multiple duplications giving rise to a tandem array of four alternative exons, which we predicted have the essential splice consensus sequences to join in-frame a common 5' exon encoding the N-terminus (Figure 5 and also see Additional File 5). The common N-terminus exon of GMCβ4 encodes the highly conserved beta-alpha-beta fold of the ADP-binding domain common to all GMC oxidoreductases. While the four downstream C-terminal encoding exons (*b*, *c*, *d*, and *e*) are more similar to each other than to other GMC oxidoreductase (bootstrap value, 100), nonetheless the sequences have diverged considerably from each other (Figure 3). Two of the predicted alternatively spliced isoforms (*a/d *and *a/e*) are present in the GenBank EST database supporting our predicted model of this gene (e.g., BX606462 and BX607386 for *a/d*; BM635774 and BM622197 for *a/e*). Approximately 9% of alternative splicing in eukaryotes involves duplicated tandem exons [25,26]. This mechanism may offer an economical strategy to expand a cluster of similar enzymes. In the case of the GMCβ4 gene, duplicated C-terminus exons that contain a substrate-binding region can gain a new function while sharing the FAD-binding region (exon *a*) and regulatory elements.

### Diversification of GLD and GOX in insects

In addition to GMC genes in the cluster, we discovered several other GMC genes that reside outside the cluster in *D. melanogaster*, *T. castaneum*, *A. gambiae*, and *A. mellifera *(Table 1). While the identity of these genes is largely unknown, our phylogenetic analysis suggests that several of them belong to a gene subfamily containing glucose dehydrogenase (GLD) and glucose oxidase (GOX) ("insect GLD/GOX/GLXr," supported by a bootstrap value, 100; Figure 3 and Table 1). These two enzymes catalyze the conversion of β-D-glucose to δ-gluconolactone but differ in the electron acceptor [1,27].

Apparent orthologues of the previously identified GLD in *D. melanogaster *were found in all four insect species (bootstrap value, 100; Figure 3). The *Gld *genes of *D. melanogaster *and *A. gambiae *share a very similar exon/intron structure while honeybee *Gld *structure is more divergent. Similar patterns in developmental expression are also observed between honeybees and Drosophila (D. L. Cox-Foster, unpublished). Because GLD is an essential gene in Drosophila for exoskeleton metabolism [28,29], we speculate that all other arthropods with exoskeletons contain GLD.

**Figure 6:**
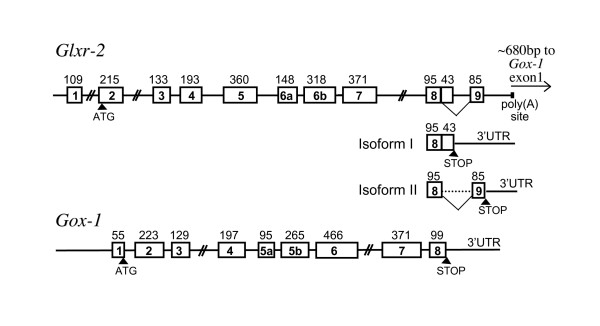
**The structures of bee *Glxr-2 *and *Gox-1 *genes**. The homologous exons of the two genes are indicated by the same numbering of exons. Exon 6 of *Glxr-2 *and exon 5 of *Gox-1 *are divided into two ("a" and "b") by a single intron insertion. The *Glxr-2 *gene is alternatively spliced at the 3' end in the diagramed manner. The length of each exon is shown in base pairs. The detailed sequence information of the alternatively spliced region can be found in Additional File 6.

In addition to GLD, honeybees and beetles have additional proteins that are closely related to GLD. One gene functionally known is GOX-1 of *A. mellifera *previously identified by K. Ohashi and coworkers [30]. The other genes in bees and beetles are functionally unknown but are nearly equal in sequence similarity to the GLD group and bee GOX-1; we denoted these as GLD/GOX related proteins (GLXr). *A. mellifera *has two GLXr genes: GLXr-1 located in the GMC cluster and GLXr-2 in tandem with GOX-1. We isolated genomic clones containing GOX-1 and discovered GLX-r2 in these clones adjacent to GOX-1 in the same transcriptional orientation. Upon completion of the genomic sequence of *A. mellifera*, we confirmed that these two genes were adjacent and only about 700 bp apart. These two genes share most of the exon/intron boundaries (Figure 6), strongly suggesting that they have arisen through tandem duplication. We generated GLX-r2 cDNAs from adult worker bees and detected two alternatively spliced mRNA isoforms of GLX-r2 that differ at the 3' end, resulting in two C-terminally different protein products (Figure 6 and Additional File 6). The coding sequence of GLXr-2 isoform I terminates in exon 8, whereas isoform II splices out of exon 8 before the termination codon and adds a unique carboxy-terminus encoded in exon 9 (85 bp).

**Figure 7:**
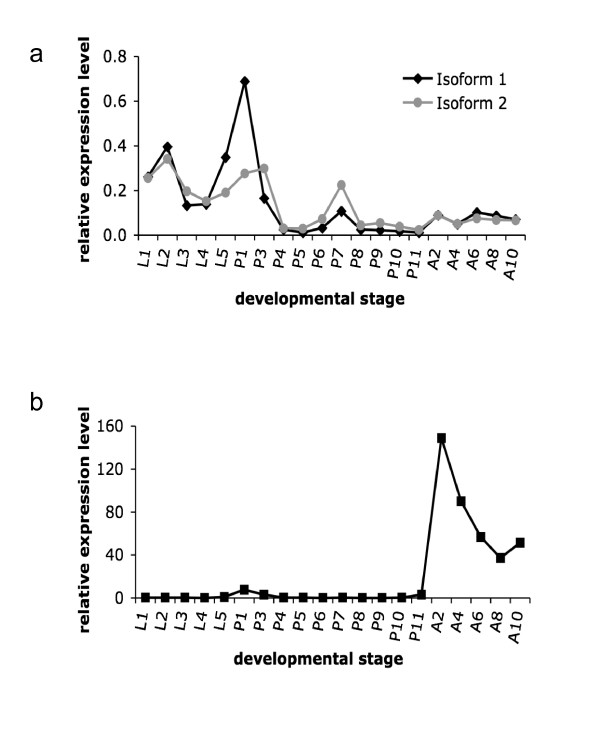
**The expression patterns of bee GLXr-2 and GOX-1**. Relative expression levels normalized to the level of the EF1α gene are shown (a, two isoforms of *Glxr-2*; b, *Gox-1*). Developmental stages are indicated as follows: L1 (first instar larval stage), P1 (post-capped day 1; the first day when a larva has been sealed in a cell), and A1 (adult day 1; the first day when a bee has emerged from a cell).

GOX-1 and GLXr-2 mRNAs have distinct patterns of expression throughout bee development (Figure 7). Expression of GOX-1 in the total-body samples was very low at pre-adult stages but was induced more than 100-fold in newly emerged adults (Figure 7b). In contrast, the overall expression of GLXr-2 in the total-body samples was higher in pre-adult stages, especially at post-capped day (PC) 1–3 (Figure 7a). In addition, GLXr-2, especially isoform II, is highly expressed in the epithelium tissues of wings of bees at PC7-10 (data not shown), while GOX has no expression in the same tissues. This expression pattern in wings is similar to the pattern of *D. melanogaster *GLD [29]. Therefore, it seems that GLXr-2 ancestor gene achieved functional diversification through duplication and alternative splicing in the bee lineage.

However, GOX-1 and GLXr-2 also share some expression patterns; for example, both are highly expressed in hemocytes [31] and are induced in similar patterns by immune challenge [32]. These data indicate that these genes may share some common regulatory elements for those expression patterns, further suggesting that genetic linkage between these two genes has been under an evolutionary constraint.

Besides insects, glucose-metabolizing GMC enzymes are found only in fungi to date. Interestingly, GLD/GOX gene copies evolved paraphyletically between insects and fungi (Figure 3). In contrast, strong evolutionary conservations are observed in sequences of choline dehydrogenase as the orthologous gene copies are identified among *E. coli*, *C. elegans*, and human. For GLD/GOX enzymes, substantial sequence differences may have accumulated over a long evolutionary divergence time between insects and fungi, which may make the phylogenetic relationship between the two groups obscure (Table 2). Alternatively, their remote relationship may indicate that the GLD/GOX genes in insects may not be descended from the fungal GOX through subsequent speciation events. Instead they may have arisen independently from a paralogue of fungal GOX on the ancestral lineage leading to insects after splitting from fungi. In this case, functional convergence may have occurred in insects and fungi independently.

**Table 2:** Pairwise amino acid sequence identity of GLD, GOX, and GLXr proteins

	1	2	3	4	5	6	7	8	9	10	11	12	13
1. AmGLXr-1													
2. TcGLXr-3	0.40												
3. TcGLXr-4	0.41	0.63											
4. TcGLD	0.45	0.53	0.49										
5. AmGLD	0.46	0.54	0.48	0.68									
6. DmGLD	0.45	0.56	0.52	0.65	0.63								
7. AgGLD	0.46	0.54	0.52	0.65	0.63	0.71							
8. AmGLXr-2	0.41	0.50	0.48	0.51	0.53	0.50	0.53						
9. AmGOX-1	0.40	0.43	0.43	0.42	0.42	0.44	0.44	0.50					
10. Human CHD	0.32	0.31	0.33	0.34	0.35	0.32	0.35	0.32	0.32				
11. Ecol CHD	0.28	0.32	0.31	0.33	0.36	0.32	0.33	0.31	0.30	0.48			
12. Anig GOX	0.25	0.23	0.23	0.24	0.25	0.24	0.25	0.23	0.23	0.23	0.27		
13. Pama GOX	0.23	0.23	0.22	0.24	0.23	0.24	0.24	0.23	0.22	0.23	0.26	0.63	
14. Aory GOX	0.24	0.22	0.23	0.23	0.22	0.22	0.23	0.22	0.21	0.22	0.26	0.65	0.73

The number of amino acids that is identical per site is calculated for each pair of sequence comparison with exclusion of gaps (pairwise gap deletion). Refer to Figure 3 for the details of sequence names.

### Evolution of GMC genes

Some GMC genes in the cluster, namely GMCθ,λ, andβ subfamilies, have homologues in one or more species that exist outside of the cluster, which suggests that the GMC cluster may have been the birthplace for all insect GMC genes including GLD and GOX. This hypothesis is supported by several facts as follows. (1) GLXr-1 is located in the GMC cluster of *A. mellifera *whereas all other GOX, GLD, and GLXr genes are located outside the GMC cluster. Importantly, GLD is present on the same chromosome as the GMC cluster in three of the four species. The *Gld *genes of *A. gambiae*, *T. castaneum*, and *A. mellifera *are located 30 Mb, 9 Mb and 1 Mb apart from the GMC cluster, respectively. (2) A cluster of three tandemly duplicated GMCβ genes in *A. mellifera *are present outside of the GMC cluster (GMCβ7–9) but are still on the same chromosome, and these have the closest relationship with GMCβ6 in the cluster (bootstrap value, 94). (3) One of the *T. castaneum *GMCθ genes, which apparently arose from a duplication event, is located ~250 kb away from the cluster on the same chromosome (GMCθ6). (4) The closest homolog of the cluster-localized *A. mellifera *GMCλ1 is *T. castaneum *GMCλ1 located outside the cluster on a different linkage group from that of the cluster (bootstrap value, 100). Together these data are consistent with the hypothesis that the GMC genes have undergone tandem duplication in the GMC cluster and then one or more copies have relocated outside of the cluster, frequently on the same chromosome, before some have been further dispersed to other chromosomes.

Relocation of genes outside the cluster would likely occur by transposition for two reasons. First, the most common event of transposition is "local hopping" to a nearby region on the same chromosome [33], and secondly, transposition would allow excising one or more genes without disrupting the *Flo-2 *gene. Other larger scale chromosome rearrangements (e.g., inversion and translocations) would likely disrupt the *Flo-2 *gene in which the GMC cluster resides. In summary, we propose that the location of the highly conserved core genes (GMCδ, ε, ζ, and θ) is constrained due to shared regulatory elements within or flanking the cluster, whereas the other GMC genes are less constrained and have spawned a number of new GMC genes that have relocated to other regions of the genome.

## Conclusion

Insects contain a cluster of GMC oxidoreductase genes that is highly conserved in gene composition, gene order, transcriptional orientation, and presence in a large intron of the *Flo-2 *gene. In addition, a smaller number of GMC oxidoreductase genes exists outside of this cluster but may have originated from the cluster and evolved independently. Although fungal GOX and insect GLD are closely related functionally, their relatively low sequence similarity suggests that they arose independently from an ancient GMC gene. In contrast several glucose oxidase and glucose dehydrogenase genes within insects have a high degree of sequence similarity consistent with the hypothesis that these two genes have more recently arose from a common GLD/GOX common ancestor since the divergence of insects.

## Methods

### Data mining

The presence of all GMC-related sequences in *A. gambiae*, *A. mellifera*, and *T. castaneum *genomes was detected by TBLASTN using *D. melanogaster *GLD sequence against the database at the National Center for Biotechnology Information (NCBI) [34]. The preliminary sequence data for *T. castaneum *genome was provided to the NCBI from Baylor College of Medicine Human Genome Sequencing Center [13]. One of the outgroup sequences used in our phylogenetic study, *Aspergillus oryzae *putative GOX, was obtained from the DOGAN (Database Of the Genomes Analyzed at NITE [National Institute of Technology and Evaluation in Japan]) [35]. All other outgroup sequences were obtained from the NCBI.

When there was a predicted protein sequence in the public database that corresponded to the BLAST hit, we evaluated the sequence based on the alignments with GMC genes in other insect species. When the sequence was reasonably aligned with the putative homolog, we used the predicted sequence. However, when the sequence had a major insertion/deletion and/or did not have major conserved regions, we manually annotated a sequence. The details of sequence information can be found in Additional Files 7, 8, and 9.

### Phylogenetic analysis

Multiple alignments of protein sequences were carried out using CLUSTALW [36,37]. We reconstructed a phylogenetic tree using a neighbor-joining algorithm [38] implemented in MEGA3 [39]. The pairwise distance matrix was estimated based on the Poisson correction model [40] with exclusions of gaps for each pair of sequence comparison (pairwise gap deletion). A bootstrap re-sampling analysis with 500 replicates was also performed to evaluate the inferred tree topology [41].

### Primers

Primers used for this study were as follows: AmGLXr-2, 5'-CGGCCCGGAGAATCATCAG-3', 5'-ATCCGCATTTACATTTCTTTGGTCTC-3' (which amplifies two products of alternatively spliced isoforms, 548 bp for Isoform I and 455 bp for Isoform II); AmGOX-1, 5'-CTGGACTGGAAGTATTACACTACGAAC-3', 5'-ACGATTGGTGATTGTGAAGGTTCT-3'; AmEF1α (elongation factor 1 alpha), 5'-ATGGGCAAGGGCTCGTTCAAGTA-3', 5'-CTTTCCGTCAGCGTTACCATCTTTGC-3'; DmGMCα1 (CG9503), 5'-TGGTGGTTATCTGACAGTTGGTGAGG-3', 5'-ATGGCTTTCGTTCGGGATAATGC-3'; DmEO-β1 (CG9504), 5'-ATGCCATTGTTTCTGCTCTTCGGTT-3', 5'-AACCAGTAGTCATCGGAATCGGC-3'; DmGMCβ3 (CG9512), 5'-AAAATGTTGGGCGGCACGAATGG-3', 5'-TCCTGAGTGCCCAAGATGTCCATTT-3'; DmGMCγ1 (CG12398), 5'-ATCCCGATGGTGATTTCAATGGT-3', 5'-CAGAATCACCTCTCGTTTGGCTC-3'; DmGMCδ1 (CG9514), 5'-GACGGGTTTCGGTTTCTATCAGTTCA-3', 5'-AATCTCTTCATAGCCTGCGTTTCACC-3'; DmGMCε1 (CG9517), 5'-CATTGGGCATCGTTGGGTAATCCG-3', 5'-TTGGAAACGATTGCGGGTGACAGT-3'; DmGMCθ2 (CG9521), 5'-TTCAAGGATGTGCTGCCGTATTTCAA-3', 5'-ACAGCATCAGTAGTTGGGGCGTATTG-3'; DmGMCι1 (CG9522), 5'-CGGAGGAGTGGAGAACATAGTGC-3', 5'-CAATCCCCGACAGCATCAGCAACT-3'; DmGMCζ1 (CG9518), 5'-TTCAATCCCACAGCCGTCACCTTTC-3', 5'-GTCTATTTGCCTGCCGCTTTACTTTGT-3'.

### Genomic library screening and 5' RACE

The *Apis mellifera *genomic library (RZPD, Germany) was screened twice: the first time with the *Gox-1 *probe produced by the above primers, and the second time with the *Glxr-2 *probe. The latter probe contained a mixture of two probes produced by the above primers (for *Glxr-2*) and the following set: 5'-GACGGGGCTCTCGCAACTG-3', 5'-GGCGCACCTCCAGTAGTCGT-3'. Sequences for *Glxr-2 *primers were obtained from two fragments of the EST contig 59 found in the adult bee brain cDNA library [42]: BB160017A20G03 and BB170027B20D05. The 5' ends of *Gox-1 *and *Glxr-2 *genes were isolated by 5' RACE System (Invitrogen) and sequenced.

### Semi-quantitative RT-PCR

Total RNA was isolated using TRI Reagent (SIGMA) followed by DNase treatment (Ambion). To assess the gene expression patterns, we performed RT-PCR (Promega) of total RNA (1 μg) from different developmental stages of *A. mellifera *or *D. melanogaster *using appropriate gene-specific primer sets. For Drosophila GMC cluster genes, band signal intensity was compared visually and categorized to either undetectable ("-") or relative expression ("+" to "++++"). As control, reactions for *eIF2α *were run to confirm the uniform quantity and quality of samples. For *A. mellifera Gox-1 *and *Glxr-2*, RT-PCR products were subjected to Southern blot analysis and probed with each gene's fragment generated by the primers used for genomic library screening. Signal intensity was detected and analyzed by STORM scanner and ImageQuant (Molecular Dynamics) and was normalized to the level of *Ef1α *signals.

## Authors' contributions

KI discovered the GMC gene cluster in the four insect species, performed phylogenetic analyses, performed gene expression analysis of GMC genes, performed molecular analyses of the *Gox-1 *and *Glxr-2 *genes in honeybees, and contributed substantially to writing the manuscript. DLC initially discovered the large array of GMC genes in the genome sequences of *Anopholes gambiae *and the GLXr-2 EST in *Apis mellifera*, and contributed to the preparation of the manuscript. XY isolated developmental stages of honeybees for developmental analysis of gene expression. WYK performed most of the phylogenetic analyses for identification of GMC subfamilies. DRC directed the research project, advised on phylogenetic and developmental analyses, contributed substantially to the interpretation of the data and writing the manuscript. All authors read and approved the final manuscript.

## Supplementary Material

Additional File 1 Alignments. Animo acid sequence alignments for different GMC subfamilies and a combined alignment across all GMC subfamilies. Click here for file

Additional File 2 Pairwise distance matrices. The average pairwise distance for different GMC subfamilies and pairwise distance matrices within each subfamily. Click here for file

Additional File 3 Phylogeny of GMC genes. Phylogenetic trees of GMC genes based on a different method and substitution model. Click here for file

Additional File 4 Insect *Flo-2 *gene coding sequence. The predicted coding sequences of *Flo-2 *genes in fly, mosquito, bee, and beetle. Click here for file

Additional File 5 The predicted alternative splicing pattern of *A. gambiae *GMCβ4. The exon/intron sequences of *A. gambiae *GMCβ4 gene. Click here for file

Additional File 6 The alternative splicing pattern of *A. mellifera *GLXr-2. The 3' end exon/intron sequences of *A. mellifera *GLXr-2 gene. Click here for file

Additional File 7 Table of gene information. The information of gene sources. Click here for file

Additional File 8 Protein sequences used in our phylogeny. All protein sequences used in our phylogenetic analysis (in FASTA format). Click here for file

Additional File 9 Coding sequences. CDS of the manually annotated sequences used in this study. Click here for file
