# Supplementary material for: Expansion and evolution of insect GMC oxidoreductases
Source: BMC Evol Biol. 2007 May 11;7:75. doi: 10.1186/1471-2148-7-75 (PMC1891103; doi:10.1186/1471-2148-7-75)
Supplement: Additional File 1 — Alignments. Animo acid sequence alignments for different GMC subfamilies and a combined alignment across all GMC subfamilies. [file 1471-2148-7-75-S1.doc]

**Supplementary Material**

Amino acid sequence alignments for different GMC gene subfamilies and the combined alignment across all subfamilies are listed below. Please see the legend of Figure 3 for the details of sequence names.

**Amino acid sequence alignment within the GMC α subfamily**

#DmGMC_A1_CG9503_{GMC-A} ---------- ---------- ---------- --------MS SAIVGAASAI GGAVTAATSN SWFIPMLMAA VAYFQYEEII DPESKPSDVG GD----DILD

#AgGMC_A1_{GMC-A} ---------- ---------- ---------- ---------M SELVGAA--F GSVATAASSV GWFVPMLVAA IAYFQYEEFM DPEARVIDVP TE----IMLD

#AmGMC_A1_{GMC-A} ---------- ---------- ---------- ---------- MGIESVL--T GGLTSASSGL SWFFPVLAAA LVYFEYE-VM DNEAPPINIP SE----VLLP

#TcGMC_A1_{GMC-A} ---------- ---------- ---------- ---------M STITAPL--L GLGATAASNL AVFIPALAAA IAYFQYD-LL DPESRPIDVS TD----ELLE

#DmGMC_A1_CG9503_{GMC-A} HYDFIVIGAG SAGAVVANRL TEVENWNVLL LEAGGDE--- -----TELTD VPLMAGYLQL SK-IDWQYKT EPSG------ ---TSCLAMQ GGRCNWPRGK

#AgGMC_A1_{GMC-A} KYDFIIIGAG SAGAVLANRL TEVENWNVLL LEAGGDE--- -----TEISE VPLMAGYLQL SK-LDWKYKS EPSG------ ---TFCLAMN GGRCNWPRGK

#AmGMC_A1_{GMC-A} AYDFIVVGGG SAGAVVASRL SEIENWNVLL LEAGSDE--- -----TEISD IPLLAGYLQL SQ-LDWQYKT EPDG------ ---QSCLAMS NGRCNWPRGK

#TcGMC_A1_{GMC-A} RYDFIVVGAG SAGAVVANRL SEIEQWNVLL LEAGGDE--- -----MEISD VPLMAAYLQL SQ-IDWKYKS EPQG------ ---QACLAMK NGRCNWPRGK

#DmGMC_A1_CG9503_{GMC-A} VLGGSSVLNY MLYLRGSKHD YDNWEA-MGN PSWSYRDALY YFKKSEDNTN QYLANT---- ----PYHATG GYLTVGEAPY HTPLAASFVE AG-VEMGYE-

#AgGMC_A1_{GMC-A} VLGGSSVLNY MLYLRGNKKD YDNWEA-MGN TGWGYKDALY YFKKSEDNTN PYLANT---- ----PYHSTG GYLTVGEAPY HTPLAAAFVE AG-VEMGYE-

#AmGMC_A1_{GMC-A} VIGGSSVLNY MLYLRGNKKD YDIWES-QGN RGWSFKDVLY YFKKSEDNQN PYLTKT---- ----PYHATG GYLTVQEAPW HTPLATAFIQ AG-QEMGYE-

#TcGMC_A1_{GMC-A} VIGGSSVLNY MLYLRGNKKD YDIWES-LGN PGWGSQDALY YFKKSEDNQN PYLSRT---- ----PYHATG GYLTVSEAPY HTPLVAAFVE GG-RQLGYA-

#DmGMC_A1_CG9503_{GMC-A} -NRDLNG-EK MTGFMIAQGT TRR-GSRCST SKAFLRP-AR L-RPNLHISM NSHVTRIMID PVT--KLAFG VEFV------ -----KDQKL YHVRATKEVV

#AgGMC_A1_{GMC-A} -NRDLNG-AK QTGFMIAQGT IRR-GGRCST GKAFLRP-AR L-RPNLHVAM FAHVTRVMID PIS--KIAFG VEFI------ -----RDRKV HHVRASKEVI

#AmGMC_A1_{GMC-A} -NRDING-EQ QTGFMIAQGT IRR-GSRCST AKAFLRP-AR L-RKNLHIAM QSHVTKILID PKS--KRAYG VEFV------ -----RDQKM FRIRAKKEVI

#TcGMC_A1_{GMC-A} -NRDING-EH QSGFMMAQGT TRR-GSRCST GKAFLRP-VR L-RKNLHVAM HAHVTKVMVD PTS--KVAFG VEFV------ -----RDKKL YRIRATKEVV

#DmGMC_A1_CG9503_{GMC-A} LSGGSVNSPQ LLMLSGVGPR KELAKHRIPL IKELS-VGEN LQDHIGLGGL TFLVNQP--- ----VSIVEN RFHTMSTVLQ YAVFG---QG PLTILGGVEG

#AgGMC_A1_{GMC-A} VSGGSVNSPQ ILMLSGIGPK SELAKHRIPL IKDLP-VGEN LQDHIGLGGL TFMVNQP--- ----VSIVEN RYHSMSTVLQ YAVLG---QG PLTILGGVEG

#AmGMC_A1_{GMC-A} VSGGSINSPQ LLMLSGIGPR EHLSKHGIPV IQDLR-VGFN MQDHVGLGGL TFLVDKE--- ----ISMVEK RLHTVQTVMQ YAIFG---NG PLTVLGGVEG

#TcGMC_A1_{GMC-A} LSAGAVNSPQ LLMLSGIGPK EDLERLKIPL VQDLK-VGHN LQDHVGLGGL TFLINRP--- ----HSILLN RLYSVSSLMQ YAIFG---GG PLTIMGGVEG

#DmGMC_A1_CG9503_{GMC-A} LAYVNTK--- ---------- -YANSSLDWP DIEFHFV-SG STNSDGGSQL RKAHGLTDAF YRAVFEPINN --RDAWSIIP MLLRPRSVGN IRLRSGN-PF

#AgGMC_A1_{GMC-A} LAFVSTK--- ---------- -YVNATDDYP DIEFHFV-SG STNSDGGNQL RKAHGLTEAF YNTVFKPINN --MDAWSIIP MLLRPHSVGT IKLRSSN-PF

#AmGMC_A1_{GMC-A} LAFVNTK--- ---------- -YVNASDDFP DIELHFV-SG STNSDGGRQI RKIHGLTKRF YDAVYGALND --MDVWSVIP MLLRPKSKGV IKLRSKD-PF

#TcGMC_A1_{GMC-A} LAFVNTK--- ---------- -YVNASDDFP DIELHFI-SG STHSDGGTQL RKAHGLTDAF YERVFGPIAD --KDAWSVIP MLLRPKSRGF IKLRSKN-PL

#DmGMC_A1_CG9503_{GMC-A} D-YPYIFPNY LT--DDFDMK TLIEGVKIAV ALS-RTKAMQ RFGSRISSIR WPGCEQVPLF T--------- DAFWECMVRR YTSTIYHPVG TCKMGPYW--

#AgGMC_A1_{GMC-A} D-YPYIYPNY LH--DDRDMR TLVEGVKIAY ALS-RTQTMQ KYQSTLSAYK FPGCAHIQMF T--------- DLYWECMIRH YTCTIYHPVG TCKMGPYW--

#AmGMC_A1_{GMC-A} A-HPLIYPNY FN--EPEDIA TLVEGVKIAV ALS-RTQAFR RFGSEVNSKQ FPGCKNIPMY S--------- DPYWECMIRH YTVTVYHPVG TCKMGPYW--

#TcGMC_A1_{GMC-A} D-YPLIYPNY FK--DDFDMK TLIEGAKLSV ALS-QTPAFK YYKSTLH--K FPDCAGFKDY S--------- DEFYECMIRL YTVTIYHPVG TCKMGPYW--

#DmGMC_A1_CG9503_{GMC-A} ---DKDAVVD AKLR-VYGIR GLRVIDASIM PKLVSANTNA PVIMIAEKGS DMIKEFWIKN ---TIV---- ---------- ---------- ----------

#AgGMC_A1_{GMC-A} ---DKQAVVD PQLR-VYGVR GLRVIDASIM PKLVSANTNA PVIMIAEKGA DMIKDFWIKR ---GVA---- ---------- ---------- ----------

#AmGMC_A1_{GMC-A} ---DPEAVVD PELR-VYGIQ GLRVIDASIM PNLVSGNTNA PVIMIGEKGS DMIKEFWLKR RSRRIVAGFV K--------- ---------- ----------

#TcGMC_A1_{GMC-A} ---DQEAVVD PQLR-VYGIK GLRVIDASIM PNLVSGNTNA PAIMIGEKGS DLIKEFWIKT ---ARYGRFV ---------- ---------- ----------

#DmGMC_A1_CG9503_{GMC-A} ---------- ---------- ---------- ---------- ---------- ---------- ---------- ---------- ---------- ----------

#AgGMC_A1_{GMC-A} ---------- ---------- ---------- ---------- ---------- ---------- ---------- ---------- ---------- ----------

#AmGMC_A1_{GMC-A} ---------- ---------- ---------- ---------- ---------- ---------- ---------- ---------- ---------- ----------

#TcGMC_A1_{GMC-A} ---------- ---------- ---------- ---------- ---------- ---------- ---------- ---------- ---------- ----------

#DmGMC_A1_CG9503_{GMC-A} ---------- ---------- ---------- ---------- ---------- ---------- ---------- --

#AgGMC_A1_{GMC-A} ---------- ---------- ---------- ---------- ---------- ---------- ---------- --

#AmGMC_A1_{GMC-A} ---------- ---------- ---------- ---------- ---------- ---------- ---------- --

#TcGMC_A1_{GMC-A} ---------- ---------- ---------- ---------- ---------- ---------- ---------- --

**Amino acid sequence alignment within the GMC δ subfamily**

#DmGMC_D1_CG9514_{GMC-D} MVVVPALGAA AVSVGGLLFK ASAASKAAAA AGVAAAGASK LGLAIAGAIK LATAVIGVGK LTILPFLIAA IAYYNYD-LF DPENRPFNVQ Q------VDL

#AgGMC_D1_{GMC-D} ---------M VVSFG----- -TLIPLLAGA ALKATPAAAG LTTAVGAAIS AATAVIGVGK LAIVPILIAS LAYYNYD-LF DPENRPFNVP E------VDR

#AmGMC_D1_{GMC-D} ---------- ---------- ---------- -------MTL ISTATLA-VK AATLLIG--K LAIIPIIIAT LAYYNYD-LM DPENQPKVTK N------LRK

#TcGMC_D1_{GMC-D} ---------- ---------- ---------- -------MAI ILPTVLASIK AGVGIIGAGK IAILPFLLAA LAYFHYD-QF DPENRPVDRK V------VDK

#DmGMC_D1_CG9514_{GMC-D} AYDFIIIGGG SAGTVLASRL SEIPHWKILL LEAGGHE--- -----TEISD VPLLSLYLHK SK-MDWKYRT QPQP------ ---TACQAMK DKRCCWTRGK

#AgGMC_D1_{GMC-D} EYDFIVVGAG SAGAVVASRL SEIGGWKVLL LEAGGHE--- -----TEISD VPILSLYLHK SK-LDWKYRT QPQK------ ---TACQAMK DNRCCWTRGK

#AmGMC_D1_{GMC-D} EYDFVVVGGG SAGSVVVNRL TENPGWSVLL LEAGGHE--- -----TEITD VPILSLYLHK SK-LDWKYRT QPQD------ ---SACQAMV DRRCCWTRGK

#TcGMC_D1_{GMC-D} EYDFVVVGGG SAGSVIANRL TEIPSWKVLL LEAGGHE--- -----TEISD VPVLSLYLHK SK-LDWGYKT EPQT------ ---EACKAMI ENRSSWTRGK

#DmGMC_D1_CG9514_{GMC-D} VLGGSSVLNT MLYIRGNKRD FDQWAD-FGN PGWSYEDILP YFRKSEDQRN PYLARNK--- ----RYHGTG GLWTVQDAPY NTPIGPAFLQ AG-EEMGYD-

#AgGMC_D1_{GMC-D} VLGGSSVLNT MLYIRGNKRD FDLWQA-LGN PGWGYEDVLP YFRKSEDQRN PYLARNK--- ----RQHGTG GLLQVQDAPY LTPLGVSFLQ AG-EEMGYD-

#AmGMC_D1_{GMC-D} VLGGSSVLNT MLYIRGNRRD FDQWES-FGN PGWGYDDILH YFKKSQDQRN PYLARNT--- ----KYHSTG GYLTVQDSPY NTPLGIAFLQ AG-EEMGYD-

#TcGMC_D1_{GMC-D} VLGGSSVLNT MLYIRGNRRD FDHWVH-QGN PGWSYEEILP YFLKSEDQRN PYLARN---- ----KYHSTG GYQTVQDSPY STPLGVAFLQ AG-QEMGYD-

#DmGMC_D1_CG9514_{GMC-D} -IVDVNG-EQ QTGFGFYQFN MRR-GSRSST AKSFLRP-AR L-RPNLHVAL FSHVTKVLTD PHT--KRATG VQFI------ -----RDGRL QNVYATREVI

#AgGMC_D1_{GMC-D} -IVDVNG-EQ QTGFAFFQFT MRR-GTRCST SKAFLRP-VR N-RKNLHVAL FAHVTRVILD PET--RRALG VEFI------ -----RNGKT HKVFATREVI

#AmGMC_D1_{GMC-D} -IVDING-EQ QTGFALYQYT MRR-GTRCSA AKAFIRP-IQ L-RRNFDLSL WSHVTRILID PRT--KRARG VEFI------ -----RGGRR EVVHARKEVI

#TcGMC_D1_{GMC-D} -IRDVNG-EK QTGFAFFQFT MRR-GTRCST SKAFLRP-IR L-RKNLHISL WSHVTKVLID PES--RRAYG VEFI------ -----KNGKK QIVLARKEVI

#DmGMC_D1_CG9514_{GMC-D} LSAGAIGSPH LMMLSGIGHG EELGRVGIPL VQHLPGVGQN LQDHIAVGGI AFLIDYP--- ----ISIVMK RMVNINTALR YAITE---DG PLTSSIGLEA

#AgGMC_D1_{GMC-D} LSAGAIGTPH LMMLSGIGPR ENLERVGIPV FHDLPGVGQN LQDHIAVGGL VFRIDQP--- ----ISVIMN RLVNLNSALR YAVTE---DG PLTSSIGLEA

#AmGMC_D1_{GMC-D} LSAGAINSPQ LLMLSGIGPR RHLEELGIPV IHDSPGVGQN LQDHIAVGGI IFPIDYP--- ----ISIMLD RVVNLNSALR YAITE---DG PLTANVGLET

#TcGMC_D1_{GMC-D} LSAGAINSPQ LLMLSGVGPA EHLQEKGIRV IHDSPGVGQN LQDHIAVGGL TFLIDPP--- ----ISLLVN RLVNLNTALR YAIKE---DG PLTSSIGLEA

#DmGMC_D1_CG9514_{GMC-D} VAFINTK--- ---------- -YANASDDWP DMNFMMT-SA SVMSDGGSQV KTAHGLTDEF YQEVFGEVNN --RDVFGVFP MMLRPKSRGY IKLASKN-PL

#AgGMC_D1_{GMC-D} VGFISTK--- ---------- -YANQTDDWP DIEFMLT-SA STPSDGGDQV KKAHGLKDEF YEDMFSSINN --QDVFGVFP MMLRPKSRGF IRLQSRN-PL

#AmGMC_D1_{GMC-D} VGFISTK--- ---------- -YANRSDDWP DIEFMLT-SS SVNSDGGTHV KNAHGLTDEF YNEVFESINR --RDVFSVFP MLLRPRSRGF LKLRSSN-PL

#TcGMC_D1_{GMC-D} VGFIPTK--- ---------- -YTNQSDDWP DIEFMIT-ST STPADGGTQV KHAHGLTDEF YNEYFSEINY --KDTFAVFP MLLRPKSRGE IKLRSKN-PL

#DmGMC_D1_CG9514_{GMC-D} R-YPLLYHNY LT--HPDDVN VLREGVKAAV AMG-ETQAMK RFGARYWNKP VPNCKHLTLY T--------- DDYWNCFIRQ YTMTIYHMSG TAKMGPPT--

#AgGMC_D1_{GMC-D} R-YPLLYHNY LT--HPDDVG VLREGVKAAI AFG-ETQAMK RFGARFHSKQ VPNCRHLPEF T--------- DEYWDCAIRQ YTMTIYHMSG TAKMGPPD--

#AmGMC_D1_{GMC-D} D-YPLMYHNY LT--DPYDID VLREGVKAAI AFG-QTSSMR RFGARFHSHP VPNCKRIPLY T--------- DEYWNCAIRQ YTMTIYHMSC TAKMGPRT--

#TcGMC_D1_{GMC-D} D-YPLLQPNY LT--DLHDVW VMREGAKAAV AFA-QTESMK RFGTRFYSKP LPNCKHLPLF T--------- DEYWDCAVRQ YTLSIYHYSC TAKMGPAE--

#DmGMC_D1_CG9514_{GMC-D} ---DPWAVVD PQLR-VYGIP GLRVIDASIM PAITNGNIHA PVVMIGEKGA DMIKQLWLTP TTAPVGVQGQ GPSPRQGHNT SPAPPPRTQW RSKRSLNSTE

#AgGMC_D1_{GMC-D} ---DPWAVVD PKLR-VYGIK GLRVIDASIM PRITSGNINA PVIMIGEKGA DMIKELWLK- ---------K GHS-RRGKRQ QFAN------ ETLSAANQTE

#AmGMC_D1_{GMC-D} ---DPMAVVD PELR-VYGVN GLRVIDASIM PTITSGNINA PVIMIGEKGA DLVKEQWWH- ---------- ---------- ---------- ESRT------

#TcGMC_D1_{GMC-D} ---DPYAVVD PELR-VYGVA GLRVIDASIM PTITNGNLNA PTIMVGEKGA DLVKYYWLQ- ---------- ---------- ---------- PSTRRRRDLR

#DmGMC_D1_CG9514_{GMC-D} ADTETENSEN LDIGTSPVHQ WPLPRS---- ---------- ---------- ---------- ---------- ---------- ---------- ----------

#AgGMC_D1_{GMC-D} AIEAVGDS-- ---SAAPCAN ETFVS----- ---------- ---------- ---------- ---------- ---------- ---------- ----------

#AmGMC_D1_{GMC-D} ---NATIG-- -----S---- ---------- ---------- ---------- ---------- ---------- ---------- ---------- ----------

#TcGMC_D1_{GMC-D} AVYNVRLG-- -----ATCPN ITSI------ ---------- ---------- ---------- ---------- ---------- ---------- ----------

#DmGMC_D1_CG9514_{GMC-D} ---------- ---------- ---------- ---------- ---------- ---------- ---------- --

#AgGMC_D1_{GMC-D} ---------- ---------- ---------- ---------- ---------- ---------- ---------- --

#AmGMC_D1_{GMC-D} ---------- ---------- ---------- ---------- ---------- ---------- ---------- --

#TcGMC_D1_{GMC-D} ---------- ---------- ---------- ---------- ---------- ---------- ---------- --

**Amino acid sequence alignment within the GMC ε subfamily**

#DmGMC_E1_CG9517_{GMC-E} ---------- ---------- ---------- --------MA FG-------- -----TMTSL LGMIPLLAIG MNFYRYQ-SV DPENKVQEPT V------IRR

#AgGMC_E1_{GMC-E} ---------- ---------- ---------- --------MA LGLASVAAVA GGLAHTPIAL LTLIPLLAVG VNYYRYQ-SV DPETNPTDQQ T------LRR

#AmGMC_E1_{GMC-E} ---------- ---------- ---------- --------MA IGLTTLFSAT -SILG----- FTLIPLVAIG LTIYKYN-QE DPESHLFDTK Q------LLR

#TcGMC_E1_{GMC-E} ---------- ---------- ---------- ------MLAT IGQVAHFAG- -SLTGNPLAI IGLVPIFAAG LAFMRYV-SI DPEAHPVNVR H------VRP

#DmGMC_E1_CG9517_{GMC-E} QYDFVVIGGG SAGAVVANRL SEVRNWTVLL LEAGGDE--- -----TEISD VPALAGYLQL TE-LDWKYQT TPSST----- --RQYCQAMK GDRCFWPRGK

#AgGMC_E1_{GMC-E} YYDFVVIGAG SAGAVVASRL SEIGDWSVLL LEAGGDE--- -----NEVTD VPSLAGYLQL TE-YDWKYQT TPSAD----- --RRYCQAMI GDRCNWPRGK

#AmGMC_E1_{GMC-E} MYDFIVVGGG SAGAVVASRL SEVSNWTVLL LEAGGDE--- -----TEISD VPLLSGYMQL TD-MDWKYQT SPPTT----- --SAYCLAMI GDRCNWPRGK

#TcGMC_E1_{GMC-E} EYDFIVVGGG SAGAVVASRL SEIANWTVLL LEAGGDE--- -----NEISD IPALSGYTQM SQ-FDWMYQT SPPGD----- --SPYCLAMI GDRCNWPRGK

#DmGMC_E1_CG9517_{GMC-E} VLGGSSVLNA MVYVRGSKND YNHWAS-LGN PGWDYDSMLK YFLKSEDVRN PYLAKT---- ----PYHETG GYLTVQEAPW RTPLSIAFLQ AG-IEMGYE-

#AgGMC_E1_{GMC-E} VMGGSSVLNA MVYVRGNRLD YDQWQE-QGN VGWGYENVLP YFIKSEDNRN PYMARS---- ----PYHGVG GYLTVQEAPW RTPLSVAFVA AG-QEMGYE-

#AmGMC_E1_{GMC-E} VLGGSSVLNA MVYVRGNRRD YDNWAR-LGN TGWSYEDVLP YFLKSEDNRN PYLART---- ----PYHATG GYLTVQESPW RSPLSIAFLQ AG-QELGYA-

#TcGMC_E1_{GMC-E} VLGGSSVLNA MIYIRGNRHD YDQWAA-MGN TGWSYPEVLP YFLKSEDNRN PYLART---- ----KYHNTG GYLTVQESPW RTPLSIAFLQ AG-RELGYE-

#DmGMC_E1_CG9517_{GMC-E} -NRDING-AQ QTGFMLTQST IRR-GARCST GKAFIRP-VR Q-RKNFDVLL HAEATRILFD -KQ--KRAIG VEYM------ -----RGGRK NVVFVRREVI

#AgGMC_E1_{GMC-E} -NRDING-AE QTGFMLLQAT IRR-GSRCST SKAFLRP-VR L-RKNLHIAM NAHVTRILFD -DQ--HRAYG VEFV------ -----RHQKR QYVFARKEII

#AmGMC_E1_{GMC-E} -NRDVNG-AY QTGFMLNQGT IRR-GSRCST AKAFLRP-VK N-RPNLHVAM KTQALRIVFN -EG--RRATG VEVL------ -----RYGRH HFIRTRREIV

#TcGMC_E1_{GMC-E} -VRDLNG-EK QTGFMLSQGT IRR-GSRCST SKAFLRP-VK S-RQNLHIAM YSQVTKVMID PKT--KTAYG VKFT------ -----RNNRP QTVRARREVI

#DmGMC_E1_CG9517_{GMC-E} ASAGALNTPK LLMLSGVGPA EHLQEHNIPV ISDLP-VGNN MQDHVGLGGL TFVVDAP--- ----LTVTRN RFQTIPVSME YILRE---RG PMTFSG-VEG

#AgGMC_E1_{GMC-E} LSAGALNTPQ ILMLSGVGPA DHLDELGIPV VSDLP-VGDN LQDHVGLGGL TFLVDQP--- ----VTVKTS RYSSVPVALE YFLNE---RG PMTFPG-IEG

#AmGMC_E1_{GMC-E} LSAGAINTPQ LLMLSGIGPK EHLAEFGIPV ISDLR-VGDH LQDHVGLGGL TFVIDEP--- ----VSLKRD RFQTLSVMMQ YVLHE---RG PMTDSG-VEG

#TcGMC_E1_{GMC-E} LSAGAIGTPH ILMLSGVGEK SHLESFKIPV MSDLK-VGYN LQDHIGLGGL TFVIDDP--- ----ITFTKT RYQTFAVAME YIVNE---RG PMTSLGGVEG

#DmGMC_E1_CG9517_{GMC-E} VAFLNTK--- ---------- -YQDPSVDWP DVQFHFC-PS SINSDGGEQI RKILNLRDGF YNTVYKPLQH --SETWSILP LLLRPKSTGW VRLNSRN-PQ

#AgGMC_E1_{GMC-E} VAFVNTK--- ---------- -YADPSGKWP DIQFHFG-PS SVNSDGGQNI RKILNLRDGF YNTVYKPIQN --AETWTILP LLLRPKSTGW VRLRSKN-PF

#AmGMC_E1_{GMC-E} VAFVNTR--- ---------- -YADKMDDYP DIQFHFL-PS SINSDG-EQI KKILGLRESV YNTMYKPLTG --ADTWSILP LLLRPKSSGW IRLKSRN-PL

#TcGMC_E1_{GMC-E} LAFVNTK--- ---------- -YAPKSGSWP DIQFHFA-PS SINSDG-EQV KKITGLRDSV YNTVYKPLKN --AETWTILP LLLRPRSTGW VRLKSKD-PN

#DmGMC_E1_CG9517_{GMC-E} H-QPKIIPNY FA--HQEDID VLVEGIKLAI NVS-NTQAFQ RFGSRLHNIP LPGCRHLPFQ S--------- NEYWACCIKE FTFTIYHPAG TCRMGPSW--

#AgGMC_E1_{GMC-E} V-QPSIEPNY FA--HEEDVA VLVEGIKIAI NVS-YTQAFQ RFNSRPHAIP LPGCRHLPFM S--------- DAYWACTIKQ FTFTIYHPAG TAKMGPSW--

#AmGMC_E1_{GMC-E} V-YPDINPNY FT--RKEDVD VLVDGIRIAM SVS-NTTAFR RFGSRPHTIR MPGCHRYPFD T--------- YDYWECAIRH FTFTIYHPVG TCKMGPRS--

#TcGMC_E1_{GMC-E} I-YPDINPNY FT--HKEDIL TLTEGIRIAL NVS-NTQSFQ RFNSRPHKIP FPKCRQYDWD S--------- DEYWECSLRH FTFTIYHPTS TAKMGPAS--

#DmGMC_E1_CG9517_{GMC-E} ---DVTAVVD PRLR-VYGVS GVRVVDASIM PTIVNGNPNA PVIAIGEKAS DLIKEDWGVR RAHTSA---- ---------- ---------- ----------

#AgGMC_E1_{GMC-E} ---DPGAVVD PRLR-VYGVS GLRVVDASIM PTIISGNPNA PVIMIGEKAA DMIKEDWGRL VGW------- ---------- ---------- ----------

#AmGMC_E1_{GMC-E} ---DPTAVVD PRLR-VYGVK GLRVADGSIM PEIVSGNPNA PIIMIGEKAS DMVKEDW-MR ---------- ---------- ---------- ----------

#TcGMC_E1_{GMC-E} ---DPDAVVD PRLR-VYGIK GLRVIDASIM PTIVSGNTNA PTIMIGEKGS DMIKQDWGIR V--------- ---------- ---------- ----------

#DmGMC_E1_CG9517_{GMC-E} ---------- ---------- ---------- ---------- ---------- ---------- ---------- ---------- ---------- ----------

#AgGMC_E1_{GMC-E} ---------- ---------- ---------- ---------- ---------- ---------- ---------- ---------- ---------- ----------

#AmGMC_E1_{GMC-E} ---------- ---------- ---------- ---------- ---------- ---------- ---------- ---------- ---------- ----------

#TcGMC_E1_{GMC-E} ---------- ---------- ---------- ---------- ---------- ---------- ---------- ---------- ---------- ----------

#DmGMC_E1_CG9517_{GMC-E} ---------- ---------- ---------- ---------- ---------- ---------- ---------- --

#AgGMC_E1_{GMC-E} ---------- ---------- ---------- ---------- ---------- ---------- ---------- --

#AmGMC_E1_{GMC-E} ---------- ---------- ---------- ---------- ---------- ---------- ---------- --

#TcGMC_E1_{GMC-E} ---------- ---------- ---------- ---------- ---------- ---------- ---------- --

**Amino acid sequence alignment within the GMC ι subfamily**

#DmGMC_I1_CG9522_{GMC-I} ---------- ---------- ---------- ------MEKL LLTQLLFLLL IPLIHSQRS- ----SQLDEL RRLGLGN-VV NVPFFSDVPQ ---------R

#DmGMC_I2_CG12539_{GMC-I} ---------- ---------- ---------- ------MQST QISQLFLLLH LLIFTTVARG DVNRLVLDQL NQVGLVN-LI EQATRPNVPR D-------LS

#AgGMC_I4_{GMC-I} ---------- ---------- ----MKHLWI AVILIATHSA LTANGFFLLL KTLAHAGRYI NEHYPDEG-- ---------- -----INYRQ S-------VP

#AgGMC_I3_{GMC-I} ---------- ---------- -------MGV LQDLLRVHDG -NGRLLFLVF LCLYLTVRCS VCQCPDTGGL GAEDPANVRL LQENSIKQAS L-------LK

#TcGMC_I5_{GMC-I} ---------- ---------- ---------- ----MLKKFL ILTALLSAIS PTSAENVDEF LAKVKKNYDN AKRSKRFIDP YEYPGAEQPL ------DEMS

#TcGMC_I6_{GMC-I} ---------- ---------- ---------- --MFHSLIWA SFLAIFFVLR PVSSN----- YDDININTIT ERFGTIFGTT DPIN--IIPN -----HKIDE

#TcGMC_I7_{GMC-I} ---------- ---------- ---------- ----MKLQTV FLLTLLFLVK SDDSENLQYI YDELSAVYGQ YSNKTFFLTP DQWAEWTLPK DPGKRESPKS

#DmGMC_I1_CG9522_{GMC-I} NYDFIIVGGG AAGCTLAARL SENPNWSVFL IEAGGVE--- -----NIVHQ VPLLAAHLQS TA-SNWGYNS TP-------- -QRHACRGMP DNKCALPRGK

#DmGMC_I2_CG12539_{GMC-I} NYDFIVIGAG AAGCTLAARL SENPQVSVAL IEAGGVE--- -----NIAHL TPVVAGYLQQ TS-SNWGYKS VP-------- -QKLSCHGMN NNECALPRGK

#AgGMC_I4_{GMC-I} EYDFIIVGAG AAGCVLANRL SENPQWKILL LEAGPGE--- -----NDLQN IPLLTTFLQN SQ-YNWADIA EA-------- -QNTSCYGMI DQRCSLPHGK

#AgGMC_I3_{GMC-I} KYDFIIVGAS PSGCLLANRL TEIRDWNVLL IEAGEQE--- -----NLFVQ VPIFSAYLQS TS-YNWGYLA EP-------- -QNYSCWGMK DQRCSYPRGK

#TcGMC_I5_{GMC-I} KYDFIVVGSG SSGSVIANRL TET-NWTVLL LEVGEEA--- -----TPLTD IPVIAPLFQF TS-LNWNYLM EK-------- -QDNMCLGLE DQRMAWPRGR

#TcGMC_I6_{GMC-I} VYDFIIIGSG SSGSVVASRL SEIPTWKILL LEAGNAA--- -----NILTK VPIMAPLFQL TP-YNWNYTM EP-------- -EPNVCQAME EETCAWPRGK

#TcGMC_I7_{GMC-I} GYDFIVVGSG SSGAVIANRL SENPNWEVLL LEAGKGE--- -----NFFSQ IPLVCPTLAF TH-YNWDFIA EY-------- -QPNVSFGFE NNRMRWPRGR

#DmGMC_I1_CG9522_{GMC-I} VLGGTSSINY MIYNRGNRRD FDAWAA-AGN PGWSYDEVLP YFLRSEHAQL QGLE-HS--- ----PYHNHS GPLSVEDVRH RTRLAHAYIR AA-QEAGHP-

#DmGMC_I2_CG12539_{GMC-I} ILGGTSSINY MIYNRGNRRD FDAWAA-AGN PGWSYDEVLP YFLRSEHAQL QGLE-QS--- ----PYHNHS GPLSVEYVRF RSQMVDAFVE AS-VESGLP-

#AgGMC_I4_{GMC-I} GLGGSTLIDY MLYGRGNPAD YDRWAA-QGN PGWSHADLFP YFLKSERAEL RGLE-NS--- ----TYHGKS GELHVEFPTF RTNLARTFVN GA-REAGHR-

#AgGMC_I3_{GMC-I} GLGGSTLINY MMYVRGNKYD YDQWSA-AGN DGWSFDEILP YFVKSEKSYL R--E-VN--- ----RYHGMD GNLDVRYLPY RTRLAKLFVN AW-RELGLE-

#TcGMC_I5_{GMC-I} GLGGSTLINY MIHVRGNRRD YNRWAK-MGN PGWSYHDIFQ YFLKSEDFLV RKQD--P--- ----GYHTTG GYLGVQDVPY RTQSAHAFVQ AA-QEAGHK-

#TcGMC_I6_{GMC-I} ALGGTSVINY MIYTRGNPLD YQKWG--EVS PGWAFQDVLP YFLKSENCNL GTAC-GS--- ----EYHNKG GPLSVEYP-F KSPITDAFLQ AG-REMGEE-

#TcGMC_I7_{GMC-I} ALGGTSVINF MIYTRGNRHD YDRWAG-QGN PGWSYRDVLP YFIKSERSTL NNPH--P--- ----GVHGTN GYLGVSDI-Y QSEILRAFIE GG-NELGLP-

#DmGMC_I1_CG9522_{GMC-I} -RTDYNG-ES QLGVSYVQAT TLK-GRRHSA FRAYIEP-IR SRRHNLHILT LARVTRVLID AAT--KSAYG VELT------ -----HQGRS FKVKARKEII

#DmGMC_I2_CG12539_{GMC-I} -RTDYNG-ES QLGVSYVQAN TLN-GRRHSA YSAYIKP-VR DLRSNLQIFT FSQVTRILID EAT--KSAYG VEFH------ -----YKNKA YTFKARKEVI

#AgGMC_I4_{GMC-I} -KLDYNG-KS QLGVSYVQTT GLR-GMRQTA YRAFVEP-VL YKRPNLHVQP YSQVLKVLIN PDT--QTAYG VTYT------ -----RHFRN YEVRARKEVI

#AgGMC_I3_{GMC-I} -SVDYNG-ES QIGVSYIQSN VRN-GRRLTA YTAFLEP-IL D-RPNLHILT NARATRVLID ATT--QQAYG VEFI------ -----KDRNR YTVYADKEIL

#TcGMC_I5_{GMC-I} -FVDYNG-KR QMGVSYVHAT TRN-GKRSSA EEAFLRP-IK H-RQNLKIST KSRVTKVLID PQT--RQAYG VQYI------ -----KNGKY HTVLASKEVI

#TcGMC_I6_{GMC-I} -IVDYNT-EK YMGFGQLQAN QKF-GRRHST FDAFIAP-II T-RKNLHIVS GARVTKILID PNT--RQTLG VIFE------ -----KKGQK YKIRASKEVI

#TcGMC_I7_{GMC-I} -YFDYNANEK SFGVSPIQAT VKR-GRRHTT ARAFLHP-IR H-RKNLHMLT SAFVTKVLID PNT--RQTYG VEFS------ -----RFGRK YQVTASKEVI

#DmGMC_I1_CG9522_{GMC-I} LSAGAFNSPQ LLMLSGIGPE DNLKAIGIPL IKALP-VGKR MFDHMCHFGP TFVTNTT--- ----GQTLFA A-QLGAPVAK EFLLG-RADT FLSSIGGVET

#DmGMC_I2_CG12539_{GMC-I} LSAGSFNSPQ LLMLSGIGPE DNLRGIGIPL IKALP-VGKR MFDHMCHFGP TFVTNTT--- ----GQTTFT S-RVTPAELI SFLLAGNPAT RMSSIGGVEA

#AgGMC_I4_{GMC-I} VTAGNINTAQ LLLLSGIGPR EHLQNFNLPL VSNLP-VGQS FVDSPVFNGL TFVLNET--- ----GQALLT DSRFQLRSLG DYFRG---EG PLTVPGGVEA

#AgGMC_I3_{GMC-I} MTAGALQTPQ LLMLSGVGPK EHLQEVGIPV IKDLP-VGQT LYDHIYFTGL AFVTNTT--- ----NLSLHG DNVLTLDAFL SFLQG---QG PMTVTGGVEA

#TcGMC_I5_{GMC-I} LSAGAFNSPQ ILMLSGIGPQ KHLQELGIPV LEDLP-VGQK MYDHITFLGL VFQVNES--- ----IVSDQK LLESPESFLQ LVLKN---NG PLTTLGGVEA

#TcGMC_I6_{GMC-I} LSAGVFNSPQ LLMLSGVGPE GHLHDLGIPP IVNLP-VGQN LYDHLAFLGV AYTINVT--- ----VEP-RE ALLSPLEGLN WFFRG---KG LYTSLGGVEA

#TcGMC_I7_{GMC-I} LSAGTFNSPK LLMLAGIGPR DHLAEMGIPL LEDLP-VGQN LHDHLTYPGL SFIIDKP--- ----LSLSVL HLINPKNIID FLFNG---TG PYTSLGGVGG

#DmGMC_I1_CG9522_{GMC-I} LTFIK----- ---------V PSGKSPATQP DVELIQV-AG SLASDDGTAL AKGANFKPEI YEKMYKNLTL RQQDHFSFLI MHFKPASVGR LWLHNRN-PL

#DmGMC_I2_CG12539_{GMC-I} LAFLK----- ---------T QRSNLPNDWP DIELIMV-TG SLASDEGTGL KLGANFKDEI YDRMYRELAQ AQQDHFTLLI MQFHPKSVGR LWLKDRN-PL

#AgGMC_I4_{GMC-I} ISFVR----- ---------T ENATTEPGVP NIAIVFS-TG SLVSDGGLGL RKGKRIKTAI YNKVYRPLET LRNDQWTASV VLLHPESRGH LKLRSIN-PY

#AgGMC_I3_{GMC-I} VAFIRNTTN- ---------P ESAATPTVLP NIEYILT-GG SQAADHGSGI RNGFRLTDTI YS-IYKPLEA NERDAMTVNI VLLHPKSKGY MRLKSCN-PL

#TcGMC_I5_{GMC-I} LLYFKTN--- ---------- -VSKGPAPYP DMELIFI-SG SMNTDLGKYY RKTFRITDEV YNTVWKPLEN --KYTFSVLP MLVHPESYGH LELKSTN-PF

#TcGMC_I6_{GMC-I} IAYINT---- ---------- -GSLPQANYP DIELIFVGTG TLQSDFGLVV AKEIRLKRSI YDTVYKPIEN --TPSWAIFP MLLHPQSKGH LQLKSTN-PH

#TcGMC_I7_{GMC-I} IGYIKTK--- ---------- -ESLEVEDIP DIELLFL-DG SLSTDYGLWN RRWMNIRDDV YYPVYGPTHN --IPTWTIFP MLLHPKSTGY LKLKSRN-PR

#DmGMC_I1_CG9522_{GMC-I} E-WPRIDPKY FS--APSDVE NLLEGIKEAL RISK-MPAMQ AIGTRLLDKP VPGCENYEFA S--------- DDYWRCSIRT LSYTLHHQVA TCRMGAES--

#DmGMC_I2_CG12539_{GMC-I} G-WPKIDPKY FV--AEEDVE YLLDGIKASL RIIE-MPAMQ RIGARLLKRT VPGCEGHQFA S--------- DDYWRCSIRT LSYTLHHQVA TCRMGAES--

#AgGMC_I4_{GMC-I} S-ALKIYPGY FG--ADRDVE TMLEGIKEAV RISK-SPAMR RYDARVLGIP LPNCEQWDQR E--------- DEYWRCAIRT LSSTAYQQLG SCRMGPAG--

#AgGMC_I3_{GMC-I} H-WPRFYSNM LK--EQEDVE TILQGIRSAL PLMD-TRAAR RYGAKLYDVP LPNCASFRFG T--------- DDYWRCAIRT QTTSIHHQIA TCKMGPPS--

#TcGMC_I5_{GMC-I} H-WPRFYGNY FTDRDNTDIK TFIAAIREVQ RIAK-MPTWQ KYGVRQVTTK IPGCQNFVFD S--------- DDYWECALRH VTTTLHHQVA TCKMGPKT--

#TcGMC_I6_{GMC-I} D-PPILHGNC FTDPGDQDIK TLLASIRYIQ KLAQ-TPSFQ KFGSKLHDIP LPTCQKHVFD S--------- DDYWLCAIKS LSTTLHHQVG TCRMGHWD--

#TcGMC_I7_{GMC-I} D-YPLLYGNY FTDPAQQDLK TMLAAIRYIQ KLAN-TRPFQ EMGTRMNPNP IPVCAHLIFD S--------- DAYWMCAIRA ISVTLHHQVG TAKMGPKD--

#DmGMC_I1_CG9522_{GMC-I} ---DPTTVVN HQLK-VHGVR KLRVVDTSII PFPPTAHTNA AAFMIGEKAA DMIRSEWS-- ---------- ---------- ---------- ----------

#DmGMC_I2_CG12539_{GMC-I} ---DPTTVVN HQLK-VHGVR KLRVVDTSII PFPPTAHTNA AAFMIGEKAA DMIRTDWELI ---------- ---------- ---------- ----------

#AgGMC_I4_{GMC-I} ---DPLAVVA PDLR-VHGVQ GLRVADVSVV PTTISAQSAA IDYMIGERAA DIIKDQWEQG SSAPTSSSDR ---------- ---------- ----------

#AgGMC_I3_{GMC-I} ---DPDAVVS SNLK-VYGVR RLRVADVGVI PYPTSGHPTA TAYMIGEKLS DLIKNEW-LG QNIPTGSGAG GI-------- ---------- ----------

#TcGMC_I5_{GMC-I} ---DPEAVVD PELR-VYGVR GLRVADTSVI PIPLTAHTNV PAFMVGEKAA DLIKETWRGA ---------- ---------- ---------- ----------

#TcGMC_I6_{GMC-I} ---DPQSVVD PRLR-VRGVK GLRVIDSSVI PVTLSAHTNA PSIMVGEKGA DLVKEDWSAI ---------- ---------- ---------- ----------

#TcGMC_I7_{GMC-I} ---DPTAVVN HELK-VYGVK GLRVADCSVI PFALGAHTNA PAIMVGEKAA DLIKADWEKV AR-------- ---------- ---------- ----------

#DmGMC_I1_CG9522_{GMC-I} ---------- ---------- ---------- ---------- ---------- ---------- ---------- ---------- ---------- ----------

#DmGMC_I2_CG12539_{GMC-I} ---------- ---------- ---------- ---------- ---------- ---------- ---------- ---------- ---------- ----------

#AgGMC_I4_{GMC-I} ---------- ---------- ---------- ---------- ---------- ---------- ---------- ---------- ---------- ----------

#AgGMC_I3_{GMC-I} ---------- ---------- ---------- ---------- ---------- ---------- ---------- ---------- ---------- ----------

#TcGMC_I5_{GMC-I} ---------- ---------- ---------- ---------- ---------- ---------- ---------- ---------- ---------- ----------

#TcGMC_I6_{GMC-I} ---------- ---------- ---------- ---------- ---------- ---------- ---------- ---------- ---------- ----------

#TcGMC_I7_{GMC-I} ---------- ---------- ---------- ---------- ---------- ---------- ---------- ---------- ---------- ----------

#DmGMC_I1_CG9522_{GMC-I} ---------- ---------- ---------- ---------- ---------- ---------- ---------- --

#DmGMC_I2_CG12539_{GMC-I} ---------- ---------- ---------- ---------- ---------- ---------- ---------- --

#AgGMC_I4_{GMC-I} ---------- ---------- ---------- ---------- ---------- ---------- ---------- --

#AgGMC_I3_{GMC-I} ---------- ---------- ---------- ---------- ---------- ---------- ---------- --

#TcGMC_I5_{GMC-I} ---------- ---------- ---------- ---------- ---------- ---------- ---------- --

#TcGMC_I6_{GMC-I} ---------- ---------- ---------- ---------- ---------- ---------- ---------- --

#TcGMC_I7_{GMC-I} ---------- ---------- ---------- ---------- ---------- ---------- ---------- --

**Amino acid sequence alignment within the GMC κ subfamily**

#AgGMC_K1_{GMC-K} ---------- ---------- ---------- --------MA AQRTSASTVV PTDAATQESY R--------- ---------- ---------- ---------T

#TcGMC_K2_{GMC-K} ---------- ---------M LTLATKLLLL G--VVSTVLS SPFKLTEQYI EEFKTGIESL KKLAH----- -EHKFAEKNF DFNLHNGSEP -----VPDDI

#TcGMC_K3_{GMC-K} ---------- ---------M LRSCVLFLIF SCAFASHYDT WPFTLADKYI EEFKTNVEEF KKFAHTYQHH EEIKYEVEEQ DLTEATKSEL HAPALITDAA

#AgGMC_K1_{GMC-K} VYDFIVVGGG TAGSVIASRL AELQQWHILL IEAGGG---- ---------- -------PSD KD-LSWNLQA QRQM------ ---GSCLGAP EQRCEIPTGR

#TcGMC_K2_{GMC-K} EYDFIIIGAG ASGSVIANRL TERPEWKVLL LEAGGPE--- -----TPYTR IPRLGHLLQN SD-YNWAYTT TPQK------ ---NWCKGMI DGSCAIAGGK

#TcGMC_K3_{GMC-K} HYDFIIVGGG TSGAILASRL SEIPEWKILL LEAGAPE--- -----TIATK VPKNWELLKN TP-YNWGYVT TPQN------ ---YSCLGMV DHKCVIPTGR

#AgGMC_K1_{GMC-K} GLGGNTLTNN MLYVRGSEAD YDAWAK-QTN VDWSYRNVLP YFLKLENFRK NASSTSR--- ----QQRGKG GPVPIAGLRE KSPLVRSFIS AC-NRLGLR-

#TcGMC_K2_{GMC-K} ALGGGTAING MMFTRGHPKD YDKWAD-LGN PGWCYNDVLP YFKKLEDADL KEFDHKY--- ----HNRG-- GPFHIEHPQH QTHLTHDVLQ AG-KELGLE-

#TcGMC_K3_{GMC-K} ALGGTTSINS MVYTRGNPRD YDLWSD-LGN EGWCWADVLP YYKKLEDAHF APFDKKY--- ----HHFG-- GPQHLEHPQY LRFLTDHTLE AA-KELDLH-

#AgGMC_K1_{GMC-K} -TTDYNA-ER NQTVGFVQLT QYR-TKRITA ADAYIRP-VK QLFNNLHIMS SARVTKVLIN GMN--RQAVG VKVL------ -----VNGKQ RKLRATKEVI

#TcGMC_K2_{GMC-K} -TIDYNG-KE QMGLGVLQMN SKH-GVRQST ATAYLEP-AE KRQN-LFVKP LSHVTKILIA PHT--KEATG VEYL------ -----HNDKL HIAKATKEII

#TcGMC_K3_{GMC-K} -LIDYNG-KH QIGISVPQLT SKC-GKRFST AEAYLER-AE KRDN-LIVKP LSQVLKVLIS THT--KEAQG VVYL------ -----HEGKT FVAKAEKEVV

#AgGMC_K1_{GMC-K} LSAGPIFTPH LLLLSGIGPR AQLDALQIPV LADLP-VGAT MNLRLVSFPL HLATNRT--- -------VPY AAQKMIEAIA FLN------- ----------

#TcGMC_K2_{GMC-K} LSAGALNTPQ ILMLSGIGPK EQLEKFEIPV VHELP-VGKH LKDHIGFYGL DLLYNGT--- -------EST PDPHYDEVID YLKNG----K GPLTTTGCEV

#TcGMC_K3_{GMC-K} LAAGALNTPK ILLLSGVGPK EDCEKLHIHH VADLK-VGHN LKIRPSFVGL DFLYTAE--- -------EAQ SHDEYHDILK YLKYG----K GPLTSPGIEA

#AgGMC_K1_{GMC-K} ---------- ---------- -TTKQNNTDP THEILFQ--- ---------- ---------- ----YEPRGT --LEYFSLGL IHLRPASRGF VQLNATN-PS

#TcGMC_K2_{GMC-K} VGYLQTE--- ---------- -ASKDQIKYP DIELFFSSRK VNAKP----S TNPFRLKPEI LDSLYKPIDG --KKIVNIGV MLTHPKSTGT VTLKDKD-PL

#TcGMC_K3_{GMC-K} LAFLKTN--- ---------- -ISKSPLTYP DIELKFLSRY HPQQD----L YSWMKPTPKH YDSLWKPLEA --HNCLKIIV TLNHPKSSGI VKLHTSN-PL

#AgGMC_K1_{GMC-K} R-NPVVYTNF FSAPN--DME EILSGITECL KIVH-SEEFT KLGLQSRKLI VPPCDKLRYG T--------- DEYWRCVVRH VGHAADQPYG TCPMGRQD--

#TcGMC_K2_{GMC-K} H-HPLVDPNQ LSDPEDHDLN TLLHGIRKAL AFAG-TETFK KLHLEVNEHP VAGCEEHKWG T--------- DEYWKCAIKH LSISLRHVSG TAKMGPGN--

#TcGMC_K3_{GMC-K} R-PPIIEPHF LSDEDEKDYH TILAGIKKAL KFSH-TEAFK KIGIKLNHHG VHGCEETEFG T--------- EAYWECAIKY LVVATEDVSG TARMGPES--

#AgGMC_K1_{GMC-K} ---NRQAVVS PELR-VHGIG NLRIADASVM LPVSNGHTQA TVYMIAEKAS DLIKSSWDWG NELERRR--- ---------- ---------- ----------

#TcGMC_K2_{GMC-K} ---DKEAVVD HELR-VHGVQ KLRVADASVI PVSVTGHTMA PAIMVGEKAA D--------- ---------- ---------- ---------- ----------

#TcGMC_K3_{GMC-K} ---DHYAVVD KKLR-VHGIH NLRVADASVI PVTMSGSLVG PTMVIGEKAA HIIMEEWLEH ---------- ---------- ---------- ----------

#AgGMC_K1_{GMC-K} ---------- ---------- ---------- ---------- ---------- ---------- ---------- ---------- ---------- ----------

#TcGMC_K2_{GMC-K} ---------- ---------- ---------- ---------- ---------- ---------- ---------- ---------- ---------- ----------

#TcGMC_K3_{GMC-K} ---------- ---------- ---------- ---------- ---------- ---------- ---------- ---------- ---------- ----------

#AgGMC_K1_{GMC-K} ---------- ---------- ---------- ---------- ---------- ---------- ---------- --

#TcGMC_K2_{GMC-K} ---------- ---------- ---------- ---------- ---------- ---------- ---------- --

#TcGMC_K3_{GMC-K} ---------- ---------- ---------- ---------- ---------- ---------- ---------- --

**Amino acid sequence alignment within the GMC λ subfamily**

#AmGMC_L1_{GMC-L} ---------- ---------- ---------- ---------- ---------- ---------M SWIPPDLATL CQPHSTVSTC QPPAFMFLAL IAH-----LE

#TcGMC_L1_{GMC-L} ---------- ---------- ---------- ---------- ---------- ---------M SWIPPNIAES CAVYTNLTSC QPPTDEYHQP RG-------R

#AmGMC_L1_{GMC-L} EYDFIIVGAG SAGCVLANRL SEVKHWKILL LEAGIEE--- -----PLVAD VPAFASMLQA SN-IDWMYRT QPER------ ---HSCRSRR DRSCAWARGK

#TcGMC_L1_{GMC-L} EYDFIVLGAG SAGCVLANRL TEIPSWSVLL LEAGDEE--- -----PEVAD VPAFAPVLQQ SS-IDWGFST QPDP------ ---NSCLARQ NGQCSWARGK

#AmGMC_L1_{GMC-L} VMGGSSTINY MIYIRGNPND YNEWAK-KGN YGWSYEEVLP YFLKSENNKD REIVKENP-- ----YYHNEG GYQSVERFPY TDINAKILLN AW-QELGHV-

#TcGMC_L1_{GMC-L} VMGGSSTINY MIYIRGNPRD YDEWAE-AGN PGWSWREVLP YFMKSEDNHN IDTVER---- ----QAHGVG GYLSVERFQF QENNVRSLFE AF-QELGLP-

#AmGMC_L1_{GMC-L} -TVDANA-GT QLGVMKLQMT SLH-GKRESV NSAYIRP-IR HKRKNLTIET QAHVTRLLTD PTT--KRVTG VDYTCT---- -----STGLS KSVLARKEVI

#TcGMC_L1_{GMC-L} -VVDQNA-GR QIGTMMLQTT TRS-GRRESA NLAFIRP-IR RKRKNLTIET KAYIIRVLID PHT--KVAYG VEYE------ -----KNGKL FQARARKEVL

#AmGMC_L1_{GMC-L} LSAGAINSPK ILMLSGIGPA DELKKHGIPV ISDLP-VGRN LQDHVTMDGL VIALNS---- ----TSTTKD NRMKKNDICY YEKTQ---MG PLSATGTLVC

#TcGMC_L1_{GMC-L} VTCGTIMTPK VLMLSGVGPA QHLQNLGIQV IKDLP-VGYN LMDHPTIDGV MFQISNE--- ----SATLVE PEQITRDVFY YREEQ---AG PLSSTGPLQV

#AmGMC_L1_{GMC-L} GAFLQTA--- ---------- --FEHEHGLP DIQYAFD--- ------ASNQ MDFLNDPAEF GETRVEPLSY --YDAINIRP ILLSPRSKGF LLLNDTD-PL

#TcGMC_L1_{GMC-L} NTFVQTK--- ---------- --YELEPGRP DIQYSID--- ------TANV VDYVTDLILA STTKVSPLSY --YNGFIIRP ILLNPVSRGV IKLNSTD-PI

#AmGMC_L1_{GMC-L} WGPPSIYPAY FT--AYPDAD VMVEGIETAL KLFH-TTWFR EYGFRLIDTP LPSCKRFIFG T--------- REYWKCAMME YTATIYHPVG TCKMGPDW--

#TcGMC_L1_{GMC-L} YGYPIIYANT FN--EQIDAL TMVEGIKQSL NLLK-TRAMQ RMGVSLITTP VAACDGYSFG T--------- EDYWLCLVRS YTSTMYHYAG TCKMGPKH--

#AmGMC_L1_{GMC-L} ---DSEAVVD PELR-VYGVA GLRVVDASIM PKIVRGNTNA PTIMIAEKAS DMIKDEWLYA WK-------- ---------- ---------- ----------

#TcGMC_L1_{GMC-L} ---DPFAVVD PKLR-VYGIK NLRVIDTSIM PRVTRGNTNA PTIMIAEKGA DFIKETWLKK KFSLPKIPKI PQIPKMDFKQ MIKTFFFNH- ----------

#AmGMC_L1_{GMC-L} ---------- ---------- ---------- ---------- ---------- ---------- ---------- ---------- ---------- ----------

#TcGMC_L1_{GMC-L} ---------- ---------- ---------- ---------- ---------- ---------- ---------- ---------- ---------- ----------

#AmGMC_L1_{GMC-L} ---------- ---------- ---------- ---------- ---------- ---------- ---------- --

#TcGMC_L1_{GMC-L} ---------- ---------- ---------- ---------- ---------- ---------- ---------- --

**Amino acid sequence alignment within the GMC θ subfamily**

#DmGMC_Q1_CG9519_{GMC-Q} ---------- ---------- ---------- ----MMTSPR NHIGTLLVVW LLEFSMVISQ TDTGNALMDM LE---IYRRG QAQLDLENLD EG---QVITT

#DmGMC_Q2_CG9521_{GMC-Q} ---------- ---------- ---------- ----MASKSL IFVG-LCLAY MATWSQISAQ NNN-NVLFET IN---FLRRG QADVELENYD NN---VILDS

#AgGMC_Q4_{GMC-Q} ---------- ---------- ---------- ----MQYLPL ---------- AAGILGMVSF SRPQDSLLSM LS---FLQDG GERMSHELPS Q----PVVRP

#AgGMC_Q3_{GMC-Q} ---------- ---------- ---------- ----MAQLP- ---------- ---------- -----GVQSI IQ---FYRDG DERLKYEKPD Q----RPLLP

#AmGMC_Q7_{GMC-Q} ---------- ---------- -------MFA TYKSSHNTHR LFFLLFLTAA LPNLSKSITH TGTENLITNV ISWNKFLNES LKFASRTQPD RT---PESNS

#TcGMC_Q6_{GMC-Q} ---------- ---------- ---------- ----MLKPVP ---------- --FFFSLFAL TSPQSLLDGL IN---FIEEG DAQSFNE-PP DT---PVLLP

#TcGMC_Q5_{GMC-Q} ---------- ---------- ---------- ----MLKTV- ---------- --ILLTIIAN TYQQSVLEGI IN---ILEEG EAQFNLE-PE DV---RNLLP

#DmGMC_Q1_CG9519_{GMC-Q} KYDFIVVGAG TAGCALAARL SENPRWRVLL LEAGGPE--- -----NYAMD IPIVAHLLQL GE-INWKYKT EP-------- -SNSYCLAMN NNRCNWPRGK

#DmGMC_Q2_CG9521_{GMC-Q} EYDFIVVGAG TAGCALAARL SENPQWRVLL LEAGGPE--- -----RLVMD VPIVAHFLQL GE-MNWKYRT QP-------- -SDHACLAMN NNRCNWPRGK

#AgGMC_Q4_{GMC-Q} EYDFIIVGAG SAGSVLANRL SEVPDWSVLL IEAGPGE--- -----NLLMD IPMAAHYLQN FN-INWDYRT KP-------- -SDQYCLAFK NNQCRFPRGK

#AgGMC_Q3_{GMC-Q} EYDFIIVGGG SAGCVLANRL TEISHWSVLL IEAGPRE--- -----NLLMD IPIFAHYLQG LS-INWDYRT KS-------- -SDQCCLAFK NNQCRLPRGK

#AmGMC_Q7_{GMC-Q} RYDFIVIGAG TAGATVASRL TEIQNLTVLL IETGLEE--- -----ELYMD IPLFANFLQR IPGLDWMYQT ES-------- -SDNYCRGMI GRKCRFPQGK

#TcGMC_Q6_{GMC-Q} SYDFIIVGAG TAGCVLANRL SENPSWNVLL LEAGRPE--- -----NYLMD LPVLANYIQF TD-ANWRYKT EP-------- -SDKFCLGME NQQCNWPRGK

#TcGMC_Q5_{GMC-Q} EYDFIVVGAG SAGCVVANRL SENPNWKVLL IEAGRTE--- -----NYLMD MPILANYLQF TD-SNWKYKT TP-------- -SGRFCMGMD NQQCKWPRGK

#DmGMC_Q1_CG9519_{GMC-Q} VMGGSSVLNY MMYTRGNRRD YDRWAR-LGN PGWSYEEVLP YFKKYEGSVV PDA--DE--- ----NLVGRN GPVKVSYSET RTRIADAFVG AT-QDAGLP-

#DmGMC_Q2_CG9521_{GMC-Q} VMGGSSVLNY MMYTRGNRRD YDRWEA-LGN PGWSFKDVLP YFKKYEGSSV PDA--EE--- ----DYVGRN GPVKVSYVNW RSKIAEAFVD AA-QQDGLK-

#AgGMC_Q4_{GMC-Q} VMGGSSVLNY MIYTRGNRRD FDHWAD-LGN PGWSYKEVLP YFKKLEHSVV PDA--NP--- ----AYAGKD GPLTISYPRF RSDTAKAFVQ GA-IEDGAP-

#AgGMC_Q3_{GMC-Q} VMGGSSVLNY MIYTRGNRRD YDAWAA-KGN AGWSFNDVLP YFQKLEKNIV PDS--HP--- ----MYAGRN GPVTISYPSY RTSVARAFVK AN-MELGLP-

#AmGMC_Q7_{GMC-Q} VMGGSSVINY MIATRGNKRD YDNWAK-MGN FGWSYDDVLK YFKRLENMMI PEYRNDT--- ----VHHGTK GPVTINYPRF ATTVARTFVE AG-HELGYP-

#TcGMC_Q6_{GMC-Q} VVGGSSVLNY MIYTRGNWRD YDKWAE-LGN EGWGFKDVLP YFKKIENFMV PGPY-NA--- ----SYHNHD GYLAVSYSPY KTKIADAVLE SA-QLMGLK-

#TcGMC_Q5_{GMC-Q} VVGGSSVLKY MIYTRENHRD YDHWAD-LGN TGWSFKEVLP YFKKVENFSV PDSP-YP--- ----EYHSKE GYLSVSYAPF KTKIADAIIE AS-NQNGIK-

#DmGMC_Q1_CG9519_{GMC-Q} -RGDYNG-DK QIRVSYLQAN IYN-ETRWSS NRAYLYP-IK GKRRNLHVKK NALVTKILID PQT--KSAFG IIVK------ -----MDGKM QKILARKEVI

#DmGMC_Q2_CG9521_{GMC-Q} -YRDYNG-RI QNGVAFLHTT TRN-STRWSS NRAYLYP-LK GKRSNLHVRK NALVTKVLID PQT--KTAYG IMVQ------ -----TEGRM QKILARKEVI

#AgGMC_Q4_{GMC-Q} -YVDYNG-PT QIGVSYIQST TKD-GKRDST NVAYLYD-MR -NRSNLHVKK NSQVTRILFD RSA--NQANG VRFF------ -----HAGRF HTVRARREVI

#AgGMC_Q3_{GMC-Q} -YVDYNG-PS QIGTSFIQST TKN-GQRVSS NNAYLYP-IR -NRTNLHIIR NAHVTKILLN RDT--KRATG VQFY------ -----ANHRY QKVRARREVI

#AmGMC_Q7_{GMC-Q} -ILDYNG-ER QVGVSLLQST TDM-GLRTSS NKAYLVG-KR --RKNLHVTK LSTVRRILFD EGR--GRAVG VEFA------ -----KRGRL FTVYVDKEVI

#TcGMC_Q6_{GMC-Q} -LVDYNG-PI QVGVSRFQVT LRD-GIRESS SRAYLHP-IK -NRPNFHMRK YSTVTKILID PTT--KKVQG VEVD------ -----TKGTI YKIGASKEVL

#TcGMC_Q5_{GMC-Q} -SVDYNG-PI QVGVSRLQVS MRD-GVRESA SRAYLHP-IR -NRPNLHVKK LAMVSKVLID PKT--KQTIG VEFF------ -----RDGTR YQIRASKEVI

#DmGMC_Q1_CG9519_{GMC-Q} LSAGAINTPQ LLMLSGVGPA KHLREMGIKP LADLA-VGYN LQDHIAP-AI SFLCN-V--- ----SSLQTS EMFRSEAMSD FLKG----RG VLRIPGGVEA

#DmGMC_Q2_CG9521_{GMC-Q} VSAGAINTPQ LLMLSGVGPA KHLREVGIKP LADLA-VGYN LQDHTAP-AV TFTTN-A--- ----TSLKFE DFSDPTLINR FNRM----EG PYGSPGGCEA

#AgGMC_Q4_{GMC-Q} VSSGAIGSPH LLMLSGIGPA DHLRANGIKP IADLP-VGHN FQDHTAAGGL TFLVNNT--- ----QTLTYK NVFRLDNFMK YQYDK---RG PFTSTGGCEA

#AgGMC_Q3_{GMC-Q} VSAGAIGSPH LLMLSGIGPA KHLRLKGIQP LANLA-VGFN FQDHVAGGAL TFLINHT--- ----ETLTSK RMFTLESFME YEHQH---TG MMASTGACEA

#AmGMC_Q7_{GMC-Q} VSAGAISSPK LLMLSGIGPA EHLREMGIEV VRDAR-VGDN LMDHIAYGSL LYDIDQR--- ----VDVIAN RLFQR-VLNN YFMDK---VG QLTSLGGTEA

#TcGMC_Q6_{GMC-Q} VAGGAVNSPQ LLMLSGIGPK KHLTQMGIPV LSNLK-VGYN LLDHVALGGL TFRIDEP--- ----YSLKTE RVLSRESLFQ FWNYH---QG PITAPGGCEV

#TcGMC_Q5_{GMC-Q} VSAGAINSPQ LLMLSGIGPR KHLTQKGIPV LSNLK-VGYN LMDHIALGGL TFIINKP--- ----YSLNTE KMITTENMRQ YLNYH---KG PLSVPGGCEV

#DmGMC_Q1_CG9519_{GMC-Q} ISFYALDDA- ---------- ---RNPDAWA DMELFVV-GG GLQTN--LAL RLALGIQSNI YETMFGELER QSANGFLIFP MILRAKSRGR IKLKSRN-PE

#DmGMC_Q2_CG9521_{GMC-Q} IAFWDLDHE- ---------- ---RDEDGWP DIELFLV-GG SMSSN--PAI SRAFGLKKSI YDTLFAEIED KSLNAFMIFP MILRPKSRGR IMLKSSD-PF

#AgGMC_Q4_{GMC-Q} IAFYDSERP- ---------- ---GDPDGWP DYELLHI-GG TIGAD--PTY EVNFNYKHKT FQTLFGEIQR RNYDGFTVFP LIMRPRSKGR ISLNGSS-PF

#AgGMC_Q3_{GMC-Q} ISFHDTTQPP ---------- -NRANEAGWP DLELLLI-GG THAAD--RIY ESNFNYKPET FNALFGDIER RGLEGYTVFP MILRPRSKGR IRLASAD-PF

#AmGMC_Q7_{GMC-Q} IAFIDVDDP- ---------- ----REREVP NVELLFL-GT SIYSV--NTL GDNFGLNEEI STKFTSYRNR ---RALSVFP ILLQPKSRGR IRLRSRD-AD

#TcGMC_Q6_{GMC-Q} VVFHDLKDP- ---------- ---TNPDGYP DIELVFL-GA SLSLD--PLL QKNLAISDYV YKTVYTPIER --FDSFMVFP MILRPQSRGR IALRDNN-YK

#TcGMC_Q5_{GMC-Q} LVFHDLKNP- ---------- ---TDPDGYP DIELLFQ-GG SIVSD--PLL RKDFGITNEL YDAVYKPIED --LDTFMVFP MLMRPKSKGR IMLKNNN-YR

#DmGMC_Q1_CG9519_{GMC-Q} E-HPRIYANY FA--NPYDMN ITVRGIEQAV SLLD-MPAFK AIGAHLLEKR IPNCAKYKWK S--------- SAYWACYARH FTFTIYHYSG TAKMGPRS--

#DmGMC_Q2_CG9521_{GMC-Q} K-YPLIHANY FA--HPYDVD ISVRGLLKAI SLME-QRGME KINARLWEKK IPTCKQHPYK S--------- WAYWACYVRH FTFTIYHYSG TAKMGPKS--

#AgGMC_Q4_{GMC-Q} Q-YPIIEPNY FD--DPYDLD ISVRAIRKAI ELSR-TGAMQ RYNARLLDIP MPGCEHYRFD S--------- DDYWKCFSRH ATFTIYHHVG TCKMGPRK--

#AgGMC_Q3_{GMC-Q} E-HPIIQPNY LG--DPYDLE VSVRGIRKAI ELTK-TNTLK SFDARLLDIP IPGCEQHRFD T--------- DDYWKCFTRH VTYTIYHHVG TCKMGPAS--

#AmGMC_Q7_{GMC-Q} D-KPRIFPNY MS--EPEDVK GLIKGIKAAN KFLLGTKAFE RLNTRLNNQT VPECEKFPFD S--------- DDYWECNLRL IPITIYHYSG TCKMGPES--

#TcGMC_Q6_{GMC-Q} S-KPRIFPNY FH--VKEDME TIIGGVRLTL NITA-QQPMR KIGTRLHDIP IPQCAHLEFA S--------- DGYFECMARH LTFTIYHHCG TCKMGPRS--

#TcGMC_Q5_{GMC-Q} A-KPYIYPNY FA--YDEDMD TIMGGVHLIL NITQ-QPALQ ALGARLHDIP IPQCAKYGFA S--------- DDYFKCMARH FTFTIYHQSG TCKMGPPS--

#DmGMC_Q1_CG9519_{GMC-Q} ---DPSAVVD ARLR-VHGID KLRVVDASIM PYLISGHPNG PVYLIAEKAA DMIKEDHNFV ---------- ---------- ---------- ----------

#DmGMC_Q2_CG9521_{GMC-Q} ---DRAAVVD HRLR-VHGIK NLRVADASIM PEIMSGHPNG PVFMIAEKAA DMIKQDHGFI Q--------- ---------- ---------- ----------

#AgGMC_Q4_{GMC-Q} ---DPTAVVD ARLR-VHGVK GLRVIDASIM PDVPAGHTNA PTIMIGEKGA DMIKQDWNEL T--------- ---------- ---------- ----------

#AgGMC_Q3_{GMC-Q} ---DRLAVVD PRLR-VHGVK GLRVIDASVM PDIPAAHTNG PTIMIAEKGA DMIKEDWNL- ---------- ---------- ---------- ----------

#AmGMC_Q7_{GMC-Q} ---DETAVVD PTLK-VIGVK GLRVVDASIM PMIPSGHTNI PTYMIAEKAS DMIKDEWGYP IS-------- ---------- ---------- ----------

#TcGMC_Q6_{GMC-Q} ---DKSAVVD PRLR-VYGVE GLRVIDASVM PEVPAAHTNA PIFMIAEKGA DMIKEEWVGN IGAD------ ---------- ---------- ----------

#TcGMC_Q5_{GMC-Q} ---DKKAVVD PRLR-VYGIK GLRVIDASIM PEVPAAHTNS PTFMIAEKGA DLIKEDWANR S--------- ---------- ---------- ----------

#DmGMC_Q1_CG9519_{GMC-Q} ---------- ---------- ---------- ---------- ---------- ---------- ---------- ---------- ---------- ----------

#DmGMC_Q2_CG9521_{GMC-Q} ---------- ---------- ---------- ---------- ---------- ---------- ---------- ---------- ---------- ----------

#AgGMC_Q4_{GMC-Q} ---------- ---------- ---------- ---------- ---------- ---------- ---------- ---------- ---------- ----------

#AgGMC_Q3_{GMC-Q} ---------- ---------- ---------- ---------- ---------- ---------- ---------- ---------- ---------- ----------

#AmGMC_Q7_{GMC-Q} ---------- ---------- ---------- ---------- ---------- ---------- ---------- ---------- ---------- ----------

#TcGMC_Q6_{GMC-Q} ---------- ---------- ---------- ---------- ---------- ---------- ---------- ---------- ---------- ----------

#TcGMC_Q5_{GMC-Q} ---------- ---------- ---------- ---------- ---------- ---------- ---------- ---------- ---------- ----------

#DmGMC_Q1_CG9519_{GMC-Q} ---------- ---------- ---------- ---------- ---------- ---------- ---------- --

#DmGMC_Q2_CG9521_{GMC-Q} ---------- ---------- ---------- ---------- ---------- ---------- ---------- --

#AgGMC_Q4_{GMC-Q} ---------- ---------- ---------- ---------- ---------- ---------- ---------- --

#AgGMC_Q3_{GMC-Q} ---------- ---------- ---------- ---------- ---------- ---------- ---------- --

#AmGMC_Q7_{GMC-Q} ---------- ---------- ---------- ---------- ---------- ---------- ---------- --

#TcGMC_Q6_{GMC-Q} ---------- ---------- ---------- ---------- ---------- ---------- ---------- --

#TcGMC_Q5_{GMC-Q} ---------- ---------- ---------- ---------- ---------- ---------- ---------- --

**Amino acid sequence alignment within the GMC ζ subfamily**

#DmGMC_Z1_CG9518_{GMC-Z} ---------- ---------- ---------- --------MV LNLLFITTVI KSTFGVVTTG LWLIPLMLAA ITYYRYD-AV DPESRPLDQL N------LYP

#AgGMC_Z1_{GMC-Z} ---------- ---------- ---------- --------MV FNVLIASSVI KTAT-VVGSS LWLIPFLLGA ISYYRYD-RV DPESRVINQE A------LLP

#AmGMC_Z1_{GMC-Z} ---------- ---------- ---------- --------MV LSAIVVASAL KGALSLIGTS LWLIPLLIAG LSYYRYD-QL DPESRPIDRY P------LYP

#TcGMC_Z1_{GMC-Z} ---------- ---------- ---------- --------MV VETLFISTAL K-TIGIVGST LWIIPLIFAG ISYYHYD-KL DPESPIINRR T------LYK

#DmGMC_Z1_CG9518_{GMC-Z} EYDFIVVGSG SAGAVVANRL SEVRKWKVLL IEAGPDE--- -----NEISD VPSLAAYLQL SK-LDWAYKT EPST------ ---KACLGMQ NNRCNWPRGR

#AgGMC_Z1_{GMC-Z} EYDFIVVGGG SAGAVVANRL TEIHRWKVLL LEAGPDE--- -----NEISD VPSLAAYLQL SK-LDWAYKT EPTN------ ---KACLGMV NNRCNWPRGK

#AmGMC_Z1_{GMC-Z} EYDFIVVGGG SAGAVVANRL SEIPKWNVLL LEAGPDE--- -----NEVTD VPSLAAYLQL TK-IDWKYKT EPTG------ ---RACLAMK DGRCNWPRGK

#TcGMC_Z1_{GMC-Z} EYDFVVVGGG SAGAVVASRL SEIPSWNVLL LEAGPDE--- -----NEISD VPSLAAYLQL SK-LDWTYKT EPTG------ ---RACLGMN NGRCNWPRGK

#DmGMC_Z1_CG9518_{GMC-Z} VLGGSSVLNY MLYVRGNRHD YDHWAS-LGN PGWDYDNVLR YFKKSEDNRN PYLANN---- ----KYHGRG GLLTVQESPW HSPLVAAFVE AG-TQLGYD-

#AgGMC_Z1_{GMC-Z} VLGGSSVLNY MIYVRGNRND FNHWES-LGN PGWAYDDVLQ FFVKSEDNRN PYLARN---- ----PYHGQG GLLTVQEAPW HTPLVAAFVE AG-TEIGYE-

#AmGMC_Z1_{GMC-Z} VLGGSSVLNY MLYVRGNRHD YDHWES-MGN PGWGYDQALY YFKKSEDNRN PYLQKS---- ----PYHSTG GYLTVQESPW KTPLVVAFVQ AG-TEIGYE-

#TcGMC_Z1_{GMC-Z} VLGGSSVLNY MLYVRGNRHD YDQWEA-MGN HGWNYENVLH YFKKSEDNRN PYLART---- ----KYHNQG GLLTVQESPW RTPLVLAFVQ AG-TELGYP-

#DmGMC_Z1_CG9518_{GMC-Z} -NRDING-AK QAGFMIAQGT IRR-GSRCST AKAFLRP-IR M-RKNFHLSM NSHVTRVIIE PGT--MRAQA VEFV------ -----KHGKV YRIAARREVI

#AgGMC_Z1_{GMC-Z} -NRDING-ER QTGFMIAQGT IRR-GSRCST AKAFLRP-IR L-RKNLHIAM NSHVSKLVID PET--KHAVG VEFF------ -----RGGKR HYVRARKEII

#AmGMC_Z1_{GMC-Z} -NRDING-ER QTGFMIAQGT IRR-GSRCST AKAFLRP-IR L-RRNIHTAM NCHVTRILID PIA--MRATG VEFV------ -----RDGRR QIVRARKEVI

#TcGMC_Z1_{GMC-Z} -NRDING-AE QAGFMVAQGT IRR-GSRCST AKAFLRP-IR L-RKNIHIAL NSHVTRVLIN PST--MRAFG VEFV------ -----RNGHK QIVLARKEVI

#DmGMC_Z1_CG9518_{GMC-Z} ISAGAINTPQ LMMLSGLGPR KHLEKHGIRV LQDLP-VGEN MQDHVGMGGL TFLVDKP--- ----VAIVQD RFNPTAVTFQ YVLRE---RG PMTTLGGVEG

#AgGMC_Z1_{GMC-Z} MSAGSINTPQ ILMLSGIGPR AHLEDVGITT IQDLP-VGEN LQDHVGMGGL TFLVDKP--- ----VAILQN RLEAGSVTMN YVINE---RG PMTILGGLEG

#AmGMC_Z1_{GMC-Z} LSAGAINSAQ ILMLSGIGPK EHLRHIGIPV IKDLR-VGDN LQDHVGMGGL TFLIDKP--- ----VAIVQD RFQAAAITMH YVANG---RG PMTTLGGVEG

#TcGMC_Z1_{GMC-Z} MSAGAINTPQ ILMLSGIGPQ PQLSKFGIPV LRDLP-VGEN LQDHVGMGGF TFLVDKP--- ----VSIVQD RFQAFPMTMQ YVMNA---KG PMTTLGGVEG

#DmGMC_Z1_CG9518_{GMC-Z} LAFVHTP--- ---------- -YSNRSLDWP DIQFHMA-PA SINSDNGARV KKVLGLKESV YQEVYHPIAN --KDSWTIMP LLLRPRSRGS VKLRSAN-PF

#AgGMC_Z1_{GMC-Z} IAFVNTP--- ---------- -FANVTDDWP DIQFHMA-PA SLNSDGGARV KKVLGLREDL YKEVFHPIED --TYSWTIMP LLLRPRSRGW VRLKSNN-PF

#AmGMC_Z1_{GMC-Z} YAFVNTK--- ---------- -YANRSIDYP DIQLHMA-PA SISSDAGAQV RKVLGITDEV YDTVFKPISN --KDAWTIMP LLLRPKSRGT VRLRSSN-PF

#TcGMC_Z1_{GMC-Z} LAFVNTK--- ---------- -YGNR--SWP DVQFHMA-PA SINSDAGVRV RKVLGLTDHL YNTVYRPIAN --KDVFTLMP LLLRPKSRGW IRLQSKN-PF

#DmGMC_Z1_CG9518_{GMC-Z} H-YPLINANY FD--DPLDAK TLVEGAKIAL RVA-EAQVFK QFGSRLWRKP LPNCKQHKFL S--------- DAYLECHVRT ISMTIYHPCG TAKMGPAW--

#AgGMC_Z1_{GMC-Z} H-YPLMNPNY FE--DPFDAA TLVEGAKIAL RVG-DAKVFK QFGNRLYRKP LPNCKQHKFL S--------- DEYLDCQVRT ISMTIYHPVG TAKMGPHW--

#AmGMC_Z1_{GMC-Z} H-SPLINANY FS--DPIDIA TLVEGAKIAM RIN-EAKVFK QFGSRVHRIK VPGCKHLNFA S--------- DAYWECHIRH ISMTIYHPVG TAKMGPSS--

#TcGMC_Z1_{GMC-Z} V-PPVINANY FD--DPIDIK VLVEGAKMAI KIG-EAQAFK QFGARVHRIK FPNCRDFEFG S--------- DEYLECHIRT ISMTIYHPVG TCKMGPSW--

#DmGMC_Z1_CG9518_{GMC-Z} ---DPEAVVD PRLR-VYGVR GLRVIDASIM PTISSGNTNA PVIMIAEKGA DLIKEDWLTN PEYKVKRQAN RLRDPDPASS NIQGIITLPN NITQGDSSNI

#AgGMC_Z1_{GMC-Z} ---DPGAVVD PRLR-VYGIS GLRVIDASIM PTIVSGNTNA AVIMIGEKGA HMIKEDWLGH DR-------- ---------- ---------- ----------

#AmGMC_Z1_{GMC-Z} ---DPTAVVD PKLR-VYGVR GLRVIDASIM PTISSGNTNA PVIMIGEKGA DLVKNDWLAI ESARN----- ---------- ---------- ----------

#TcGMC_Z1_{GMC-Z} ---DKEAVVD PRLK-VYGVE GLRVIDASIM PTIPSGNTNA PAIMVGEKGA DLVKEDWLQR ---------- ---------- ---------- ----------

#DmGMC_Z1_CG9518_{GMC-Z} SDRSNMESNF SNSSHINNIN FNSNSNSSNI ESSSNFTLNY ANS------- ---------- ---------- ---------- ---------- ----------

#AgGMC_Z1_{GMC-Z} ---------- ---------- ---------- ---------- ---------- ---------- ---------- ---------- ---------- ----------

#AmGMC_Z1_{GMC-Z} ---------- ---------- ---------- ---------- ---------- ---------- ---------- ---------- ---------- ----------

#TcGMC_Z1_{GMC-Z} ---------- ---------- ---------- ---------- ---------- ---------- ---------- ---------- ---------- ----------

#DmGMC_Z1_CG9518_{GMC-Z} ---------- ---------- ---------- ---------- ---------- ---------- ---------- --

#AgGMC_Z1_{GMC-Z} ---------- ---------- ---------- ---------- ---------- ---------- ---------- --

#AmGMC_Z1_{GMC-Z} ---------- ---------- ---------- ---------- ---------- ---------- ---------- --

#TcGMC_Z1_{GMC-Z} ---------- ---------- ---------- ---------- ---------- ---------- ---------- --

**Amino acid sequence alignment within the GMC β subfamily**

#DmEO_B1_CG9504_{GMC-B} ------MGDQ RVHKPRTAKT RTLRVNRILL PMLLLFSGET TSVVTQLFSV DSSGLGISLM QSVAIALNAS SLALANNTAW PLQHEPPEDR LE------IE

#DmGMC_B2_CG9509_{GMC-B} ---------- -----MSLDG GQNLNLDAAA IGPDPCGSVP ATPGIGLWSG --------MV TILVQTLLSS QCLVSPASQW P------VDY VGDL----SQ

#DmGMC_B3_CG9512_{GMC-B} ---------- -----MEFLS AQ-------- -----CAARS AGPANTLMS- ---------- -LLLSTLITK YCDLSGQNQW PEDKGDWLEQ AGGF----KH

#AgGMC_B4_iso4_{GMC-B} ---------- -----MEALM GG-------- ----QCAAQS VGPANQLFG- ---------- -LLVQTILAA QCAISPPDMW PKDYGPTALQ RG------LD

#AgGMC_B4_iso3_{GMC-B} ---------- -----MEALM GG-------- ----QCAAQS VGPANQLFG- ---------- -LLVQTILAA QCAISPPDMW PKDYGPTALQ RG------LD

#AgGMC_B4_iso2_{GMC-B} ---------- -----MEALM GG-------- ----QCAAQS VGPANQLFG- ---------- -LLVQTILAA QCAISPPDMW PKDYGPTALQ RG------LD

#AgGMC_B4_iso1_{GMC-B} ---------- -----MEALM GG-------- ----QCAAQS VGPANQLFG- ---------- -LLVQTILAA QCAISPPDMW PKDYGPTALQ RG------LD

#AmGMC_B6_{GMC-B} ---------- -----MPS-- ---------- -----GMSST S--------- --------IF TLLLQAIMTS YYDLSDPRQY PADRTEEILN SN-------K

#AmGMC_B7_{GMC-B} ---------- -----MES-- ---------- -----CMSRT CSSVIAQQS- ----SPASIF TFLIQTLIAS RCKLNNPDEY PRDRVNDVLR SN-------K

#AmGMC_B8_{GMC-B} ---------- -----MES-- ---------- -----CMTTS CSPMLH--S- ----SPVCIF TLFLQTLEAS YYGLSNPNTY PRNRKQEILD SK-------I

#AmGMC_B9_{GMC-B} ---------- -----MES-- ---------- -----CARGT CSSALQ--S- ----SPASIF TMLIQTLIAS RCQLSNTNKY PTSNEEKILN SK-------M

#AmGMC_B10_{GMC-B} ---------- -----MES-- ---------- -----CMAAT CASGQS---- ----TPASTF TMLIQTIMAS YC-ASNVGKY PTDRAEEIFA STR------K

#TcGMC_B5_{GMC-B} ---------- -----MDHAI DP-------- -----CPSST SGVSAHLFL- ---------- -TLINSLLAS KCRISSPSNY PQNRASTLSD ND--------

#DmEO_B1_CG9504_{GMC-B} SYDYIVVGAG SAGSIVASRL SELCQVKVLL LEEGQLP--- -----PLESE IFGLTGALHH DE-RYMFLEE AVP------N PKCCQAMASM HG-CVWWHGR

#DmGMC_B2_CG9509_{GMC-B} PYDFVVIGAG SAGSVVASRL SENPDWRVLV LEAGGDP--- -----PVESE LPALFFGLQH TN-FTWNYFT E--------- PSDEACQAMK DGRCYWPRGK

#DmGMC_B3_CG9512_{GMC-B} DYDFIVIGSG TSGAVVAGRL AEVKNWKVLL LEAGGDP--- -----PIETE FVAWHMATQF SE-WDWQYHS K--------- PNGRACMAMK GESCHWPRGK

#AgGMC_B4_iso4_{GMC-B} EYDFVIVGAG SAGSVVANRL SENPDWKVLL LEAGGDP--- -----PIESE VPYLAFALLN GS-HVWNYYA E--------- RSDTASKGYK RG-SYWPRGK

#AgGMC_B4_iso3_{GMC-B} EYDFVIVGAG SAGSVVANRL SENPDWKVLL LEAGGDP--- -----PIESE IPETFFTIQK TD-ADWENYV E--------- PTPHASKGSK DG-AFWPRGR

#AgGMC_B4_iso2_{GMC-B} EYDFVIVGAG SAGSVVANRL SENPDWKVLL LEAGGDP--- -----PIESE IASMAMALQH SD-VDWAYNV Q--------- RSDSSSLGTR NG-TFWPRGR

#AgGMC_B4_iso1_{GMC-B} EYDFVIVGAG SAGSVVANRL SENPDWKVLL LEAGGDP--- -----PIESE IPFMQIHLAK SS-VDWVYYA DSRDKLNPHN RTACRASTSP AG-CFWPRGK

#AmGMC_B6_{GMC-B} EFDFVIVGGG TAGSVLAHRL TEVMDWDVLL VERGEDP--- -----LPETE VPALVFNNFG SS-QDYRYAT E--------- YQEGACMSMK GKRCKWSKGK

#AmGMC_B7_{GMC-B} EFDFVIIGGG TAGSILARRL TEVKNWNVLL IERGGYP--- -----LPETA VPALFTSNLG FP-QDYAYKI E--------- YQKEACLSQV DKRCRWSKGK

#AmGMC_B8_{GMC-B} EFDFVIVGGG SAGSVLARRL TEVEDWKVLL VERGGYP--- -----LPETE IPGFFANNLG LK-QDYAYKV E--------- NQEEACLSQV DKRCRWSKGK

#AmGMC_B9_{GMC-B} EFDFVIVGGG SAGSVLARRL TEVEDWNVLL IERGVDP--- -----LPETI PPGLYNNNLG GP-QDYYYTL E--------- PQESSCLSNK DKRCIWSRGK

#AmGMC_B10_{GMC-B} EFDFIIVGSG SAGSILASRL TEVNDWDVLL IERGEDP--- -----LPETS SPALFFDNID GP-QNYHYLT E--------- YQNTSCLGTV HQRCKWASGK

#TcGMC_B5_{GMC-B} EFDFIIVGAG SSGSVVANQL SLNRNWKVLV LESGNLP--- -----PPDSE IPSLLFSLQG TE-SDWQYAT E--------- PNQKSCQGFI EKKCRWPRGK

#DmEO_B1_CG9504_{GMC-B} MMGGGGAING NIFIPGSREN FRRWN----S TGWDWTQVHK TYSRLQQRLN PSYLQP---- ---------- --------NK LNLKLANLIY SGSAELGVPR

#DmGMC_B2_CG9509_{GMC-B} MLGGSGGVNA MLYVRGNRRD FDGWAA-MGS TGWSYDQVMP FFEKSVTPQG NATHPK---- ---------- GYVTLKPFER KDNDIHQMII DGGRELGQPY

#DmGMC_B3_CG9512_{GMC-B} MLGGTNGMNA MIYARGTRKD FDDWEE-RGN PGWGYDEVLK HFRKAEDLRS TRPDYKP--- ----GDHGVG GPMGLNNYVS -DNEFRTTIR AGMQEMGYGS

#AgGMC_B4_iso4_{GMC-B} MLGGSSSNNI MLYVRGNSRD YDRWEE-QGN PGWGWKDVLE YFKKSEDNGA QHLLQER--- ---ADYHAQG GLLKVNSFMS -NDMTKLVIT EAAQELGIPE

#AgGMC_B4_iso3_{GMC-B} TLGGCGAINA MLYVRGNSRD YDGWAE-LGN PNWEWSDVLP YFKKSEDNHD SELLRRDG-- ---GKYHAAG GYLKVGNFPV -NHPLAEVML QAFKDAGFES

#AgGMC_B4_iso2_{GMC-B} TLGGSGAINA MMYVRGNRRD YDRWQS-LGN PEWGWEDVLP YFRKSENMNN PRLVRGEG-- ---AKYHRTG GYLNVEQRID -NTTLNGILR RGALELGYEW

#AgGMC_B4_iso1_{GMC-B} MLGGSGAMNA MVYIRGNARD YDAWEF-EGN SGWGWRDVLP YFRKSENNHD AAVVG--D-- ---GTYHGTG GYLSVSSASG -HSGHMEHLI AAVQESGYDY

#AmGMC_B6_{GMC-B} ALGGSSVINA MLHVFGNRMD YDDWAS-EGN EGWGYEQVLP YFRKSLS-CS PDHVARFG-- ---SDYCGTS GPMRIRNYNY TATDIQDVML DAARELGYEI

#AmGMC_B7_{GMC-B} ALGGSSVINA MLHIFGNKRD YDTWEN-IGN PGWNYEQVLP YFRKSLS-CA PEFIAKYG-- ---TDYCGTD GPMRIRHYNY TATDAEDIIL EAAHEAGYDV

#AmGMC_B8_{GMC-B} ALGGSSVINA MFYIFGNKRD FDTWEN-IGN PGWNYEQVLP YFRKSLS-CS PEFIAKYG-- ---TDYCGTD GPLKIRNYNY TETDAINILS EAVQQAGYDI

#AmGMC_B9_{GMC-B} ALGGSSVING MIHIFGNRRD FDGWAS-QGN PGWNFEEVLP YFRKSIS-CS PEYIAENG-- ---DKYCGTD GPLRVRYYNY TVTDFEDVVL EAAREAGHPI

#AmGMC_B10_{GMC-B} ALGGSSVTNG MLYVIGNEKD YNDWEE-SGN DGWGFASVLP YFAKSTN-CS ASYVSRYG-- ---TKYCGDN GPVRIGHFEA ASPGVQKILM DGVREAGHDV

#TcGMC_B5_{GMC-B} CLGGSSAINA NLYIRGNRRD YDTWAE-LGN EGWDYDSVME YYKKLEDVDG FDGYGR---- ---------G GFVPLNVYQS -NEPVGEALK DSARVLGYPT

#DmEO_B1_CG9504_{GMC-B} MKQPLIAGAT FGYTHHVPVT VNQ-RRRASS ARLYLANDQV NRRGNLKVIR GAQVQRVLLN AAGSRATGVI YTLNGVE--- ---------- HTAKTLGEVI

#DmGMC_B2_CG9509_{GMC-B} VERFQEGSDT G--YSHVPGT VRQ-GQRMST GKGYLGAVSK SRP-NLHVVK NALVTKLDLD G-ETVKE-VK FERAG-V--- ---------T HRVKVTKDVV

#DmGMC_B3_CG9512_{GMC-B} APDFTEGSFV G--QMDILGT QDG-GRRITT ARSHL---KK NTP-NLHILR HAHVKKINLD RNNRAES-VT FVHRGKK--- ---------E YTVKASKEVI

#AgGMC_B4_iso4_{GMC-B} IMDINSDEYI G--YNVAQGT VHK-GRRWST AKAFLNT-AA DRP-NLHIIK NAHVTKINFE GTAATG--VT FDVPSQT--- ---------G VSASIRKEVI

#AgGMC_B4_iso3_{GMC-B} TADINGARQV G--FGRAQGT IVN-GTRCSP AKAFLVP-VK DRP-NLHVIK HAVVVTVERD PSTERFKYVN FMIDNKV--- ---------L KVAHARKEVI

#AgGMC_B4_iso2_{GMC-B} IDDFNRDRHN G--YGNTQYT IIG-GTRCSP AKAFLTP-VR KRQ-NLHVIK YAFVNRVLID ERNVATG-VR FVVDGSQ--- --------RV QQVAVRREVI

#AgGMC_B4_iso1_{GMC-B} LEDFNGENHI G--FGRVQLN TIE-GARCSP AKAFLAP-IK DRR-NLHVIK RALATKLEVD AHQRVSS-VR FVIDEHNDSS N----DQTRV LEVKVRKETI

#AmGMC_B6_{GMC-B} LEPLNGDRFV G--FGRAMGT LDD-GRRLNA AKAFLSP-VK YRR-NLYVMK SSRVDRVLFG EDGRASG-VR ITLKNN---- --------EQ IDVRAAKEVI

#AmGMC_B7_{GMC-B} LEPLNGDRFI G--FGRAMGT LDN-GQRENC AKAFLSP-VK DRK-NLYVMT SSRVDKILF- ERKRAVG-VR ITLDNN---- --------QS VQVRATKEVI

#AmGMC_B8_{GMC-B} LEPVNCDRFI G--FGRAMGN IDN-GQRQSC AKAFLSP-VK NRE-NLYVMT SSRVDKILF- EGERAVG-VR ITLDND---- --------EP IEVKATKEVI

#AmGMC_B9_{GMC-B} LKAVNGDRYL G--FGRVLGT LDE-GRRQTC SKAFLTP-VR DRK-NLYVIT STRANKILF- EGKRAVG-VQ ITLSNN---- --------ET AEVRATKEVI

#AmGMC_B10_{GMC-B} LEVVNGDRFV G--FGRAMGT VHD-GRRENA AMAFLSP-AK GRK-NLSVMK SSAVEKVLF- EEGRAIG-VR VRSEQKG--- --------FV AEVRARKEVI

#TcGMC_B5_{GMC-B} IP---QEGNF G--YFEALQT VDK-GIRANA GKIFLGR-AK DRE-NLVVAM GATVEKILLK EKKTEG--VL VNIGGRQ--- ---------- IALKARKEVI

#DmEO_B1_CG9504_{GMC-B} LSAGTLNSAK LLLLSGIGPR EELQRWNITT HQDLP-VGRN LQDHGMMPLF LLFGSNCA-- -VNSTRDPTE NPYAPVSITQ YLLDN--QKG PLASG--FYM

#DmGMC_B2_CG9509_{GMC-B} ISAGAIDSPA LLLRSGIGPS KHLKELGIPV KLDLPGVGRN LQDHVLVPVF LRLDE----- --GQGEPM-T DQAALDSIYQ YLIY---RAG PLAAHSTASL

#DmGMC_B3_CG9512_{GMC-B} VSAGAIGSPQ ILLLSGIGPA DHLKSLGIPV KLDLP-VGEN LKDHASLPMI FQIDK----- --STARKP-T EEELVDAMYN LLMG---RYS KLLHHEATAL

#AgGMC_B4_iso4_{GMC-B} ISAGAINTPQ VLQLSGLGAK EQLDRLDIPL VKEIPSVGEN LQDHLIVPLF LSLHG----- --SRPIER-S MDELLDSIYS YFRY---GLG TFGTVGITDL

#AgGMC_B4_iso3_{GMC-B} LAAGAINTPH ILQLSGIGPK ALLEKVNIPL VADLP-VGEN LQDHLFVPLL FKMHK----- --STAENYNI QQELAKNLFQ YIMT---RSG PMAGHGVTSV

#AgGMC_B4_iso2_{GMC-B} LAAGAINTPQ LLMLSGVGRT DELKQFGIPP KVDLN-VGGN LQDHVAVPLF FKFYA----- --LQEQDINE QLARINELYT YVVQN--RSQ AVVRTGPLNT

#AgGMC_B4_iso1_{GMC-B} VSAGAVNTPQ LLMLSGIGQE EDLREHGIRI VSDLP-VGRN LQDHVMVPLF YCINR----- --SSATDFDL NRNVIGHMYD YLMH---RNG PLSEIGINAF

#AmGMC_B6_{GMC-B} LSAGSVASPQ ILMLSGIGPR RHLDEMGISL VHDLP-VGEN LQDHAIWLGT NLLFVN---- -ESITSPMPV DAIYDS-AYE YLIH----KT GQLRDLPIDL

#AmGMC_B7_{GMC-B} LSAGSIASPQ VLMLSGIGPK NHLKKMGIPT LVDLP-VGKN LQDHAIWLGI YLAYNN---- -ESVTSPPSE KSQLDD-IYD YLEF----NA GPLRVLPLDL

#AmGMC_B8_{GMC-B} LSAGSIASPQ ILMLSGIGPK EHLNKMGIPT LVDLP-VGMN LQDHVSWLSF YLRYTN---- -ESITPPFDE KNQLDDAVYE YLKQ----NT GPLRTLPVEF

#AmGMC_B9_{GMC-B} LSTGTMVSPQ LLMLSGIGPK EHLKKLGIPV LVDLP-VGKN LQDHVIWFGL YYSFVN---- -ESVTSAPSE KDQLDS-AYE YLEF----NT GPLSTLANDL

#AmGMC_B10_{GMC-B} LSAGSIATPQ LLMLSGIGPR EHLEKMGIPV VADRP-VGKN LQDHLAWTGM YITYAN---- -ESSISSPSL NRSLSS-IYE YMME----NR GPLRAYRTDF

#TcGMC_B5_{GMC-B} LSAGAINSPQ LLMLSGIGPK KHLQDVGIDP VMDLQ-VGEN LQDHIFYLGL LVAVD----- --DKVSQV-- QTNVIDEIYK YFMY---NEG AVGQIGITNL

#DmEO_B1_CG9504_{GMC-B} MGYINS---- ---------- ---SSPSSSR GEPDLHVVAH TLLPKGSTGS FGYLGFRPEL IQAQQDILQK --GDLLQIMG SLLRPLSHGK VSLSSK--NS

#DmGMC_B2_CG9509_{GMC-B} VGFINTN--- ---------- --ASSDGAYP DTENHHMFFQ RAHHASLELF TKGLSIQDQY TEVLQEYLKD --SHLLCVFV LLSHPAARGE LRLKST--DP

#DmGMC_B3_CG9512_{GMC-B} TGFINT---- ---------- --TSIEGPNP DIQTTN-FFS LMQSPELKGY VAATGFNDRV AKSILSANQE --TNTYITYL LHLKPFSAGS LTLQSA--NY

#AgGMC_B4_iso4_{GMC-B} LAFVNT---- ---------- --QSPAAKFP DIQYHHSLIL WK-TPDIARL TQCFGWEDYI SHQIIEQNQK --SEILMVMV TLLNPKSKGN VQLRSS--NP

#AgGMC_B4_iso3_{GMC-B} IGFINT---- ---------- --LDATSPFA DIEYHFFQFE KG-SGKSVLF CDKVGYTQEI SQSMLEAATE --ADVVMAIV VLLNPKSKGR VTLATEDFNE

#AgGMC_B4_iso2_{GMC-B} GAFLNT---- ---------- --KNTSDPFP NLQILNFAFP RG-GRFSEAQ TRHFEFTDII SASVQEVDRV --TPAMYVHI TALNPKSRGR VKLSSA--NP

#AgGMC_B4_iso1_{GMC-B} TGFVNT---- ---------- --VNHSDPFP NIQYHHMYSR KR-SNIAGRW LRMMELDEPF SSSVADANNE --ADVLGAFV ILLKPKSWGR IRLQSG--QI

#AmGMC_B6_{GMC-B} QGFVNV---- ---------- --TDPSSRYP DVQFLVAPIH RFESHILTSV MNSFDMMDEL VTDMSRVITN --ASMVIVYP ILLKPRSRGV VRLRST--DP

#AmGMC_B7_{GMC-B} NGFVDV---- ---------- --NDPHSKYP NVQFMFVPYQ RYTNN-LLSL LQGYNMNDDI IQEMQQAVKK --MSLISICP VLIRPLSRGF VELRNT--NP

#AmGMC_B8_{GMC-B} TGFVDV---- ---------- --NDPHSKYP NVQFIFMPVQ -FLSQ-LRDY LRAFNVDNDL IKKIENDVKE --MKIIFSSA TLLKPLSRGF LELRST--NP

#AmGMC_B9_{GMC-B} VAFINP---- ---------- --VDPKSIYP EVQLLFSQIQ RYDKNGLKTL LHSYNANDEI LQIMTDVIMK --RSLIIAYA SLMRPLSRGV IELRNA--DP

#AmGMC_B10_{GMC-B} LGTVNV---- ---------- --NDPNSSYP DVQFLFVPFE RHERVQLSVF LETIGLREEI GGKLVEEIER --TSVIVVLS ILLKPRSRGM VELRST--DP

#TcGMC_B5_{GMC-B} LGFVNS---- ---------- ---RNDSNYP NLQFHHILYI KGDNYLLPEI LRVTGLGPEV ASIELQANQK --SPMFKIAP TLLNPKSRGN ILLKSK--NP

#DmEO_B1_CG9504_{GMC-B} ADQAKIENHY GE--AVEDQQ TLLRYVRYIQ KLSK-TRPFR RCGLRLWKPP LHECDTLA-A DS-------- DDYWLCYIRY FYVGAWHSVG TCRMAPRKGV

#DmGMC_B2_CG9509_{GMC-B} KVPPILTSNY LT--ESEDVA TLMRGIRYIE SLEQ-TKAFQ DHLAEIARIP IKECDQIENY RS-------- EEYWRCYAKY FTVTCYHQSG TVKMGP----

#DmGMC_B3_CG9512_{GMC-B} LDAPIIDPGY MT--DERDVD TYIRALNIYK NLPN-TKAFS EREAALHKLD LEACNGLT-Y QS-------- DDYWRCYIRH MTTTVYHPVG TTRMGP----

#AgGMC_B4_iso4_{GMC-B} YDAPIINANY LD--DQRDVK TIIRGIRFFR KLLD-TENFG YHELKEFHLK IEECDRLE-Y ES-------- DSYWECYARY MSSTIYHPTG TAKMGP----

#AgGMC_B4_iso3_{GMC-B} FNPPRIQSGY LE--AKEDVE AVLRGIRYIN KIVD-TPTFR EHEGELHRMK LSECDELV-Y DS-------- DDYWECYARY TTLTLYHPVG TAKMGP----

#AgGMC_B4_iso2_{GMC-B} RVHPIIEANY FE--HTDDLN VLVQGIRLQQ RLLQ-TEAFR SAGAALHRID IPGCQELV-Y DT-------- DAYWECYVRQ LTVTTYHPVG TAKMGP----

#AgGMC_B4_iso1_{GMC-B} EQKPKIDAGY LT--HRQDIE TLIEGIRIHQ DIMT-TDAAK PMEPEPVRIE LPSCQDEL-Y DS-------- NAYWECYIRE LTLTLYHPVG TAKMGP----

#AmGMC_B6_{GMC-B} ADPVKIHANY FA--EKADLE TLLKSVDVIK ALVN-TETLK RHGMRLHHFD IPGCRHAK-P DT-------- EEYWECNVRH VTTSLFHACG TARMGP----

#AmGMC_B7_{GMC-B} ADPVKIYANY FA--EKEDFN NLLKSVNIVK AFLN-TDILK KYNMTLYYPN ISGCQHTE-P GT-------- DEYWECNLEH LSTTLFHPCG TAMMGP----

#AmGMC_B8_{GMC-B} ADPVKIYPNY FA--EKEDFN TLLKSVNVIK NLLN-TKVLK KYNMKLFYPD IPGCRHTK-P GT-------- DEYWECNLKY LSTTLFHPCG TAMMGP----

#AmGMC_B9_{GMC-B} AEQVKIYSNY YT--VPDDWK RLAKAVPTLK SLLN-TTILQ KYKANFHTYD VPQCRNLT-A DT-------- EEYYECNIRH TTGTNFHACC TNRMGP----

#AmGMC_B10_{GMC-B} TDPVKIYANY LV--EAEDTR TLVKSVDKMK EILD-TDALT GNGMRFNRLD VPGCRRFE-P DT-------- EQYWECSVRH VSVSYYHSCG TSRMGP----

#TcGMC_B5_{GMC-B} NDKPLIFANY LD--DPLDVE TLLEGIKFGL KQIE-SDPFA KFKPKLIDYN LKECQKFE-Y KS-------- DDYWRCAIRW LTTTLYHPVG TCKMGP----

#DmEO_B1_CG9504_{GMC-B} DSQENGGVVD ERLR-VHGVK GLRVVDASIM PELPAGNTNG PAMMIGEKGA QMILDDRE-- -ANNEVIQEC ---------- ---------- ----------

#DmGMC_B2_CG9509_{GMC-B} -DYDNEACVS QRLK-VHGLE NLRVADASIM PAVVSANTNA ATVMIGERAA HFIQEDYQGE AVGANGGLWV PHMHADEF-- ---------- ----------

#DmGMC_B3_CG9512_{GMC-B} -STDPTAVVD PQLR-VHGAK GLRVIDASIM PDIVGANTNA ACIMIAEKGA DMIKEEYLG- --GKHTEL-- ---------- ---------- ----------

#AgGMC_B4_iso4_{GMC-B} -NGDQASVVD SRLK-VRGVQ NLRVIDASIM PDIVSGNTNA PTIMIGEKGA DMIKEDYGVE KKEAATHTEL ---------- ---------- ----------

#AgGMC_B4_iso3_{GMC-B} -DSDKEAVVD ARLR-VKGVE GLRVVDGSIM PNIVSGNTNA PIMMIGEKAS DMIKEDWG-- --EGPNHTEL ---------- ---------- ----------

#AgGMC_B4_iso2_{GMC-B} -ATDPDAVVD SKLR-VRGVH GLRVIDASIM PLIVSGNTNA PTIMIAEMGS DFIKQEHG-- ELLDETGNEF ---------- ---------- ----------

#AgGMC_B4_iso1_{GMC-B} -SNDPDAVVD PRLR-VKGVA GLRVVDASIM PDIVSGNTNA AVIMIGEKAS DMIKQDHG-- WNEEEKQRN- ---------- ---------- ----------

#AmGMC_B6_{GMC-B} -ADDSRAVVD SRLK-VHGVD RLRVIDASIM PTIVSGNTNA PTMMIAEKGA DMIKEDWCKD LRVEEGDDTR QTCM------ ---------- ----------

#AmGMC_B7_{GMC-B} -ANDSRAVVD SRLK-VHGVQ NLRVIDASIM PEVTSGNTNA PTMMIAEKGA DIIKQDWGVK IQI------- ---------- ---------- ----------

#AmGMC_B8_{GMC-B} -ANDSRAVVD SRLK-VHGIE NLRVIDASIM PEVTSGNTNA PTIMIGEKGA DIIKEDWGIK I--------- ---------- ---------- ----------

#AmGMC_B9_{GMC-B} -ANDSRTVVD ARLR-VHGVT NLRVIDASIM PNITSANINA PTIMIAEKGA DLIKQDWGIQ V--------- ---------- ---------- ----------

#AmGMC_B10_{GMC-B} -GNDTRAVVD PRLR-VHGVD GLRVIDGSII PEIPAANPNA ATMMIAEKGA DMVKRDWGVK AR-------- ---------- ---------- ----------

#TcGMC_B5_{GMC-B} -RADPTSVVD PRLR-VHGIE GLRVIDASIM PLIISGNTNA PCLMIGLKGG AMILEDWG-- ----VKHDEL ---------- ---------- ----------

#DmEO_B1_CG9504_{GMC-B} ---------- ---------- ---------- ---------- ---------- ---------- ---------- ---------- ---------- ----------

#DmGMC_B2_CG9509_{GMC-B} ---------- ---------- ---------- ---------- ---------- ---------- ---------- ---------- ---------- ----------

#DmGMC_B3_CG9512_{GMC-B} ---------- ---------- ---------- ---------- ---------- ---------- ---------- ---------- ---------- ----------

#AgGMC_B4_iso4_{GMC-B} ---------- ---------- ---------- ---------- ---------- ---------- ---------- ---------- ---------- ----------

#AgGMC_B4_iso3_{GMC-B} ---------- ---------- ---------- ---------- ---------- ---------- ---------- ---------- ---------- ----------

#AgGMC_B4_iso2_{GMC-B} ---------- ---------- ---------- ---------- ---------- ---------- ---------- ---------- ---------- ----------

#AgGMC_B4_iso1_{GMC-B} ---------- ---------- ---------- ---------- ---------- ---------- ---------- ---------- ---------- ----------

#AmGMC_B6_{GMC-B} ---------- ---------- ---------- ---------- ---------- ---------- ---------- ---------- ---------- ----------

#AmGMC_B7_{GMC-B} ---------- ---------- ---------- ---------- ---------- ---------- ---------- ---------- ---------- ----------

#AmGMC_B8_{GMC-B} ---------- ---------- ---------- ---------- ---------- ---------- ---------- ---------- ---------- ----------

#AmGMC_B9_{GMC-B} ---------- ---------- ---------- ---------- ---------- ---------- ---------- ---------- ---------- ----------

#AmGMC_B10_{GMC-B} ---------- ---------- ---------- ---------- ---------- ---------- ---------- ---------- ---------- ----------

#TcGMC_B5_{GMC-B} ---------- ---------- ---------- ---------- ---------- ---------- ---------- ---------- ---------- ----------

#DmEO_B1_CG9504_{GMC-B} ---------- ---------- ---------- ---------- ---------- ---------- ---------- --

#DmGMC_B2_CG9509_{GMC-B} ---------- ---------- ---------- ---------- ---------- ---------- ---------- --

#DmGMC_B3_CG9512_{GMC-B} ---------- ---------- ---------- ---------- ---------- ---------- ---------- --

#AgGMC_B4_iso4_{GMC-B} ---------- ---------- ---------- ---------- ---------- ---------- ---------- --

#AgGMC_B4_iso3_{GMC-B} ---------- ---------- ---------- ---------- ---------- ---------- ---------- --

#AgGMC_B4_iso2_{GMC-B} ---------- ---------- ---------- ---------- ---------- ---------- ---------- --

#AgGMC_B4_iso1_{GMC-B} ---------- ---------- ---------- ---------- ---------- ---------- ---------- --

#AmGMC_B6_{GMC-B} ---------- ---------- ---------- ---------- ---------- ---------- ---------- --

#AmGMC_B7_{GMC-B} ---------- ---------- ---------- ---------- ---------- ---------- ---------- --

#AmGMC_B8_{GMC-B} ---------- ---------- ---------- ---------- ---------- ---------- ---------- --

#AmGMC_B9_{GMC-B} ---------- ---------- ---------- ---------- ---------- ---------- ---------- --

#AmGMC_B10_{GMC-B} ---------- ---------- ---------- ---------- ---------- ---------- ---------- --

#TcGMC_B5_{GMC-B} ---------- ---------- ---------- ---------- ---------- ---------- ---------- --

**Amino acid sequence alignment within the CG6142-like gene group**

#Dm_CG6142_{CG6142} ---------- ---------- ---------- ----MRFCGV LIICLWYIQV TWSQLLVDLA RDFETSFLNN ---------- ---RIPDTTR FL-------P

#Ag_CG6142like_1_{CG6142} ---------- ---------- ---------- ---------S PIWLVLFILT TPNLLADGHD DTFFQTLFDE TSVLFNNG-- -TPRIPDTKA FR-------K

#Ag_CG6142like_2_{CG6142} ---------- ---------- ---------- --MVRRTVGT RLPVLMAFLL TASPPTDAQQ FPVFETLFDE VSLLVRTGPN ASVPIPEAKR IR-------D

#Am_CG6142like_3_{CG6142} ---------- ---------- ---------- ---MLKFLII ALLPSSVQSI IPPAILKTIY LFLFGLLKGQ DD-------- ---SIPDQTR FS-------Q

#Tc_CG6142like_4_{CG6142} ---------- ---------- ---------- ------MKTI LCFTVLFLCA NSQNLLNDFR RFFGAS---- ---------- ---DVRDTAN FR-------K

#Dm_CG6142_{CG6142} EYDFIIVGAG SAGCVMANRL SEISSASVLL LEAGDQE--- -----TFISD VPLTAALTQM TR-YNWGYKA EP-------- -TEHACQGLK GGVCNWPKGR

#Ag_CG6142like_1_{CG6142} EYDFIIIGAG SGGSVMANRL SEVRDWNVLL LEAGKEG--- -----NMLTE VPLTAGLTTI TG-YNWGYKA DP-------- -MKGACLGLK GGVCNWPKGR

#Ag_CG6142like_2_{CG6142} EYDFVVIGAG SGGSVMANRL SEVRDWSVLL LEVGKEE--- -----NLISN VPLTAGLTTA TG-YSWGYRS DP-------- -MRNACRGLE QGVCYWPKGR

#Am_CG6142like_3_{CG6142} EYDFIVIGAG SAGSVLTNRL TENPQWNVLL LEEGKDE--- -----IFLTD IPLLAPALHV TD-YVRLHTS EPRPRNTD-- GTDGYCLSMK NGRCNLPGGR

#Tc_CG6142like_4_{CG6142} SYDFVVVGAG SGGCVVANRL SENPEWSVLL LEAGDDE--- -----NFLTD VPLIASLQTI TS-YNWGYKS ER-------- -LATACLGLI DGRCNMPRGK

#Dm_CG6142_{CG6142} GVGGTSLINF MLYTRGHRRD YDEWAA-ANN SGWSYDELLP YFRKSERIGI PELY-KS--- ----PYHGRN GQLDVQYTDY RSQLLKAFLK SG-REMGYE-

#Ag_CG6142like_1_{CG6142} GLGGTSLINF LIYTRGHRSD YDGWEQ-AGN PGWGYREVLQ YFKKSERVQI PELR-HS--- ----PYRSTA GLVDVEESQF ETPLLKRFIE AG-RDLGYM-

#Ag_CG6142like_2_{CG6142} GLGGTSLINF LLYGRGHQRD YDDWER-AGN YGWGYRDVRR YFEKAEQIKG ------Q--- ----PYN-PH GYLHIEESSF ETPMLGRYIE AG-KRFGYR-

#Am_CG6142like_3_{CG6142} AVGGSSVVNF MIYSRGSPND YDNWAA-QGN PGWSYQNVLP YFIKSENCKL LD-Q-DI--- ----RFHGKG GYLDVISSPY VSPLRECFLR GG-EELGYD-

#Tc_CG6142like_4_{CG6142} ALGGTSVINF LLYTRGTKQD FDQWAE-LGN PGWGYDQVLP YFIKSENCTK CREI-DG--- ----KYHGKS GYLSVEHPGY ESPLVKRFIK SG-EELGYK-

#Dm_CG6142_{CG6142} -ITDPNG-EH LMGFARSQAT IRN-GRRCST SKAFIQP-VV N-RKNLHISM KSWVTRLIID PIT--KTATG VEFV------ -----KQRQR YVVRARKEVI

#Ag_CG6142like_1_{CG6142} -ETDPNG-EI QLGFGKAQAT MRR-GRRCSA SKAYLVP-AS R-RPNLDISM YSRVTKVLID PVT--KHAYG VEFI------ -----KRRRR YVIRARKEVI

#Ag_CG6142like_2_{CG6142} -HIDPND-PV QLGFYKAQAT MVN-GERCSA ARAYLKP-VA D-RPNLDIST RSWATRILID PVT--KTAFG VEFT------ -----KNKRL HTVRVRKEVI

#Am_CG6142like_3_{CG6142} -VIDYNA-AN VIGFSTAQVH LRN-GRRVSA SKAFLRP-IR E-RKNFHLSK LSRATRIVID PKK--KVAVG VEFV------ -----KNGRK RFVSASKEII

#Tc_CG6142like_4_{CG6142} -NNDPSA-PY GLGFSKVLAT MRN-GMRCSA SKAFLKP-IL H-RTNLHVSI KTRVTKILID PST--KQAYG VQFW------ -----KNRRK FTVLATKEVV

#Dm_CG6142_{CG6142} LSAGTIASPQ LLMLSGIGPA EHLREHNITV MQDLP-VGYN LQDHITLNGL VFVVND---- ----STVNDA RLL-NPSDIF RYIFA--GQG PYTIPGGAEA

#Ag_CG6142like_1_{CG6142} LAAGAIASPQ LLMLSGVGPR EHLKEMGIPV VQDLP-VGYN MQDHLNLPGL VFPVNQP--- ----VTVRER DMR-SPRPII DYLVH--GRG PFTSPGGAEG

#Ag_CG6142like_2_{CG6142} LAAGAIASPQ LLMLSGVGPR EHLQQLDIPV VKDLR-VGYN LQDHQTLSGL VFTVNQP--- ----VTIRER DMR-RPAPFL SYLFA--RRG PFTVPGGAEG

#Am_CG6142like_3_{CG6142} LSTGTLNSPQ LLMLSGIGPK DHLESLNIDS IEDLQ-VGYN LQDHVSMSML TFLVNES--- ----VTIVEP RIASNLANIL DYFVK--GTG PLTVPGGAEC

#Tc_CG6142like_4_{CG6142} LSAGSINSPH LLMLSGVGPR DDLTRVGIPL LQNLK-VGYN LQDHMAMSAL VFFVNES--- ----ITVSDR GVQ-NPVDIF NYVFN--GRG PYTIPGGAEA

#Dm_CG6142_{CG6142} FAFVRTP--- ---------- -SSKFAKDYP DMELVLG-AG SLSGDRFGTM RNLLGITDEF YDYMFGDLQS --KETFGLVP VLLRPKSRGR ISLRSRN-PF

#Ag_CG6142like_1_{CG6142} VAFVKTN--- ---------- -ISFTPSDYP DIELVMG-TG AYNNDESGTL RATIGFTDQF YHSTYGSILG --KHAFSVSP VLMRPKSRGR ISLKSTN-PF

#Ag_CG6142like_2_{CG6142} IAFVKTN--- ---------- -NSRSPEDYP DVELVLG-TG AVNNDESGSL RHTFGMTREF YDRSFGSARG --QHAFGIAP VLMRPKSRGR VWLKSRN-PF

#Am_CG6142like_3_{CG6142} LAFIDTKEDR SIRLMKKFQV NNTKFQTNVP DIELVLG-IS ALTGDISGSY RGLLGLTNEF YKEVFTGYEG --YDAFSIVP VLLQPKSRGR VTLKSSD-PF

#Tc_CG6142like_4_{CG6142} LAFVQTK--- ---------- -YAKIG-GYP DIELVLG-AG ALNGDVYGSL RSLLGIPRSL FERVYAPHAY --KPAFSIAP VLMRPKSRGR VVIKDGN-PL

#Dm_CG6142_{CG6142} H-WPRMEPNF MQ--HPDDVR AMIEGIEMIL KLSR-SKPMA KMGTRFHDRP FPGCENLKFA S--------- EAYWKCCLRR YGSSLQHQSG TCKMGPAT--

#Ag_CG6142like_1_{CG6142} H-WPRMEGNF FA--DYDDLL VLREGVKLTV DLIE-SRSFR DVGARLHSTP FYGCEQHRFR S--------- DEYWECAIRR IGSTLQHQCG TCKMGPVT--

#Ag_CG6142like_2_{CG6142} H-WPHMEGNF FD--HPDDLA TMVEGIKLAV RIGE-SDSFA SYGARLLGTP FYGCEAHPFR S--------- DDYWRCCLRQ VGASIQHQSG TCKMGPAS--

#Am_CG6142like_3_{CG6142} D-RPIFETNY YD--HEDDLR TML--IFQAI EVAS-TKAFK RFNATLLPVA FPGCKHVPFG T--------- DPYWACVARQ VTTTLGHFVG TCKMGPRR--

#Tc_CG6142like_4_{CG6142} H-WPKLIPNY FE--NEEDVK TMVEGIKMAI TITQ-SRHFQ KYNITMITTP FPGCETVPFG S--------- DEYWACAVRH VATTLGHQVG TCKMGPPS--

#Dm_CG6142_{CG6142} ---DNTSVVD AQLR-IHGIR GLRVVDASVL PNVPAGHTNA IVIMVAEKAG DMIKDAWRMP ITPLSS---- ---------- ---------- ----------

#Ag_CG6142like_1_{CG6142} ---DPEAVVN PQLQ-VYGIK GLRVVDASII PTIPASHTNA VVFMIGEKAA DM-------- ---------- ---------- ---------- ----------

#Ag_CG6142like_2_{CG6142} ---DPDAVVD PELR-VHGVG GLRVVDASIF PVIPAAHTNG VVIMVGEKAA DMVKDYWNNH IP-------- ---------- ---------- ----------

#Am_CG6142like_3_{CG6142} ---N-SGVVD HRLR-VHGIN GLRVVDASII PTIVTGHTNA VAYMIAEKAA DMIKEDWKVL NTEFDRTFRK N--------- ---------- ----------

#Tc_CG6142like_4_{CG6142} ---DPDAVVD ERLR-VYGIK GLRVVDGSIM PNVVAGHTNA VIMMIGEKAS DMIKQEWARK ---------- ---------- ---------- ----------

#Dm_CG6142_{CG6142} ---------- ---------- ---------- ---------- ---------- ---------- ---------- ---------- ---------- ----------

#Ag_CG6142like_1_{CG6142} ---------- ---------- ---------- ---------- ---------- ---------- ---------- ---------- ---------- ----------

#Ag_CG6142like_2_{CG6142} ---------- ---------- ---------- ---------- ---------- ---------- ---------- ---------- ---------- ----------

#Am_CG6142like_3_{CG6142} ---------- ---------- ---------- ---------- ---------- ---------- ---------- ---------- ---------- ----------

#Tc_CG6142like_4_{CG6142} ---------- ---------- ---------- ---------- ---------- ---------- ---------- ---------- ---------- ----------

#Dm_CG6142_{CG6142} ---------- ---------- ---------- ---------- ---------- ---------- ---------- --

#Ag_CG6142like_1_{CG6142} ---------- ---------- ---------- ---------- ---------- ---------- ---------- --

#Ag_CG6142like_2_{CG6142} ---------- ---------- ---------- ---------- ---------- ---------- ---------- --

#Am_CG6142like_3_{CG6142} ---------- ---------- ---------- ---------- ---------- ---------- ---------- --

#Tc_CG6142like_4_{CG6142} ---------- ---------- ---------- ---------- ---------- ---------- ---------- --

**Amino acid sequence alignment within the CHD-like gene group**

#Human_CHD_{CHD} ---------- ---------- ---------- ---------- ---------- ---------- -MWCLLRGLG RPGALARGAL GQQQSLGARA LASAGSESRD

#Cele_CHD_{CHD} ---------- ---------- ---------- ---------- ---------- ---------M LQNHAARSLQ R---TSKRWI NWRDSMKEGT FSADIAENKP

#Ecol_CHD_{CHD} ---------- ---------- ---------- ---------- ---------- ---------- ---------- ---------- ---------- ---------M

#Human_CHD_{CHD} EYSYVVVGAG SAGCVLAGRL TEDPAERVLL LEAGPKDVLA GSKRLSWKIH MPAALVANLC DDRYNWCYHT EVQR------ -------GLD GRVLYWPRGR

#Cele_CHD_{CHD} --THIIVGAG SAGCVLANRL TEDPSNRVLL IEAGPVD--- --HKWDWRIH MPAALMYNLC SDTYNWHYHT TAQK------ -------NLG NRVFYWPRGR

#Ecol_CHD_{CHD} QFDYIIIGAG SAGNVLATRL TEDPNTSVLL LEAGGPD--- --YRFDFRTQ MPAALAFPLQ GKRYNWAYET EPEP------ -------FMN NRRMECGRGK

#Human_CHD_{CHD} VWGGSSSLNA MVYVRGHAED YERWQRQ-GA RGWDYAHCLP YFRKAQGHEL GASR------ -----YRGAD GPLRVSRGK- TNHPLHCAFL EATQQAGYP-

#Cele_CHD_{CHD} VWGGSSTLNA MCYVRGHAYD YNRWEKE-GA SGWNYANCLP YFKKAETYSD ATGPNDP--- -----YRGNN GPLYVKKGD- AENPLHKAWL NVGKEHPLG-

#Ecol_CHD_{CHD} GLGGSSLING MCYIRGNALD LDNWAQEPGL ENWSYLDCLP YYRKAETRDM GEND------ -----YHGGD GPVSVTTSKP GVNPLFEAMI EAGVQAGYP-

#Human_CHD_{CHD} LTEDMNG-FQ QEGFGWMDMT IHE-GKRWSA ACAYLHP--A LSRTNLKAEA ETLVSRVLFE GTR----AVG VEYVKNG--- --------QS HRAYASKEVI

#Cele_CHD_{CHD} WTNDMNG-EK QEGISTMDMT IHN-GERWSA SKAYVHP--I RNRPNLITSS GITCTRVLFD TNK----AIG IEFIRKLNFV GTDSIDSYSR EKIYCQGDVI

#Ecol_CHD_{CHD} RTDDLNG-YQ QEGFGPMDRT VTPQGRRAST ARGYLDQ--A KSRPNLTIRT HAMTDHIIFD GKR----AVG VEWLEGD--- ------STIP TRATANKEVL

#Human_CHD_{CHD} LSGGAINSPQ LLMLSGIGNA DDLKKLGIPV VCHLPGVGQN LQDHLEIYIQ QACTRPITLH ------SAQK PLRKVCIGLE WLWKF----T GEGATAHLET

#Cele_CHD_{CHD} LAGGAINTPQ LLMLSGVGPA DHLRSHEIPI VANLPGVGQN LQDHLEIYVQ QESTQPVTLY N---KSSWKF PHNMIKIGLE WFTNR----T GLGASSHLET

#Ecol_CHD_{CHD} LCAGAIASPQ ILQRSGVGNA ELLAEFDIPL VHELPGVGEN LQDHLEMYLQ YECKEPVSLY ------PALQ WWNQPKIGAE WLFGG----T GVGASNHFEA

#Human_CHD_{CHD} GGFIRSQ--- ---------- ----PGVPHP DIQFHFL--- ---------- ---------P SQVIDHGRVP TQQEAYQVHV GPMRGTSVGW LKLRSAN--P

#Cele_CHD_{CHD} GGFARSD--- ---------- ----DTVTHP DIQFHFL--- ---------- ---------P STVHDDGRTN GTCHGYQVHV GPMRSQSKGY IMLQAKD--P

#Ecol_CHD_{CHD} GGFIRSR--- ---------- ----EEFAWP NIQYHFL--- ---------- ---------P VAINYNGSNA VKEHGFQCHV GSMRSPSRGH VRIKSRD--P

#Human_CHD_{CHD} QDHPVIQPNY LS--TETDIE DFRLCVKLTR EIFAQEALAP FRGKELQPGS HIQSD----- ---------- -KEIDAFVRA KADSAYHPSC TCKMGQP---

#Cele_CHD_{CHD} RRAPIINPNY ME--EDSDWR EFRKCIRVSR ELFASKAFDE FRGKELAPGP DCQSD----- ---------- -ADIDRFVKE KAASAYHPSC TCKMGSE---

#Ecol_CHD_{CHD} HQHPAILFNY MS--HEQDWQ EFRDAIRITR EIMHQPALDQ YRGREISPGV ECQTD----- ---------- -EQLDEFVRN HAETAFHPCG TCKMG-----

#Human_CHD_{CHD} --SDPTAVVD PQTR-VLGVE NLRVVDASIM PSMVSGNLNA PTIMIAEKAA DIIKGQPALW D-KDVPVYKP RTLATQR--- ---------- ----------

#Cele_CHD_{CHD} --NDKMAVVN PETMGVYGTE NLKVVDASVM PSIVSGNLNA PVIMMAERAA DLIKHKKQLL PPSDANVWHH N--------- ---------- ----------

#Ecol_CHD_{CHD} --YDEMSVVD GEGR-VHGLE GLRVVDASIM PQIITGNLNA TTIMIGEKIA DMIRGQEALP R-STAGYFVA NGMPVRAKK- ---------- ----------

#Human_CHD_{CHD} ---------- ---------- ---------- ---------- ---------- ---------- ---------- ---------- ---------- ----------

#Cele_CHD_{CHD} ---------- ---------- ---------- ---------- ---------- ---------- ---------- ---------- ---------- ----------

#Ecol_CHD_{CHD} ---------- ---------- ---------- ---------- ---------- ---------- ---------- ---------- ---------- ----------

#Human_CHD_{CHD} ---------- ---------- ---------- ---------- ---------- ---------- ---------- --

#Cele_CHD_{CHD} ---------- ---------- ---------- ---------- ---------- ---------- ---------- --

#Ecol_CHD_{CHD} ---------- ---------- ---------- ---------- ---------- ---------- ---------- --

**Amino acid sequence alignment within the Insect GLD-like gene group**

#AmGLXr_1_{Insect_GLD-GOX} ---------- ---------- ---------- ---------- ---------- ---MASTCG- -VKTSFMSLV EKVIASTCDI ANPCNR---- -------LEH

#TcGLXr_3_{Insect_GLD-GOX} ---------- ---------- ---------- ---------M ECGC-AAPYI GPSLANTCGG GAFVLFMSLL DTFIRNKCDL SEICQRVVPK TQ-----PDI

#TcGLXr_4_{Insect_GLD-GOX} ---------- ---------- ---------- ---------M SCCA-NEPYI GPPLDRTCFG GSYIVFMHLL NTLITQQCDV SEICQRINPQ LQ-----PDS

#TcGLD_{Insect_GLD-GOX} ---------- ---------- ---------- ---------M ACNCPVTQ-P GPTLASTCGG AQYMLFMGLL EVFLRSQCDL EDPCGRPHNT P------VLP

#AmGLD_{Insect_GLD-GOX} ---------- ---------- ---------- ---------M SCNCPLNPST GPTLASTCGG SSFMLFMGLL EVFLRSQCDL EDPCNRPLPP PT-----VNS

#DmGLD_{Insect_GLD-GOX} ---------- ---------- ---------- ----MSASAS ACDCLVGVPT GPTLASTCGG SAFMLFMGLL EVFIRSQCDL EDPCGRASSR FRS---EPDY

#AgGLD_{Insect_GLD-GOX} ---------- ---------- ---------- --------MS SCACPMTSPV GATLAALCGG TQYMLFMGLL EVFIRSQCDL EDPCGRTKAK SSR---NVDY

#AmGLXr_2_{Insect_GLD-GOX} ---------- ---------- ---------- ---MSYNLSI SPICPDPNLG -PSLAQVCPG PQFLTFMSLF NTFALAKEEV SLLCQRFEPV EP-----AEY

#AmGOX_{Insect_GLD-GOX} ---------- ---------- --------MA ILNSMYNNVS PLQCTSPFLG GPQLTDVCSA SNGELFLALL NFFVATSPVI GEPCQRVHSS RI-----PDL

#AmGLXr_1_{Insect_GLD-GOX} RFDFIVVGAG VAGPVIARRL SDNPWWRVLL IEAGPEE--- -----PSMTS IPGLAVHAVN -STLDWRFKT EPTE------ PHPTACLEN- DGVCSWPRGK

#TcGLXr_3_{Insect_GLD-GOX} EYDFVVIGGG SGGATAAGRL SEVPEWKVLL IEAGGDE--- -----PPGSQ VPSMVISYHG DPHMDWNYKT E--------- PEQQACLGFP EKRCSWPRGK

#TcGLXr_4_{Insect_GLD-GOX} EYDFVVIGGG AGGSVVAGRL SENPNWKILL IEAGGDE--- -----PPGSQ VPSMMNNYLG DSQMDWRYRT E--------- PQEMACLGRP GRRCDWPRGR

#TcGLD_{Insect_GLD-GOX} EYDFIIVGGG SSGAVVASRL SEIPEWNVLL IEAGLDE--- -----PTGTQ VPSMFLNFIG -SEIDWGYQT E--------- PEPSACLAET EQRCYWPRGK

#AmGLD_{Insect_GLD-GOX} RYDFVVIGGG SAGATVASRL SEEPRFSVLL LEAGLDE--- -----PTGTQ IPSFFFNFIG -TDIDWQYNT E--------- SEDTACLNKD DRKCYWPRGK

#DmGLD_{Insect_GLD-GOX} EYDFIVIGGG SAGSVVASRL SEVPQWKVLL IEAGGDE--- -----PVGAQ IPSMFLNFIG -SDIDYRYNT E--------- PEPMACLSSM EQRCYWPRGK

#AgGLD_{Insect_GLD-GOX} EYDFIVVGGG SGGSVIASRL SEIKNWKVLL IEAGPDE--- -----PTGAQ IPSMFLNYLG -SDIDWKFNT E--------- PEQYACLGSP EQRCYWPRGK

#AmGLXr_2_{Insect_GLD-GOX} YYDFIVVGGG TAGSVVASRL SEQREWKVLL LEAGPDE--- -----PPGTD VPSMVAMFLG -SDIDWGYRT T--------- NEKNACLSS- GGSCFWPRGK

#AmGOX_{Insect_GLD-GOX} SYDFIVVGGG AARAVVAGRL SEVSNWKVLL LEAGPDE--- -----PAGAE IPSNLQLYLG -GDLDWKYYT T--------- NESHACLST- GGSCYWPRGK

#AmGLXr_1_{Insect_GLD-GOX} MMSGTAGMYG MMYSRGHPEV YNGWAR-GGA TGWSYDEVTH YFERAEDPID QSILSDKP-- ----RTVPVP GPMKIQFYPD KPAFADEILK AA-SELGYRT

#TcGLXr_3_{Insect_GLD-GOX} VLGGCSVING MMYMRGHPKD YDNWAT-MGN TGWGYQDVLP VFKKSEDNLQ IGTLVDA--- ----AYHGTG GPMTTSRFPH HPELAEDVMQ AA-KELGYPV

#TcGLXr_4_{Insect_GLD-GOX} VLGGSGVIHG MMYMRGLPSD YNEWEA-RGN EGWGYKDVEE YFKKSEGNRD IGDGVEG--- ----RYHSSD GPMLVQRFPD QPQIAEDVLR AG-AELGYPV

#TcGLD_{Insect_GLD-GOX} VLGGTSVMNG MMYIRGSRKD YDDWAK-AGN EGWSYNEVLP YFLKSEDNKQ A-DSMDR--- ----GYHSTG GLLTVSQFPY HPPLSQALLK AA-QELGYPI

#AmGLD_{Insect_GLD-GOX} VLGGTSVMNG MMYIRGSRKD YDDWAR-LGN IGWSYQDVLP YFIRSEDNLQ A-NTMDY--- ----GYHGVG GPLTVTQFPY HPPLSYSILE AG-KELGYGI

#DmGLD_{Insect_GLD-GOX} VLGGTSVLNG MMYVRGNRED YDDWAA-DGN PGWAYNDVLP FFKKSEDNLD L-DEVGT--- ----EYHAKG GLLPVGKFPY NPPLSYAILK AG-EELGFSV

#AgGLD_{Insect_GLD-GOX} VLGGTSVLNG MMYIRGNPQD YDDWDA-MGN PGWKWKDVLP YFMKSEDNLQ I-NEVDS--- ----KYHSTG GMLPVGRFPY NPPFSYSVLK GG-EQLGYQV

#AmGLXr_2_{Insect_GLD-GOX} NLGGTSSHNG MMYTRGHPKD YDDWAA-MGN DGWSWQDVLP YFMCSENNTE I-NRVGR--- ----KYHSTG GLLNVERFSW RPDISNDILA AA-AELGYPI

#AmGOX_{Insect_GLD-GOX} NLGGTTLHHG MAYHRGHRKD YERWVQ-QGA FGWSWDEVMP YYLKSENNTE L-SRVGT--- ----KYHRSG GLMNVERFPY QPPFAWKILK AA-EEAGFGV

#AmGLXr_1_{Insect_GLD-GOX} S-KLKEY--T QTGFMIAPMT TDN-GVRGTA TRNYLRP-VH G-RSNLRVLI NAHVTKVLMD WQG---KAYG VELVD----- -----KDGYK RIAKANKEVV

#TcGLXr_3_{Insect_GLD-GOX} SDDLNGR--Q YHGFTIAQSS VRN-GSRLSS ARAFLRP-GR D-RPNLHVML NSTATKILIN SSNNQKTVSG VQFLY----- -----N-NKL HTVRVKREVV

#TcGLXr_4_{Insect_GLD-GOX} VGDLNGE--Q HWGFTIAQAN IKN-GSRLSS ARAFLRP-AR N-RPNLHVMI NSTATKILIN SNDTAKTISA VEFTY----- -----N-NQS FTVKVRREAI

#TcGLD_{Insect_GLD-GOX} R-DLNGA--Y HTGFNIAQTT NRN-GSRLST AKAFLRP-FK N-RRNLNILM NSTVTRVLIN TTT--KQAYG VEVIN----- -----N-GVK QVIYASKEVI

#AmGLD_{Insect_GLD-GOX} A-DLNGR--T HTGFAIAQTT SRN-GSRLST ARAFLRP-AK N-RPNLHIML NSTATRILFD NN---KRAVG VEFVH----- -----D-GKI HRVSVAKEVV

#DmGLD_{Insect_GLD-GOX} H-DLNGQ--N STGFMIAQMT ARN-GIRYSS ARAFLRP-AR M-RNNLHILL NTTATKILIH PHT--KNVLG VEVSD----- -----QFGST RKILVKKEVV

#AgGLD_{Insect_GLD-GOX} Q-DLNGA--N TTGFMIAQMT NKN-GIRYSA ARAFLRP-AV N-RANLHILL NTTVTKVLVH PTS--KTAHG VEIVD----- -----EDGHM RKILVKKEVI

#AmGLXr_2_{Insect_GLD-GOX} PEELNGD--Q FAGFTVAQMM SKD-GVRRST ATAFLRP-FR N-RSNLQVIT NATVTKILLK EKK----AVG VQYYK----- -----N-GEL RVARASREII

#AmGOX_{Insect_GLD-GOX} SEDLSGD--R INGFTVAQTI SRN-GVRLSS ARAFITP-FE N-RSNLHVIV NATVTKVRTL NKR----ATG VNVLI----- -----N-GRR RIIFARREVI

#AmGLXr_1_{Insect_GLD-GOX} LTGGTIGSAH ILLNSGIGPK DQLTKLGMHV VKDLP-VGKN LHNHVSIGVQ FSIK------ ------DTAY EAMTMNSVNE YLETR---TG PMTSTGLTQV

#TcGLXr_3_{Insect_GLD-GOX} VSAGAINSPQ ILLLSGIGPK EELDKVNIQQ VHQLPGVGKN LHNHVTFYMT YEMKK----- ------QKAV HDLDWAHALD YILNR---RG PMSSTGMSQV

#TcGLXr_4_{Insect_GLD-GOX} VSAGAINTPH LLLLSGIGPR EELDKVGIEQ VHNLPGVGQN LKNHVSFAVN FQLTK----- ------IENY NDLNWNTVRE YLTER---RG PMSSTGVTQV

#TcGLD_{Insect_GLD-GOX} VSGGAINSPQ ILLLSGIGPS QDLQQVNVPV VHNLPGVGKN LQNHVAHFVN FNIN------ ------DTNS APLNWATAME YLLFR---DG LMSGTGISEV

#AmGLD_{Insect_GLD-GOX} ISGGAVNSPQ ILLNSGIGPR EELNAVGVPV IHDLPGVGKN LHNHVAYTLA FTIN------ ------DTDT TPLNWATAME YLLFR---DG LMSGTGISEV

#DmGLD_{Insect_GLD-GOX} LSAGAVNSPH ILLLSGVGPK DELQQVNVRT VHNLPGVGKN LHNHVTYFTN FFID------ ------DADT APLNWATAME YLLFR---DG LMSGTGISDV

#AgGLD_{Insect_GLD-GOX} VSGGAVNSPQ ILLLSGIGPR EHLEKVGVRP IHDLPGVGKN LHNHVAYFIN FFLN------ ------DTNT APLNWATAME YLLFR---DG LMSGTGVSAV

#AmGLXr_2_{Insect_GLD-GOX} ISGGAVNSPQ ILLLSGIGPK EHLEAVNVSV VHDLPGVGEN LHNHVSFTLP FTIN------ ------RPNE FDLSWPSLLE YIAFT---KG PIASTGLSQL

#AmGOX_{Insect_GLD-GOX} LSAGSVNTPQ LLMLSGIGPK EHLRSLGIPV VVDLPGVGEN LHNHQSFGMD FSLN------ ------EDFY PTFNQTNVDQ YLYNQ---TG PLSSTGLAQV

#AmGLXr_1_{Insect_GLD-GOX} TAFFESS--- ---------- -YAV--TGIP DIQVFFD--- -------GFA PRCPRTGLEF ECLNGALGLC PERRQINVRP TALTAASKGY LKLRSSD-PL

#TcGLXr_3_{Insect_GLD-GOX} TARINSK--- ---------- -FADPSGTHP DLQIFFA--- -------GYL ANCAASGEVR AAKD--PEHP DAPRHLTISP VVLHPKSRGH IGLKSNN-PL

#TcGLXr_4_{Insect_GLD-GOX} AARISSK--- ---------- -YANPDGKNP DLQFFFS--- -------GFL AHCSLSGGVK EPED--PTNP TAAKSFTIRP TFLRPRSRGF IGLNSRD-PK

#TcGLD_{Insect_GLD-GOX} TGFINTK--- ---------- -YNDPRLEHP DIQLFFG--- -------GFL ANCARTGQVG ------ERVD NGTRQIQMIP TVLHPKSRGV LKLRDNN-PL

#AmGLD_{Insect_GLD-GOX} TAMINTK--- ---------- -YANPKDDHP DVQLIFG--- -------GYL ADCAETGMVG ------ETKG N-NRTIYIIP TYLHPKSRGY LRLRNND-PL

#DmGLD_{Insect_GLD-GOX} TAKLATR--- ---------- -YAD-SPERP DLQLYFG--- -------GYL ASCARTGQVG ------ELLS NNSRSIQIFP AVLNPRSRGF IGLRSAD-PL

#AgGLD_{Insect_GLD-GOX} TAKISSK--- ---------- -YAE-RPDDP DLQFYFG--- -------GFL ADCAKTGQVG ------ELLS NDSRSVQIFP AVLHPKSRGY IELKSND-PL

#AmGLXr_2_{Insect_GLD-GOX} TGIVSSI--- ---------- -YTS--EDDP DLQIFFG--- -------GYQ AACATTGQLG ------ALMD GGGRHVSISP TNLHPRSRGS LRLASND-PF

#AmGOX_{Insect_GLD-GOX} TGIWHSN--- ---------- -LTT--PDDP DIQIFFA--- -------GYQ AICKPKLKIA ------DLSA HDKQAVRMSA LNVQPTSKGR ITLNSKD-PL

#AmGLXr_1_{Insect_GLD-GOX} A-PPLIYPNY FV--DTKDLK VLVEGIKKSI QLVDTQALK- QWDFRLDTVV HPMCT-DYHF GS-------- DAYWECYVRA ATGPENHQSG TCKMGAYD--

#TcGLXr_3_{Insect_GLD-GOX} D-PPLMYANY LS--EPEDVA TLVEGIRVTQ RLANTSVLQN KYGLTLMRDE YGDCEKKFTY DS-------- DDFWQCAARY YTGPENHQAG SCKMGPAS--

#TcGLXr_4_{Insect_GLD-GOX} E-PPLMQPNY LT--DEEDVK RMVAGIRIAQ NLANTTILTT KYGIQMVNTD YGDCSRNYTF DS-------- DEFWACALRY DTGPENHQSC SCKMGPAS--

#TcGLD_{Insect_GLD-GOX} S-TPLIYANY FT--HPNDVK VITEGIKFAM KLSETKALK- RYGFQLDRTP VQGCE-SLTF GT-------- DPYWDCAVKR QTGPENHQAG SCKMGPSS--

#AmGLD_{Insect_GLD-GOX} S-KPLIYPKY LS--HPDDVA GLIEAIKFSI RLSETEALS- RYGFQLDRTP VKNCE-HLEF GC-------- DAYWECAVKH DTAPENHQAG SCKMGPPD--

#DmGLD_{Insect_GLD-GOX} E-PPRIVANY LT--HERDVK TLVEGIKFVI RLSQTTPLK- QYGMRLDKTV VKGCE-AHAF GS-------- DAYWECAVRQ NTGPENHQAG SCKMGPSH--

#AgGLD_{Insect_GLD-GOX} E-HPKIVVNY LK--EDHDVK VLVEGIKFAV RLSETDALQ- AYGMDLDRTP VKACQ-DKDF GS-------- QEYWECAVRQ NTGAENHQAG SCKMGPTS--

#AmGLXr_2_{Insect_GLD-GOX} A-KPVIHGNY LS--DPMDEA VLLHGIRIAL SLSNTSALA- RYNMTLANLP LPACS-QHTY LS-------- DDYWRCAMRQ DTGPENHQAG SCKMGPVS--

#AmGOX_{Insect_GLD-GOX} D-PPVIWSND LA--TEHDRS VMIQAIRVVQ KLVNTTVMR- DLGVEFQKIE LKQCD-EFVE DS-------- DDYWNCVIQY NTRAENHQTG TAKMGPSY--

#AmGLXr_1_{Insect_GLD-GOX} ---DPTAVVD PELR-VRGVS NLRVADASVF PLVPNGNPVA AILMVAEKAA DMITHAWSKI ---------- ---------- ---------- ----------

#TcGLXr_3_{Insect_GLD-GOX} ---DPMAVVD PKLQ-VYGIE GLRVMDASIM PALVSGNTHA TIVMIADKGV EYIKQKWLRG GTIANRFGGG TSQSNQNAPH FYPSASSNYP NYPKQHAPYH

#TcGLXr_4_{Insect_GLD-GOX} ---DPSAVVD PKLQ-VHGIE GLRIMDASVM PTVLSGNTHA TVVMIAEKGS DYIKQKWSDK ---------- ---------- ---------- ----------

#TcGLD_{Insect_GLD-GOX} ---DPMAVVN PMLQ-VHGID RLRVIDASIM PAVTTGNTNA PCIMIAEKGS DLIKSRWLTP QAGFFYTNMP NQRIDRQWGS W--------- ----------

#AmGLD_{Insect_GLD-GOX} ---DPLAVVD NQLR-VRGVR GVRVADTSIM PRVISGNTNA PAIMIGERAA DFIKRTWVG- ---------- ---------- ---------- ----------

#DmGLD_{Insect_GLD-GOX} ---DPMAVVN HELR-VHGIR GLRVMDTSIM PKVSSGNTHA PAVMIAEKGA YLLKRAWGAK V--------- ---------- ---------- ----------

#AgGLD_{Insect_GLD-GOX} ---DPLAVVD HELR-VHGVR NLRVVDASVM PKVTSGNTNA PIIMIAEKGA HLIRRAWGAR ---------- ---------- ---------- ----------

#AmGLXr_2_{Insect_GLD-GOX} ---DRMAVVD PRLR-VHGVD GLRVADTSIM PKVTSGNTAA PAIMIGERAA AFVKSDWGGA PAKWYGHTSS SHLWREA--- ---------- ----------

#AmGOX_{Insect_GLD-GOX} ---DPMAVVS PRLK-VHGIR GLRVADASVQ PQVISGNPVA SVNMVGERAA DFIKEDWG-- ---------- -ELLQLL--- ---------- ----------

#AmGLXr_1_{Insect_GLD-GOX} ---------- ---------- ---------- ---------- ---------- ---------- ---------- ---------- ---------- ----------

#TcGLXr_3_{Insect_GLD-GOX} YQQGVKSTGF RHNEQFHRNH PQMPNPFMST PRPQRAYNQQ GFNQGYQGYQ HNYPDYQQDQ EYNNFNAY-- ---------- ---------- ----------

#TcGLXr_4_{Insect_GLD-GOX} ---------- ---------- ---------- ---------- ---------- ---------- ---------- ---------- ---------- ----------

#TcGLD_{Insect_GLD-GOX} ---------- ---------- ---------- ---------- ---------- ---------- ---------- ---------- ---------- ----------

#AmGLD_{Insect_GLD-GOX} ---------- ---------- ---------- ---------- ---------- ---------- ---------- ---------- ---------- ----------

#DmGLD_{Insect_GLD-GOX} ---------- ---------- ---------- ---------- ---------- ---------- ---------- ---------- ---------- ----------

#AgGLD_{Insect_GLD-GOX} ---------- ---------- ---------- ---------- ---------- ---------- ---------- ---------- ---------- ----------

#AmGLXr_2_{Insect_GLD-GOX} ---------- ---------- ---------- ---------- ---------- ---------- ---------- ---------- ---------- ----------

#AmGOX_{Insect_GLD-GOX} ---------- ---------- ---------- ---------- ---------- ---------- ---------- ---------- ---------- ----------

#AmGLXr_1_{Insect_GLD-GOX} ---------- ---------- ---------- ---------- ---------- ---------- ---------- --

#TcGLXr_3_{Insect_GLD-GOX} ---------- ---------- ---------- ---------- ---------- ---------- ---------- --

#TcGLXr_4_{Insect_GLD-GOX} ---------- ---------- ---------- ---------- ---------- ---------- ---------- --

#TcGLD_{Insect_GLD-GOX} ---------- ---------- ---------- ---------- ---------- ---------- ---------- --

#AmGLD_{Insect_GLD-GOX} ---------- ---------- ---------- ---------- ---------- ---------- ---------- --

#DmGLD_{Insect_GLD-GOX} ---------- ---------- ---------- ---------- ---------- ---------- ---------- --

#AgGLD_{Insect_GLD-GOX} ---------- ---------- ---------- ---------- ---------- ---------- ---------- --

#AmGLXr_2_{Insect_GLD-GOX} ---------- ---------- ---------- ---------- ---------- ---------- ---------- --

#AmGOX_{Insect_GLD-GOX} ---------- ---------- ---------- ---------- ---------- ---------- ---------- --

**Amino acid sequence alignment within Nina G gene subfamily**

#DmNinaG_CG6728_{NinaG} ---------- ---------- ---------- ---------- ---------M GMKFQKILVL AGIVIGFLSI IVVLAGTLLK NSVPNVLAPV ER------HF

#Tc_NinaG_like_{NinaG} ---------- ---------- ---------- ---------- -------MCC SNPAFYISVY VLAVNLFGLY LRFVYFHNYF ECFACRELDF KD-------Q

#DmNinaG_CG6728_{NinaG} AFDYVIVGGG TGGSTLTSLL AKNSNGSVLL IEAGGQFG-- ------LLSR IPLLTTFQQK GI-NDWSFLS VPQK------ ---HSSRGLI ERRQCLPRGK

#Tc_NinaG_like_{NinaG} AYDYIVVGSG SAGSIVARRL AENPSVKVLL IEAGASG--- -----NGILQ IPTVSLMLQD SV-FDWQYRT VPQK------ ---HACLGLD KKVSHWPMGK

#DmNinaG_CG6728_{NinaG} GLGGSANLNY MLHFDGHGPD FDSWRDHHNL SDWSWAQMRS FMAAAKPKNP ---------- ---------- ---DMLEIPR RYSKLTEALE EAQAQFAYK-

#Tc_NinaG_like_{NinaG} ILGGTAMLNN MIYVRGHPQD FAEWYKDSCN FNYT-IDVLP YFKKLESNET NKHKCS---- ---------- --VFVEDMPF KSNLSDYFLQ AG-LCLGFG-

#DmNinaG_CG6728_{NinaG} ---------- DWIFRRSLYN IRN-GLRHSV VQQFLNP--V IHHSNLRLLP DALVKRIQLA PSPFLQATSI LVGIKDE--- ----ENREKE FSIEVRRELI

#Tc_NinaG_like_{NinaG} ---LSDGVNS EPGFSATKVT MRN-GQRWTP YHQLEKT--- -KKRNLVVIT NSLVEKVLLK SNY---EAYG VKYT------ -----HLDET YYVRATKGVI

#DmNinaG_CG6728_{NinaG} LCAGAYQTPQ LLMASGIGDV SALKKLGIPA QHSLPLVGHN LHDHFNLPLF VSMGVTG--- ----PTLNQN TLLNPMTLIN YLSSG---SG PLGNFGVLGN

#Tc_NinaG_like_{NinaG} LSAGVIGSPK ILMLSGIGPK KHLEKLKIAP RLDLP-VGEN LQDHVTTGLD LITLEAP--- ----PDMGLQ QMLSPWSASR YFLWG---EG PWTSPG-CES

#DmNinaG_CG6728_{NinaG} VVSYGGLG-- ---------- -------APP YGITFFG--- -AGAIDESAL MSISNFKGPA FRALFPRYYN SSQEGFVVIS SCLQPKSRGS VGLLNRH--M

#Tc_NinaG_like_{NinaG} VGFFNSE--- ---------- -----DEKIP ELQFMIL-PY GAAIDGGSYL RGLVGIGERL WEGYFRRVNG ---STMTVLP VVLHPKSRGT VRLKSKD--P

#DmNinaG_CG6728_{NinaG} RRNPLIDPNY LS--SEEDVA CTISAIRSAV ELVN-STAFA ALHPRIHWPR VQECSNFGPF ERDFFDNRPS DQYLECLMRH VGLGSHHPGG TCALG-----

#Tc_NinaG_like_{NinaG} RTPPLIDPNY LA--EGYDVD ILLEGIELVK EFLE-TPPMR RLGAKLNAVK FPGCEGLEFD T--------- RPYWVCYVRH FTLSSYHPVG TCALGR----

#DmNinaG_CG6728_{NinaG} ------SVVD SQLR-LKGVS NVRVVDASVL PRPISGNPNS VVVAIALRAA SWILKSELQA GDSK------ ---------- ---------- ----------

#Tc_NinaG_like_{NinaG} -------VID EGFQ-VKGTN KLYVVDGSVL PSLPSGNPQG AIMMMAERAA EIIKHHCWLS QRRCCSSDVF QDQCSCY--- ---------- ----------

#DmNinaG_CG6728_{NinaG} ---------- ---------- ---------- ---------- ---------- ---------- ---------- ---------- ---------- ----------

#Tc_NinaG_like_{NinaG} ---------- ---------- ---------- ---------- ---------- ---------- ---------- ---------- ---------- ----------

#DmNinaG_CG6728_{NinaG} ---------- ---------- ---------- ---------- ---------- ---------- ---------- --

#Tc_NinaG_like_{NinaG} ---------- ---------- ---------- ---------- ---------- ---------- ---------- --

**Amino acid sequence alignment within other Beetle GNC gene group**

#Tc_XM_961446_{otherBeetleGMC} ---------- ---------- ---------- -------MSG KLLLVVLLTF SANSHAYYYQ D-KIEYYVQL ITGAFRNALT TVLPTDSYQY FSGEVRRSYG

#Tc_XM_961538_{otherBeetleGMC} ---------- ---------- ---------- -------MNL KILRVVLPVM FVYAQGYLSE Q-KIDYFVRL ISKANENAMT YQLPKNAYEY YTKDRQQKFG

#Tc_XM_968249_{otherBeetleGMC} ---------- ---------- ---------- -------MN- KDLSVCILLF CAVLQAHTDN Q-TVQYYVDL ISEAYENGLT YVFPTSAYEY YSDTIPKKYG

#Tc_XM_967481_{otherBeetleGMC} ---------- ---------- ---------- ---------- --MWATLSVF IAILPKIITS DPAIDFYKKV IDENVAKVES YKLPD----- ---------G

#Tc_XM_961446_{otherBeetleGMC} SFDFVVIGAG AAGAVIANRL TEVEDWNVLV LEAGGYG--- -----NDFSD IPDMYWPIEF TD-FNWGYNS TPQR------ ---TACLGLI DQECFYPRGR

#Tc_XM_961538_{otherBeetleGMC} TFDFVVIGAG AGGTVVANRL SEVANWNILV LEAGGYG--- -----NDFSD IPNMYFPIQF SH-FNWGYNS TPQT------ ---TACLGLE NHVCLYPRGK

#Tc_XM_968249_{otherBeetleGMC} TFDFVVIGSG AAGSVAASRL SEINKWSVLV LEAGTFW--- -----NNFSD IPNMYEPIAF TH-FNWEFNS TPQT------ ---TACLGLV NQICNYFFFK

#Tc_XM_967481_{otherBeetleGMC} NYDFIIIGAG SAGSVLATRL SENENWKILL LEAGGEE--- -----NDFST IPSMWANLQM SE-INWGYRT ISQK------ ---NCCLGMK NRQCLEPRGK

#Tc_XM_961446_{otherBeetleGMC} GVGGSTLING LIYSRGHKTD FDHWGRLVGN DRWSYRSVLQ YFKKSENFVY RDYTQPIE-- ---PEYHGTN GYWQVEHHLP RSPQLDVFLD AN-REMGLGV

#Tc_XM_961538_{otherBeetleGMC} GIGGSTLING LVYSRGHKTD FDKWGEVVGS KRWSYNKVLK YFKKSEDFVY RDYEVPYE-- ---PQYHGTG GYLRVENYIY RSPQLNAFLA AN-QELGLGV

#Tc_XM_968249_{otherBeetleGMC} GVGGSTLING LVYARGHKSD FDKWGKVAGN RRWSYETVLK YFKKSENFVY RDADAPYE-- ---PPYHGEG GDLQVEYHLP RSPQLNAWLE AN-RELGYEI

#Tc_XM_967481_{otherBeetleGMC} AIGGSSTINA IMYVRGNPED YNEWVR-LGN PGWSYEEVLP YFLKSE---- -NSQVEGD-- ---PGFHGKG GLWNIQYSLP PSELFSNFLQ AN-KELGLEA

#Tc_XM_961446_{otherBeetleGMC} ADYNAN---- RLGASSAQLN TAF-GRRMDT GKAFIRS-VL K-RPNLKVLT GSFVTRIVID KFT--RSAVG VEFT------ -----HGGSN YFVRAKKEVI

#Tc_XM_961538_{otherBeetleGMC} VDYNAN---- KLGASASQLN THN-GRRFDG GKAFIHP-VL N-RPNLKVLT GSYVTRIVIN KET--KSATG VEFT------ -----HDGKY YYVEAKKEVI

#Tc_XM_968249_{otherBeetleGMC} VDYNAN---- RLGASPSQLN TRN-GRRDDD GQAFLRH-AR K-RRNLKILT GSYVTKIQIE KE----SANG VEFT------ -----HKGKN YYVEVRKEVI

#Tc_XM_967481_{otherBeetleGMC} VDYNGYR--- QFGASKAQTN IKH-GKRQST GTAFLKY-AR Q-RRNLNVIT NALVTEIVID KKN--KSAEG VMFI------ -----KDNQK FRANANLEVI

#Tc_XM_961446_{otherBeetleGMC} LSAGAFNTPQ LLMLSGIGPG YHLQELGIEV IQDLE-VGST LRDNPTFYGV AFQTNYT--- ---------- --EPIEPLEN YIEQYFQGVG PLAIPGNNQG

#Tc_XM_961538_{otherBeetleGMC} LCAGAFGSPQ ILMLSGVGPK KHLQDVGIEV IKDLE-VGST LRDNPTFFGL NFGTNYT--- ---------- --EPVRPLKD YVLQYLEGVG PLTIPGSNQG

#Tc_XM_968249_{otherBeetleGMC} LSAGVFGTPQ ILMLSGVGPR KHLEEKGIEV IKDLE-VGST LRDNPTFYGL NYGTNYT--- ---------- --EPIRPLAD YVKEYLNGVG PLAIPGSTQG

#Tc_XM_967481_{otherBeetleGMC} VSAGAFNSPQ LLMLSGIGPK EHLEELGIDL IEDLP-VGQN LLEHPMFSGL AFRTNFT--- ---------- --VTAE---- ---------- ----------

#Tc_XM_961446_{otherBeetleGMC} VGFYESS--- ---------- --YTRGTGIP DLEFMFI--- -PAVASTILQ QRAFRLTDQT YNDVYQFQDV G--STFGVYV IVLHSKSVGT VRLRSRD-PF

#Tc_XM_961538_{otherBeetleGMC} VGFYESS--- ---------- --YTKGTGIP EIELMFI--- -PANATSNLS QRSFGLTDET YEDVWKYANI P--QTFLFYV VDLHSQSVGT VRLKSKN-PF

#Tc_XM_968249_{otherBeetleGMC} VGFYESS--- ---------- --YSKGTGIP DIELMIA--- -VANATDQLT QRYFSLTDQT YEDVWKYNNI P--QTFIFHV VNLHAQSSGS VRLKSKN-PF

#Tc_XM_967481_{otherBeetleGMC} ---------- ---------- ---SPGT-VP PIEYIFL--- -PQTGTPSAF D-MFNFNQEL ENSYLAKINS S--TDFNIFV VLLHQKSKGQ IRLKSKN-PT

#Tc_XM_961446_{otherBeetleGMC} Q-FPLIDANF LSDPENKDIN VLYEGVQLLM QMA-QTRAFR SMDATLAGGQ LSACSQYEFL S--------- REYWYCAIRQ LTINVYHPLG TCPMGRDP--

#Tc_XM_961538_{otherBeetleGMC} E-YPLIDSRF LSDPEDRDIN TLYEGVQLAL KLT-QTRPFK AINATLQGGP LRACKHFPYL S--------- KPYWYCALRQ LTINLYHPLG TCPMGKDP--

#Tc_XM_968249_{otherBeetleGMC} E-YPVINSNF LSDPESRDIN TLYEGIQICL KMG-ETKAMK AINATLQGGP LRACKRYQYL S--------- KDYWYCVLRQ ITVNLYHPLG SCPMGKDP--

#Tc_XM_967481_{otherBeetleGMC} D-FPEIDLNL FEEQE--DVD TFIDGINFVI KLT-ETQAFR DVNATLID-- IPICQEYEKY S--------- RDFWECAIRH MSMTLYHPCG TTAMGPN---

#Tc_XM_961446_{otherBeetleGMC} ---REGAVVD SELK-VFGIK KLRVADSSVF PFALAGHPTA PSVMVGEQMG DILK------ ---------- ---------- --EKYK---- ----------

#Tc_XM_961538_{otherBeetleGMC} ---KKGAVVD AKLR-VFGIK NLRVADASVF PFALAGHPNA PTVMVGEQLG DLVK------ ---------- ---------- --SDYS---- -------III

#Tc_XM_968249_{otherBeetleGMC} ---KKGAVVD SELR-VFGIK KLRVADASVF PFALAGHPNA PTVMVGEQLG DLVKRAHGVD EYLNGVSPLA IPGSTQGVGF YESSYSKGTG IPDIELMIAV

#Tc_XM_967481_{otherBeetleGMC} ---GTTAVVD NQLR-VHGIE KLRVVDAGVM PSTVSGHLNA PTVMIAEKIS DVIK------ ---------- ---------- --ATYN---- ----------

#Tc_XM_961446_{otherBeetleGMC} YNDNY----- ---------- ----YDVFHD YF-------- ---------- ---------- ---------- ---------- ---------- ----------

#Tc_XM_961538_{otherBeetleGMC} FNSAVPFAN- ---------- ----YIMISK FVVFFVRS-- ---------- ---------- ---------- ---------- ---------- ----------

#Tc_XM_968249_{otherBeetleGMC} ANATDQLTQR YFSLTDQTYE DVWKYNNIPQ TFIFHVVNLH AQSSGSVRLK SKNPFEYPVI NSNFLSDPEN RDINTLYKGI QICLKMGETK AMEAINATLQ

#Tc_XM_967481_{otherBeetleGMC} ---------- ---------- ---------- ---------- ---------- ---------- ---------- ---------- ---------- ----------

#Tc_XM_961446_{otherBeetleGMC} ---------- ---------- ---------- ---------- ---------- ---------- ---------- --

#Tc_XM_961538_{otherBeetleGMC} ---------- ---------- ---------- ---------- ---------- ---------- ---------- --

#Tc_XM_968249_{otherBeetleGMC} GGPLRACKRY QYLSKDYWYC ALRQITVNLY QPLGSCPMGK DPKKGAVVVS ELRVFGERAV GGFGQKGPWG RW

#Tc_XM_967481_{otherBeetleGMC} ---------- ---------- ---------- ---------- ---------- ---------- ---------- --

**Amino acid sequence alignment within the fungal GOX gene group**

#Anig_GOX_{Fungal_GOX} ---------- ---------- ---------- ---------- ---------- ---MQTLLVS SLVVSLAAAL PHYIRSN-GI EASLLTDPKD VS------GR

#Pama_GOX_{Fungal_GOX} ---------- ---------- ---------- ---------- ---------- ---MVSVFLS TLLLSAAAVQ AYLPAQQIDV QSSLLSDPSK VA------GK

#Aory_GOX_{Fungal_GOX} ---------- ---------- ---------- ---------- ---------- ---MKSAIFS PILFSLALAQ NYSLEKHFDV QSSLISDPKE VS------EK

#Anig_GOX_{Fungal_GOX} TVDYIIAGGG LTGLTTAARL TENPNISVLV IESGSYE--- --SDRGPIIE DLNAYGDIFG SS-VDHAYET VELA------ --------TN NQTALIRSGN

#Pama_GOX_{Fungal_GOX} TYDYIIAGGG LTGLTVAAKL TENPKIKVLV IEKGFYE--- --SNDGAIIE DPNAYGQIFG TT-VDQNYLT VPL------- --------IN NRTNNIKAGK

#Aory_GOX_{Fungal_GOX} TFDYVIAGGG LTGLTVATKL TENPDIEVLV IEKGFYE--- --SNCGSIVE DLNEYGDIFG TD-VDQAYQT VPLA------ --------VN NRTELIRSGN

#Anig_GOX_{Fungal_GOX} GLGGSTLVNG GTWTRPHKAQ VDSWETVFGN EGWNWDNVAA YSLQAERARA PNAKQIAAGH YFNASCHGVN GTVHAGPRDT GDDYSPIVKA LMSAVEDRGV

#Pama_GOX_{Fungal_GOX} GLGGSTLING DSWTRPDKVQ IDSWEKVFGM EGWNWDNMFE YMKKAEAART PTAAQLAAGH SFNATCHGTN GTVQSGARDN GQPWSPIMKA LMNTVSALGV

#Aory_GOX_{Fungal_GOX} GLGGSTLING GSWTRPDKVQ IDSWERVFGN EGWNWDSLFE YMKKAEHSRP PNEAQIAAGH SYDPACHGTN GTVQAGPRDN GKPWSPIMKA LINTASERGV

#Anig_GOX_{Fungal_GOX} PTKKDFGCGD PHGVSMFPNT LHEDQVRSDA AREWLLP--N YQRPNLQVLT GQYVGKVLLS QNGTTPRAVG VEFG------ ----THKGNT HNVYAKHEVL

#Pama_GOX_{Fungal_GOX} PVQQDFLCGH PRGVSMIMNN LDENQVRVDA ARAWLLP--N YQRSNLEILT GQMVGKVLFK QTASGPQAVG VNFG------ ----TNKAVN FDVFAKHEVL

#Aory_GOX_{Fungal_GOX} PTQQDFHCGH PRGVSMIPNA VHEDQTRSDT AREWLLP--N HERPNLKVLT GQRVGKVLLN KTESGAKATG LNFG------ ----THRKVN YNVYAKHEVL

#Anig_GOX_{Fungal_GOX} LAAGSAVSPT ILEYSGIGMK SILEPLGIDT VVDLP-VGLN LQDQTTATVR SRITSAG--- ---------- --AGQGQAAW FATFN----- ETFGDYSEKA

#Pama_GOX_{Fungal_GOX} LAAGSAISPL ILEYSGIGLK SVLDQANVTQ LLDLP-VGIN MQDQTTTTVS SRASSAG--- ---------- --AGQGQAVF FANFT----- ETFGDYAPQA

#Aory_GOX_{Fungal_GOX} LAAGSAISPL ILEWSGIGLK DVLSAAGVEQ VVDLP-VGLN MQDQTTTNVR SQAQASG--- ---------- --AGQGQAVY FASFN----- ETFGDYAHKA

#Anig_GOX_{Fungal_GOX} HELLNTK--- ---------- -----LEQWA EEAVARG--G FHNTTALLIQ YENYRDWIVN HNVAYSELFL DTAGVASFDV WDLLPFDRGY VHILDKD-PY

#Pama_GOX_{Fungal_GOX} RDLLNTK--- ---------- -----LDQWA EETVARG--G FHNVTALKVQ YENYRNWLLD EDVAFAELFM DTEGKINFDL WDLIPFTRGS VHILSSD-PY

#Aory_GOX_{Fungal_GOX} MELLNTK--- ---------- -----LDQWA EETVRNG--G FHNVTALKIQ YENYRDWLLN EDVAFAELFL DTEGKINFDL WDLIPFTRGS VHILNGD-PY

#Anig_GOX_{Fungal_GOX} LHHFAYDPQY FLN------E LDLLGQAAAT QLARNISNSG AMQTYSLGRL LPGDNLAYDA D--------- LSAWTEYIPY HFRPNYHDVG TCSMMPKEMG

#Pama_GOX_{Fungal_GOX} LWQFANDPKF FLN------E FDLLGQAAAS KLARDLTSQG AMKEYFAGET LPGYNLVQNA T--------- LSQWSDYVLQ NFRPNWHAVS SCSMMSRELG

#Aory_GOX_{Fungal_GOX} LHRYANDPKF FLN------E FDILGQAAAT KLARELSNTG EMKKYFAGEI IPGDNLAYDA S--------- LEQWADYVKE NFRANWHAVS SCSMMSREMG

#Anig_GOX_{Fungal_GOX} ------SVVD NAAR-VYGVR GLRVIDGSIP PTQMSSHVMT VFYAMALKIS DAILEDYASM Q--------- ---------- ---------- ----------

#Pama_GOX_{Fungal_GOX} ------GVVD ATAK-VYGTQ GLRVIDGSIP PTQVSSHVMT IFYGMALKVA DAILDDYAKS A--------- ---------- ---------- ----------

#Aory_GOX_{Fungal_GOX} ------GVVD SAAR-VYDVE NLRIVDGSIP PTQVSSHVMT IFYGMALKVA DAILADYSKN ---------- ---------- ---------- ----------

#Anig_GOX_{Fungal_GOX} ---------- ---------- ---------- ---------- ---------- ---------- ---------- ---------- ---------- ----------

#Pama_GOX_{Fungal_GOX} ---------- ---------- ---------- ---------- ---------- ---------- ---------- ---------- ---------- ----------

#Aory_GOX_{Fungal_GOX} ---------- ---------- ---------- ---------- ---------- ---------- ---------- ---------- ---------- ----------

#Anig_GOX_{Fungal_GOX} ---------- ---------- ---------- ---------- ---------- ---------- ---------- --

#Pama_GOX_{Fungal_GOX} ---------- ---------- ---------- ---------- ---------- ---------- ---------- --

#Aory_GOX_{Fungal_GOX} ---------- ---------- ---------- ---------- ---------- ---------- ---------- --

**Amino acid sequence alignment for all genes across different subfamilies**

#Ag_CG6142like_1_{CG6142} ---------- ---------- ---------- ---------S PIWLVLFILT TPNLLADGHD DTFFQTLFDE TSVLFNNG-- -TPRIPDTKA FR-------K

#Ag_CG6142like_2_{CG6142} ---------- ---------- ---------- --MVRRTVGT RLPVLMAFLL TASPPTDAQQ FPVFETLFDE VSLLVRTGPN ASVPIPEAKR IR-------D

#Am_CG6142like_3_{CG6142} ---------- ---------- ---------- ---MLKFLII ALLPSSVQSI IPPAILKTIY LFLFGLLKGQ DD-------- ---SIPDQTR FS-------Q

#Dm_CG6142_{CG6142} ---------- ---------- ---------- ----MRFCGV LIICLWYIQV TWSQLLVDLA RDFETSFLNN ---------- ---RIPDTTR FL-------P

#Tc_CG6142like_4_{CG6142} ---------- ---------- ---------- ------MKTI LCFTVLFLCA NSQNLLNDFR RFFGAS---- ---------- ---DVRDTAN FR-------K

#Cele_CHD_{CHD} ---------- ---------- ---------- ---------- ---------- ---------M LQNHAARSLQ R---TSKRWI NWRDSMKEGT FSADIAENKP

#Ecol_CHD_{CHD} ---------- ---------- ---------- ---------- ---------- ---------- ---------- ---------- ---------- ---------M

#Human_CHD_{CHD} ---------- ---------- ---------- ---------- ---------- ---------- -MWCLLRGLG RPGALARGAL GQQQSLGARA LASAGSESRD

#Anig_GOX_{Fungal_GOX} ---------- ---------- ---------- ---------- ---------- ---MQTLLVS SLVVSLAAAL PHYIRSN-GI EASLLTDPKD VS------GR

#Aory_GOX_{Fungal_GOX} ---------- ---------- ---------- ---------- ---------- ---MKSAIFS PILFSLALAQ NYSLEKHFDV QSSLISDPKE VS------EK

#Pama_GOX_{Fungal_GOX} ---------- ---------- ---------- ---------- ---------- ---MVSVFLS TLLLSAAAVQ AYLPAQQIDV QSSLLSDPSK VA------GK

#AgGMC_A1_{GMC-A} ---------- ---------- ---------- ---------M SELVGAA--F GSVATAASSV GWFVPMLVAA IAYFQYEEFM DPEARVIDVP TE----IMLD

#AmGMC_A1_{GMC-A} ---------- ---------- ---------- ---------- MGIESVL--T GGLTSASSGL SWFFPVLAAA LVYFEYE-VM DNEAPPINIP SE----VLLP

#DmGMC_A1_CG9503_{GMC-A} ---------- ---------- ---------- --------MS SAIVGAASAI GGAVTAATSN SWFIPMLMAA VAYFQYEEII DPESKPSDVG GD----DILD

#TcGMC_A1_{GMC-A} ---------- ---------- ---------- ---------M STITAPL--L GLGATAASNL AVFIPALAAA IAYFQYD-LL DPESRPIDVS TD----ELLE

#AgGMC_B4_iso1_{GMC-B} ---------- -----MEALM GG-------- ----QCAAQS VGPANQLFG- ---------- -LLVQTILAA QCAISPPDMW PKDYGPTALQ RG------LD

#AgGMC_B4_iso2_{GMC-B} ---------- -----MEALM GG-------- ----QCAAQS VGPANQLFG- ---------- -LLVQTILAA QCAISPPDMW PKDYGPTALQ RG------LD

#AgGMC_B4_iso3_{GMC-B} ---------- -----MEALM GG-------- ----QCAAQS VGPANQLFG- ---------- -LLVQTILAA QCAISPPDMW PKDYGPTALQ RG------LD

#AgGMC_B4_iso4_{GMC-B} ---------- -----MEALM GG-------- ----QCAAQS VGPANQLFG- ---------- -LLVQTILAA QCAISPPDMW PKDYGPTALQ RG------LD

#AmGMC_B10_{GMC-B} ---------- -----MES-- ---------- -----CMAAT CASGQS---- ----TPASTF TMLIQTIMAS YC-ASNVGKY PTDRAEEIFA STR------K

#AmGMC_B6_{GMC-B} ---------- -----MPS-- ---------- -----GMSST S--------- --------IF TLLLQAIMTS YYDLSDPRQY PADRTEEILN SN-------K

#AmGMC_B7_{GMC-B} ---------- -----MES-- ---------- -----CMSRT CSSVIAQQS- ----SPASIF TFLIQTLIAS RCKLNNPDEY PRDRVNDVLR SN-------K

#AmGMC_B8_{GMC-B} ---------- -----MES-- ---------- -----CMTTS CSPMLH--S- ----SPVCIF TLFLQTLEAS YYGLSNPNTY PRNRKQEILD SK-------I

#AmGMC_B9_{GMC-B} ---------- -----MES-- ---------- -----CARGT CSSALQ--S- ----SPASIF TMLIQTLIAS RCQLSNTNKY PTSNEEKILN SK-------M

#DmEO_B1_CG9504_{GMC-B} ------MGDQ RVHKPRTAKT RTLRVNRILL PMLLLFSGET TSVVTQLFSV DSSGLGISLM QSVAIALNAS SLALANNTAW PLQHEPPEDR LE------IE

#DmGMC_B2_CG9509_{GMC-B} ---------- -----MSLDG GQNLNLDAAA IGPDPCGSVP ATPGIGLWSG --------MV TILVQTLLSS QCLVSPASQW P------VDY VGDL----SQ

#DmGMC_B3_CG9512_{GMC-B} ---------- -----MEFLS AQ-------- -----CAARS AGPANTLMS- ---------- -LLLSTLITK YCDLSGQNQW PEDKGDWLEQ AGGF----KH

#TcGMC_B5_{GMC-B} ---------- -----MDHAI DP-------- -----CPSST SGVSAHLFL- ---------- -TLINSLLAS KCRISSPSNY PQNRASTLSD ND--------

#AgGMC_D1_{GMC-D} ---------M VVSFG----- -TLIPLLAGA ALKATPAAAG LTTAVGAAIS AATAVIGVGK LAIVPILIAS LAYYNYD-LF DPENRPFNVP E------VDR

#AmGMC_D1_{GMC-D} ---------- ---------- ---------- -------MTL ISTATLA-VK AATLLIG--K LAIIPIIIAT LAYYNYD-LM DPENQPKVTK N------LRK

#DmGMC_D1_CG9514_{GMC-D} MVVVPALGAA AVSVGGLLFK ASAASKAAAA AGVAAAGASK LGLAIAGAIK LATAVIGVGK LTILPFLIAA IAYYNYD-LF DPENRPFNVQ Q------VDL

#TcGMC_D1_{GMC-D} ---------- ---------- ---------- -------MAI ILPTVLASIK AGVGIIGAGK IAILPFLLAA LAYFHYD-QF DPENRPVDRK V------VDK

#AgGMC_E1_{GMC-E} ---------- ---------- ---------- --------MA LGLASVAAVA GGLAHTPIAL LTLIPLLAVG VNYYRYQ-SV DPETNPTDQQ T------LRR

#AmGMC_E1_{GMC-E} ---------- ---------- ---------- --------MA IGLTTLFSAT -SILG----- FTLIPLVAIG LTIYKYN-QE DPESHLFDTK Q------LLR

#DmGMC_E1_CG9517_{GMC-E} ---------- ---------- ---------- --------MA FG-------- -----TMTSL LGMIPLLAIG MNFYRYQ-SV DPENKVQEPT V------IRR

#TcGMC_E1_{GMC-E} ---------- ---------- ---------- ------MLAT IGQVAHFAG- -SLTGNPLAI IGLVPIFAAG LAFMRYV-SI DPEAHPVNVR H------VRP

#AgGMC_G2_{GMC-G} ---------- ---------- ---------- ---------- MNAVGSYEVR TRLLYTSRIG TVFLLLIDAS IWLQRPD-IV DFHHRVQPIP GP----FVQD

#AgGMC_G3_{GMC-G} ---------- ---------- ---------- --------MG FGPLESQYTR MRIMLTRPTS ALILLILDAC IWLQRTD-VV DYRNRVQDIP SQ----FIYD

#AmGMC_G1_{GMC-G} ---------- ---------- ---------- ---------- ---------- ----MSYGPE LSFLVLLRIL IGMHRSD-II DHESRVR--P TL----APQT

#DmGMC_G1_CG12398_{GMC-G} ---------- ---------- ---------- ---------- MSAINTFVAM WRFLLTLGPS AMIILLLNKG ILEQRPD-IV DEQHRVRSIH IE----DLRE

#TcGMC_G1_{GMC-G} ---------- ---------- ---------- --------MA SHAQMNLFSI TRMALTLGPG LGFLLYLHSS TMTHRPD-IL DREHRVHDVP MY----QILP

#AgGMC_I3_{GMC-I} ---------- ---------- -------MGV LQDLLRVHDG -NGRLLFLVF LCLYLTVRCS VCQCPDTGGL GAEDPANVRL LQENSIKQAS L-------LK

#AgGMC_I4_{GMC-I} ---------- ---------- ----MKHLWI AVILIATHSA LTANGFFLLL KTLAHAGRYI NEHYPDEG-- ---------- -----INYRQ S-------VP

#DmGMC_I1_CG9522_{GMC-I} ---------- ---------- ---------- ------MEKL LLTQLLFLLL IPLIHSQRS- ----SQLDEL RRLGLGN-VV NVPFFSDVPQ ---------R

#DmGMC_I2_CG12539_{GMC-I} ---------- ---------- ---------- ------MQST QISQLFLLLH LLIFTTVARG DVNRLVLDQL NQVGLVN-LI EQATRPNVPR D-------LS

#TcGMC_I5_{GMC-I} ---------- ---------- ---------- ----MLKKFL ILTALLSAIS PTSAENVDEF LAKVKKNYDN AKRSKRFIDP YEYPGAEQPL ------DEMS

#TcGMC_I6_{GMC-I} ---------- ---------- ---------- --MFHSLIWA SFLAIFFVLR PVSSN----- YDDININTIT ERFGTIFGTT DPIN--IIPN -----HKIDE

#TcGMC_I7_{GMC-I} ---------- ---------- ---------- ----MKLQTV FLLTLLFLVK SDDSENLQYI YDELSAVYGQ YSNKTFFLTP DQWAEWTLPK DPGKRESPKS

#AgGMC_K1_{GMC-K} ---------- ---------- ---------- --------MA AQRTSASTVV PTDAATQESY R--------- ---------- ---------- ---------T

#TcGMC_K2_{GMC-K} ---------- ---------M LTLATKLLLL G--VVSTVLS SPFKLTEQYI EEFKTGIESL KKLAH----- -EHKFAEKNF DFNLHNGSEP -----VPDDI

#TcGMC_K3_{GMC-K} ---------- ---------M LRSCVLFLIF SCAFASHYDT WPFTLADKYI EEFKTNVEEF KKFAHTYQHH EEIKYEVEEQ DLTEATKSEL HAPALITDAA

#AmGMC_L1_{GMC-L} ---------- ---------- ---------- ---------- ---------- ---------M SWIPPDLATL CQPHSTVSTC QPPAFMFLAL IAH-----LE

#TcGMC_L1_{GMC-L} ---------- ---------- ---------- ---------- ---------- ---------M SWIPPNIAES CAVYTNLTSC QPPTDEYHQP RG-------R

#AmGMC_M1_{GMC-M} ---------- ---------- ---------- -----MIGSD KIAHLTLLVI YTTFLAEIRT ISLFHSYK-- --------LP NDILNRDEGD N--------R

#AgGMC_Q3_{GMC-Q} ---------- ---------- ---------- ----MAQLP- ---------- ---------- -----GVQSI IQ---FYRDG DERLKYEKPD Q----RPLLP

#AgGMC_Q4_{GMC-Q} ---------- ---------- ---------- ----MQYLPL ---------- AAGILGMVSF SRPQDSLLSM LS---FLQDG GERMSHELPS Q----PVVRP

#AmGMC_Q7_{GMC-Q} ---------- ---------- -------MFA TYKSSHNTHR LFFLLFLTAA LPNLSKSITH TGTENLITNV ISWNKFLNES LKFASRTQPD RT---PESNS

#DmGMC_Q1_CG9519_{GMC-Q} ---------- ---------- ---------- ----MMTSPR NHIGTLLVVW LLEFSMVISQ TDTGNALMDM LE---IYRRG QAQLDLENLD EG---QVITT

#DmGMC_Q2_CG9521_{GMC-Q} ---------- ---------- ---------- ----MASKSL IFVG-LCLAY MATWSQISAQ NNN-NVLFET IN---FLRRG QADVELENYD NN---VILDS

#TcGMC_Q5_{GMC-Q} ---------- ---------- ---------- ----MLKTV- ---------- --ILLTIIAN TYQQSVLEGI IN---ILEEG EAQFNLE-PE DV---RNLLP

#TcGMC_Q6_{GMC-Q} ---------- ---------- ---------- ----MLKPVP ---------- --FFFSLFAL TSPQSLLDGL IN---FIEEG DAQSFNE-PP DT---PVLLP

#AgGMC_Z1_{GMC-Z} ---------- ---------- ---------- --------MV FNVLIASSVI KTAT-VVGSS LWLIPFLLGA ISYYRYD-RV DPESRVINQE A------LLP

#AmGMC_Z1_{GMC-Z} ---------- ---------- ---------- --------MV LSAIVVASAL KGALSLIGTS LWLIPLLIAG LSYYRYD-QL DPESRPIDRY P------LYP

#DmGMC_Z1_CG9518_{GMC-Z} ---------- ---------- ---------- --------MV LNLLFITTVI KSTFGVVTTG LWLIPLMLAA ITYYRYD-AV DPESRPLDQL N------LYP

#TcGMC_Z1_{GMC-Z} ---------- ---------- ---------- --------MV VETLFISTAL K-TIGIVGST LWIIPLIFAG ISYYHYD-KL DPESPIINRR T------LYK

#AgGLD_{Insect_GLD-GOX} ---------- ---------- ---------- --------MS SCACPMTSPV GATLAALCGG TQYMLFMGLL EVFIRSQCDL EDPCGRTKAK SSR---NVDY

#AmGLD_{Insect_GLD-GOX} ---------- ---------- ---------- ---------M SCNCPLNPST GPTLASTCGG SSFMLFMGLL EVFLRSQCDL EDPCNRPLPP PT-----VNS

#AmGLXr_1_{Insect_GLD-GOX} ---------- ---------- ---------- ---------- ---------- ---MASTCG- -VKTSFMSLV EKVIASTCDI ANPCNR---- -------LEH

#AmGLXr_2_{Insect_GLD-GOX} ---------- ---------- ---------- ---MSYNLSI SPICPDPNLG -PSLAQVCPG PQFLTFMSLF NTFALAKEEV SLLCQRFEPV EP-----AEY

#AmGOX_{Insect_GLD-GOX} ---------- ---------- --------MA ILNSMYNNVS PLQCTSPFLG GPQLTDVCSA SNGELFLALL NFFVATSPVI GEPCQRVHSS RI-----PDL

#DmGLD_{Insect_GLD-GOX} ---------- ---------- ---------- ----MSASAS ACDCLVGVPT GPTLASTCGG SAFMLFMGLL EVFIRSQCDL EDPCGRASSR FRS---EPDY

#TcGLD_{Insect_GLD-GOX} ---------- ---------- ---------- ---------M ACNCPVTQ-P GPTLASTCGG AQYMLFMGLL EVFLRSQCDL EDPCGRPHNT P------VLP

#TcGLXr_3_{Insect_GLD-GOX} ---------- ---------- ---------- ---------M ECGC-AAPYI GPSLANTCGG GAFVLFMSLL DTFIRNKCDL SEICQRVVPK TQ-----PDI

#TcGLXr_4_{Insect_GLD-GOX} ---------- ---------- ---------- ---------M SCCA-NEPYI GPPLDRTCFG GSYIVFMHLL NTLITQQCDV SEICQRINPQ LQ-----PDS

#DmNinaG_CG6728_{NinaG} ---------- ---------- ---------- ---------- ---------M GMKFQKILVL AGIVIGFLSI IVVLAGTLLK NSVPNVLAPV ER------HF

#Tc_NinaG_like_{NinaG} ---------- ---------- ---------- ---------- -------MCC SNPAFYISVY VLAVNLFGLY LRFVYFHNYF ECFACRELDF KD-------Q

#Tc_XM_961446_{otherBeetleGMC} ---------- ---------- ---------- -------MSG KLLLVVLLTF SANSHAYYYQ D-KIEYYVQL ITGAFRNALT TVLPTDSYQY FSGEVRRSYG

#Tc_XM_961538_{otherBeetleGMC} ---------- ---------- ---------- -------MNL KILRVVLPVM FVYAQGYLSE Q-KIDYFVRL ISKANENAMT YQLPKNAYEY YTKDRQQKFG

#Tc_XM_967481_{otherBeetleGMC} ---------- ---------- ---------- ---------- --MWATLSVF IAILPKIITS DPAIDFYKKV IDENVAKVES YKLPD----- ---------G

#Tc_XM_968249_{otherBeetleGMC} ---------- ---------- ---------- -------MN- KDLSVCILLF CAVLQAHTDN Q-TVQYYVDL ISEAYENGLT YVFPTSAYEY YSDTIPKKYG

#Ag_CG6142like_1_{CG6142} EYDFIIIGAG SGGSVMANRL SEVRDWNVLL LEAGKEG--- -----NMLTE VPLTAGLTTI TG-YNWGYKA DP-------- -MKGACLGLK GGVCNWPKGR

#Ag_CG6142like_2_{CG6142} EYDFVVIGAG SGGSVMANRL SEVRDWSVLL LEVGKEE--- -----NLISN VPLTAGLTTA TG-YSWGYRS DP-------- -MRNACRGLE QGVCYWPKGR

#Am_CG6142like_3_{CG6142} EYDFIVIGAG SAGSVLTNRL TENPQWNVLL LEEGKDE--- -----IFLTD IPLLAPALHV TD-YVRLHTS EPRPRNTD-- GTDGYCLSMK NGRCNLPGGR

#Dm_CG6142_{CG6142} EYDFIIVGAG SAGCVMANRL SEISSASVLL LEAGDQE--- -----TFISD VPLTAALTQM TR-YNWGYKA EP-------- -TEHACQGLK GGVCNWPKGR

#Tc_CG6142like_4_{CG6142} SYDFVVVGAG SGGCVVANRL SENPEWSVLL LEAGDDE--- -----NFLTD VPLIASLQTI TS-YNWGYKS ER-------- -LATACLGLI DGRCNMPRGK

#Cele_CHD_{CHD} --THIIVGAG SAGCVLANRL TEDPSNRVLL IEAGPVD--- --HKWDWRIH MPAALMYNLC SDTYNWHYHT TAQK------ -------NLG NRVFYWPRGR

#Ecol_CHD_{CHD} QFDYIIIGAG SAGNVLATRL TEDPNTSVLL LEAGGPD--- --YRFDFRTQ MPAALAFPLQ GKRYNWAYET EPEP------ -------FMN NRRMECGRGK

#Human_CHD_{CHD} EYSYVVVGAG SAGCVLAGRL TEDPAERVLL LEAGPKDVLA GSKRLSWKIH MPAALVANLC DDRYNWCYHT EVQR------ -------GLD GRVLYWPRGR

#Anig_GOX_{Fungal_GOX} TVDYIIAGGG LTGLTTAARL TENPNISVLV IESGSYE--- --SDRGPIIE DLNAYGDIFG SS-VDHAYET VELA------ --------TN NQTALIRSGN

#Aory_GOX_{Fungal_GOX} TFDYVIAGGG LTGLTVATKL TENPDIEVLV IEKGFYE--- --SNCGSIVE DLNEYGDIFG TD-VDQAYQT VPLA------ --------VN NRTELIRSGN

#Pama_GOX_{Fungal_GOX} TYDYIIAGGG LTGLTVAAKL TENPKIKVLV IEKGFYE--- --SNDGAIIE DPNAYGQIFG TT-VDQNYLT VPL------- --------IN NRTNNIKAGK

#AgGMC_A1_{GMC-A} KYDFIIIGAG SAGAVLANRL TEVENWNVLL LEAGGDE--- -----TEISE VPLMAGYLQL SK-LDWKYKS EPSG------ ---TFCLAMN GGRCNWPRGK

#AmGMC_A1_{GMC-A} AYDFIVVGGG SAGAVVASRL SEIENWNVLL LEAGSDE--- -----TEISD IPLLAGYLQL SQ-LDWQYKT EPDG------ ---QSCLAMS NGRCNWPRGK

#DmGMC_A1_CG9503_{GMC-A} HYDFIVIGAG SAGAVVANRL TEVENWNVLL LEAGGDE--- -----TELTD VPLMAGYLQL SK-IDWQYKT EPSG------ ---TSCLAMQ GGRCNWPRGK

#TcGMC_A1_{GMC-A} RYDFIVVGAG SAGAVVANRL SEIEQWNVLL LEAGGDE--- -----MEISD VPLMAAYLQL SQ-IDWKYKS EPQG------ ---QACLAMK NGRCNWPRGK

#AgGMC_B4_iso1_{GMC-B} EYDFVIVGAG SAGSVVANRL SENPDWKVLL LEAGGDP--- -----PIESE IPFMQIHLAK SS-VDWVYYA DSRDKLNPHN RTACRASTSP AG-CFWPRGK

#AgGMC_B4_iso2_{GMC-B} EYDFVIVGAG SAGSVVANRL SENPDWKVLL LEAGGDP--- -----PIESE IASMAMALQH SD-VDWAYNV Q--------- RSDSSSLGTR NG-TFWPRGR

#AgGMC_B4_iso3_{GMC-B} EYDFVIVGAG SAGSVVANRL SENPDWKVLL LEAGGDP--- -----PIESE IPETFFTIQK TD-ADWENYV E--------- PTPHASKGSK DG-AFWPRGR

#AgGMC_B4_iso4_{GMC-B} EYDFVIVGAG SAGSVVANRL SENPDWKVLL LEAGGDP--- -----PIESE VPYLAFALLN GS-HVWNYYA E--------- RSDTASKGYK RG-SYWPRGK

#AmGMC_B10_{GMC-B} EFDFIIVGSG SAGSILASRL TEVNDWDVLL IERGEDP--- -----LPETS SPALFFDNID GP-QNYHYLT E--------- YQNTSCLGTV HQRCKWASGK

#AmGMC_B6_{GMC-B} EFDFVIVGGG TAGSVLAHRL TEVMDWDVLL VERGEDP--- -----LPETE VPALVFNNFG SS-QDYRYAT E--------- YQEGACMSMK GKRCKWSKGK

#AmGMC_B7_{GMC-B} EFDFVIIGGG TAGSILARRL TEVKNWNVLL IERGGYP--- -----LPETA VPALFTSNLG FP-QDYAYKI E--------- YQKEACLSQV DKRCRWSKGK

#AmGMC_B8_{GMC-B} EFDFVIVGGG SAGSVLARRL TEVEDWKVLL VERGGYP--- -----LPETE IPGFFANNLG LK-QDYAYKV E--------- NQEEACLSQV DKRCRWSKGK

#AmGMC_B9_{GMC-B} EFDFVIVGGG SAGSVLARRL TEVEDWNVLL IERGVDP--- -----LPETI PPGLYNNNLG GP-QDYYYTL E--------- PQESSCLSNK DKRCIWSRGK

#DmEO_B1_CG9504_{GMC-B} SYDYIVVGAG SAGSIVASRL SELCQVKVLL LEEGQLP--- -----PLESE IFGLTGALHH DE-RYMFLEE AVP------N PKCCQAMASM HG-CVWWHGR

#DmGMC_B2_CG9509_{GMC-B} PYDFVVIGAG SAGSVVASRL SENPDWRVLV LEAGGDP--- -----PVESE LPALFFGLQH TN-FTWNYFT E--------- PSDEACQAMK DGRCYWPRGK

#DmGMC_B3_CG9512_{GMC-B} DYDFIVIGSG TSGAVVAGRL AEVKNWKVLL LEAGGDP--- -----PIETE FVAWHMATQF SE-WDWQYHS K--------- PNGRACMAMK GESCHWPRGK

#TcGMC_B5_{GMC-B} EFDFIIVGAG SSGSVVANQL SLNRNWKVLV LESGNLP--- -----PPDSE IPSLLFSLQG TE-SDWQYAT E--------- PNQKSCQGFI EKKCRWPRGK

#AgGMC_D1_{GMC-D} EYDFIVVGAG SAGAVVASRL SEIGGWKVLL LEAGGHE--- -----TEISD VPILSLYLHK SK-LDWKYRT QPQK------ ---TACQAMK DNRCCWTRGK

#AmGMC_D1_{GMC-D} EYDFVVVGGG SAGSVVVNRL TENPGWSVLL LEAGGHE--- -----TEITD VPILSLYLHK SK-LDWKYRT QPQD------ ---SACQAMV DRRCCWTRGK

#DmGMC_D1_CG9514_{GMC-D} AYDFIIIGGG SAGTVLASRL SEIPHWKILL LEAGGHE--- -----TEISD VPLLSLYLHK SK-MDWKYRT QPQP------ ---TACQAMK DKRCCWTRGK

#TcGMC_D1_{GMC-D} EYDFVVVGGG SAGSVIANRL TEIPSWKVLL LEAGGHE--- -----TEISD VPVLSLYLHK SK-LDWGYKT EPQT------ ---EACKAMI ENRSSWTRGK

#AgGMC_E1_{GMC-E} YYDFVVIGAG SAGAVVASRL SEIGDWSVLL LEAGGDE--- -----NEVTD VPSLAGYLQL TE-YDWKYQT TPSAD----- --RRYCQAMI GDRCNWPRGK

#AmGMC_E1_{GMC-E} MYDFIVVGGG SAGAVVASRL SEVSNWTVLL LEAGGDE--- -----TEISD VPLLSGYMQL TD-MDWKYQT SPPTT----- --SAYCLAMI GDRCNWPRGK

#DmGMC_E1_CG9517_{GMC-E} QYDFVVIGGG SAGAVVANRL SEVRNWTVLL LEAGGDE--- -----TEISD VPALAGYLQL TE-LDWKYQT TPSST----- --RQYCQAMK GDRCFWPRGK

#TcGMC_E1_{GMC-E} EYDFIVVGGG SAGAVVASRL SEIANWTVLL LEAGGDE--- -----NEISD IPALSGYTQM SQ-FDWMYQT SPPGD----- --SPYCLAMI GDRCNWPRGK

#AgGMC_G2_{GMC-G} IYDFVVVGAG SAGAVMAARL SEICHWDVLL LEAGTDE--- -----SFLTD IPFLYPTLQT SR-VDWKFRT EPSD------ ---RFCLAMK DQRCRWPRGK

#AgGMC_G3_{GMC-G} VYDFVVIGGG SAGAAAAARL SEVCDWNVLL LEAGTDE--- -----SFLSD LPYLYPALQK GP-LDWQFET EPNE------ ---RFCQGMR GNRCSWPRGK

#AmGMC_G1_{GMC-G} SYDFVIIGGG SAGSVLANRL SENSNWTVLL LEAGADE--- -----PDFSD VPSIFPVLQL TP-VDWQFKT EPSD------ ---NYCKAMR GHECNWPRGK

#DmGMC_G1_CG12398_{GMC-G} SYDFIVIGGG SAGCVLAARL SENPEWSVLL LEAGGDE--- -----PLLID LPQLYPVFQR SP-WDWKYLT EPSD------ ---RYCLAME DQRCFWPRAK

#TcGMC_G1_{GMC-G} SYDFIIVGGG SAGAVLANRL SENPEWKVLL LEAGPDE--- -----ISLTD LPLLFPTLQL SP-FDWQFKT QPGE------ ---KYCQAMT RGQCNWPRGK

#AgGMC_I3_{GMC-I} KYDFIIVGAS PSGCLLANRL TEIRDWNVLL IEAGEQE--- -----NLFVQ VPIFSAYLQS TS-YNWGYLA EP-------- -QNYSCWGMK DQRCSYPRGK

#AgGMC_I4_{GMC-I} EYDFIIVGAG AAGCVLANRL SENPQWKILL LEAGPGE--- -----NDLQN IPLLTTFLQN SQ-YNWADIA EA-------- -QNTSCYGMI DQRCSLPHGK

#DmGMC_I1_CG9522_{GMC-I} NYDFIIVGGG AAGCTLAARL SENPNWSVFL IEAGGVE--- -----NIVHQ VPLLAAHLQS TA-SNWGYNS TP-------- -QRHACRGMP DNKCALPRGK

#DmGMC_I2_CG12539_{GMC-I} NYDFIVIGAG AAGCTLAARL SENPQVSVAL IEAGGVE--- -----NIAHL TPVVAGYLQQ TS-SNWGYKS VP-------- -QKLSCHGMN NNECALPRGK

#TcGMC_I5_{GMC-I} KYDFIVVGSG SSGSVIANRL TET-NWTVLL LEVGEEA--- -----TPLTD IPVIAPLFQF TS-LNWNYLM EK-------- -QDNMCLGLE DQRMAWPRGR

#TcGMC_I6_{GMC-I} VYDFIIIGSG SSGSVVASRL SEIPTWKILL LEAGNAA--- -----NILTK VPIMAPLFQL TP-YNWNYTM EP-------- -EPNVCQAME EETCAWPRGK

#TcGMC_I7_{GMC-I} GYDFIVVGSG SSGAVIANRL SENPNWEVLL LEAGKGE--- -----NFFSQ IPLVCPTLAF TH-YNWDFIA EY-------- -QPNVSFGFE NNRMRWPRGR

#AgGMC_K1_{GMC-K} VYDFIVVGGG TAGSVIASRL AELQQWHILL IEAGGG---- ---------- -------PSD KD-LSWNLQA QRQM------ ---GSCLGAP EQRCEIPTGR

#TcGMC_K2_{GMC-K} EYDFIIIGAG ASGSVIANRL TERPEWKVLL LEAGGPE--- -----TPYTR IPRLGHLLQN SD-YNWAYTT TPQK------ ---NWCKGMI DGSCAIAGGK

#TcGMC_K3_{GMC-K} HYDFIIVGGG TSGAILASRL SEIPEWKILL LEAGAPE--- -----TIATK VPKNWELLKN TP-YNWGYVT TPQN------ ---YSCLGMV DHKCVIPTGR

#AmGMC_L1_{GMC-L} EYDFIIVGAG SAGCVLANRL SEVKHWKILL LEAGIEE--- -----PLVAD VPAFASMLQA SN-IDWMYRT QPER------ ---HSCRSRR DRSCAWARGK

#TcGMC_L1_{GMC-L} EYDFIVLGAG SAGCVLANRL TEIPSWSVLL LEAGDEE--- -----PEVAD VPAFAPVLQQ SS-IDWGFST QPDP------ ---NSCLARQ NGQCSWARGK

#AmGMC_M1_{GMC-M} RYDFIIVGAG SGGSVLANRL SENKEWNILL LEAGNTE--- -----NLFMQ VPSFSVFMQL SR-FNWGYKV EP-------- -QENACLSMI NRQCDWPRGK

#AgGMC_Q3_{GMC-Q} EYDFIIVGGG SAGCVLANRL TEISHWSVLL IEAGPRE--- -----NLLMD IPIFAHYLQG LS-INWDYRT KS-------- -SDQCCLAFK NNQCRLPRGK

#AgGMC_Q4_{GMC-Q} EYDFIIVGAG SAGSVLANRL SEVPDWSVLL IEAGPGE--- -----NLLMD IPMAAHYLQN FN-INWDYRT KP-------- -SDQYCLAFK NNQCRFPRGK

#AmGMC_Q7_{GMC-Q} RYDFIVIGAG TAGATVASRL TEIQNLTVLL IETGLEE--- -----ELYMD IPLFANFLQR IPGLDWMYQT ES-------- -SDNYCRGMI GRKCRFPQGK

#DmGMC_Q1_CG9519_{GMC-Q} KYDFIVVGAG TAGCALAARL SENPRWRVLL LEAGGPE--- -----NYAMD IPIVAHLLQL GE-INWKYKT EP-------- -SNSYCLAMN NNRCNWPRGK

#DmGMC_Q2_CG9521_{GMC-Q} EYDFIVVGAG TAGCALAARL SENPQWRVLL LEAGGPE--- -----RLVMD VPIVAHFLQL GE-MNWKYRT QP-------- -SDHACLAMN NNRCNWPRGK

#TcGMC_Q5_{GMC-Q} EYDFIVVGAG SAGCVVANRL SENPNWKVLL IEAGRTE--- -----NYLMD MPILANYLQF TD-SNWKYKT TP-------- -SGRFCMGMD NQQCKWPRGK

#TcGMC_Q6_{GMC-Q} SYDFIIVGAG TAGCVLANRL SENPSWNVLL LEAGRPE--- -----NYLMD LPVLANYIQF TD-ANWRYKT EP-------- -SDKFCLGME NQQCNWPRGK

#AgGMC_Z1_{GMC-Z} EYDFIVVGGG SAGAVVANRL TEIHRWKVLL LEAGPDE--- -----NEISD VPSLAAYLQL SK-LDWAYKT EPTN------ ---KACLGMV NNRCNWPRGK

#AmGMC_Z1_{GMC-Z} EYDFIVVGGG SAGAVVANRL SEIPKWNVLL LEAGPDE--- -----NEVTD VPSLAAYLQL TK-IDWKYKT EPTG------ ---RACLAMK DGRCNWPRGK

#DmGMC_Z1_CG9518_{GMC-Z} EYDFIVVGSG SAGAVVANRL SEVRKWKVLL IEAGPDE--- -----NEISD VPSLAAYLQL SK-LDWAYKT EPST------ ---KACLGMQ NNRCNWPRGR

#TcGMC_Z1_{GMC-Z} EYDFVVVGGG SAGAVVASRL SEIPSWNVLL LEAGPDE--- -----NEISD VPSLAAYLQL SK-LDWTYKT EPTG------ ---RACLGMN NGRCNWPRGK

#AgGLD_{Insect_GLD-GOX} EYDFIVVGGG SGGSVIASRL SEIKNWKVLL IEAGPDE--- -----PTGAQ IPSMFLNYLG -SDIDWKFNT E--------- PEQYACLGSP EQRCYWPRGK

#AmGLD_{Insect_GLD-GOX} RYDFVVIGGG SAGATVASRL SEEPRFSVLL LEAGLDE--- -----PTGTQ IPSFFFNFIG -TDIDWQYNT E--------- SEDTACLNKD DRKCYWPRGK

#AmGLXr_1_{Insect_GLD-GOX} RFDFIVVGAG VAGPVIARRL SDNPWWRVLL IEAGPEE--- -----PSMTS IPGLAVHAVN -STLDWRFKT EPTE------ PHPTACLEN- DGVCSWPRGK

#AmGLXr_2_{Insect_GLD-GOX} YYDFIVVGGG TAGSVVASRL SEQREWKVLL LEAGPDE--- -----PPGTD VPSMVAMFLG -SDIDWGYRT T--------- NEKNACLSS- GGSCFWPRGK

#AmGOX_{Insect_GLD-GOX} SYDFIVVGGG AARAVVAGRL SEVSNWKVLL LEAGPDE--- -----PAGAE IPSNLQLYLG -GDLDWKYYT T--------- NESHACLST- GGSCYWPRGK

#DmGLD_{Insect_GLD-GOX} EYDFIVIGGG SAGSVVASRL SEVPQWKVLL IEAGGDE--- -----PVGAQ IPSMFLNFIG -SDIDYRYNT E--------- PEPMACLSSM EQRCYWPRGK

#TcGLD_{Insect_GLD-GOX} EYDFIIVGGG SSGAVVASRL SEIPEWNVLL IEAGLDE--- -----PTGTQ VPSMFLNFIG -SEIDWGYQT E--------- PEPSACLAET EQRCYWPRGK

#TcGLXr_3_{Insect_GLD-GOX} EYDFVVIGGG SGGATAAGRL SEVPEWKVLL IEAGGDE--- -----PPGSQ VPSMVISYHG DPHMDWNYKT E--------- PEQQACLGFP EKRCSWPRGK

#TcGLXr_4_{Insect_GLD-GOX} EYDFVVIGGG AGGSVVAGRL SENPNWKILL IEAGGDE--- -----PPGSQ VPSMMNNYLG DSQMDWRYRT E--------- PQEMACLGRP GRRCDWPRGR

#DmNinaG_CG6728_{NinaG} AFDYVIVGGG TGGSTLTSLL AKNSNGSVLL IEAGGQFG-- ------LLSR IPLLTTFQQK GI-NDWSFLS VPQK------ ---HSSRGLI ERRQCLPRGK

#Tc_NinaG_like_{NinaG} AYDYIVVGSG SAGSIVARRL AENPSVKVLL IEAGASG--- -----NGILQ IPTVSLMLQD SV-FDWQYRT VPQK------ ---HACLGLD KKVSHWPMGK

#Tc_XM_961446_{otherBeetleGMC} SFDFVVIGAG AAGAVIANRL TEVEDWNVLV LEAGGYG--- -----NDFSD IPDMYWPIEF TD-FNWGYNS TPQR------ ---TACLGLI DQECFYPRGR

#Tc_XM_961538_{otherBeetleGMC} TFDFVVIGAG AGGTVVANRL SEVANWNILV LEAGGYG--- -----NDFSD IPNMYFPIQF SH-FNWGYNS TPQT------ ---TACLGLE NHVCLYPRGK

#Tc_XM_967481_{otherBeetleGMC} NYDFIIIGAG SAGSVLATRL SENENWKILL LEAGGEE--- -----NDFST IPSMWANLQM SE-INWGYRT ISQK------ ---NCCLGMK NRQCLEPRGK

#Tc_XM_968249_{otherBeetleGMC} TFDFVVIGSG AAGSVAASRL SEINKWSVLV LEAGTFW--- -----NNFSD IPNMYEPIAF TH-FNWEFNS TPQT------ ---TACLGLV NQICNYFFFK

#Ag_CG6142like_1_{CG6142} GLGGTSLINF LIYTRGHRSD YDGWEQ-AGN PGWGYREVLQ YFKKSERVQI PELR-HS--- ----PYRSTA GLVDVEESQF ETPLLKRFIE AG-RDLGYM-

#Ag_CG6142like_2_{CG6142} GLGGTSLINF LLYGRGHQRD YDDWER-AGN YGWGYRDVRR YFEKAEQIKG ------Q--- ----PYN-PH GYLHIEESSF ETPMLGRYIE AG-KRFGYR-

#Am_CG6142like_3_{CG6142} AVGGSSVVNF MIYSRGSPND YDNWAA-QGN PGWSYQNVLP YFIKSENCKL LD-Q-DI--- ----RFHGKG GYLDVISSPY VSPLRECFLR GG-EELGYD-

#Dm_CG6142_{CG6142} GVGGTSLINF MLYTRGHRRD YDEWAA-ANN SGWSYDELLP YFRKSERIGI PELY-KS--- ----PYHGRN GQLDVQYTDY RSQLLKAFLK SG-REMGYE-

#Tc_CG6142like_4_{CG6142} ALGGTSVINF LLYTRGTKQD FDQWAE-LGN PGWGYDQVLP YFIKSENCTK CREI-DG--- ----KYHGKS GYLSVEHPGY ESPLVKRFIK SG-EELGYK-

#Cele_CHD_{CHD} VWGGSSTLNA MCYVRGHAYD YNRWEKE-GA SGWNYANCLP YFKKAETYSD ATGPNDP--- -----YRGNN GPLYVKKGD- AENPLHKAWL NVGKEHPLG-

#Ecol_CHD_{CHD} GLGGSSLING MCYIRGNALD LDNWAQEPGL ENWSYLDCLP YYRKAETRDM GEND------ -----YHGGD GPVSVTTSKP GVNPLFEAMI EAGVQAGYP-

#Human_CHD_{CHD} VWGGSSSLNA MVYVRGHAED YERWQRQ-GA RGWDYAHCLP YFRKAQGHEL GASR------ -----YRGAD GPLRVSRGK- TNHPLHCAFL EATQQAGYP-

#Anig_GOX_{Fungal_GOX} GLGGSTLVNG GTWTRPHKAQ VDSWETVFGN EGWNWDNVAA YSLQAERARA PNAKQIAAGH YFNASCHGVN GTVHAGPRDT GDDYSPIVKA LMSAVEDRGV

#Aory_GOX_{Fungal_GOX} GLGGSTLING GSWTRPDKVQ IDSWERVFGN EGWNWDSLFE YMKKAEHSRP PNEAQIAAGH SYDPACHGTN GTVQAGPRDN GKPWSPIMKA LINTASERGV

#Pama_GOX_{Fungal_GOX} GLGGSTLING DSWTRPDKVQ IDSWEKVFGM EGWNWDNMFE YMKKAEAART PTAAQLAAGH SFNATCHGTN GTVQSGARDN GQPWSPIMKA LMNTVSALGV

#AgGMC_A1_{GMC-A} VLGGSSVLNY MLYLRGNKKD YDNWEA-MGN TGWGYKDALY YFKKSEDNTN PYLANT---- ----PYHSTG GYLTVGEAPY HTPLAAAFVE AG-VEMGYE-

#AmGMC_A1_{GMC-A} VIGGSSVLNY MLYLRGNKKD YDIWES-QGN RGWSFKDVLY YFKKSEDNQN PYLTKT---- ----PYHATG GYLTVQEAPW HTPLATAFIQ AG-QEMGYE-

#DmGMC_A1_CG9503_{GMC-A} VLGGSSVLNY MLYLRGSKHD YDNWEA-MGN PSWSYRDALY YFKKSEDNTN QYLANT---- ----PYHATG GYLTVGEAPY HTPLAASFVE AG-VEMGYE-

#TcGMC_A1_{GMC-A} VIGGSSVLNY MLYLRGNKKD YDIWES-LGN PGWGSQDALY YFKKSEDNQN PYLSRT---- ----PYHATG GYLTVSEAPY HTPLVAAFVE GG-RQLGYA-

#AgGMC_B4_iso1_{GMC-B} MLGGSGAMNA MVYIRGNARD YDAWEF-EGN SGWGWRDVLP YFRKSENNHD AAVVG--D-- ---GTYHGTG GYLSVSSASG -HSGHMEHLI AAVQESGYDY

#AgGMC_B4_iso2_{GMC-B} TLGGSGAINA MMYVRGNRRD YDRWQS-LGN PEWGWEDVLP YFRKSENMNN PRLVRGEG-- ---AKYHRTG GYLNVEQRID -NTTLNGILR RGALELGYEW

#AgGMC_B4_iso3_{GMC-B} TLGGCGAINA MLYVRGNSRD YDGWAE-LGN PNWEWSDVLP YFKKSEDNHD SELLRRDG-- ---GKYHAAG GYLKVGNFPV -NHPLAEVML QAFKDAGFES

#AgGMC_B4_iso4_{GMC-B} MLGGSSSNNI MLYVRGNSRD YDRWEE-QGN PGWGWKDVLE YFKKSEDNGA QHLLQER--- ---ADYHAQG GLLKVNSFMS -NDMTKLVIT EAAQELGIPE

#AmGMC_B10_{GMC-B} ALGGSSVTNG MLYVIGNEKD YNDWEE-SGN DGWGFASVLP YFAKSTN-CS ASYVSRYG-- ---TKYCGDN GPVRIGHFEA ASPGVQKILM DGVREAGHDV

#AmGMC_B6_{GMC-B} ALGGSSVINA MLHVFGNRMD YDDWAS-EGN EGWGYEQVLP YFRKSLS-CS PDHVARFG-- ---SDYCGTS GPMRIRNYNY TATDIQDVML DAARELGYEI

#AmGMC_B7_{GMC-B} ALGGSSVINA MLHIFGNKRD YDTWEN-IGN PGWNYEQVLP YFRKSLS-CA PEFIAKYG-- ---TDYCGTD GPMRIRHYNY TATDAEDIIL EAAHEAGYDV

#AmGMC_B8_{GMC-B} ALGGSSVINA MFYIFGNKRD FDTWEN-IGN PGWNYEQVLP YFRKSLS-CS PEFIAKYG-- ---TDYCGTD GPLKIRNYNY TETDAINILS EAVQQAGYDI

#AmGMC_B9_{GMC-B} ALGGSSVING MIHIFGNRRD FDGWAS-QGN PGWNFEEVLP YFRKSIS-CS PEYIAENG-- ---DKYCGTD GPLRVRYYNY TVTDFEDVVL EAAREAGHPI

#DmEO_B1_CG9504_{GMC-B} MMGGGGAING NIFIPGSREN FRRWN----S TGWDWTQVHK TYSRLQQRLN PSYLQP---- ---------- --------NK LNLKLANLIY SGSAELGVPR

#DmGMC_B2_CG9509_{GMC-B} MLGGSGGVNA MLYVRGNRRD FDGWAA-MGS TGWSYDQVMP FFEKSVTPQG NATHPK---- ---------- GYVTLKPFER KDNDIHQMII DGGRELGQPY

#DmGMC_B3_CG9512_{GMC-B} MLGGTNGMNA MIYARGTRKD FDDWEE-RGN PGWGYDEVLK HFRKAEDLRS TRPDYKP--- ----GDHGVG GPMGLNNYVS -DNEFRTTIR AGMQEMGYGS

#TcGMC_B5_{GMC-B} CLGGSSAINA NLYIRGNRRD YDTWAE-LGN EGWDYDSVME YYKKLEDVDG FDGYGR---- ---------G GFVPLNVYQS -NEPVGEALK DSARVLGYPT

#AgGMC_D1_{GMC-D} VLGGSSVLNT MLYIRGNKRD FDLWQA-LGN PGWGYEDVLP YFRKSEDQRN PYLARNK--- ----RQHGTG GLLQVQDAPY LTPLGVSFLQ AG-EEMGYD-

#AmGMC_D1_{GMC-D} VLGGSSVLNT MLYIRGNRRD FDQWES-FGN PGWGYDDILH YFKKSQDQRN PYLARNT--- ----KYHSTG GYLTVQDSPY NTPLGIAFLQ AG-EEMGYD-

#DmGMC_D1_CG9514_{GMC-D} VLGGSSVLNT MLYIRGNKRD FDQWAD-FGN PGWSYEDILP YFRKSEDQRN PYLARNK--- ----RYHGTG GLWTVQDAPY NTPIGPAFLQ AG-EEMGYD-

#TcGMC_D1_{GMC-D} VLGGSSVLNT MLYIRGNRRD FDHWVH-QGN PGWSYEEILP YFLKSEDQRN PYLARN---- ----KYHSTG GYQTVQDSPY STPLGVAFLQ AG-QEMGYD-

#AgGMC_E1_{GMC-E} VMGGSSVLNA MVYVRGNRLD YDQWQE-QGN VGWGYENVLP YFIKSEDNRN PYMARS---- ----PYHGVG GYLTVQEAPW RTPLSVAFVA AG-QEMGYE-

#AmGMC_E1_{GMC-E} VLGGSSVLNA MVYVRGNRRD YDNWAR-LGN TGWSYEDVLP YFLKSEDNRN PYLART---- ----PYHATG GYLTVQESPW RSPLSIAFLQ AG-QELGYA-

#DmGMC_E1_CG9517_{GMC-E} VLGGSSVLNA MVYVRGSKND YNHWAS-LGN PGWDYDSMLK YFLKSEDVRN PYLAKT---- ----PYHETG GYLTVQEAPW RTPLSIAFLQ AG-IEMGYE-

#TcGMC_E1_{GMC-E} VLGGSSVLNA MIYIRGNRHD YDQWAA-MGN TGWSYPEVLP YFLKSEDNRN PYLART---- ----KYHNTG GYLTVQESPW RTPLSIAFLQ AG-RELGYE-

#AgGMC_G2_{GMC-G} ALGGSSTINA MLYVRGNPRD FDAWRD-LGN PGWSYDDMLP YFLKLEDMRD PRYANL---- ----SYHGRG GPISVERFRY HTPLRNHLLA GL-EEMGLTN

#AgGMC_G3_{GMC-G} VLGGSSVLNA MMYVRGHPED YDEWAR-FGN RGWSWQDVLP YFVKMENVRD PNIAGR---- ----PYHGTT GPMTVELIRN RSALQPMFLQ AA-QELGMK-

#AmGMC_G1_{GMC-G} VLGGSSVLNV MLYIRGNRKD YDNWER-MGN EGWGYEDVLT YFKKSEDMRI EEYRDS---- ----PYHQTG GHLTVEHFHY RLSIIDYLMK AG-TEMGYE-

#DmGMC_G1_CG12398_{GMC-G} VLGGCSSINA MMYIRGNRRD YDQWAA-LGN PGWNYDNILH YFRKLEDMRV PGFEHS---- ----PYHGHG GPISVERYRF PSPLLDIFMR AA-QQLGMVH

#TcGMC_G1_{GMC-G} VLGGSSVLNA MLYVRGNKRD YDRWEM-EGN IGWGYDEVLP YFKKSEDMKI EGYQDD---- ----YYHGTG GYLSVELFRY HSPIADWFLQ AA-QEFGYE-

#AgGMC_I3_{GMC-I} GLGGSTLINY MMYVRGNKYD YDQWSA-AGN DGWSFDEILP YFVKSEKSYL R--E-VN--- ----RYHGMD GNLDVRYLPY RTRLAKLFVN AW-RELGLE-

#AgGMC_I4_{GMC-I} GLGGSTLIDY MLYGRGNPAD YDRWAA-QGN PGWSHADLFP YFLKSERAEL RGLE-NS--- ----TYHGKS GELHVEFPTF RTNLARTFVN GA-REAGHR-

#DmGMC_I1_CG9522_{GMC-I} VLGGTSSINY MIYNRGNRRD FDAWAA-AGN PGWSYDEVLP YFLRSEHAQL QGLE-HS--- ----PYHNHS GPLSVEDVRH RTRLAHAYIR AA-QEAGHP-

#DmGMC_I2_CG12539_{GMC-I} ILGGTSSINY MIYNRGNRRD FDAWAA-AGN PGWSYDEVLP YFLRSEHAQL QGLE-QS--- ----PYHNHS GPLSVEYVRF RSQMVDAFVE AS-VESGLP-

#TcGMC_I5_{GMC-I} GLGGSTLINY MIHVRGNRRD YNRWAK-MGN PGWSYHDIFQ YFLKSEDFLV RKQD--P--- ----GYHTTG GYLGVQDVPY RTQSAHAFVQ AA-QEAGHK-

#TcGMC_I6_{GMC-I} ALGGTSVINY MIYTRGNPLD YQKWG--EVS PGWAFQDVLP YFLKSENCNL GTAC-GS--- ----EYHNKG GPLSVEYP-F KSPITDAFLQ AG-REMGEE-

#TcGMC_I7_{GMC-I} ALGGTSVINF MIYTRGNRHD YDRWAG-QGN PGWSYRDVLP YFIKSERSTL NNPH--P--- ----GVHGTN GYLGVSDI-Y QSEILRAFIE GG-NELGLP-

#AgGMC_K1_{GMC-K} GLGGNTLTNN MLYVRGSEAD YDAWAK-QTN VDWSYRNVLP YFLKLENFRK NASSTSR--- ----QQRGKG GPVPIAGLRE KSPLVRSFIS AC-NRLGLR-

#TcGMC_K2_{GMC-K} ALGGGTAING MMFTRGHPKD YDKWAD-LGN PGWCYNDVLP YFKKLEDADL KEFDHKY--- ----HNRG-- GPFHIEHPQH QTHLTHDVLQ AG-KELGLE-

#TcGMC_K3_{GMC-K} ALGGTTSINS MVYTRGNPRD YDLWSD-LGN EGWCWADVLP YYKKLEDAHF APFDKKY--- ----HHFG-- GPQHLEHPQY LRFLTDHTLE AA-KELDLH-

#AmGMC_L1_{GMC-L} VMGGSSTINY MIYIRGNPND YNEWAK-KGN YGWSYEEVLP YFLKSENNKD REIVKENP-- ----YYHNEG GYQSVERFPY TDINAKILLN AW-QELGHV-

#TcGMC_L1_{GMC-L} VMGGSSTINY MIYIRGNPRD YDEWAE-AGN PGWSWREVLP YFMKSEDNHN IDTVER---- ----QAHGVG GYLSVERFQF QENNVRSLFE AF-QELGLP-

#AmGMC_M1_{GMC-M} VVGGTSTINY MIHTRGNKLD YDRWAK-MGN EGWSYRDVLP YFKKSERFNI PGIE-NS--- ----SYHGYD GRLCVERSPY RSEISKAFLE VG-KEFGYK-

#AgGMC_Q3_{GMC-Q} VMGGSSVLNY MIYTRGNRRD YDAWAA-KGN AGWSFNDVLP YFQKLEKNIV PDS--HP--- ----MYAGRN GPVTISYPSY RTSVARAFVK AN-MELGLP-

#AgGMC_Q4_{GMC-Q} VMGGSSVLNY MIYTRGNRRD FDHWAD-LGN PGWSYKEVLP YFKKLEHSVV PDA--NP--- ----AYAGKD GPLTISYPRF RSDTAKAFVQ GA-IEDGAP-

#AmGMC_Q7_{GMC-Q} VMGGSSVINY MIATRGNKRD YDNWAK-MGN FGWSYDDVLK YFKRLENMMI PEYRNDT--- ----VHHGTK GPVTINYPRF ATTVARTFVE AG-HELGYP-

#DmGMC_Q1_CG9519_{GMC-Q} VMGGSSVLNY MMYTRGNRRD YDRWAR-LGN PGWSYEEVLP YFKKYEGSVV PDA--DE--- ----NLVGRN GPVKVSYSET RTRIADAFVG AT-QDAGLP-

#DmGMC_Q2_CG9521_{GMC-Q} VMGGSSVLNY MMYTRGNRRD YDRWEA-LGN PGWSFKDVLP YFKKYEGSSV PDA--EE--- ----DYVGRN GPVKVSYVNW RSKIAEAFVD AA-QQDGLK-

#TcGMC_Q5_{GMC-Q} VVGGSSVLKY MIYTRENHRD YDHWAD-LGN TGWSFKEVLP YFKKVENFSV PDSP-YP--- ----EYHSKE GYLSVSYAPF KTKIADAIIE AS-NQNGIK-

#TcGMC_Q6_{GMC-Q} VVGGSSVLNY MIYTRGNWRD YDKWAE-LGN EGWGFKDVLP YFKKIENFMV PGPY-NA--- ----SYHNHD GYLAVSYSPY KTKIADAVLE SA-QLMGLK-

#AgGMC_Z1_{GMC-Z} VLGGSSVLNY MIYVRGNRND FNHWES-LGN PGWAYDDVLQ FFVKSEDNRN PYLARN---- ----PYHGQG GLLTVQEAPW HTPLVAAFVE AG-TEIGYE-

#AmGMC_Z1_{GMC-Z} VLGGSSVLNY MLYVRGNRHD YDHWES-MGN PGWGYDQALY YFKKSEDNRN PYLQKS---- ----PYHSTG GYLTVQESPW KTPLVVAFVQ AG-TEIGYE-

#DmGMC_Z1_CG9518_{GMC-Z} VLGGSSVLNY MLYVRGNRHD YDHWAS-LGN PGWDYDNVLR YFKKSEDNRN PYLANN---- ----KYHGRG GLLTVQESPW HSPLVAAFVE AG-TQLGYD-

#TcGMC_Z1_{GMC-Z} VLGGSSVLNY MLYVRGNRHD YDQWEA-MGN HGWNYENVLH YFKKSEDNRN PYLART---- ----KYHNQG GLLTVQESPW RTPLVLAFVQ AG-TELGYP-

#AgGLD_{Insect_GLD-GOX} VLGGTSVLNG MMYIRGNPQD YDDWDA-MGN PGWKWKDVLP YFMKSEDNLQ I-NEVDS--- ----KYHSTG GMLPVGRFPY NPPFSYSVLK GG-EQLGYQV

#AmGLD_{Insect_GLD-GOX} VLGGTSVMNG MMYIRGSRKD YDDWAR-LGN IGWSYQDVLP YFIRSEDNLQ A-NTMDY--- ----GYHGVG GPLTVTQFPY HPPLSYSILE AG-KELGYGI

#AmGLXr_1_{Insect_GLD-GOX} MMSGTAGMYG MMYSRGHPEV YNGWAR-GGA TGWSYDEVTH YFERAEDPID QSILSDKP-- ----RTVPVP GPMKIQFYPD KPAFADEILK AA-SELGYRT

#AmGLXr_2_{Insect_GLD-GOX} NLGGTSSHNG MMYTRGHPKD YDDWAA-MGN DGWSWQDVLP YFMCSENNTE I-NRVGR--- ----KYHSTG GLLNVERFSW RPDISNDILA AA-AELGYPI

#AmGOX_{Insect_GLD-GOX} NLGGTTLHHG MAYHRGHRKD YERWVQ-QGA FGWSWDEVMP YYLKSENNTE L-SRVGT--- ----KYHRSG GLMNVERFPY QPPFAWKILK AA-EEAGFGV

#DmGLD_{Insect_GLD-GOX} VLGGTSVLNG MMYVRGNRED YDDWAA-DGN PGWAYNDVLP FFKKSEDNLD L-DEVGT--- ----EYHAKG GLLPVGKFPY NPPLSYAILK AG-EELGFSV

#TcGLD_{Insect_GLD-GOX} VLGGTSVMNG MMYIRGSRKD YDDWAK-AGN EGWSYNEVLP YFLKSEDNKQ A-DSMDR--- ----GYHSTG GLLTVSQFPY HPPLSQALLK AA-QELGYPI

#TcGLXr_3_{Insect_GLD-GOX} VLGGCSVING MMYMRGHPKD YDNWAT-MGN TGWGYQDVLP VFKKSEDNLQ IGTLVDA--- ----AYHGTG GPMTTSRFPH HPELAEDVMQ AA-KELGYPV

#TcGLXr_4_{Insect_GLD-GOX} VLGGSGVIHG MMYMRGLPSD YNEWEA-RGN EGWGYKDVEE YFKKSEGNRD IGDGVEG--- ----RYHSSD GPMLVQRFPD QPQIAEDVLR AG-AELGYPV

#DmNinaG_CG6728_{NinaG} GLGGSANLNY MLHFDGHGPD FDSWRDHHNL SDWSWAQMRS FMAAAKPKNP ---------- ---------- ---DMLEIPR RYSKLTEALE EAQAQFAYK-

#Tc_NinaG_like_{NinaG} ILGGTAMLNN MIYVRGHPQD FAEWYKDSCN FNYT-IDVLP YFKKLESNET NKHKCS---- ---------- --VFVEDMPF KSNLSDYFLQ AG-LCLGFG-

#Tc_XM_961446_{otherBeetleGMC} GVGGSTLING LIYSRGHKTD FDHWGRLVGN DRWSYRSVLQ YFKKSENFVY RDYTQPIE-- ---PEYHGTN GYWQVEHHLP RSPQLDVFLD AN-REMGLGV

#Tc_XM_961538_{otherBeetleGMC} GIGGSTLING LVYSRGHKTD FDKWGEVVGS KRWSYNKVLK YFKKSEDFVY RDYEVPYE-- ---PQYHGTG GYLRVENYIY RSPQLNAFLA AN-QELGLGV

#Tc_XM_967481_{otherBeetleGMC} AIGGSSTINA IMYVRGNPED YNEWVR-LGN PGWSYEEVLP YFLKSE---- -NSQVEGD-- ---PGFHGKG GLWNIQYSLP PSELFSNFLQ AN-KELGLEA

#Tc_XM_968249_{otherBeetleGMC} GVGGSTLING LVYARGHKSD FDKWGKVAGN RRWSYETVLK YFKKSENFVY RDADAPYE-- ---PPYHGEG GDLQVEYHLP RSPQLNAWLE AN-RELGYEI

#Ag_CG6142like_1_{CG6142} -ETDPNG-EI QLGFGKAQAT MRR-GRRCSA SKAYLVP-AS R-RPNLDISM YSRVTKVLID PVT--KHAYG VEFI------ -----KRRRR YVIRARKEVI

#Ag_CG6142like_2_{CG6142} -HIDPND-PV QLGFYKAQAT MVN-GERCSA ARAYLKP-VA D-RPNLDIST RSWATRILID PVT--KTAFG VEFT------ -----KNKRL HTVRVRKEVI

#Am_CG6142like_3_{CG6142} -VIDYNA-AN VIGFSTAQVH LRN-GRRVSA SKAFLRP-IR E-RKNFHLSK LSRATRIVID PKK--KVAVG VEFV------ -----KNGRK RFVSASKEII

#Dm_CG6142_{CG6142} -ITDPNG-EH LMGFARSQAT IRN-GRRCST SKAFIQP-VV N-RKNLHISM KSWVTRLIID PIT--KTATG VEFV------ -----KQRQR YVVRARKEVI

#Tc_CG6142like_4_{CG6142} -NNDPSA-PY GLGFSKVLAT MRN-GMRCSA SKAFLKP-IL H-RTNLHVSI KTRVTKILID PST--KQAYG VQFW------ -----KNRRK FTVLATKEVV

#Cele_CHD_{CHD} WTNDMNG-EK QEGISTMDMT IHN-GERWSA SKAYVHP--I RNRPNLITSS GITCTRVLFD TNK----AIG IEFIRKLNFV GTDSIDSYSR EKIYCQGDVI

#Ecol_CHD_{CHD} RTDDLNG-YQ QEGFGPMDRT VTPQGRRAST ARGYLDQ--A KSRPNLTIRT HAMTDHIIFD GKR----AVG VEWLEGD--- ------STIP TRATANKEVL

#Human_CHD_{CHD} LTEDMNG-FQ QEGFGWMDMT IHE-GKRWSA ACAYLHP--A LSRTNLKAEA ETLVSRVLFE GTR----AVG VEYVKNG--- --------QS HRAYASKEVI

#Anig_GOX_{Fungal_GOX} PTKKDFGCGD PHGVSMFPNT LHEDQVRSDA AREWLLP--N YQRPNLQVLT GQYVGKVLLS QNGTTPRAVG VEFG------ ----THKGNT HNVYAKHEVL

#Aory_GOX_{Fungal_GOX} PTQQDFHCGH PRGVSMIPNA VHEDQTRSDT AREWLLP--N HERPNLKVLT GQRVGKVLLN KTESGAKATG LNFG------ ----THRKVN YNVYAKHEVL

#Pama_GOX_{Fungal_GOX} PVQQDFLCGH PRGVSMIMNN LDENQVRVDA ARAWLLP--N YQRSNLEILT GQMVGKVLFK QTASGPQAVG VNFG------ ----TNKAVN FDVFAKHEVL

#AgGMC_A1_{GMC-A} -NRDLNG-AK QTGFMIAQGT IRR-GGRCST GKAFLRP-AR L-RPNLHVAM FAHVTRVMID PIS--KIAFG VEFI------ -----RDRKV HHVRASKEVI

#AmGMC_A1_{GMC-A} -NRDING-EQ QTGFMIAQGT IRR-GSRCST AKAFLRP-AR L-RKNLHIAM QSHVTKILID PKS--KRAYG VEFV------ -----RDQKM FRIRAKKEVI

#DmGMC_A1_CG9503_{GMC-A} -NRDLNG-EK MTGFMIAQGT TRR-GSRCST SKAFLRP-AR L-RPNLHISM NSHVTRIMID PVT--KLAFG VEFV------ -----KDQKL YHVRATKEVV

#TcGMC_A1_{GMC-A} -NRDING-EH QSGFMMAQGT TRR-GSRCST GKAFLRP-VR L-RKNLHVAM HAHVTKVMVD PTS--KVAFG VEFV------ -----RDKKL YRIRATKEVV

#AgGMC_B4_iso1_{GMC-B} LEDFNGENHI G--FGRVQLN TIE-GARCSP AKAFLAP-IK DRR-NLHVIK RALATKLEVD AHQRVSS-VR FVIDEHNDSS N----DQTRV LEVKVRKETI

#AgGMC_B4_iso2_{GMC-B} IDDFNRDRHN G--YGNTQYT IIG-GTRCSP AKAFLTP-VR KRQ-NLHVIK YAFVNRVLID ERNVATG-VR FVVDGSQ--- --------RV QQVAVRREVI

#AgGMC_B4_iso3_{GMC-B} TADINGARQV G--FGRAQGT IVN-GTRCSP AKAFLVP-VK DRP-NLHVIK HAVVVTVERD PSTERFKYVN FMIDNKV--- ---------L KVAHARKEVI

#AgGMC_B4_iso4_{GMC-B} IMDINSDEYI G--YNVAQGT VHK-GRRWST AKAFLNT-AA DRP-NLHIIK NAHVTKINFE GTAATG--VT FDVPSQT--- ---------G VSASIRKEVI

#AmGMC_B10_{GMC-B} LEVVNGDRFV G--FGRAMGT VHD-GRRENA AMAFLSP-AK GRK-NLSVMK SSAVEKVLF- EEGRAIG-VR VRSEQKG--- --------FV AEVRARKEVI

#AmGMC_B6_{GMC-B} LEPLNGDRFV G--FGRAMGT LDD-GRRLNA AKAFLSP-VK YRR-NLYVMK SSRVDRVLFG EDGRASG-VR ITLKNN---- --------EQ IDVRAAKEVI

#AmGMC_B7_{GMC-B} LEPLNGDRFI G--FGRAMGT LDN-GQRENC AKAFLSP-VK DRK-NLYVMT SSRVDKILF- ERKRAVG-VR ITLDNN---- --------QS VQVRATKEVI

#AmGMC_B8_{GMC-B} LEPVNCDRFI G--FGRAMGN IDN-GQRQSC AKAFLSP-VK NRE-NLYVMT SSRVDKILF- EGERAVG-VR ITLDND---- --------EP IEVKATKEVI

#AmGMC_B9_{GMC-B} LKAVNGDRYL G--FGRVLGT LDE-GRRQTC SKAFLTP-VR DRK-NLYVIT STRANKILF- EGKRAVG-VQ ITLSNN---- --------ET AEVRATKEVI

#DmEO_B1_CG9504_{GMC-B} MKQPLIAGAT FGYTHHVPVT VNQ-RRRASS ARLYLANDQV NRRGNLKVIR GAQVQRVLLN AAGSRATGVI YTLNGVE--- ---------- HTAKTLGEVI

#DmGMC_B2_CG9509_{GMC-B} VERFQEGSDT G--YSHVPGT VRQ-GQRMST GKGYLGAVSK SRP-NLHVVK NALVTKLDLD G-ETVKE-VK FERAG-V--- ---------T HRVKVTKDVV

#DmGMC_B3_CG9512_{GMC-B} APDFTEGSFV G--QMDILGT QDG-GRRITT ARSHL---KK NTP-NLHILR HAHVKKINLD RNNRAES-VT FVHRGKK--- ---------E YTVKASKEVI

#TcGMC_B5_{GMC-B} IP---QEGNF G--YFEALQT VDK-GIRANA GKIFLGR-AK DRE-NLVVAM GATVEKILLK EKKTEG--VL VNIGGRQ--- ---------- IALKARKEVI

#AgGMC_D1_{GMC-D} -IVDVNG-EQ QTGFAFFQFT MRR-GTRCST SKAFLRP-VR N-RKNLHVAL FAHVTRVILD PET--RRALG VEFI------ -----RNGKT HKVFATREVI

#AmGMC_D1_{GMC-D} -IVDING-EQ QTGFALYQYT MRR-GTRCSA AKAFIRP-IQ L-RRNFDLSL WSHVTRILID PRT--KRARG VEFI------ -----RGGRR EVVHARKEVI

#DmGMC_D1_CG9514_{GMC-D} -IVDVNG-EQ QTGFGFYQFN MRR-GSRSST AKSFLRP-AR L-RPNLHVAL FSHVTKVLTD PHT--KRATG VQFI------ -----RDGRL QNVYATREVI

#TcGMC_D1_{GMC-D} -IRDVNG-EK QTGFAFFQFT MRR-GTRCST SKAFLRP-IR L-RKNLHISL WSHVTKVLID PES--RRAYG VEFI------ -----KNGKK QIVLARKEVI

#AgGMC_E1_{GMC-E} -NRDING-AE QTGFMLLQAT IRR-GSRCST SKAFLRP-VR L-RKNLHIAM NAHVTRILFD -DQ--HRAYG VEFV------ -----RHQKR QYVFARKEII

#AmGMC_E1_{GMC-E} -NRDVNG-AY QTGFMLNQGT IRR-GSRCST AKAFLRP-VK N-RPNLHVAM KTQALRIVFN -EG--RRATG VEVL------ -----RYGRH HFIRTRREIV

#DmGMC_E1_CG9517_{GMC-E} -NRDING-AQ QTGFMLTQST IRR-GARCST GKAFIRP-VR Q-RKNFDVLL HAEATRILFD -KQ--KRAIG VEYM------ -----RGGRK NVVFVRREVI

#TcGMC_E1_{GMC-E} -VRDLNG-EK QTGFMLSQGT IRR-GSRCST SKAFLRP-VK S-RQNLHIAM YSQVTKVMID PKT--KTAYG VKFT------ -----RNNRP QTVRARREVI

#AgGMC_G2_{GMC-G} RYGEVNG-PM QSGFAVPHGS IRN-GLRCST AKGYLRP-AA A-RKNLHIST KTMVERVLID PND--RRAYG VQFE------ -----KGGRR YQVMVSKEVI

#AgGMC_G3_{GMC-G} LADEVNG-PD QLVFAPLHGS IRD-GLRCST AKAYLRP-IG N-RKNLHISM NSMVERILID PKD--RRAYG VVFR------ -----KGNRR QFVLVTKEIV

#AmGMC_G1_{GMC-G} -IVDVNG-AR QTGFTYSHGT LRN-GLRCSA AKAFLRS-VS R-RRNLDIGT KSMVEKILV- ---------- ---------- ---------R R---------

#DmGMC_G1_CG12398_{GMC-G} PDGDFNG-RS QTGFAPPHGS LRD-GLRCSA NKGYIRR-SW Q-RPNLDIVL KAFVERIVID PQS--HRAIG VIFE------ -----YGLLK HTVRAKREVI

#TcGMC_G1_{GMC-G} -IRDING-EY QTGFTLAHGT LKD-GLRCST AKGFLRP-VS K-RPNLHVSL HSLVEKIIID EVT--KQARG VTFN------ -----KFGAR RTIYSDRETI

#AgGMC_I3_{GMC-I} -SVDYNG-ES QIGVSYIQSN VRN-GRRLTA YTAFLEP-IL D-RPNLHILT NARATRVLID ATT--QQAYG VEFI------ -----KDRNR YTVYADKEIL

#AgGMC_I4_{GMC-I} -KLDYNG-KS QLGVSYVQTT GLR-GMRQTA YRAFVEP-VL YKRPNLHVQP YSQVLKVLIN PDT--QTAYG VTYT------ -----RHFRN YEVRARKEVI

#DmGMC_I1_CG9522_{GMC-I} -RTDYNG-ES QLGVSYVQAT TLK-GRRHSA FRAYIEP-IR SRRHNLHILT LARVTRVLID AAT--KSAYG VELT------ -----HQGRS FKVKARKEII

#DmGMC_I2_CG12539_{GMC-I} -RTDYNG-ES QLGVSYVQAN TLN-GRRHSA YSAYIKP-VR DLRSNLQIFT FSQVTRILID EAT--KSAYG VEFH------ -----YKNKA YTFKARKEVI

#TcGMC_I5_{GMC-I} -FVDYNG-KR QMGVSYVHAT TRN-GKRSSA EEAFLRP-IK H-RQNLKIST KSRVTKVLID PQT--RQAYG VQYI------ -----KNGKY HTVLASKEVI

#TcGMC_I6_{GMC-I} -IVDYNT-EK YMGFGQLQAN QKF-GRRHST FDAFIAP-II T-RKNLHIVS GARVTKILID PNT--RQTLG VIFE------ -----KKGQK YKIRASKEVI

#TcGMC_I7_{GMC-I} -YFDYNANEK SFGVSPIQAT VKR-GRRHTT ARAFLHP-IR H-RKNLHMLT SAFVTKVLID PNT--RQTYG VEFS------ -----RFGRK YQVTASKEVI

#AgGMC_K1_{GMC-K} -TTDYNA-ER NQTVGFVQLT QYR-TKRITA ADAYIRP-VK QLFNNLHIMS SARVTKVLIN GMN--RQAVG VKVL------ -----VNGKQ RKLRATKEVI

#TcGMC_K2_{GMC-K} -TIDYNG-KE QMGLGVLQMN SKH-GVRQST ATAYLEP-AE KRQN-LFVKP LSHVTKILIA PHT--KEATG VEYL------ -----HNDKL HIAKATKEII

#TcGMC_K3_{GMC-K} -LIDYNG-KH QIGISVPQLT SKC-GKRFST AEAYLER-AE KRDN-LIVKP LSQVLKVLIS THT--KEAQG VVYL------ -----HEGKT FVAKAEKEVV

#AmGMC_L1_{GMC-L} -TVDANA-GT QLGVMKLQMT SLH-GKRESV NSAYIRP-IR HKRKNLTIET QAHVTRLLTD PTT--KRVTG VDYTCT---- -----STGLS KSVLARKEVI

#TcGMC_L1_{GMC-L} -VVDQNA-GR QIGTMMLQTT TRS-GRRESA NLAFIRP-IR RKRKNLTIET KAYIIRVLID PHT--KVAYG VEYE------ -----KNGKL FQARARKEVL

#AmGMC_M1_{GMC-M} -VVDYNG-EK QIGFSLIQAN LDA-GMRCSA AKAYLR---- VNRPNLNIVT QARVTKLLIE G----RQVHG VVYA------ -----RNKRW TKVFATKEVI

#AgGMC_Q3_{GMC-Q} -YVDYNG-PS QIGTSFIQST TKN-GQRVSS NNAYLYP-IR -NRTNLHIIR NAHVTKILLN RDT--KRATG VQFY------ -----ANHRY QKVRARREVI

#AgGMC_Q4_{GMC-Q} -YVDYNG-PT QIGVSYIQST TKD-GKRDST NVAYLYD-MR -NRSNLHVKK NSQVTRILFD RSA--NQANG VRFF------ -----HAGRF HTVRARREVI

#AmGMC_Q7_{GMC-Q} -ILDYNG-ER QVGVSLLQST TDM-GLRTSS NKAYLVG-KR --RKNLHVTK LSTVRRILFD EGR--GRAVG VEFA------ -----KRGRL FTVYVDKEVI

#DmGMC_Q1_CG9519_{GMC-Q} -RGDYNG-DK QIRVSYLQAN IYN-ETRWSS NRAYLYP-IK GKRRNLHVKK NALVTKILID PQT--KSAFG IIVK------ -----MDGKM QKILARKEVI

#DmGMC_Q2_CG9521_{GMC-Q} -YRDYNG-RI QNGVAFLHTT TRN-STRWSS NRAYLYP-LK GKRSNLHVRK NALVTKVLID PQT--KTAYG IMVQ------ -----TEGRM QKILARKEVI

#TcGMC_Q5_{GMC-Q} -SVDYNG-PI QVGVSRLQVS MRD-GVRESA SRAYLHP-IR -NRPNLHVKK LAMVSKVLID PKT--KQTIG VEFF------ -----RDGTR YQIRASKEVI

#TcGMC_Q6_{GMC-Q} -LVDYNG-PI QVGVSRFQVT LRD-GIRESS SRAYLHP-IK -NRPNFHMRK YSTVTKILID PTT--KKVQG VEVD------ -----TKGTI YKIGASKEVL

#AgGMC_Z1_{GMC-Z} -NRDING-ER QTGFMIAQGT IRR-GSRCST AKAFLRP-IR L-RKNLHIAM NSHVSKLVID PET--KHAVG VEFF------ -----RGGKR HYVRARKEII

#AmGMC_Z1_{GMC-Z} -NRDING-ER QTGFMIAQGT IRR-GSRCST AKAFLRP-IR L-RRNIHTAM NCHVTRILID PIA--MRATG VEFV------ -----RDGRR QIVRARKEVI

#DmGMC_Z1_CG9518_{GMC-Z} -NRDING-AK QAGFMIAQGT IRR-GSRCST AKAFLRP-IR M-RKNFHLSM NSHVTRVIIE PGT--MRAQA VEFV------ -----KHGKV YRIAARREVI

#TcGMC_Z1_{GMC-Z} -NRDING-AE QAGFMVAQGT IRR-GSRCST AKAFLRP-IR L-RKNIHIAL NSHVTRVLIN PST--MRAFG VEFV------ -----RNGHK QIVLARKEVI

#AgGLD_{Insect_GLD-GOX} Q-DLNGA--N TTGFMIAQMT NKN-GIRYSA ARAFLRP-AV N-RANLHILL NTTVTKVLVH PTS--KTAHG VEIVD----- -----EDGHM RKILVKKEVI

#AmGLD_{Insect_GLD-GOX} A-DLNGR--T HTGFAIAQTT SRN-GSRLST ARAFLRP-AK N-RPNLHIML NSTATRILFD NN---KRAVG VEFVH----- -----D-GKI HRVSVAKEVV

#AmGLXr_1_{Insect_GLD-GOX} S-KLKEY--T QTGFMIAPMT TDN-GVRGTA TRNYLRP-VH G-RSNLRVLI NAHVTKVLMD WQG---KAYG VELVD----- -----KDGYK RIAKANKEVV

#AmGLXr_2_{Insect_GLD-GOX} PEELNGD--Q FAGFTVAQMM SKD-GVRRST ATAFLRP-FR N-RSNLQVIT NATVTKILLK EKK----AVG VQYYK----- -----N-GEL RVARASREII

#AmGOX_{Insect_GLD-GOX} SEDLSGD--R INGFTVAQTI SRN-GVRLSS ARAFITP-FE N-RSNLHVIV NATVTKVRTL NKR----ATG VNVLI----- -----N-GRR RIIFARREVI

#DmGLD_{Insect_GLD-GOX} H-DLNGQ--N STGFMIAQMT ARN-GIRYSS ARAFLRP-AR M-RNNLHILL NTTATKILIH PHT--KNVLG VEVSD----- -----QFGST RKILVKKEVV

#TcGLD_{Insect_GLD-GOX} R-DLNGA--Y HTGFNIAQTT NRN-GSRLST AKAFLRP-FK N-RRNLNILM NSTVTRVLIN TTT--KQAYG VEVIN----- -----N-GVK QVIYASKEVI

#TcGLXr_3_{Insect_GLD-GOX} SDDLNGR--Q YHGFTIAQSS VRN-GSRLSS ARAFLRP-GR D-RPNLHVML NSTATKILIN SSNNQKTVSG VQFLY----- -----N-NKL HTVRVKREVV

#TcGLXr_4_{Insect_GLD-GOX} VGDLNGE--Q HWGFTIAQAN IKN-GSRLSS ARAFLRP-AR N-RPNLHVMI NSTATKILIN SNDTAKTISA VEFTY----- -----N-NQS FTVKVRREAI

#DmNinaG_CG6728_{NinaG} ---------- DWIFRRSLYN IRN-GLRHSV VQQFLNP--V IHHSNLRLLP DALVKRIQLA PSPFLQATSI LVGIKDE--- ----ENREKE FSIEVRRELI

#Tc_NinaG_like_{NinaG} ---LSDGVNS EPGFSATKVT MRN-GQRWTP YHQLEKT--- -KKRNLVVIT NSLVEKVLLK SNY---EAYG VKYT------ -----HLDET YYVRATKGVI

#Tc_XM_961446_{otherBeetleGMC} ADYNAN---- RLGASSAQLN TAF-GRRMDT GKAFIRS-VL K-RPNLKVLT GSFVTRIVID KFT--RSAVG VEFT------ -----HGGSN YFVRAKKEVI

#Tc_XM_961538_{otherBeetleGMC} VDYNAN---- KLGASASQLN THN-GRRFDG GKAFIHP-VL N-RPNLKVLT GSYVTRIVIN KET--KSATG VEFT------ -----HDGKY YYVEAKKEVI

#Tc_XM_967481_{otherBeetleGMC} VDYNGYR--- QFGASKAQTN IKH-GKRQST GTAFLKY-AR Q-RRNLNVIT NALVTEIVID KKN--KSAEG VMFI------ -----KDNQK FRANANLEVI

#Tc_XM_968249_{otherBeetleGMC} VDYNAN---- RLGASPSQLN TRN-GRRDDD GQAFLRH-AR K-RRNLKILT GSYVTKIQIE KE----SANG VEFT------ -----HKGKN YYVEVRKEVI

#Ag_CG6142like_1_{CG6142} LAAGAIASPQ LLMLSGVGPR EHLKEMGIPV VQDLP-VGYN MQDHLNLPGL VFPVNQP--- ----VTVRER DMR-SPRPII DYLVH--GRG PFTSPGGAEG

#Ag_CG6142like_2_{CG6142} LAAGAIASPQ LLMLSGVGPR EHLQQLDIPV VKDLR-VGYN LQDHQTLSGL VFTVNQP--- ----VTIRER DMR-RPAPFL SYLFA--RRG PFTVPGGAEG

#Am_CG6142like_3_{CG6142} LSTGTLNSPQ LLMLSGIGPK DHLESLNIDS IEDLQ-VGYN LQDHVSMSML TFLVNES--- ----VTIVEP RIASNLANIL DYFVK--GTG PLTVPGGAEC

#Dm_CG6142_{CG6142} LSAGTIASPQ LLMLSGIGPA EHLREHNITV MQDLP-VGYN LQDHITLNGL VFVVND---- ----STVNDA RLL-NPSDIF RYIFA--GQG PYTIPGGAEA

#Tc_CG6142like_4_{CG6142} LSAGSINSPH LLMLSGVGPR DDLTRVGIPL LQNLK-VGYN LQDHMAMSAL VFFVNES--- ----ITVSDR GVQ-NPVDIF NYVFN--GRG PYTIPGGAEA

#Cele_CHD_{CHD} LAGGAINTPQ LLMLSGVGPA DHLRSHEIPI VANLPGVGQN LQDHLEIYVQ QESTQPVTLY N---KSSWKF PHNMIKIGLE WFTNR----T GLGASSHLET

#Ecol_CHD_{CHD} LCAGAIASPQ ILQRSGVGNA ELLAEFDIPL VHELPGVGEN LQDHLEMYLQ YECKEPVSLY ------PALQ WWNQPKIGAE WLFGG----T GVGASNHFEA

#Human_CHD_{CHD} LSGGAINSPQ LLMLSGIGNA DDLKKLGIPV VCHLPGVGQN LQDHLEIYIQ QACTRPITLH ------SAQK PLRKVCIGLE WLWKF----T GEGATAHLET

#Anig_GOX_{Fungal_GOX} LAAGSAVSPT ILEYSGIGMK SILEPLGIDT VVDLP-VGLN LQDQTTATVR SRITSAG--- ---------- --AGQGQAAW FATFN----- ETFGDYSEKA

#Aory_GOX_{Fungal_GOX} LAAGSAISPL ILEWSGIGLK DVLSAAGVEQ VVDLP-VGLN MQDQTTTNVR SQAQASG--- ---------- --AGQGQAVY FASFN----- ETFGDYAHKA

#Pama_GOX_{Fungal_GOX} LAAGSAISPL ILEYSGIGLK SVLDQANVTQ LLDLP-VGIN MQDQTTTTVS SRASSAG--- ---------- --AGQGQAVF FANFT----- ETFGDYAPQA

#AgGMC_A1_{GMC-A} VSGGSVNSPQ ILMLSGIGPK SELAKHRIPL IKDLP-VGEN LQDHIGLGGL TFMVNQP--- ----VSIVEN RYHSMSTVLQ YAVLG---QG PLTILGGVEG

#AmGMC_A1_{GMC-A} VSGGSINSPQ LLMLSGIGPR EHLSKHGIPV IQDLR-VGFN MQDHVGLGGL TFLVDKE--- ----ISMVEK RLHTVQTVMQ YAIFG---NG PLTVLGGVEG

#DmGMC_A1_CG9503_{GMC-A} LSGGSVNSPQ LLMLSGVGPR KELAKHRIPL IKELS-VGEN LQDHIGLGGL TFLVNQP--- ----VSIVEN RFHTMSTVLQ YAVFG---QG PLTILGGVEG

#TcGMC_A1_{GMC-A} LSAGAVNSPQ LLMLSGIGPK EDLERLKIPL VQDLK-VGHN LQDHVGLGGL TFLINRP--- ----HSILLN RLYSVSSLMQ YAIFG---GG PLTIMGGVEG

#AgGMC_B4_iso1_{GMC-B} VSAGAVNTPQ LLMLSGIGQE EDLREHGIRI VSDLP-VGRN LQDHVMVPLF YCINR----- --SSATDFDL NRNVIGHMYD YLMH---RNG PLSEIGINAF

#AgGMC_B4_iso2_{GMC-B} LAAGAINTPQ LLMLSGVGRT DELKQFGIPP KVDLN-VGGN LQDHVAVPLF FKFYA----- --LQEQDINE QLARINELYT YVVQN--RSQ AVVRTGPLNT

#AgGMC_B4_iso3_{GMC-B} LAAGAINTPH ILQLSGIGPK ALLEKVNIPL VADLP-VGEN LQDHLFVPLL FKMHK----- --STAENYNI QQELAKNLFQ YIMT---RSG PMAGHGVTSV

#AgGMC_B4_iso4_{GMC-B} ISAGAINTPQ VLQLSGLGAK EQLDRLDIPL VKEIPSVGEN LQDHLIVPLF LSLHG----- --SRPIER-S MDELLDSIYS YFRY---GLG TFGTVGITDL

#AmGMC_B10_{GMC-B} LSAGSIATPQ LLMLSGIGPR EHLEKMGIPV VADRP-VGKN LQDHLAWTGM YITYAN---- -ESSISSPSL NRSLSS-IYE YMME----NR GPLRAYRTDF

#AmGMC_B6_{GMC-B} LSAGSVASPQ ILMLSGIGPR RHLDEMGISL VHDLP-VGEN LQDHAIWLGT NLLFVN---- -ESITSPMPV DAIYDS-AYE YLIH----KT GQLRDLPIDL

#AmGMC_B7_{GMC-B} LSAGSIASPQ VLMLSGIGPK NHLKKMGIPT LVDLP-VGKN LQDHAIWLGI YLAYNN---- -ESVTSPPSE KSQLDD-IYD YLEF----NA GPLRVLPLDL

#AmGMC_B8_{GMC-B} LSAGSIASPQ ILMLSGIGPK EHLNKMGIPT LVDLP-VGMN LQDHVSWLSF YLRYTN---- -ESITPPFDE KNQLDDAVYE YLKQ----NT GPLRTLPVEF

#AmGMC_B9_{GMC-B} LSTGTMVSPQ LLMLSGIGPK EHLKKLGIPV LVDLP-VGKN LQDHVIWFGL YYSFVN---- -ESVTSAPSE KDQLDS-AYE YLEF----NT GPLSTLANDL

#DmEO_B1_CG9504_{GMC-B} LSAGTLNSAK LLLLSGIGPR EELQRWNITT HQDLP-VGRN LQDHGMMPLF LLFGSNCA-- -VNSTRDPTE NPYAPVSITQ YLLDN--QKG PLASG--FYM

#DmGMC_B2_CG9509_{GMC-B} ISAGAIDSPA LLLRSGIGPS KHLKELGIPV KLDLPGVGRN LQDHVLVPVF LRLDE----- --GQGEPM-T DQAALDSIYQ YLIY---RAG PLAAHSTASL

#DmGMC_B3_CG9512_{GMC-B} VSAGAIGSPQ ILLLSGIGPA DHLKSLGIPV KLDLP-VGEN LKDHASLPMI FQIDK----- --STARKP-T EEELVDAMYN LLMG---RYS KLLHHEATAL

#TcGMC_B5_{GMC-B} LSAGAINSPQ LLMLSGIGPK KHLQDVGIDP VMDLQ-VGEN LQDHIFYLGL LVAVD----- --DKVSQV-- QTNVIDEIYK YFMY---NEG AVGQIGITNL

#AgGMC_D1_{GMC-D} LSAGAIGTPH LMMLSGIGPR ENLERVGIPV FHDLPGVGQN LQDHIAVGGL VFRIDQP--- ----ISVIMN RLVNLNSALR YAVTE---DG PLTSSIGLEA

#AmGMC_D1_{GMC-D} LSAGAINSPQ LLMLSGIGPR RHLEELGIPV IHDSPGVGQN LQDHIAVGGI IFPIDYP--- ----ISIMLD RVVNLNSALR YAITE---DG PLTANVGLET

#DmGMC_D1_CG9514_{GMC-D} LSAGAIGSPH LMMLSGIGHG EELGRVGIPL VQHLPGVGQN LQDHIAVGGI AFLIDYP--- ----ISIVMK RMVNINTALR YAITE---DG PLTSSIGLEA

#TcGMC_D1_{GMC-D} LSAGAINSPQ LLMLSGVGPA EHLQEKGIRV IHDSPGVGQN LQDHIAVGGL TFLIDPP--- ----ISLLVN RLVNLNTALR YAIKE---DG PLTSSIGLEA

#AgGMC_E1_{GMC-E} LSAGALNTPQ ILMLSGVGPA DHLDELGIPV VSDLP-VGDN LQDHVGLGGL TFLVDQP--- ----VTVKTS RYSSVPVALE YFLNE---RG PMTFPG-IEG

#AmGMC_E1_{GMC-E} LSAGAINTPQ LLMLSGIGPK EHLAEFGIPV ISDLR-VGDH LQDHVGLGGL TFVIDEP--- ----VSLKRD RFQTLSVMMQ YVLHE---RG PMTDSG-VEG

#DmGMC_E1_CG9517_{GMC-E} ASAGALNTPK LLMLSGVGPA EHLQEHNIPV ISDLP-VGNN MQDHVGLGGL TFVVDAP--- ----LTVTRN RFQTIPVSME YILRE---RG PMTFSG-VEG

#TcGMC_E1_{GMC-E} LSAGAIGTPH ILMLSGVGEK SHLESFKIPV MSDLK-VGYN LQDHIGLGGL TFVIDDP--- ----ITFTKT RYQTFAVAME YIVNE---RG PMTSLGGVEG

#AgGMC_G2_{GMC-G} LSAGALNSPQ LLMLSGIGPR QELERHGIRV IQDLPGVGQN MQDHVATGAG GYTIRPPPG- -SPPLAYDFG DAVGVDTLRR FLFNE---DG MLYGMSLCEV

#AgGMC_G3_{GMC-G} LSAGALNSPH LLMLSGVGPR DQLQRHGIRV IHELPGVGQN LQDHVAAGGG VFLIQNPTG- -SAPLSIRLV EVNEVSVARD FLFRN---QG RLLSMPSCEV

#AmGMC_G1_{GMC-G} ---GKL---- ---------- ---------- ---------- ---------- ---------- ---------- ---------- FANNY---SG PMYSVNVAEG

#DmGMC_G1_CG12398_{GMC-G} LSAGSLASPQ LLMVSGVGPR DQLEPQGIPV VQHLPGVGGN LQDHISTSGA IYTFDSGQ-- -NRHLSFIVP EMMNEQAVED FVQGS---DS FFYAMPVSEV

#TcGMC_G1_{GMC-G} LSAGALQSPQ LLMLSGVGPQ AHLEEVGVEP LVDSPGVGSN LQDHVAMGGV TFLFEPSEEY QNKTCGFILP KVFSPETIND FAQRR---QG PVYWLPECEL

#AgGMC_I3_{GMC-I} MTAGALQTPQ LLMLSGVGPK EHLQEVGIPV IKDLP-VGQT LYDHIYFTGL AFVTNTT--- ----NLSLHG DNVLTLDAFL SFLQG---QG PMTVTGGVEA

#AgGMC_I4_{GMC-I} VTAGNINTAQ LLLLSGIGPR EHLQNFNLPL VSNLP-VGQS FVDSPVFNGL TFVLNET--- ----GQALLT DSRFQLRSLG DYFRG---EG PLTVPGGVEA

#DmGMC_I1_CG9522_{GMC-I} LSAGAFNSPQ LLMLSGIGPE DNLKAIGIPL IKALP-VGKR MFDHMCHFGP TFVTNTT--- ----GQTLFA A-QLGAPVAK EFLLG-RADT FLSSIGGVET

#DmGMC_I2_CG12539_{GMC-I} LSAGSFNSPQ LLMLSGIGPE DNLRGIGIPL IKALP-VGKR MFDHMCHFGP TFVTNTT--- ----GQTTFT S-RVTPAELI SFLLAGNPAT RMSSIGGVEA

#TcGMC_I5_{GMC-I} LSAGAFNSPQ ILMLSGIGPQ KHLQELGIPV LEDLP-VGQK MYDHITFLGL VFQVNES--- ----IVSDQK LLESPESFLQ LVLKN---NG PLTTLGGVEA

#TcGMC_I6_{GMC-I} LSAGVFNSPQ LLMLSGVGPE GHLHDLGIPP IVNLP-VGQN LYDHLAFLGV AYTINVT--- ----VEP-RE ALLSPLEGLN WFFRG---KG LYTSLGGVEA

#TcGMC_I7_{GMC-I} LSAGTFNSPK LLMLAGIGPR DHLAEMGIPL LEDLP-VGQN LHDHLTYPGL SFIIDKP--- ----LSLSVL HLINPKNIID FLFNG---TG PYTSLGGVGG

#AgGMC_K1_{GMC-K} LSAGPIFTPH LLLLSGIGPR AQLDALQIPV LADLP-VGAT MNLRLVSFPL HLATNRT--- -------VPY AAQKMIEAIA FLN------- ----------

#TcGMC_K2_{GMC-K} LSAGALNTPQ ILMLSGIGPK EQLEKFEIPV VHELP-VGKH LKDHIGFYGL DLLYNGT--- -------EST PDPHYDEVID YLKNG----K GPLTTTGCEV

#TcGMC_K3_{GMC-K} LAAGALNTPK ILLLSGVGPK EDCEKLHIHH VADLK-VGHN LKIRPSFVGL DFLYTAE--- -------EAQ SHDEYHDILK YLKYG----K GPLTSPGIEA

#AmGMC_L1_{GMC-L} LSAGAINSPK ILMLSGIGPA DELKKHGIPV ISDLP-VGRN LQDHVTMDGL VIALNS---- ----TSTTKD NRMKKNDICY YEKTQ---MG PLSATGTLVC

#TcGMC_L1_{GMC-L} VTCGTIMTPK VLMLSGVGPA QHLQNLGIQV IKDLP-VGYN LMDHPTIDGV MFQISNE--- ----SATLVE PEQITRDVFY YREEQ---AG PLSSTGPLQV

#AmGMC_M1_{GMC-M} LSAGSVESPK LLMLSGIGPR EHLEELGIKV IQDSK-VGYN VYDHLGFLGL SFKVKNV--- ----ATQSIK KTLKLETFLE YFFNG---NG YLSSIGGPEA

#AgGMC_Q3_{GMC-Q} VSAGAIGSPH LLMLSGIGPA KHLRLKGIQP LANLA-VGFN FQDHVAGGAL TFLINHT--- ----ETLTSK RMFTLESFME YEHQH---TG MMASTGACEA

#AgGMC_Q4_{GMC-Q} VSSGAIGSPH LLMLSGIGPA DHLRANGIKP IADLP-VGHN FQDHTAAGGL TFLVNNT--- ----QTLTYK NVFRLDNFMK YQYDK---RG PFTSTGGCEA

#AmGMC_Q7_{GMC-Q} VSAGAISSPK LLMLSGIGPA EHLREMGIEV VRDAR-VGDN LMDHIAYGSL LYDIDQR--- ----VDVIAN RLFQR-VLNN YFMDK---VG QLTSLGGTEA

#DmGMC_Q1_CG9519_{GMC-Q} LSAGAINTPQ LLMLSGVGPA KHLREMGIKP LADLA-VGYN LQDHIAP-AI SFLCN-V--- ----SSLQTS EMFRSEAMSD FLKG----RG VLRIPGGVEA

#DmGMC_Q2_CG9521_{GMC-Q} VSAGAINTPQ LLMLSGVGPA KHLREVGIKP LADLA-VGYN LQDHTAP-AV TFTTN-A--- ----TSLKFE DFSDPTLINR FNRM----EG PYGSPGGCEA

#TcGMC_Q5_{GMC-Q} VSAGAINSPQ LLMLSGIGPR KHLTQKGIPV LSNLK-VGYN LMDHIALGGL TFIINKP--- ----YSLNTE KMITTENMRQ YLNYH---KG PLSVPGGCEV

#TcGMC_Q6_{GMC-Q} VAGGAVNSPQ LLMLSGIGPK KHLTQMGIPV LSNLK-VGYN LLDHVALGGL TFRIDEP--- ----YSLKTE RVLSRESLFQ FWNYH---QG PITAPGGCEV

#AgGMC_Z1_{GMC-Z} MSAGSINTPQ ILMLSGIGPR AHLEDVGITT IQDLP-VGEN LQDHVGMGGL TFLVDKP--- ----VAILQN RLEAGSVTMN YVINE---RG PMTILGGLEG

#AmGMC_Z1_{GMC-Z} LSAGAINSAQ ILMLSGIGPK EHLRHIGIPV IKDLR-VGDN LQDHVGMGGL TFLIDKP--- ----VAIVQD RFQAAAITMH YVANG---RG PMTTLGGVEG

#DmGMC_Z1_CG9518_{GMC-Z} ISAGAINTPQ LMMLSGLGPR KHLEKHGIRV LQDLP-VGEN MQDHVGMGGL TFLVDKP--- ----VAIVQD RFNPTAVTFQ YVLRE---RG PMTTLGGVEG

#TcGMC_Z1_{GMC-Z} MSAGAINTPQ ILMLSGIGPQ PQLSKFGIPV LRDLP-VGEN LQDHVGMGGF TFLVDKP--- ----VSIVQD RFQAFPMTMQ YVMNA---KG PMTTLGGVEG

#AgGLD_{Insect_GLD-GOX} VSGGAVNSPQ ILLLSGIGPR EHLEKVGVRP IHDLPGVGKN LHNHVAYFIN FFLN------ ------DTNT APLNWATAME YLLFR---DG LMSGTGVSAV

#AmGLD_{Insect_GLD-GOX} ISGGAVNSPQ ILLNSGIGPR EELNAVGVPV IHDLPGVGKN LHNHVAYTLA FTIN------ ------DTDT TPLNWATAME YLLFR---DG LMSGTGISEV

#AmGLXr_1_{Insect_GLD-GOX} LTGGTIGSAH ILLNSGIGPK DQLTKLGMHV VKDLP-VGKN LHNHVSIGVQ FSIK------ ------DTAY EAMTMNSVNE YLETR---TG PMTSTGLTQV

#AmGLXr_2_{Insect_GLD-GOX} ISGGAVNSPQ ILLLSGIGPK EHLEAVNVSV VHDLPGVGEN LHNHVSFTLP FTIN------ ------RPNE FDLSWPSLLE YIAFT---KG PIASTGLSQL

#AmGOX_{Insect_GLD-GOX} LSAGSVNTPQ LLMLSGIGPK EHLRSLGIPV VVDLPGVGEN LHNHQSFGMD FSLN------ ------EDFY PTFNQTNVDQ YLYNQ---TG PLSSTGLAQV

#DmGLD_{Insect_GLD-GOX} LSAGAVNSPH ILLLSGVGPK DELQQVNVRT VHNLPGVGKN LHNHVTYFTN FFID------ ------DADT APLNWATAME YLLFR---DG LMSGTGISDV

#TcGLD_{Insect_GLD-GOX} VSGGAINSPQ ILLLSGIGPS QDLQQVNVPV VHNLPGVGKN LQNHVAHFVN FNIN------ ------DTNS APLNWATAME YLLFR---DG LMSGTGISEV

#TcGLXr_3_{Insect_GLD-GOX} VSAGAINSPQ ILLLSGIGPK EELDKVNIQQ VHQLPGVGKN LHNHVTFYMT YEMKK----- ------QKAV HDLDWAHALD YILNR---RG PMSSTGMSQV

#TcGLXr_4_{Insect_GLD-GOX} VSAGAINTPH LLLLSGIGPR EELDKVGIEQ VHNLPGVGQN LKNHVSFAVN FQLTK----- ------IENY NDLNWNTVRE YLTER---RG PMSSTGVTQV

#DmNinaG_CG6728_{NinaG} LCAGAYQTPQ LLMASGIGDV SALKKLGIPA QHSLPLVGHN LHDHFNLPLF VSMGVTG--- ----PTLNQN TLLNPMTLIN YLSSG---SG PLGNFGVLGN

#Tc_NinaG_like_{NinaG} LSAGVIGSPK ILMLSGIGPK KHLEKLKIAP RLDLP-VGEN LQDHVTTGLD LITLEAP--- ----PDMGLQ QMLSPWSASR YFLWG---EG PWTSPG-CES

#Tc_XM_961446_{otherBeetleGMC} LSAGAFNTPQ LLMLSGIGPG YHLQELGIEV IQDLE-VGST LRDNPTFYGV AFQTNYT--- ---------- --EPIEPLEN YIEQYFQGVG PLAIPGNNQG

#Tc_XM_961538_{otherBeetleGMC} LCAGAFGSPQ ILMLSGVGPK KHLQDVGIEV IKDLE-VGST LRDNPTFFGL NFGTNYT--- ---------- --EPVRPLKD YVLQYLEGVG PLTIPGSNQG

#Tc_XM_967481_{otherBeetleGMC} VSAGAFNSPQ LLMLSGIGPK EHLEELGIDL IEDLP-VGQN LLEHPMFSGL AFRTNFT--- ---------- --VTAE---- ---------- ----------

#Tc_XM_968249_{otherBeetleGMC} LSAGVFGTPQ ILMLSGVGPR KHLEEKGIEV IKDLE-VGST LRDNPTFYGL NYGTNYT--- ---------- --EPIRPLAD YVKEYLNGVG PLAIPGSTQG

#Ag_CG6142like_1_{CG6142} VAFVKTN--- ---------- -ISFTPSDYP DIELVMG-TG AYNNDESGTL RATIGFTDQF YHSTYGSILG --KHAFSVSP VLMRPKSRGR ISLKSTN-PF

#Ag_CG6142like_2_{CG6142} IAFVKTN--- ---------- -NSRSPEDYP DVELVLG-TG AVNNDESGSL RHTFGMTREF YDRSFGSARG --QHAFGIAP VLMRPKSRGR VWLKSRN-PF

#Am_CG6142like_3_{CG6142} LAFIDTKEDR SIRLMKKFQV NNTKFQTNVP DIELVLG-IS ALTGDISGSY RGLLGLTNEF YKEVFTGYEG --YDAFSIVP VLLQPKSRGR VTLKSSD-PF

#Dm_CG6142_{CG6142} FAFVRTP--- ---------- -SSKFAKDYP DMELVLG-AG SLSGDRFGTM RNLLGITDEF YDYMFGDLQS --KETFGLVP VLLRPKSRGR ISLRSRN-PF

#Tc_CG6142like_4_{CG6142} LAFVQTK--- ---------- -YAKIG-GYP DIELVLG-AG ALNGDVYGSL RSLLGIPRSL FERVYAPHAY --KPAFSIAP VLMRPKSRGR VVIKDGN-PL

#Cele_CHD_{CHD} GGFARSD--- ---------- ----DTVTHP DIQFHFL--- ---------- ---------P STVHDDGRTN GTCHGYQVHV GPMRSQSKGY IMLQAKD--P

#Ecol_CHD_{CHD} GGFIRSR--- ---------- ----EEFAWP NIQYHFL--- ---------- ---------P VAINYNGSNA VKEHGFQCHV GSMRSPSRGH VRIKSRD--P

#Human_CHD_{CHD} GGFIRSQ--- ---------- ----PGVPHP DIQFHFL--- ---------- ---------P SQVIDHGRVP TQQEAYQVHV GPMRGTSVGW LKLRSAN--P

#Anig_GOX_{Fungal_GOX} HELLNTK--- ---------- -----LEQWA EEAVARG--G FHNTTALLIQ YENYRDWIVN HNVAYSELFL DTAGVASFDV WDLLPFDRGY VHILDKD-PY

#Aory_GOX_{Fungal_GOX} MELLNTK--- ---------- -----LDQWA EETVRNG--G FHNVTALKIQ YENYRDWLLN EDVAFAELFL DTEGKINFDL WDLIPFTRGS VHILNGD-PY

#Pama_GOX_{Fungal_GOX} RDLLNTK--- ---------- -----LDQWA EETVARG--G FHNVTALKVQ YENYRNWLLD EDVAFAELFM DTEGKINFDL WDLIPFTRGS VHILSSD-PY

#AgGMC_A1_{GMC-A} LAFVSTK--- ---------- -YVNATDDYP DIEFHFV-SG STNSDGGNQL RKAHGLTEAF YNTVFKPINN --MDAWSIIP MLLRPHSVGT IKLRSSN-PF

#AmGMC_A1_{GMC-A} LAFVNTK--- ---------- -YVNASDDFP DIELHFV-SG STNSDGGRQI RKIHGLTKRF YDAVYGALND --MDVWSVIP MLLRPKSKGV IKLRSKD-PF

#DmGMC_A1_CG9503_{GMC-A} LAYVNTK--- ---------- -YANSSLDWP DIEFHFV-SG STNSDGGSQL RKAHGLTDAF YRAVFEPINN --RDAWSIIP MLLRPRSVGN IRLRSGN-PF

#TcGMC_A1_{GMC-A} LAFVNTK--- ---------- -YVNASDDFP DIELHFI-SG STHSDGGTQL RKAHGLTDAF YERVFGPIAD --KDAWSVIP MLLRPKSRGF IKLRSKN-PL

#AgGMC_B4_iso1_{GMC-B} TGFVNT---- ---------- --VNHSDPFP NIQYHHMYSR KR-SNIAGRW LRMMELDEPF SSSVADANNE --ADVLGAFV ILLKPKSWGR IRLQSG--QI

#AgGMC_B4_iso2_{GMC-B} GAFLNT---- ---------- --KNTSDPFP NLQILNFAFP RG-GRFSEAQ TRHFEFTDII SASVQEVDRV --TPAMYVHI TALNPKSRGR VKLSSA--NP

#AgGMC_B4_iso3_{GMC-B} IGFINT---- ---------- --LDATSPFA DIEYHFFQFE KG-SGKSVLF CDKVGYTQEI SQSMLEAATE --ADVVMAIV VLLNPKSKGR VTLATEDFNE

#AgGMC_B4_iso4_{GMC-B} LAFVNT---- ---------- --QSPAAKFP DIQYHHSLIL WK-TPDIARL TQCFGWEDYI SHQIIEQNQK --SEILMVMV TLLNPKSKGN VQLRSS--NP

#AmGMC_B10_{GMC-B} LGTVNV---- ---------- --NDPNSSYP DVQFLFVPFE RHERVQLSVF LETIGLREEI GGKLVEEIER --TSVIVVLS ILLKPRSRGM VELRST--DP

#AmGMC_B6_{GMC-B} QGFVNV---- ---------- --TDPSSRYP DVQFLVAPIH RFESHILTSV MNSFDMMDEL VTDMSRVITN --ASMVIVYP ILLKPRSRGV VRLRST--DP

#AmGMC_B7_{GMC-B} NGFVDV---- ---------- --NDPHSKYP NVQFMFVPYQ RYTNN-LLSL LQGYNMNDDI IQEMQQAVKK --MSLISICP VLIRPLSRGF VELRNT--NP

#AmGMC_B8_{GMC-B} TGFVDV---- ---------- --NDPHSKYP NVQFIFMPVQ -FLSQ-LRDY LRAFNVDNDL IKKIENDVKE --MKIIFSSA TLLKPLSRGF LELRST--NP

#AmGMC_B9_{GMC-B} VAFINP---- ---------- --VDPKSIYP EVQLLFSQIQ RYDKNGLKTL LHSYNANDEI LQIMTDVIMK --RSLIIAYA SLMRPLSRGV IELRNA--DP

#DmEO_B1_CG9504_{GMC-B} MGYINS---- ---------- ---SSPSSSR GEPDLHVVAH TLLPKGSTGS FGYLGFRPEL IQAQQDILQK --GDLLQIMG SLLRPLSHGK VSLSSK--NS

#DmGMC_B2_CG9509_{GMC-B} VGFINTN--- ---------- --ASSDGAYP DTENHHMFFQ RAHHASLELF TKGLSIQDQY TEVLQEYLKD --SHLLCVFV LLSHPAARGE LRLKST--DP

#DmGMC_B3_CG9512_{GMC-B} TGFINT---- ---------- --TSIEGPNP DIQTTN-FFS LMQSPELKGY VAATGFNDRV AKSILSANQE --TNTYITYL LHLKPFSAGS LTLQSA--NY

#TcGMC_B5_{GMC-B} LGFVNS---- ---------- ---RNDSNYP NLQFHHILYI KGDNYLLPEI LRVTGLGPEV ASIELQANQK --SPMFKIAP TLLNPKSRGN ILLKSK--NP

#AgGMC_D1_{GMC-D} VGFISTK--- ---------- -YANQTDDWP DIEFMLT-SA STPSDGGDQV KKAHGLKDEF YEDMFSSINN --QDVFGVFP MMLRPKSRGF IRLQSRN-PL

#AmGMC_D1_{GMC-D} VGFISTK--- ---------- -YANRSDDWP DIEFMLT-SS SVNSDGGTHV KNAHGLTDEF YNEVFESINR --RDVFSVFP MLLRPRSRGF LKLRSSN-PL

#DmGMC_D1_CG9514_{GMC-D} VAFINTK--- ---------- -YANASDDWP DMNFMMT-SA SVMSDGGSQV KTAHGLTDEF YQEVFGEVNN --RDVFGVFP MMLRPKSRGY IKLASKN-PL

#TcGMC_D1_{GMC-D} VGFIPTK--- ---------- -YTNQSDDWP DIEFMIT-ST STPADGGTQV KHAHGLTDEF YNEYFSEINY --KDTFAVFP MLLRPKSRGE IKLRSKN-PL

#AgGMC_E1_{GMC-E} VAFVNTK--- ---------- -YADPSGKWP DIQFHFG-PS SVNSDGGQNI RKILNLRDGF YNTVYKPIQN --AETWTILP LLLRPKSTGW VRLRSKN-PF

#AmGMC_E1_{GMC-E} VAFVNTR--- ---------- -YADKMDDYP DIQFHFL-PS SINSDG-EQI KKILGLRESV YNTMYKPLTG --ADTWSILP LLLRPKSSGW IRLKSRN-PL

#DmGMC_E1_CG9517_{GMC-E} VAFLNTK--- ---------- -YQDPSVDWP DVQFHFC-PS SINSDGGEQI RKILNLRDGF YNTVYKPLQH --SETWSILP LLLRPKSTGW VRLNSRN-PQ

#TcGMC_E1_{GMC-E} LAFVNTK--- ---------- -YAPKSGSWP DIQFHFA-PS SINSDG-EQV KKITGLRDSV YNTVYKPLKN --AETWTILP LLLRPRSTGW VRLKSKD-PN

#AgGMC_G2_{GMC-G} MGFLNTK--- ---------- -YQDPELDWP DVELFLA-SL SDLTDGGRFG KRGSGISNNY YAQVYEEQVY --QNSYMVIP MLSRPLSTGW LELASKN-PH

#AgGMC_G3_{GMC-G} MGFINTK--- ---------- -YNKPGSRRG DVQIFMS-AQ SDISDGGTEG QAGAGLTYEY YARNFESWVY --HDSFLIMP LLMHPESRGW LELPSAN-PM

#AmGMC_G1_{GMC-G} MAFINTK--- ---------- -YANESADYP DIQLFLS-SM ADNTDGGLFG KRDCNVMDNF YERLYENILY --QDSYMIIP LLLRPKSRGY IKLRTRH-IY

#DmGMC_G1_CG12398_{GMC-G} MGFFSTR--- ---------- -YQDPRLDWP DVQIFMG-SY GYGADGGMIG RRGAAITLDN YAEAFEPVLY --QDSFVIAP LLMRPRSRGY LQLRSAD-PK

#TcGMC_G1_{GMC-G} IGFVKTK--- ---------- -YEDQDDDWP DIQYFVT-AY ADNTDGGLFG KKAAGLTDEF YSAVYEEVLY --KDAFNVII LLLRPKSRGR LFLKDAN-IN

#AgGMC_I3_{GMC-I} VAFIRNTTN- ---------P ESAATPTVLP NIEYILT-GG SQAADHGSGI RNGFRLTDTI YS-IYKPLEA NERDAMTVNI VLLHPKSKGY MRLKSCN-PL

#AgGMC_I4_{GMC-I} ISFVR----- ---------T ENATTEPGVP NIAIVFS-TG SLVSDGGLGL RKGKRIKTAI YNKVYRPLET LRNDQWTASV VLLHPESRGH LKLRSIN-PY

#DmGMC_I1_CG9522_{GMC-I} LTFIK----- ---------V PSGKSPATQP DVELIQV-AG SLASDDGTAL AKGANFKPEI YEKMYKNLTL RQQDHFSFLI MHFKPASVGR LWLHNRN-PL

#DmGMC_I2_CG12539_{GMC-I} LAFLK----- ---------T QRSNLPNDWP DIELIMV-TG SLASDEGTGL KLGANFKDEI YDRMYRELAQ AQQDHFTLLI MQFHPKSVGR LWLKDRN-PL

#TcGMC_I5_{GMC-I} LLYFKTN--- ---------- -VSKGPAPYP DMELIFI-SG SMNTDLGKYY RKTFRITDEV YNTVWKPLEN --KYTFSVLP MLVHPESYGH LELKSTN-PF

#TcGMC_I6_{GMC-I} IAYINT---- ---------- -GSLPQANYP DIELIFVGTG TLQSDFGLVV AKEIRLKRSI YDTVYKPIEN --TPSWAIFP MLLHPQSKGH LQLKSTN-PH

#TcGMC_I7_{GMC-I} IGYIKTK--- ---------- -ESLEVEDIP DIELLFL-DG SLSTDYGLWN RRWMNIRDDV YYPVYGPTHN --IPTWTIFP MLLHPKSTGY LKLKSRN-PR

#AgGMC_K1_{GMC-K} ---------- ---------- -TTKQNNTDP THEILFQ--- ---------- ---------- ----YEPRGT --LEYFSLGL IHLRPASRGF VQLNATN-PS

#TcGMC_K2_{GMC-K} VGYLQTE--- ---------- -ASKDQIKYP DIELFFSSRK VNAKP----S TNPFRLKPEI LDSLYKPIDG --KKIVNIGV MLTHPKSTGT VTLKDKD-PL

#TcGMC_K3_{GMC-K} LAFLKTN--- ---------- -ISKSPLTYP DIELKFLSRY HPQQD----L YSWMKPTPKH YDSLWKPLEA --HNCLKIIV TLNHPKSSGI VKLHTSN-PL

#AmGMC_L1_{GMC-L} GAFLQTA--- ---------- --FEHEHGLP DIQYAFD--- ------ASNQ MDFLNDPAEF GETRVEPLSY --YDAINIRP ILLSPRSKGF LLLNDTD-PL

#TcGMC_L1_{GMC-L} NTFVQTK--- ---------- --YELEPGRP DIQYSID--- ------TANV VDYVTDLILA STTKVSPLSY --YNGFIIRP ILLNPVSRGV IKLNSTD-PI

#AmGMC_M1_{GMC-M} IAFVR----- ---------- -TKYANDNRP DLELLFI-SA SLNSD-GGIL GKAMSVRKDV YEAVFESLGN --NETWTIWP IVQFPKSVGR ISLKSKN-PF

#AgGMC_Q3_{GMC-Q} ISFHDTTQPP ---------- -NRANEAGWP DLELLLI-GG THAAD--RIY ESNFNYKPET FNALFGDIER RGLEGYTVFP MILRPRSKGR IRLASAD-PF

#AgGMC_Q4_{GMC-Q} IAFYDSERP- ---------- ---GDPDGWP DYELLHI-GG TIGAD--PTY EVNFNYKHKT FQTLFGEIQR RNYDGFTVFP LIMRPRSKGR ISLNGSS-PF

#AmGMC_Q7_{GMC-Q} IAFIDVDDP- ---------- ----REREVP NVELLFL-GT SIYSV--NTL GDNFGLNEEI STKFTSYRNR ---RALSVFP ILLQPKSRGR IRLRSRD-AD

#DmGMC_Q1_CG9519_{GMC-Q} ISFYALDDA- ---------- ---RNPDAWA DMELFVV-GG GLQTN--LAL RLALGIQSNI YETMFGELER QSANGFLIFP MILRAKSRGR IKLKSRN-PE

#DmGMC_Q2_CG9521_{GMC-Q} IAFWDLDHE- ---------- ---RDEDGWP DIELFLV-GG SMSSN--PAI SRAFGLKKSI YDTLFAEIED KSLNAFMIFP MILRPKSRGR IMLKSSD-PF

#TcGMC_Q5_{GMC-Q} LVFHDLKNP- ---------- ---TDPDGYP DIELLFQ-GG SIVSD--PLL RKDFGITNEL YDAVYKPIED --LDTFMVFP MLMRPKSKGR IMLKNNN-YR

#TcGMC_Q6_{GMC-Q} VVFHDLKDP- ---------- ---TNPDGYP DIELVFL-GA SLSLD--PLL QKNLAISDYV YKTVYTPIER --FDSFMVFP MILRPQSRGR IALRDNN-YK

#AgGMC_Z1_{GMC-Z} IAFVNTP--- ---------- -FANVTDDWP DIQFHMA-PA SLNSDGGARV KKVLGLREDL YKEVFHPIED --TYSWTIMP LLLRPRSRGW VRLKSNN-PF

#AmGMC_Z1_{GMC-Z} YAFVNTK--- ---------- -YANRSIDYP DIQLHMA-PA SISSDAGAQV RKVLGITDEV YDTVFKPISN --KDAWTIMP LLLRPKSRGT VRLRSSN-PF

#DmGMC_Z1_CG9518_{GMC-Z} LAFVHTP--- ---------- -YSNRSLDWP DIQFHMA-PA SINSDNGARV KKVLGLKESV YQEVYHPIAN --KDSWTIMP LLLRPRSRGS VKLRSAN-PF

#TcGMC_Z1_{GMC-Z} LAFVNTK--- ---------- -YGNR--SWP DVQFHMA-PA SINSDAGVRV RKVLGLTDHL YNTVYRPIAN --KDVFTLMP LLLRPKSRGW IRLQSKN-PF

#AgGLD_{Insect_GLD-GOX} TAKISSK--- ---------- -YAE-RPDDP DLQFYFG--- -------GFL ADCAKTGQVG ------ELLS NDSRSVQIFP AVLHPKSRGY IELKSND-PL

#AmGLD_{Insect_GLD-GOX} TAMINTK--- ---------- -YANPKDDHP DVQLIFG--- -------GYL ADCAETGMVG ------ETKG N-NRTIYIIP TYLHPKSRGY LRLRNND-PL

#AmGLXr_1_{Insect_GLD-GOX} TAFFESS--- ---------- -YAV--TGIP DIQVFFD--- -------GFA PRCPRTGLEF ECLNGALGLC PERRQINVRP TALTAASKGY LKLRSSD-PL

#AmGLXr_2_{Insect_GLD-GOX} TGIVSSI--- ---------- -YTS--EDDP DLQIFFG--- -------GYQ AACATTGQLG ------ALMD GGGRHVSISP TNLHPRSRGS LRLASND-PF

#AmGOX_{Insect_GLD-GOX} TGIWHSN--- ---------- -LTT--PDDP DIQIFFA--- -------GYQ AICKPKLKIA ------DLSA HDKQAVRMSA LNVQPTSKGR ITLNSKD-PL

#DmGLD_{Insect_GLD-GOX} TAKLATR--- ---------- -YAD-SPERP DLQLYFG--- -------GYL ASCARTGQVG ------ELLS NNSRSIQIFP AVLNPRSRGF IGLRSAD-PL

#TcGLD_{Insect_GLD-GOX} TGFINTK--- ---------- -YNDPRLEHP DIQLFFG--- -------GFL ANCARTGQVG ------ERVD NGTRQIQMIP TVLHPKSRGV LKLRDNN-PL

#TcGLXr_3_{Insect_GLD-GOX} TARINSK--- ---------- -FADPSGTHP DLQIFFA--- -------GYL ANCAASGEVR AAKD--PEHP DAPRHLTISP VVLHPKSRGH IGLKSNN-PL

#TcGLXr_4_{Insect_GLD-GOX} AARISSK--- ---------- -YANPDGKNP DLQFFFS--- -------GFL AHCSLSGGVK EPED--PTNP TAAKSFTIRP TFLRPRSRGF IGLNSRD-PK

#DmNinaG_CG6728_{NinaG} VVSYGGLG-- ---------- -------APP YGITFFG--- -AGAIDESAL MSISNFKGPA FRALFPRYYN SSQEGFVVIS SCLQPKSRGS VGLLNRH--M

#Tc_NinaG_like_{NinaG} VGFFNSE--- ---------- -----DEKIP ELQFMIL-PY GAAIDGGSYL RGLVGIGERL WEGYFRRVNG ---STMTVLP VVLHPKSRGT VRLKSKD--P

#Tc_XM_961446_{otherBeetleGMC} VGFYESS--- ---------- --YTRGTGIP DLEFMFI--- -PAVASTILQ QRAFRLTDQT YNDVYQFQDV G--STFGVYV IVLHSKSVGT VRLRSRD-PF

#Tc_XM_961538_{otherBeetleGMC} VGFYESS--- ---------- --YTKGTGIP EIELMFI--- -PANATSNLS QRSFGLTDET YEDVWKYANI P--QTFLFYV VDLHSQSVGT VRLKSKN-PF

#Tc_XM_967481_{otherBeetleGMC} ---------- ---------- ---SPGT-VP PIEYIFL--- -PQTGTPSAF D-MFNFNQEL ENSYLAKINS S--TDFNIFV VLLHQKSKGQ IRLKSKN-PT

#Tc_XM_968249_{otherBeetleGMC} VGFYESS--- ---------- --YSKGTGIP DIELMIA--- -VANATDQLT QRYFSLTDQT YEDVWKYNNI P--QTFIFHV VNLHAQSSGS VRLKSKN-PF

#Ag_CG6142like_1_{CG6142} H-WPRMEGNF FA--DYDDLL VLREGVKLTV DLIE-SRSFR DVGARLHSTP FYGCEQHRFR S--------- DEYWECAIRR IGSTLQHQCG TCKMGPVT--

#Ag_CG6142like_2_{CG6142} H-WPHMEGNF FD--HPDDLA TMVEGIKLAV RIGE-SDSFA SYGARLLGTP FYGCEAHPFR S--------- DDYWRCCLRQ VGASIQHQSG TCKMGPAS--

#Am_CG6142like_3_{CG6142} D-RPIFETNY YD--HEDDLR TML--IFQAI EVAS-TKAFK RFNATLLPVA FPGCKHVPFG T--------- DPYWACVARQ VTTTLGHFVG TCKMGPRR--

#Dm_CG6142_{CG6142} H-WPRMEPNF MQ--HPDDVR AMIEGIEMIL KLSR-SKPMA KMGTRFHDRP FPGCENLKFA S--------- EAYWKCCLRR YGSSLQHQSG TCKMGPAT--

#Tc_CG6142like_4_{CG6142} H-WPKLIPNY FE--NEEDVK TMVEGIKMAI TITQ-SRHFQ KYNITMITTP FPGCETVPFG S--------- DEYWACAVRH VATTLGHQVG TCKMGPPS--

#Cele_CHD_{CHD} RRAPIINPNY ME--EDSDWR EFRKCIRVSR ELFASKAFDE FRGKELAPGP DCQSD----- ---------- -ADIDRFVKE KAASAYHPSC TCKMGSE---

#Ecol_CHD_{CHD} HQHPAILFNY MS--HEQDWQ EFRDAIRITR EIMHQPALDQ YRGREISPGV ECQTD----- ---------- -EQLDEFVRN HAETAFHPCG TCKMG-----

#Human_CHD_{CHD} QDHPVIQPNY LS--TETDIE DFRLCVKLTR EIFAQEALAP FRGKELQPGS HIQSD----- ---------- -KEIDAFVRA KADSAYHPSC TCKMGQP---

#Anig_GOX_{Fungal_GOX} LHHFAYDPQY FLN------E LDLLGQAAAT QLARNISNSG AMQTYSLGRL LPGDNLAYDA D--------- LSAWTEYIPY HFRPNYHDVG TCSMMPKEMG

#Aory_GOX_{Fungal_GOX} LHRYANDPKF FLN------E FDILGQAAAT KLARELSNTG EMKKYFAGEI IPGDNLAYDA S--------- LEQWADYVKE NFRANWHAVS SCSMMSREMG

#Pama_GOX_{Fungal_GOX} LWQFANDPKF FLN------E FDLLGQAAAS KLARDLTSQG AMKEYFAGET LPGYNLVQNA T--------- LSQWSDYVLQ NFRPNWHAVS SCSMMSRELG

#AgGMC_A1_{GMC-A} D-YPYIYPNY LH--DDRDMR TLVEGVKIAY ALS-RTQTMQ KYQSTLSAYK FPGCAHIQMF T--------- DLYWECMIRH YTCTIYHPVG TCKMGPYW--

#AmGMC_A1_{GMC-A} A-HPLIYPNY FN--EPEDIA TLVEGVKIAV ALS-RTQAFR RFGSEVNSKQ FPGCKNIPMY S--------- DPYWECMIRH YTVTVYHPVG TCKMGPYW--

#DmGMC_A1_CG9503_{GMC-A} D-YPYIFPNY LT--DDFDMK TLIEGVKIAV ALS-RTKAMQ RFGSRISSIR WPGCEQVPLF T--------- DAFWECMVRR YTSTIYHPVG TCKMGPYW--

#TcGMC_A1_{GMC-A} D-YPLIYPNY FK--DDFDMK TLIEGAKLSV ALS-QTPAFK YYKSTLH--K FPDCAGFKDY S--------- DEFYECMIRL YTVTIYHPVG TCKMGPYW--

#AgGMC_B4_iso1_{GMC-B} EQKPKIDAGY LT--HRQDIE TLIEGIRIHQ DIMT-TDAAK PMEPEPVRIE LPSCQDEL-Y DS-------- NAYWECYIRE LTLTLYHPVG TAKMGP----

#AgGMC_B4_iso2_{GMC-B} RVHPIIEANY FE--HTDDLN VLVQGIRLQQ RLLQ-TEAFR SAGAALHRID IPGCQELV-Y DT-------- DAYWECYVRQ LTVTTYHPVG TAKMGP----

#AgGMC_B4_iso3_{GMC-B} FNPPRIQSGY LE--AKEDVE AVLRGIRYIN KIVD-TPTFR EHEGELHRMK LSECDELV-Y DS-------- DDYWECYARY TTLTLYHPVG TAKMGP----

#AgGMC_B4_iso4_{GMC-B} YDAPIINANY LD--DQRDVK TIIRGIRFFR KLLD-TENFG YHELKEFHLK IEECDRLE-Y ES-------- DSYWECYARY MSSTIYHPTG TAKMGP----

#AmGMC_B10_{GMC-B} TDPVKIYANY LV--EAEDTR TLVKSVDKMK EILD-TDALT GNGMRFNRLD VPGCRRFE-P DT-------- EQYWECSVRH VSVSYYHSCG TSRMGP----

#AmGMC_B6_{GMC-B} ADPVKIHANY FA--EKADLE TLLKSVDVIK ALVN-TETLK RHGMRLHHFD IPGCRHAK-P DT-------- EEYWECNVRH VTTSLFHACG TARMGP----

#AmGMC_B7_{GMC-B} ADPVKIYANY FA--EKEDFN NLLKSVNIVK AFLN-TDILK KYNMTLYYPN ISGCQHTE-P GT-------- DEYWECNLEH LSTTLFHPCG TAMMGP----

#AmGMC_B8_{GMC-B} ADPVKIYPNY FA--EKEDFN TLLKSVNVIK NLLN-TKVLK KYNMKLFYPD IPGCRHTK-P GT-------- DEYWECNLKY LSTTLFHPCG TAMMGP----

#AmGMC_B9_{GMC-B} AEQVKIYSNY YT--VPDDWK RLAKAVPTLK SLLN-TTILQ KYKANFHTYD VPQCRNLT-A DT-------- EEYYECNIRH TTGTNFHACC TNRMGP----

#DmEO_B1_CG9504_{GMC-B} ADQAKIENHY GE--AVEDQQ TLLRYVRYIQ KLSK-TRPFR RCGLRLWKPP LHECDTLA-A DS-------- DDYWLCYIRY FYVGAWHSVG TCRMAPRKGV

#DmGMC_B2_CG9509_{GMC-B} KVPPILTSNY LT--ESEDVA TLMRGIRYIE SLEQ-TKAFQ DHLAEIARIP IKECDQIENY RS-------- EEYWRCYAKY FTVTCYHQSG TVKMGP----

#DmGMC_B3_CG9512_{GMC-B} LDAPIIDPGY MT--DERDVD TYIRALNIYK NLPN-TKAFS EREAALHKLD LEACNGLT-Y QS-------- DDYWRCYIRH MTTTVYHPVG TTRMGP----

#TcGMC_B5_{GMC-B} NDKPLIFANY LD--DPLDVE TLLEGIKFGL KQIE-SDPFA KFKPKLIDYN LKECQKFE-Y KS-------- DDYWRCAIRW LTTTLYHPVG TCKMGP----

#AgGMC_D1_{GMC-D} R-YPLLYHNY LT--HPDDVG VLREGVKAAI AFG-ETQAMK RFGARFHSKQ VPNCRHLPEF T--------- DEYWDCAIRQ YTMTIYHMSG TAKMGPPD--

#AmGMC_D1_{GMC-D} D-YPLMYHNY LT--DPYDID VLREGVKAAI AFG-QTSSMR RFGARFHSHP VPNCKRIPLY T--------- DEYWNCAIRQ YTMTIYHMSC TAKMGPRT--

#DmGMC_D1_CG9514_{GMC-D} R-YPLLYHNY LT--HPDDVN VLREGVKAAV AMG-ETQAMK RFGARYWNKP VPNCKHLTLY T--------- DDYWNCFIRQ YTMTIYHMSG TAKMGPPT--

#TcGMC_D1_{GMC-D} D-YPLLQPNY LT--DLHDVW VMREGAKAAV AFA-QTESMK RFGTRFYSKP LPNCKHLPLF T--------- DEYWDCAVRQ YTLSIYHYSC TAKMGPAE--

#AgGMC_E1_{GMC-E} V-QPSIEPNY FA--HEEDVA VLVEGIKIAI NVS-YTQAFQ RFNSRPHAIP LPGCRHLPFM S--------- DAYWACTIKQ FTFTIYHPAG TAKMGPSW--

#AmGMC_E1_{GMC-E} V-YPDINPNY FT--RKEDVD VLVDGIRIAM SVS-NTTAFR RFGSRPHTIR MPGCHRYPFD T--------- YDYWECAIRH FTFTIYHPVG TCKMGPRS--

#DmGMC_E1_CG9517_{GMC-E} H-QPKIIPNY FA--HQEDID VLVEGIKLAI NVS-NTQAFQ RFGSRLHNIP LPGCRHLPFQ S--------- NEYWACCIKE FTFTIYHPAG TCRMGPSW--

#TcGMC_E1_{GMC-E} I-YPDINPNY FT--HKEDIL TLTEGIRIAL NVS-NTQSFQ RFNSRPHKIP FPKCRQYDWD S--------- DEYWECSLRH FTFTIYHPTS TAKMGPAS--

#AgGMC_G2_{GMC-G} D-HIRIHPNY FD--NPKDMM VLIEGLKFAE ALA-NTTAMR NINATLLDYS RSACRASNFP NK-------- DDFYTCLVRH YTQTIYHPCG TAKMGPVT--

#AgGMC_G3_{GMC-G} D-KIKIYPNY FA--VERDLD ILVEGLKFGV RVA-ETSVMR KINATFI-YD AEHGDTCNGQ VG-------- DAFFKCLIQH YSQTIYHPSG TAKMGPAT--

#AmGMC_G1_{GMC-G} D-QPIIVPNY FD--DPHDLD VLAEGAKFIY EMS-KTATMK RLKARPNPNK LSECSSFEYP S--------- IDYWRCYARY YTMTIYHPSG TCKMGPAS--

#DmGMC_G1_CG12398_{GMC-G} V-HPLIHANY YD--DPHDMA VMVEGLKLAH RLT-QTPVMQ SLNATMNIYE WRNCPEVEYL S--------- DAFWECLARF YSQTIYHPVG TCKMAPAS--

#TcGMC_G1_{GMC-G} S-HVVIYPNY FD--DPQDMQ VLIEGAKIAY DLSTKTPTMS QYKTTFNHFK IPGCHHLPFL S--------- DEYWACQASH YTLTIYHPVG TAKMGPPN--

#AgGMC_I3_{GMC-I} H-WPRFYSNM LK--EQEDVE TILQGIRSAL PLMD-TRAAR RYGAKLYDVP LPNCASFRFG T--------- DDYWRCAIRT QTTSIHHQIA TCKMGPPS--

#AgGMC_I4_{GMC-I} S-ALKIYPGY FG--ADRDVE TMLEGIKEAV RISK-SPAMR RYDARVLGIP LPNCEQWDQR E--------- DEYWRCAIRT LSSTAYQQLG SCRMGPAG--

#DmGMC_I1_CG9522_{GMC-I} E-WPRIDPKY FS--APSDVE NLLEGIKEAL RISK-MPAMQ AIGTRLLDKP VPGCENYEFA S--------- DDYWRCSIRT LSYTLHHQVA TCRMGAES--

#DmGMC_I2_CG12539_{GMC-I} G-WPKIDPKY FV--AEEDVE YLLDGIKASL RIIE-MPAMQ RIGARLLKRT VPGCEGHQFA S--------- DDYWRCSIRT LSYTLHHQVA TCRMGAES--

#TcGMC_I5_{GMC-I} H-WPRFYGNY FTDRDNTDIK TFIAAIREVQ RIAK-MPTWQ KYGVRQVTTK IPGCQNFVFD S--------- DDYWECALRH VTTTLHHQVA TCKMGPKT--

#TcGMC_I6_{GMC-I} D-PPILHGNC FTDPGDQDIK TLLASIRYIQ KLAQ-TPSFQ KFGSKLHDIP LPTCQKHVFD S--------- DDYWLCAIKS LSTTLHHQVG TCRMGHWD--

#TcGMC_I7_{GMC-I} D-YPLLYGNY FTDPAQQDLK TMLAAIRYIQ KLAN-TRPFQ EMGTRMNPNP IPVCAHLIFD S--------- DAYWMCAIRA ISVTLHHQVG TAKMGPKD--

#AgGMC_K1_{GMC-K} R-NPVVYTNF FSAPN--DME EILSGITECL KIVH-SEEFT KLGLQSRKLI VPPCDKLRYG T--------- DEYWRCVVRH VGHAADQPYG TCPMGRQD--

#TcGMC_K2_{GMC-K} H-HPLVDPNQ LSDPEDHDLN TLLHGIRKAL AFAG-TETFK KLHLEVNEHP VAGCEEHKWG T--------- DEYWKCAIKH LSISLRHVSG TAKMGPGN--

#TcGMC_K3_{GMC-K} R-PPIIEPHF LSDEDEKDYH TILAGIKKAL KFSH-TEAFK KIGIKLNHHG VHGCEETEFG T--------- EAYWECAIKY LVVATEDVSG TARMGPES--

#AmGMC_L1_{GMC-L} WGPPSIYPAY FT--AYPDAD VMVEGIETAL KLFH-TTWFR EYGFRLIDTP LPSCKRFIFG T--------- REYWKCAMME YTATIYHPVG TCKMGPDW--

#TcGMC_L1_{GMC-L} YGYPIIYANT FN--EQIDAL TMVEGIKQSL NLLK-TRAMQ RMGVSLITTP VAACDGYSFG T--------- EDYWLCLVRS YTSTMYHYAG TCKMGPKH--

#AmGMC_M1_{GMC-M} D-PPRLEPNF FS--DPLDVE IILEGIKIAV NISN-SKIFQ RYESALHRGI IPGCRIFEFG S--------- DDYWRCAIRH LPSMMNHEVG SVKMGPRS--

#AgGMC_Q3_{GMC-Q} E-HPIIQPNY LG--DPYDLE VSVRGIRKAI ELTK-TNTLK SFDARLLDIP IPGCEQHRFD T--------- DDYWKCFTRH VTYTIYHHVG TCKMGPAS--

#AgGMC_Q4_{GMC-Q} Q-YPIIEPNY FD--DPYDLD ISVRAIRKAI ELSR-TGAMQ RYNARLLDIP MPGCEHYRFD S--------- DDYWKCFSRH ATFTIYHHVG TCKMGPRK--

#AmGMC_Q7_{GMC-Q} D-KPRIFPNY MS--EPEDVK GLIKGIKAAN KFLLGTKAFE RLNTRLNNQT VPECEKFPFD S--------- DDYWECNLRL IPITIYHYSG TCKMGPES--

#DmGMC_Q1_CG9519_{GMC-Q} E-HPRIYANY FA--NPYDMN ITVRGIEQAV SLLD-MPAFK AIGAHLLEKR IPNCAKYKWK S--------- SAYWACYARH FTFTIYHYSG TAKMGPRS--

#DmGMC_Q2_CG9521_{GMC-Q} K-YPLIHANY FA--HPYDVD ISVRGLLKAI SLME-QRGME KINARLWEKK IPTCKQHPYK S--------- WAYWACYVRH FTFTIYHYSG TAKMGPKS--

#TcGMC_Q5_{GMC-Q} A-KPYIYPNY FA--YDEDMD TIMGGVHLIL NITQ-QPALQ ALGARLHDIP IPQCAKYGFA S--------- DDYFKCMARH FTFTIYHQSG TCKMGPPS--

#TcGMC_Q6_{GMC-Q} S-KPRIFPNY FH--VKEDME TIIGGVRLTL NITA-QQPMR KIGTRLHDIP IPQCAHLEFA S--------- DGYFECMARH LTFTIYHHCG TCKMGPRS--

#AgGMC_Z1_{GMC-Z} H-YPLMNPNY FE--DPFDAA TLVEGAKIAL RVG-DAKVFK QFGNRLYRKP LPNCKQHKFL S--------- DEYLDCQVRT ISMTIYHPVG TAKMGPHW--

#AmGMC_Z1_{GMC-Z} H-SPLINANY FS--DPIDIA TLVEGAKIAM RIN-EAKVFK QFGSRVHRIK VPGCKHLNFA S--------- DAYWECHIRH ISMTIYHPVG TAKMGPSS--

#DmGMC_Z1_CG9518_{GMC-Z} H-YPLINANY FD--DPLDAK TLVEGAKIAL RVA-EAQVFK QFGSRLWRKP LPNCKQHKFL S--------- DAYLECHVRT ISMTIYHPCG TAKMGPAW--

#TcGMC_Z1_{GMC-Z} V-PPVINANY FD--DPIDIK VLVEGAKMAI KIG-EAQAFK QFGARVHRIK FPNCRDFEFG S--------- DEYLECHIRT ISMTIYHPVG TCKMGPSW--

#AgGLD_{Insect_GLD-GOX} E-HPKIVVNY LK--EDHDVK VLVEGIKFAV RLSETDALQ- AYGMDLDRTP VKACQ-DKDF GS-------- QEYWECAVRQ NTGAENHQAG SCKMGPTS--

#AmGLD_{Insect_GLD-GOX} S-KPLIYPKY LS--HPDDVA GLIEAIKFSI RLSETEALS- RYGFQLDRTP VKNCE-HLEF GC-------- DAYWECAVKH DTAPENHQAG SCKMGPPD--

#AmGLXr_1_{Insect_GLD-GOX} A-PPLIYPNY FV--DTKDLK VLVEGIKKSI QLVDTQALK- QWDFRLDTVV HPMCT-DYHF GS-------- DAYWECYVRA ATGPENHQSG TCKMGAYD--

#AmGLXr_2_{Insect_GLD-GOX} A-KPVIHGNY LS--DPMDEA VLLHGIRIAL SLSNTSALA- RYNMTLANLP LPACS-QHTY LS-------- DDYWRCAMRQ DTGPENHQAG SCKMGPVS--

#AmGOX_{Insect_GLD-GOX} D-PPVIWSND LA--TEHDRS VMIQAIRVVQ KLVNTTVMR- DLGVEFQKIE LKQCD-EFVE DS-------- DDYWNCVIQY NTRAENHQTG TAKMGPSY--

#DmGLD_{Insect_GLD-GOX} E-PPRIVANY LT--HERDVK TLVEGIKFVI RLSQTTPLK- QYGMRLDKTV VKGCE-AHAF GS-------- DAYWECAVRQ NTGPENHQAG SCKMGPSH--

#TcGLD_{Insect_GLD-GOX} S-TPLIYANY FT--HPNDVK VITEGIKFAM KLSETKALK- RYGFQLDRTP VQGCE-SLTF GT-------- DPYWDCAVKR QTGPENHQAG SCKMGPSS--

#TcGLXr_3_{Insect_GLD-GOX} D-PPLMYANY LS--EPEDVA TLVEGIRVTQ RLANTSVLQN KYGLTLMRDE YGDCEKKFTY DS-------- DDFWQCAARY YTGPENHQAG SCKMGPAS--

#TcGLXr_4_{Insect_GLD-GOX} E-PPLMQPNY LT--DEEDVK RMVAGIRIAQ NLANTTILTT KYGIQMVNTD YGDCSRNYTF DS-------- DEFWACALRY DTGPENHQSC SCKMGPAS--

#DmNinaG_CG6728_{NinaG} RRNPLIDPNY LS--SEEDVA CTISAIRSAV ELVN-STAFA ALHPRIHWPR VQECSNFGPF ERDFFDNRPS DQYLECLMRH VGLGSHHPGG TCALG-----

#Tc_NinaG_like_{NinaG} RTPPLIDPNY LA--EGYDVD ILLEGIELVK EFLE-TPPMR RLGAKLNAVK FPGCEGLEFD T--------- RPYWVCYVRH FTLSSYHPVG TCALGR----

#Tc_XM_961446_{otherBeetleGMC} Q-FPLIDANF LSDPENKDIN VLYEGVQLLM QMA-QTRAFR SMDATLAGGQ LSACSQYEFL S--------- REYWYCAIRQ LTINVYHPLG TCPMGRDP--

#Tc_XM_961538_{otherBeetleGMC} E-YPLIDSRF LSDPEDRDIN TLYEGVQLAL KLT-QTRPFK AINATLQGGP LRACKHFPYL S--------- KPYWYCALRQ LTINLYHPLG TCPMGKDP--

#Tc_XM_967481_{otherBeetleGMC} D-FPEIDLNL FEEQE--DVD TFIDGINFVI KLT-ETQAFR DVNATLID-- IPICQEYEKY S--------- RDFWECAIRH MSMTLYHPCG TTAMGPN---

#Tc_XM_968249_{otherBeetleGMC} E-YPVINSNF LSDPESRDIN TLYEGIQICL KMG-ETKAMK AINATLQGGP LRACKRYQYL S--------- KDYWYCVLRQ ITVNLYHPLG SCPMGKDP--

#Ag_CG6142like_1_{CG6142} ---DPEAVVN PQLQ-VYGIK GLRVVDASII PTIPASHTNA VVFMIGEKAA DM-------- ---------- ---------- ---------- ----------

#Ag_CG6142like_2_{CG6142} ---DPDAVVD PELR-VHGVG GLRVVDASIF PVIPAAHTNG VVIMVGEKAA DMVKDYWNNH IP-------- ---------- ---------- ----------

#Am_CG6142like_3_{CG6142} ---N-SGVVD HRLR-VHGIN GLRVVDASII PTIVTGHTNA VAYMIAEKAA DMIKEDWKVL NTEFDRTFRK N--------- ---------- ----------

#Dm_CG6142_{CG6142} ---DNTSVVD AQLR-IHGIR GLRVVDASVL PNVPAGHTNA IVIMVAEKAG DMIKDAWRMP ITPLSS---- ---------- ---------- ----------

#Tc_CG6142like_4_{CG6142} ---DPDAVVD ERLR-VYGIK GLRVVDGSIM PNVVAGHTNA VIMMIGEKAS DMIKQEWARK ---------- ---------- ---------- ----------

#Cele_CHD_{CHD} --NDKMAVVN PETMGVYGTE NLKVVDASVM PSIVSGNLNA PVIMMAERAA DLIKHKKQLL PPSDANVWHH N--------- ---------- ----------

#Ecol_CHD_{CHD} --YDEMSVVD GEGR-VHGLE GLRVVDASIM PQIITGNLNA TTIMIGEKIA DMIRGQEALP R-STAGYFVA NGMPVRAKK- ---------- ----------

#Human_CHD_{CHD} --SDPTAVVD PQTR-VLGVE NLRVVDASIM PSMVSGNLNA PTIMIAEKAA DIIKGQPALW D-KDVPVYKP RTLATQR--- ---------- ----------

#Anig_GOX_{Fungal_GOX} ------SVVD NAAR-VYGVR GLRVIDGSIP PTQMSSHVMT VFYAMALKIS DAILEDYASM Q--------- ---------- ---------- ----------

#Aory_GOX_{Fungal_GOX} ------GVVD SAAR-VYDVE NLRIVDGSIP PTQVSSHVMT IFYGMALKVA DAILADYSKN ---------- ---------- ---------- ----------

#Pama_GOX_{Fungal_GOX} ------GVVD ATAK-VYGTQ GLRVIDGSIP PTQVSSHVMT IFYGMALKVA DAILDDYAKS A--------- ---------- ---------- ----------

#AgGMC_A1_{GMC-A} ---DKQAVVD PQLR-VYGVR GLRVIDASIM PKLVSANTNA PVIMIAEKGA DMIKDFWIKR ---GVA---- ---------- ---------- ----------

#AmGMC_A1_{GMC-A} ---DPEAVVD PELR-VYGIQ GLRVIDASIM PNLVSGNTNA PVIMIGEKGS DMIKEFWLKR RSRRIVAGFV K--------- ---------- ----------

#DmGMC_A1_CG9503_{GMC-A} ---DKDAVVD AKLR-VYGIR GLRVIDASIM PKLVSANTNA PVIMIAEKGS DMIKEFWIKN ---TIV---- ---------- ---------- ----------

#TcGMC_A1_{GMC-A} ---DQEAVVD PQLR-VYGIK GLRVIDASIM PNLVSGNTNA PAIMIGEKGS DLIKEFWIKT ---ARYGRFV ---------- ---------- ----------

#AgGMC_B4_iso1_{GMC-B} -SNDPDAVVD PRLR-VKGVA GLRVVDASIM PDIVSGNTNA AVIMIGEKAS DMIKQDHG-- WNEEEKQRN- ---------- ---------- ----------

#AgGMC_B4_iso2_{GMC-B} -ATDPDAVVD SKLR-VRGVH GLRVIDASIM PLIVSGNTNA PTIMIAEMGS DFIKQEHG-- ELLDETGNEF ---------- ---------- ----------

#AgGMC_B4_iso3_{GMC-B} -DSDKEAVVD ARLR-VKGVE GLRVVDGSIM PNIVSGNTNA PIMMIGEKAS DMIKEDWG-- --EGPNHTEL ---------- ---------- ----------

#AgGMC_B4_iso4_{GMC-B} -NGDQASVVD SRLK-VRGVQ NLRVIDASIM PDIVSGNTNA PTIMIGEKGA DMIKEDYGVE KKEAATHTEL ---------- ---------- ----------

#AmGMC_B10_{GMC-B} -GNDTRAVVD PRLR-VHGVD GLRVIDGSII PEIPAANPNA ATMMIAEKGA DMVKRDWGVK AR-------- ---------- ---------- ----------

#AmGMC_B6_{GMC-B} -ADDSRAVVD SRLK-VHGVD RLRVIDASIM PTIVSGNTNA PTMMIAEKGA DMIKEDWCKD LRVEEGDDTR QTCM------ ---------- ----------

#AmGMC_B7_{GMC-B} -ANDSRAVVD SRLK-VHGVQ NLRVIDASIM PEVTSGNTNA PTMMIAEKGA DIIKQDWGVK IQI------- ---------- ---------- ----------

#AmGMC_B8_{GMC-B} -ANDSRAVVD SRLK-VHGIE NLRVIDASIM PEVTSGNTNA PTIMIGEKGA DIIKEDWGIK I--------- ---------- ---------- ----------

#AmGMC_B9_{GMC-B} -ANDSRTVVD ARLR-VHGVT NLRVIDASIM PNITSANINA PTIMIAEKGA DLIKQDWGIQ V--------- ---------- ---------- ----------

#DmEO_B1_CG9504_{GMC-B} DSQENGGVVD ERLR-VHGVK GLRVVDASIM PELPAGNTNG PAMMIGEKGA QMILDDRE-- -ANNEVIQEC ---------- ---------- ----------

#DmGMC_B2_CG9509_{GMC-B} -DYDNEACVS QRLK-VHGLE NLRVADASIM PAVVSANTNA ATVMIGERAA HFIQEDYQGE AVGANGGLWV PHMHADEF-- ---------- ----------

#DmGMC_B3_CG9512_{GMC-B} -STDPTAVVD PQLR-VHGAK GLRVIDASIM PDIVGANTNA ACIMIAEKGA DMIKEEYLG- --GKHTEL-- ---------- ---------- ----------

#TcGMC_B5_{GMC-B} -RADPTSVVD PRLR-VHGIE GLRVIDASIM PLIISGNTNA PCLMIGLKGG AMILEDWG-- ----VKHDEL ---------- ---------- ----------

#AgGMC_D1_{GMC-D} ---DPWAVVD PKLR-VYGIK GLRVIDASIM PRITSGNINA PVIMIGEKGA DMIKELWLK- ---------K GHS-RRGKRQ QFAN------ ETLSAANQTE

#AmGMC_D1_{GMC-D} ---DPMAVVD PELR-VYGVN GLRVIDASIM PTITSGNINA PVIMIGEKGA DLVKEQWWH- ---------- ---------- ---------- ESRT------

#DmGMC_D1_CG9514_{GMC-D} ---DPWAVVD PQLR-VYGIP GLRVIDASIM PAITNGNIHA PVVMIGEKGA DMIKQLWLTP TTAPVGVQGQ GPSPRQGHNT SPAPPPRTQW RSKRSLNSTE

#TcGMC_D1_{GMC-D} ---DPYAVVD PELR-VYGVA GLRVIDASIM PTITNGNLNA PTIMVGEKGA DLVKYYWLQ- ---------- ---------- ---------- PSTRRRRDLR

#AgGMC_E1_{GMC-E} ---DPGAVVD PRLR-VYGVS GLRVVDASIM PTIISGNPNA PVIMIGEKAA DMIKEDWGRL VGW------- ---------- ---------- ----------

#AmGMC_E1_{GMC-E} ---DPTAVVD PRLR-VYGVK GLRVADGSIM PEIVSGNPNA PIIMIGEKAS DMVKEDW-MR ---------- ---------- ---------- ----------

#DmGMC_E1_CG9517_{GMC-E} ---DVTAVVD PRLR-VYGVS GVRVVDASIM PTIVNGNPNA PVIAIGEKAS DLIKEDWGVR RAHTSA---- ---------- ---------- ----------

#TcGMC_E1_{GMC-E} ---DPDAVVD PRLR-VYGIK GLRVIDASIM PTIVSGNTNA PTIMIGEKGS DMIKQDWGIR V--------- ---------- ---------- ----------

#AgGMC_G2_{GMC-G} ---DPMAVVD RFLR-VHHIG GLRVVDASIF PVITTGNTNV PTIATGEKAA DLVKAAYAAD LRAHADTLRE CKTLHTDYS- ------AKAM EESQAV----

#AgGMC_G3_{GMC-G} ---DPMAVVD DQLR-VHGIG GLRVVDASIM PKITTGNTNA PTIMIAERAA DLIKYAHLPA L-AREEHYRQ CASIDYQYHP STSNTVTKVI KSSKKVP---

#AmGMC_G1_{GMC-G} ---DKMAVVD PRLR-VHGVQ GLRVIDASIM PTIVSGNTNA PTIMIAEKAA DMIKEDWG-- ----KEKKEA ETDIECSE-- ---------- ----------

#DmGMC_G1_CG12398_{GMC-G} ---DPAGVVD PRLR-VRGMR GLRVIDASIM PTIPTGNTNA PTLMLAERGA DIIK------ -----EDWRH YRDGGWG--- ---------- ----------

#TcGMC_G1_{GMC-G} ---DTMAVVD PRLR-VYGVK NLRVVDGSIM PHIVSGNTNA PIIMIAEKAA DMIKEDWAVF E---EQDQEE EPVVGFGKTP -------KVQ DLDYW-----

#AgGMC_I3_{GMC-I} ---DPDAVVS SNLK-VYGVR RLRVADVGVI PYPTSGHPTA TAYMIGEKLS DLIKNEW-LG QNIPTGSGAG GI-------- ---------- ----------

#AgGMC_I4_{GMC-I} ---DPLAVVA PDLR-VHGVQ GLRVADVSVV PTTISAQSAA IDYMIGERAA DIIKDQWEQG SSAPTSSSDR ---------- ---------- ----------

#DmGMC_I1_CG9522_{GMC-I} ---DPTTVVN HQLK-VHGVR KLRVVDTSII PFPPTAHTNA AAFMIGEKAA DMIRSEWS-- ---------- ---------- ---------- ----------

#DmGMC_I2_CG12539_{GMC-I} ---DPTTVVN HQLK-VHGVR KLRVVDTSII PFPPTAHTNA AAFMIGEKAA DMIRTDWELI ---------- ---------- ---------- ----------

#TcGMC_I5_{GMC-I} ---DPEAVVD PELR-VYGVR GLRVADTSVI PIPLTAHTNV PAFMVGEKAA DLIKETWRGA ---------- ---------- ---------- ----------

#TcGMC_I6_{GMC-I} ---DPQSVVD PRLR-VRGVK GLRVIDSSVI PVTLSAHTNA PSIMVGEKGA DLVKEDWSAI ---------- ---------- ---------- ----------

#TcGMC_I7_{GMC-I} ---DPTAVVN HELK-VYGVK GLRVADCSVI PFALGAHTNA PAIMVGEKAA DLIKADWEKV AR-------- ---------- ---------- ----------

#AgGMC_K1_{GMC-K} ---NRQAVVS PELR-VHGIG NLRIADASVM LPVSNGHTQA TVYMIAEKAS DLIKSSWDWG NELERRR--- ---------- ---------- ----------

#TcGMC_K2_{GMC-K} ---DKEAVVD HELR-VHGVQ KLRVADASVI PVSVTGHTMA PAIMVGEKAA D--------- ---------- ---------- ---------- ----------

#TcGMC_K3_{GMC-K} ---DHYAVVD KKLR-VHGIH NLRVADASVI PVTMSGSLVG PTMVIGEKAA HIIMEEWLEH ---------- ---------- ---------- ----------

#AmGMC_L1_{GMC-L} ---DSEAVVD PELR-VYGVA GLRVVDASIM PKIVRGNTNA PTIMIAEKAS DMIKDEWLYA WK-------- ---------- ---------- ----------

#TcGMC_L1_{GMC-L} ---DPFAVVD PKLR-VYGIK NLRVIDTSIM PRVTRGNTNA PTIMIAEKGA DFIKETWLKK KFSLPKIPKI PQIPKMDFKQ MIKTFFFNH- ----------

#AmGMC_M1_{GMC-M} ---DPDAVVD PQLR-VYGVW GLRVVDGSIM PTITSGHVNA AIYMIGEKAA DMIKQEWRIG H--------- ---------- ---------- ----------

#AgGMC_Q3_{GMC-Q} ---DRLAVVD PRLR-VHGVK GLRVIDASVM PDIPAAHTNG PTIMIAEKGA DMIKEDWNL- ---------- ---------- ---------- ----------

#AgGMC_Q4_{GMC-Q} ---DPTAVVD ARLR-VHGVK GLRVIDASIM PDVPAGHTNA PTIMIGEKGA DMIKQDWNEL T--------- ---------- ---------- ----------

#AmGMC_Q7_{GMC-Q} ---DETAVVD PTLK-VIGVK GLRVVDASIM PMIPSGHTNI PTYMIAEKAS DMIKDEWGYP IS-------- ---------- ---------- ----------

#DmGMC_Q1_CG9519_{GMC-Q} ---DPSAVVD ARLR-VHGID KLRVVDASIM PYLISGHPNG PVYLIAEKAA DMIKEDHNFV ---------- ---------- ---------- ----------

#DmGMC_Q2_CG9521_{GMC-Q} ---DRAAVVD HRLR-VHGIK NLRVADASIM PEIMSGHPNG PVFMIAEKAA DMIKQDHGFI Q--------- ---------- ---------- ----------

#TcGMC_Q5_{GMC-Q} ---DKKAVVD PRLR-VYGIK GLRVIDASIM PEVPAAHTNS PTFMIAEKGA DLIKEDWANR S--------- ---------- ---------- ----------

#TcGMC_Q6_{GMC-Q} ---DKSAVVD PRLR-VYGVE GLRVIDASVM PEVPAAHTNA PIFMIAEKGA DMIKEEWVGN IGAD------ ---------- ---------- ----------

#AgGMC_Z1_{GMC-Z} ---DPGAVVD PRLR-VYGIS GLRVIDASIM PTIVSGNTNA AVIMIGEKGA HMIKEDWLGH DR-------- ---------- ---------- ----------

#AmGMC_Z1_{GMC-Z} ---DPTAVVD PKLR-VYGVR GLRVIDASIM PTISSGNTNA PVIMIGEKGA DLVKNDWLAI ESARN----- ---------- ---------- ----------

#DmGMC_Z1_CG9518_{GMC-Z} ---DPEAVVD PRLR-VYGVR GLRVIDASIM PTISSGNTNA PVIMIAEKGA DLIKEDWLTN PEYKVKRQAN RLRDPDPASS NIQGIITLPN NITQGDSSNI

#TcGMC_Z1_{GMC-Z} ---DKEAVVD PRLK-VYGVE GLRVIDASIM PTIPSGNTNA PAIMVGEKGA DLVKEDWLQR ---------- ---------- ---------- ----------

#AgGLD_{Insect_GLD-GOX} ---DPLAVVD HELR-VHGVR NLRVVDASVM PKVTSGNTNA PIIMIAEKGA HLIRRAWGAR ---------- ---------- ---------- ----------

#AmGLD_{Insect_GLD-GOX} ---DPLAVVD NQLR-VRGVR GVRVADTSIM PRVISGNTNA PAIMIGERAA DFIKRTWVG- ---------- ---------- ---------- ----------

#AmGLXr_1_{Insect_GLD-GOX} ---DPTAVVD PELR-VRGVS NLRVADASVF PLVPNGNPVA AILMVAEKAA DMITHAWSKI ---------- ---------- ---------- ----------

#AmGLXr_2_{Insect_GLD-GOX} ---DRMAVVD PRLR-VHGVD GLRVADTSIM PKVTSGNTAA PAIMIGERAA AFVKSDWGGA PAKWYGHTSS SHLWREA--- ---------- ----------

#AmGOX_{Insect_GLD-GOX} ---DPMAVVS PRLK-VHGIR GLRVADASVQ PQVISGNPVA SVNMVGERAA DFIKEDWG-- ---------- -ELLQLL--- ---------- ----------

#DmGLD_{Insect_GLD-GOX} ---DPMAVVN HELR-VHGIR GLRVMDTSIM PKVSSGNTHA PAVMIAEKGA YLLKRAWGAK V--------- ---------- ---------- ----------

#TcGLD_{Insect_GLD-GOX} ---DPMAVVN PMLQ-VHGID RLRVIDASIM PAVTTGNTNA PCIMIAEKGS DLIKSRWLTP QAGFFYTNMP NQRIDRQWGS W--------- ----------

#TcGLXr_3_{Insect_GLD-GOX} ---DPMAVVD PKLQ-VYGIE GLRVMDASIM PALVSGNTHA TIVMIADKGV EYIKQKWLRG GTIANRFGGG TSQSNQNAPH FYPSASSNYP NYPKQHAPYH

#TcGLXr_4_{Insect_GLD-GOX} ---DPSAVVD PKLQ-VHGIE GLRIMDASVM PTVLSGNTHA TVVMIAEKGS DYIKQKWSDK ---------- ---------- ---------- ----------

#DmNinaG_CG6728_{NinaG} ------SVVD SQLR-LKGVS NVRVVDASVL PRPISGNPNS VVVAIALRAA SWILKSELQA GDSK------ ---------- ---------- ----------

#Tc_NinaG_like_{NinaG} -------VID EGFQ-VKGTN KLYVVDGSVL PSLPSGNPQG AIMMMAERAA EIIKHHCWLS QRRCCSSDVF QDQCSCY--- ---------- ----------

#Tc_XM_961446_{otherBeetleGMC} ---REGAVVD SELK-VFGIK KLRVADSSVF PFALAGHPTA PSVMVGEQMG DILK------ ---------- ---------- --EKYK---- ----------

#Tc_XM_961538_{otherBeetleGMC} ---KKGAVVD AKLR-VFGIK NLRVADASVF PFALAGHPNA PTVMVGEQLG DLVK------ ---------- ---------- --SDYS---- -------III

#Tc_XM_967481_{otherBeetleGMC} ---GTTAVVD NQLR-VHGIE KLRVVDAGVM PSTVSGHLNA PTVMIAEKIS DVIK------ ---------- ---------- --ATYN---- ----------

#Tc_XM_968249_{otherBeetleGMC} ---KKGAVVD SELR-VFGIK KLRVADASVF PFALAGHPNA PTVMVGEQLG DLVKRAHGVD EYLNGVSPLA IPGSTQGVGF YESSYSKGTG IPDIELMIAV

#Ag_CG6142like_1_{CG6142} ---------- ---------- ---------- ---------- ---------- ---------- ---------- ---------- ---------- ----------

#Ag_CG6142like_2_{CG6142} ---------- ---------- ---------- ---------- ---------- ---------- ---------- ---------- ---------- ----------

#Am_CG6142like_3_{CG6142} ---------- ---------- ---------- ---------- ---------- ---------- ---------- ---------- ---------- ----------

#Dm_CG6142_{CG6142} ---------- ---------- ---------- ---------- ---------- ---------- ---------- ---------- ---------- ----------

#Tc_CG6142like_4_{CG6142} ---------- ---------- ---------- ---------- ---------- ---------- ---------- ---------- ---------- ----------

#Cele_CHD_{CHD} ---------- ---------- ---------- ---------- ---------- ---------- ---------- ---------- ---------- ----------

#Ecol_CHD_{CHD} ---------- ---------- ---------- ---------- ---------- ---------- ---------- ---------- ---------- ----------

#Human_CHD_{CHD} ---------- ---------- ---------- ---------- ---------- ---------- ---------- ---------- ---------- ----------

#Anig_GOX_{Fungal_GOX} ---------- ---------- ---------- ---------- ---------- ---------- ---------- ---------- ---------- ----------

#Aory_GOX_{Fungal_GOX} ---------- ---------- ---------- ---------- ---------- ---------- ---------- ---------- ---------- ----------

#Pama_GOX_{Fungal_GOX} ---------- ---------- ---------- ---------- ---------- ---------- ---------- ---------- ---------- ----------

#AgGMC_A1_{GMC-A} ---------- ---------- ---------- ---------- ---------- ---------- ---------- ---------- ---------- ----------

#AmGMC_A1_{GMC-A} ---------- ---------- ---------- ---------- ---------- ---------- ---------- ---------- ---------- ----------

#DmGMC_A1_CG9503_{GMC-A} ---------- ---------- ---------- ---------- ---------- ---------- ---------- ---------- ---------- ----------

#TcGMC_A1_{GMC-A} ---------- ---------- ---------- ---------- ---------- ---------- ---------- ---------- ---------- ----------

#AgGMC_B4_iso1_{GMC-B} ---------- ---------- ---------- ---------- ---------- ---------- ---------- ---------- ---------- ----------

#AgGMC_B4_iso2_{GMC-B} ---------- ---------- ---------- ---------- ---------- ---------- ---------- ---------- ---------- ----------

#AgGMC_B4_iso3_{GMC-B} ---------- ---------- ---------- ---------- ---------- ---------- ---------- ---------- ---------- ----------

#AgGMC_B4_iso4_{GMC-B} ---------- ---------- ---------- ---------- ---------- ---------- ---------- ---------- ---------- ----------

#AmGMC_B10_{GMC-B} ---------- ---------- ---------- ---------- ---------- ---------- ---------- ---------- ---------- ----------

#AmGMC_B6_{GMC-B} ---------- ---------- ---------- ---------- ---------- ---------- ---------- ---------- ---------- ----------

#AmGMC_B7_{GMC-B} ---------- ---------- ---------- ---------- ---------- ---------- ---------- ---------- ---------- ----------

#AmGMC_B8_{GMC-B} ---------- ---------- ---------- ---------- ---------- ---------- ---------- ---------- ---------- ----------

#AmGMC_B9_{GMC-B} ---------- ---------- ---------- ---------- ---------- ---------- ---------- ---------- ---------- ----------

#DmEO_B1_CG9504_{GMC-B} ---------- ---------- ---------- ---------- ---------- ---------- ---------- ---------- ---------- ----------

#DmGMC_B2_CG9509_{GMC-B} ---------- ---------- ---------- ---------- ---------- ---------- ---------- ---------- ---------- ----------

#DmGMC_B3_CG9512_{GMC-B} ---------- ---------- ---------- ---------- ---------- ---------- ---------- ---------- ---------- ----------

#TcGMC_B5_{GMC-B} ---------- ---------- ---------- ---------- ---------- ---------- ---------- ---------- ---------- ----------

#AgGMC_D1_{GMC-D} AIEAVGDS-- ---SAAPCAN ETFVS----- ---------- ---------- ---------- ---------- ---------- ---------- ----------

#AmGMC_D1_{GMC-D} ---NATIG-- -----S---- ---------- ---------- ---------- ---------- ---------- ---------- ---------- ----------

#DmGMC_D1_CG9514_{GMC-D} ADTETENSEN LDIGTSPVHQ WPLPRS---- ---------- ---------- ---------- ---------- ---------- ---------- ----------

#TcGMC_D1_{GMC-D} AVYNVRLG-- -----ATCPN ITSI------ ---------- ---------- ---------- ---------- ---------- ---------- ----------

#AgGMC_E1_{GMC-E} ---------- ---------- ---------- ---------- ---------- ---------- ---------- ---------- ---------- ----------

#AmGMC_E1_{GMC-E} ---------- ---------- ---------- ---------- ---------- ---------- ---------- ---------- ---------- ----------

#DmGMC_E1_CG9517_{GMC-E} ---------- ---------- ---------- ---------- ---------- ---------- ---------- ---------- ---------- ----------

#TcGMC_E1_{GMC-E} ---------- ---------- ---------- ---------- ---------- ---------- ---------- ---------- ---------- ----------

#AgGMC_G2_{GMC-G} ---------- ---------- ---------- ---------- ---------- ---------- ---------- ---------- ---------- ----------

#AgGMC_G3_{GMC-G} ---------- ---------- ---------- ---------- ---------- ---------- ---------- ---------- ---------- ----------

#AmGMC_G1_{GMC-G} ---------- ---------- ---------- ---------- ---------- ---------- ---------- ---------- ---------- ----------

#DmGMC_G1_CG12398_{GMC-G} ---------- ---------- ---------- ---------- ---------- ---------- ---------- ---------- ---------- ----------

#TcGMC_G1_{GMC-G} ---------- ---------- ---------- ---------- ---------- ---------- ---------- ---------- ---------- ----------

#AgGMC_I3_{GMC-I} ---------- ---------- ---------- ---------- ---------- ---------- ---------- ---------- ---------- ----------

#AgGMC_I4_{GMC-I} ---------- ---------- ---------- ---------- ---------- ---------- ---------- ---------- ---------- ----------

#DmGMC_I1_CG9522_{GMC-I} ---------- ---------- ---------- ---------- ---------- ---------- ---------- ---------- ---------- ----------

#DmGMC_I2_CG12539_{GMC-I} ---------- ---------- ---------- ---------- ---------- ---------- ---------- ---------- ---------- ----------

#TcGMC_I5_{GMC-I} ---------- ---------- ---------- ---------- ---------- ---------- ---------- ---------- ---------- ----------

#TcGMC_I6_{GMC-I} ---------- ---------- ---------- ---------- ---------- ---------- ---------- ---------- ---------- ----------

#TcGMC_I7_{GMC-I} ---------- ---------- ---------- ---------- ---------- ---------- ---------- ---------- ---------- ----------

#AgGMC_K1_{GMC-K} ---------- ---------- ---------- ---------- ---------- ---------- ---------- ---------- ---------- ----------

#TcGMC_K2_{GMC-K} ---------- ---------- ---------- ---------- ---------- ---------- ---------- ---------- ---------- ----------

#TcGMC_K3_{GMC-K} ---------- ---------- ---------- ---------- ---------- ---------- ---------- ---------- ---------- ----------

#AmGMC_L1_{GMC-L} ---------- ---------- ---------- ---------- ---------- ---------- ---------- ---------- ---------- ----------

#TcGMC_L1_{GMC-L} ---------- ---------- ---------- ---------- ---------- ---------- ---------- ---------- ---------- ----------

#AmGMC_M1_{GMC-M} ---------- ---------- ---------- ---------- ---------- ---------- ---------- ---------- ---------- ----------

#AgGMC_Q3_{GMC-Q} ---------- ---------- ---------- ---------- ---------- ---------- ---------- ---------- ---------- ----------

#AgGMC_Q4_{GMC-Q} ---------- ---------- ---------- ---------- ---------- ---------- ---------- ---------- ---------- ----------

#AmGMC_Q7_{GMC-Q} ---------- ---------- ---------- ---------- ---------- ---------- ---------- ---------- ---------- ----------

#DmGMC_Q1_CG9519_{GMC-Q} ---------- ---------- ---------- ---------- ---------- ---------- ---------- ---------- ---------- ----------

#DmGMC_Q2_CG9521_{GMC-Q} ---------- ---------- ---------- ---------- ---------- ---------- ---------- ---------- ---------- ----------

#TcGMC_Q5_{GMC-Q} ---------- ---------- ---------- ---------- ---------- ---------- ---------- ---------- ---------- ----------

#TcGMC_Q6_{GMC-Q} ---------- ---------- ---------- ---------- ---------- ---------- ---------- ---------- ---------- ----------

#AgGMC_Z1_{GMC-Z} ---------- ---------- ---------- ---------- ---------- ---------- ---------- ---------- ---------- ----------

#AmGMC_Z1_{GMC-Z} ---------- ---------- ---------- ---------- ---------- ---------- ---------- ---------- ---------- ----------

#DmGMC_Z1_CG9518_{GMC-Z} SDRSNMESNF SNSSHINNIN FNSNSNSSNI ESSSNFTLNY ANS------- ---------- ---------- ---------- ---------- ----------

#TcGMC_Z1_{GMC-Z} ---------- ---------- ---------- ---------- ---------- ---------- ---------- ---------- ---------- ----------

#AgGLD_{Insect_GLD-GOX} ---------- ---------- ---------- ---------- ---------- ---------- ---------- ---------- ---------- ----------

#AmGLD_{Insect_GLD-GOX} ---------- ---------- ---------- ---------- ---------- ---------- ---------- ---------- ---------- ----------

#AmGLXr_1_{Insect_GLD-GOX} ---------- ---------- ---------- ---------- ---------- ---------- ---------- ---------- ---------- ----------

#AmGLXr_2_{Insect_GLD-GOX} ---------- ---------- ---------- ---------- ---------- ---------- ---------- ---------- ---------- ----------

#AmGOX_{Insect_GLD-GOX} ---------- ---------- ---------- ---------- ---------- ---------- ---------- ---------- ---------- ----------

#DmGLD_{Insect_GLD-GOX} ---------- ---------- ---------- ---------- ---------- ---------- ---------- ---------- ---------- ----------

#TcGLD_{Insect_GLD-GOX} ---------- ---------- ---------- ---------- ---------- ---------- ---------- ---------- ---------- ----------

#TcGLXr_3_{Insect_GLD-GOX} YQQGVKSTGF RHNEQFHRNH PQMPNPFMST PRPQRAYNQQ GFNQGYQGYQ HNYPDYQQDQ EYNNFNAY-- ---------- ---------- ----------

#TcGLXr_4_{Insect_GLD-GOX} ---------- ---------- ---------- ---------- ---------- ---------- ---------- ---------- ---------- ----------

#DmNinaG_CG6728_{NinaG} ---------- ---------- ---------- ---------- ---------- ---------- ---------- ---------- ---------- ----------

#Tc_NinaG_like_{NinaG} ---------- ---------- ---------- ---------- ---------- ---------- ---------- ---------- ---------- ----------

#Tc_XM_961446_{otherBeetleGMC} YNDNY----- ---------- ----YDVFHD YF-------- ---------- ---------- ---------- ---------- ---------- ----------

#Tc_XM_961538_{otherBeetleGMC} FNSAVPFAN- ---------- ----YIMISK FVVFFVRS-- ---------- ---------- ---------- ---------- ---------- ----------

#Tc_XM_967481_{otherBeetleGMC} ---------- ---------- ---------- ---------- ---------- ---------- ---------- ---------- ---------- ----------

#Tc_XM_968249_{otherBeetleGMC} ANATDQLTQR YFSLTDQTYE DVWKYNNIPQ TFIFHVVNLH AQSSGSVRLK SKNPFEYPVI NSNFLSDPEN RDINTLYKGI QICLKMGETK AMEAINATLQ

#Ag_CG6142like_1_{CG6142} ---------- ---------- ---------- ---------- ---------- ---------- ---------- --

#Ag_CG6142like_2_{CG6142} ---------- ---------- ---------- ---------- ---------- ---------- ---------- --

#Am_CG6142like_3_{CG6142} ---------- ---------- ---------- ---------- ---------- ---------- ---------- --

#Dm_CG6142_{CG6142} ---------- ---------- ---------- ---------- ---------- ---------- ---------- --

#Tc_CG6142like_4_{CG6142} ---------- ---------- ---------- ---------- ---------- ---------- ---------- --

#Cele_CHD_{CHD} ---------- ---------- ---------- ---------- ---------- ---------- ---------- --

#Ecol_CHD_{CHD} ---------- ---------- ---------- ---------- ---------- ---------- ---------- --

#Human_CHD_{CHD} ---------- ---------- ---------- ---------- ---------- ---------- ---------- --

#Anig_GOX_{Fungal_GOX} ---------- ---------- ---------- ---------- ---------- ---------- ---------- --

#Aory_GOX_{Fungal_GOX} ---------- ---------- ---------- ---------- ---------- ---------- ---------- --

#Pama_GOX_{Fungal_GOX} ---------- ---------- ---------- ---------- ---------- ---------- ---------- --

#AgGMC_A1_{GMC-A} ---------- ---------- ---------- ---------- ---------- ---------- ---------- --

#AmGMC_A1_{GMC-A} ---------- ---------- ---------- ---------- ---------- ---------- ---------- --

#DmGMC_A1_CG9503_{GMC-A} ---------- ---------- ---------- ---------- ---------- ---------- ---------- --

#TcGMC_A1_{GMC-A} ---------- ---------- ---------- ---------- ---------- ---------- ---------- --

#AgGMC_B4_iso1_{GMC-B} ---------- ---------- ---------- ---------- ---------- ---------- ---------- --

#AgGMC_B4_iso2_{GMC-B} ---------- ---------- ---------- ---------- ---------- ---------- ---------- --

#AgGMC_B4_iso3_{GMC-B} ---------- ---------- ---------- ---------- ---------- ---------- ---------- --

#AgGMC_B4_iso4_{GMC-B} ---------- ---------- ---------- ---------- ---------- ---------- ---------- --

#AmGMC_B10_{GMC-B} ---------- ---------- ---------- ---------- ---------- ---------- ---------- --

#AmGMC_B6_{GMC-B} ---------- ---------- ---------- ---------- ---------- ---------- ---------- --

#AmGMC_B7_{GMC-B} ---------- ---------- ---------- ---------- ---------- ---------- ---------- --

#AmGMC_B8_{GMC-B} ---------- ---------- ---------- ---------- ---------- ---------- ---------- --

#AmGMC_B9_{GMC-B} ---------- ---------- ---------- ---------- ---------- ---------- ---------- --

#DmEO_B1_CG9504_{GMC-B} ---------- ---------- ---------- ---------- ---------- ---------- ---------- --

#DmGMC_B2_CG9509_{GMC-B} ---------- ---------- ---------- ---------- ---------- ---------- ---------- --

#DmGMC_B3_CG9512_{GMC-B} ---------- ---------- ---------- ---------- ---------- ---------- ---------- --

#TcGMC_B5_{GMC-B} ---------- ---------- ---------- ---------- ---------- ---------- ---------- --

#AgGMC_D1_{GMC-D} ---------- ---------- ---------- ---------- ---------- ---------- ---------- --

#AmGMC_D1_{GMC-D} ---------- ---------- ---------- ---------- ---------- ---------- ---------- --

#DmGMC_D1_CG9514_{GMC-D} ---------- ---------- ---------- ---------- ---------- ---------- ---------- --

#TcGMC_D1_{GMC-D} ---------- ---------- ---------- ---------- ---------- ---------- ---------- --

#AgGMC_E1_{GMC-E} ---------- ---------- ---------- ---------- ---------- ---------- ---------- --

#AmGMC_E1_{GMC-E} ---------- ---------- ---------- ---------- ---------- ---------- ---------- --

#DmGMC_E1_CG9517_{GMC-E} ---------- ---------- ---------- ---------- ---------- ---------- ---------- --

#TcGMC_E1_{GMC-E} ---------- ---------- ---------- ---------- ---------- ---------- ---------- --

#AgGMC_G2_{GMC-G} ---------- ---------- ---------- ---------- ---------- ---------- ---------- --

#AgGMC_G3_{GMC-G} ---------- ---------- ---------- ---------- ---------- ---------- ---------- --

#AmGMC_G1_{GMC-G} ---------- ---------- ---------- ---------- ---------- ---------- ---------- --

#DmGMC_G1_CG12398_{GMC-G} ---------- ---------- ---------- ---------- ---------- ---------- ---------- --

#TcGMC_G1_{GMC-G} ---------- ---------- ---------- ---------- ---------- ---------- ---------- --

#AgGMC_I3_{GMC-I} ---------- ---------- ---------- ---------- ---------- ---------- ---------- --

#AgGMC_I4_{GMC-I} ---------- ---------- ---------- ---------- ---------- ---------- ---------- --

#DmGMC_I1_CG9522_{GMC-I} ---------- ---------- ---------- ---------- ---------- ---------- ---------- --

#DmGMC_I2_CG12539_{GMC-I} ---------- ---------- ---------- ---------- ---------- ---------- ---------- --

#TcGMC_I5_{GMC-I} ---------- ---------- ---------- ---------- ---------- ---------- ---------- --

#TcGMC_I6_{GMC-I} ---------- ---------- ---------- ---------- ---------- ---------- ---------- --

#TcGMC_I7_{GMC-I} ---------- ---------- ---------- ---------- ---------- ---------- ---------- --

#AgGMC_K1_{GMC-K} ---------- ---------- ---------- ---------- ---------- ---------- ---------- --

#TcGMC_K2_{GMC-K} ---------- ---------- ---------- ---------- ---------- ---------- ---------- --

#TcGMC_K3_{GMC-K} ---------- ---------- ---------- ---------- ---------- ---------- ---------- --

#AmGMC_L1_{GMC-L} ---------- ---------- ---------- ---------- ---------- ---------- ---------- --

#TcGMC_L1_{GMC-L} ---------- ---------- ---------- ---------- ---------- ---------- ---------- --

#AmGMC_M1_{GMC-M} ---------- ---------- ---------- ---------- ---------- ---------- ---------- --

#AgGMC_Q3_{GMC-Q} ---------- ---------- ---------- ---------- ---------- ---------- ---------- --

#AgGMC_Q4_{GMC-Q} ---------- ---------- ---------- ---------- ---------- ---------- ---------- --

#AmGMC_Q7_{GMC-Q} ---------- ---------- ---------- ---------- ---------- ---------- ---------- --

#DmGMC_Q1_CG9519_{GMC-Q} ---------- ---------- ---------- ---------- ---------- ---------- ---------- --

#DmGMC_Q2_CG9521_{GMC-Q} ---------- ---------- ---------- ---------- ---------- ---------- ---------- --

#TcGMC_Q5_{GMC-Q} ---------- ---------- ---------- ---------- ---------- ---------- ---------- --

#TcGMC_Q6_{GMC-Q} ---------- ---------- ---------- ---------- ---------- ---------- ---------- --

#AgGMC_Z1_{GMC-Z} ---------- ---------- ---------- ---------- ---------- ---------- ---------- --

#AmGMC_Z1_{GMC-Z} ---------- ---------- ---------- ---------- ---------- ---------- ---------- --

#DmGMC_Z1_CG9518_{GMC-Z} ---------- ---------- ---------- ---------- ---------- ---------- ---------- --

#TcGMC_Z1_{GMC-Z} ---------- ---------- ---------- ---------- ---------- ---------- ---------- --

#AgGLD_{Insect_GLD-GOX} ---------- ---------- ---------- ---------- ---------- ---------- ---------- --

#AmGLD_{Insect_GLD-GOX} ---------- ---------- ---------- ---------- ---------- ---------- ---------- --

#AmGLXr_1_{Insect_GLD-GOX} ---------- ---------- ---------- ---------- ---------- ---------- ---------- --

#AmGLXr_2_{Insect_GLD-GOX} ---------- ---------- ---------- ---------- ---------- ---------- ---------- --

#AmGOX_{Insect_GLD-GOX} ---------- ---------- ---------- ---------- ---------- ---------- ---------- --

#DmGLD_{Insect_GLD-GOX} ---------- ---------- ---------- ---------- ---------- ---------- ---------- --

#TcGLD_{Insect_GLD-GOX} ---------- ---------- ---------- ---------- ---------- ---------- ---------- --

#TcGLXr_3_{Insect_GLD-GOX} ---------- ---------- ---------- ---------- ---------- ---------- ---------- --

#TcGLXr_4_{Insect_GLD-GOX} ---------- ---------- ---------- ---------- ---------- ---------- ---------- --

#DmNinaG_CG6728_{NinaG} ---------- ---------- ---------- ---------- ---------- ---------- ---------- --

#Tc_NinaG_like_{NinaG} ---------- ---------- ---------- ---------- ---------- ---------- ---------- --

#Tc_XM_961446_{otherBeetleGMC} ---------- ---------- ---------- ---------- ---------- ---------- ---------- --

#Tc_XM_961538_{otherBeetleGMC} ---------- ---------- ---------- ---------- ---------- ---------- ---------- --

#Tc_XM_967481_{otherBeetleGMC} ---------- ---------- ---------- ---------- ---------- ---------- ---------- --

#Tc_XM_968249_{otherBeetleGMC} GGPLRACKRY QYLSKDYWYC ALRQITVNLY QPLGSCPMGK DPKKGAVVVS ELRVFGERAV GGFGQKGPWG RW
