# Supplementary material for: Expansion and evolution of insect GMC oxidoreductases
Source: BMC Evol Biol. 2007 May 11;7:75. doi: 10.1186/1471-2148-7-75 (PMC1891103; doi:10.1186/1471-2148-7-75)
Supplement: Additional File 2 — Pairwise distance matrices. The average pairwise distance for different GMC subfamilies and pairwise distance matrices within each subfamily. [file 1471-2148-7-75-S2.doc]

Average pairwise distance within different GMC subfamilies

|  | **Average** | **S.E.** |
| --- | --- | --- |
| GMCζ | 0.28 | 0.012 |
| GMCα | 0.28 | 0.014 |
| GMCδ | 0.29 | 0.012 |
| GMCε | 0.32 | 0.015 |
| GMCλ | 0.46 | 0.020 |
| GMCθ | 0.49 | 0.013 |
| GMCγ | 0.50 | 0.014 |
| GMCι | 0.55 | 0.012 |
| GMCκ | 0.59 | 0.017 |
| GMCβ | 0.59 | 0.011 |
| Insect GLD/GOX/GLXr | 0.49 | 0.012 |
| Other beetle GMC | 0.46 | 0.013 |
| CG6142 | 0.49 | 0.013 |
| CHD | 0.51 | 0.017 |
| Fungal GOX | 0.33 | 0.014 |
| NinaG | 0.72 | 0.018 |

The average pairwise distance (the number of amino acid differences per site) was computed for each GMC subfamily. Standard error (S.E.) was estimated by 500 replicates of bootstrap re-sampling. Gaps were deleted for each pairwise comparison (pairwise deletion). Estimations are not applicable for the GMCμ subfamily because it contains only one sequence.

**Pairwise distance matrices within different GMC subfamilies**

Pairwise distance matrices for different GMC subfamilies are shown below. Estimates of pairwise distance (the number of amino acid differences per site) are listed on the lower left part of each matrix and estimates of analytic standard error (S.E.) are listed on the upper right part of each matrix. Gaps were deleted for each pairwise comparison (pairwise deletion). Distance matrix is not applicable for the GMCμ subfamily because it contains only one sequence.

Distance matrix for GMC α subfamily

[1] #DmGMC_A1_CG9503_{GMC-A}

[2] #AgGMC_A1_{GMC-A}

[3] #AmGMC_A1_{GMC-A}

[4] #TcGMC_A1_{GMC-A}

[ 1 2 3 4 ]

[1] [0.02 ][0.02 ][0.02 ]

[2] 0.21 [0.02 ][0.02 ]

[3] 0.31 0.30 [0.02 ]

[4] 0.30 0.29 0.29

Distance matrix for GMC β subfamily

[ 1] #DmEO_B1_CG9504_{GMC-B}

[ 2] #DmGMC_B2_CG9509_{GMC-B}

[ 3] #DmGMC_B3_CG9512_{GMC-B}

[ 4] #AgGMC_B4_iso4_{GMC-B}

[ 5] #AgGMC_B4_iso3_{GMC-B}

[ 6] #AgGMC_B4_iso2_{GMC-B}

[ 7] #AgGMC_B4_iso1_{GMC-B}

[ 8] #AmGMC_B6_{GMC-B}

[ 9] #AmGMC_B7_{GMC-B}

[10] #AmGMC_B8_{GMC-B}

[11] #AmGMC_B9_{GMC-B}

[12] #AmGMC_B10_{GMC-B}

[13] #TcGMC_B5_{GMC-B}

[ 1 2 3 4 5 6 7 8 9 10 11 12 13 ]

[ 1] [0.02 ][0.02 ][0.02 ][0.02 ][0.02 ][0.02 ][0.02 ][0.02 ][0.02 ][0.02 ][0.02 ][0.02 ]

[ 2] 0.68 [0.02 ][0.02 ][0.02 ][0.02 ][0.02 ][0.02 ][0.02 ][0.02 ][0.02 ][0.02 ][0.02 ]

[ 3] 0.70 0.60 [0.02 ][0.02 ][0.02 ][0.02 ][0.02 ][0.02 ][0.02 ][0.02 ][0.02 ][0.02 ]

[ 4] 0.69 0.58 0.59 [0.02 ][0.02 ][0.02 ][0.02 ][0.02 ][0.02 ][0.02 ][0.02 ][0.02 ]

[ 5] 0.67 0.59 0.60 0.45 [0.02 ][0.02 ][0.02 ][0.02 ][0.02 ][0.02 ][0.02 ][0.02 ]

[ 6] 0.69 0.62 0.61 0.49 0.46 [0.02 ][0.02 ][0.02 ][0.02 ][0.02 ][0.02 ][0.02 ]

[ 7] 0.69 0.61 0.59 0.49 0.45 0.45 [0.02 ][0.02 ][0.02 ][0.02 ][0.02 ][0.02 ]

[ 8] 0.70 0.65 0.63 0.62 0.62 0.61 0.62 [0.02 ][0.02 ][0.02 ][0.02 ][0.02 ]

[ 9] 0.71 0.65 0.65 0.62 0.63 0.62 0.64 0.37 [0.02 ][0.02 ][0.02 ][0.02 ]

[10] 0.71 0.64 0.62 0.61 0.62 0.60 0.60 0.38 0.26 [0.02 ][0.02 ][0.02 ]

[11] 0.70 0.66 0.64 0.63 0.64 0.62 0.64 0.43 0.37 0.41 [0.02 ][0.02 ]

[12] 0.71 0.66 0.66 0.63 0.62 0.63 0.62 0.43 0.45 0.49 0.49 [0.02 ]

[13] 0.67 0.62 0.63 0.56 0.59 0.61 0.59 0.61 0.60 0.61 0.64 0.63

Distance matrix for GMC γ subfamily

[1] #DmGMC_G1_CG12398_{GMC-G}

[2] #AgGMC_G3_{GMC-G}

[3] #AgGMC_G2_{GMC-G}

[4] #AmGMC_G1_{GMC-G}

[5] #TcGMC_G1_{GMC-G}

[ 1 2 3 4 5 ]

[1] [0.02 ][0.02 ][0.02 ][0.02 ]

[2] 0.51 [0.02 ][0.02 ][0.02 ]

[3] 0.50 0.44 [0.02 ][0.02 ]

[4] 0.51 0.54 0.52 [0.02 ]

[5] 0.52 0.54 0.54 0.42

Distance matrix for GMC δ subfamily

[1] #DmGMC_D1_CG9514_{GMC-D}

[2] #AgGMC_D1_{GMC-D}

[3] #AmGMC_D1_{GMC-D}

[4] #TcGMC_D1_{GMC-D}

[ 1 2 3 4 ]

[1] [0.02 ][0.02 ][0.02 ]

[2] 0.26 [0.02 ][0.02 ]

[3] 0.30 0.28 [0.02 ]

[4] 0.33 0.30 0.28

Distance matrix for GMC ε subfamily

[1] #DmGMC_E1_CG9517_{GMC-E}

[2] #AgGMC_E1_{GMC-E}

[3] #AmGMC_E1_{GMC-E}

[4] #TcGMC_E1_{GMC-E}

[ 1 2 3 4 ]

[1] [0.02 ][0.02 ][0.02 ]

[2] 0.27 [0.02 ][0.02 ]

[3] 0.32 0.34 [0.02 ]

[4] 0.36 0.34 0.31

Distance matrix for GMC ζ subfamily

[1] #DmGMC_Z1_CG9518_{GMC-Z}

[2] #AgGMC_Z1_{GMC-Z}

[3] #AmGMC_Z1_{GMC-Z}

[4] #TcGMC_Z1_{GMC-Z}

[ 1 2 3 4 ]

[1] [0.02 ][0.02 ][0.02 ]

[2] 0.24 [0.02 ][0.02 ]

[3] 0.29 0.29 [0.02 ]

[4] 0.27 0.30 0.25

Distance matrix for GMC θ subfamily

[1] #DmGMC_Q1_CG9519_{GMC-Q}

[2] #DmGMC_Q2_CG9521_{GMC-Q}

[3] #AgGMC_Q4_{GMC-Q}

[4] #AgGMC_Q3_{GMC-Q}

[5] #AmGMC_Q7_{GMC-Q}

[6] #TcGMC_Q6_{GMC-Q}

[7] #TcGMC_Q5_{GMC-Q}

[ 1 2 3 4 5 6 7 ]

[1] [0.02 ][0.02 ][0.02 ][0.02 ][0.02 ][0.02 ]

[2] 0.35 [0.02 ][0.02 ][0.02 ][0.02 ][0.02 ]

[3] 0.50 0.48 [0.02 ][0.02 ][0.02 ][0.02 ]

[4] 0.49 0.47 0.33 [0.02 ][0.02 ][0.02 ]

[5] 0.58 0.57 0.54 0.53 [0.02 ][0.02 ]

[6] 0.51 0.50 0.52 0.50 0.57 [0.02 ]

[7] 0.50 0.48 0.49 0.48 0.56 0.32

Distance matrix for GMC ι subfamily

[1] #DmGMC_I1_CG9522_{GMC-I}

[2] #DmGMC_I2_CG12539_{GMC-I}

[3] #AgGMC_I4_{GMC-I}

[4] #AgGMC_I3_{GMC-I}

[5] #TcGMC_I5_{GMC-I}

[6] #TcGMC_I6_{GMC-I}

[7] #TcGMC_I7_{GMC-I}

[ 1 2 3 4 5 6 7 ]

[1] [0.02 ][0.02 ][0.02 ][0.02 ][0.02 ][0.02 ]

[2] 0.29 [0.02 ][0.02 ][0.02 ][0.02 ][0.02 ]

[3] 0.54 0.56 [0.02 ][0.02 ][0.02 ][0.02 ]

[4] 0.54 0.55 0.54 [0.02 ][0.02 ][0.02 ]

[5] 0.56 0.58 0.59 0.56 [0.02 ][0.02 ]

[6] 0.58 0.58 0.60 0.60 0.53 [0.02 ]

[7] 0.58 0.59 0.60 0.59 0.51 0.49

Distance matrix for GMC CG6142 subfamily

[1] #Dm_CG6142_{CG6142}

[2] #Ag_CG6142like_1_{CG6142}

[3] #Ag_CG6142like_2_{CG6142}

[4] #Am_CG6142like_3_{CG6142}

[5] #Tc_CG6142like_4_{CG6142}

[ 1 2 3 4 5 ]

[1] [0.02 ][0.02 ][0.02 ][0.02 ]

[2] 0.43 [0.02 ][0.02 ][0.02 ]

[3] 0.48 0.35 [0.02 ][0.02 ]

[4] 0.54 0.55 0.55 [0.02 ]

[5] 0.49 0.47 0.50 0.49

Distance matrix for GMC κ subfamily

[1] #AgGMC_K1_{GMC-K}

[2] #TcGMC_K2_{GMC-K}

[3] #TcGMC_K3_{GMC-K}

[ 1 2 3 ]

[1] [0.02 ][0.02 ]

[2] 0.63 [0.02 ]

[3] 0.66 0.47

Distance matrix for Insect GLD gene family

[1] #AmGLXr_1_{Insect_GLD-GOX}

[2] #TcGLXr_3_{Insect_GLD-GOX}

[3] #TcGLXr_4_{Insect_GLD-GOX}

[4] #TcGLD_{Insect_GLD-GOX}

[5] #AmGLD_{Insect_GLD-GOX}

[6] #DmGLD_{Insect_GLD-GOX}

[7] #AgGLD_{Insect_GLD-GOX}

[8] #AmGLXr_2_{Insect_GLD-GOX}

[9] #AmGOX_{Insect_GLD-GOX}

[ 1 2 3 4 5 6 7 8 9 ]

[1] [0.02 ][0.02 ][0.02 ][0.02 ][0.02 ][0.02 ][0.02 ][0.02 ]

[2] 0.60 [0.02 ][0.02 ][0.02 ][0.02 ][0.02 ][0.02 ][0.02 ]

[3] 0.59 0.37 [0.02 ][0.02 ][0.02 ][0.02 ][0.02 ][0.02 ]

[4] 0.55 0.47 0.51 [0.02 ][0.02 ][0.02 ][0.02 ][0.02 ]

[5] 0.55 0.46 0.52 0.32 [0.02 ][0.02 ][0.02 ][0.02 ]

[6] 0.55 0.44 0.48 0.35 0.37 [0.02 ][0.02 ][0.02 ]

[7] 0.54 0.46 0.48 0.35 0.37 0.29 [0.02 ][0.02 ]

[8] 0.59 0.50 0.53 0.49 0.47 0.50 0.46 [0.02 ]

[9] 0.60 0.57 0.57 0.58 0.58 0.56 0.56 0.50

Distance matrix for GMC λ subfamily

[1] #AmGMC_L1_{GMC-L}

[2] #TcGMC_L1_{GMC-L}

[ 1 2 ]

[1] [0.02 ]

[2] 0.46

Distance matrix for other Beetle GMC genes

[1] #Tc_XM_961446_{otherBeetleGMC}

[2] #Tc_XM_961538_{otherBeetleGMC}

[3] #Tc_XM_968249_{otherBeetleGMC}

[4] #Tc_XM_967481_{otherBeetleGMC}

[ 1 2 3 4 ]

[1] [0.02 ][0.02 ][0.02 ]

[2] 0.34 [0.02 ][0.02 ]

[3] 0.38 0.30 [0.02 ]

[4] 0.57 0.56 0.58

Distance matrix for CHD gene group

[1] #Human_CHD_{CHD}

[2] #Cele_CHD_{CHD}

[3] #Ecol_CHD_{CHD}

[ 1 2 3 ]

[1] [0.02 ][0.02 ]

[2] 0.46 [0.02 ]

[3] 0.52 0.55

Distance matrix for the fungal GOX gene group

[1] #Anig_GOX_{Fungal_GOX}

[2] #Pama_GOX_{Fungal_GOX}

[3] #Aory_GOX_{Fungal_GOX}

[ 1 2 3 ]

[1] [0.02 ][0.02 ]

[2] 0.37 [0.02 ]

[3] 0.35 0.27

Distance matrix for Nina G gene group

[1] #DmNinaG_CG6728_{NinaG}

[2] #Tc_NinaG_like_{NinaG}

[ 1 2 ]

[1] [0.02 ]

[2] 0.72
