# Supplementary material for: Expansion and evolution of insect GMC oxidoreductases
Source: BMC Evol Biol. 2007 May 11;7:75. doi: 10.1186/1471-2148-7-75 (PMC1891103; doi:10.1186/1471-2148-7-75)

Supplementary material

A maximum parsimony tree of GMC gene subfamilies. Close-neighbor-interchange (CNI) method was employed for taxa clustering at search level = 1 (default setting in MEGA3). Scores of bootstrap re-sampling analysis with 500 replicates are shown at tree nodes. See Figure 3 for the details of the sequence names.

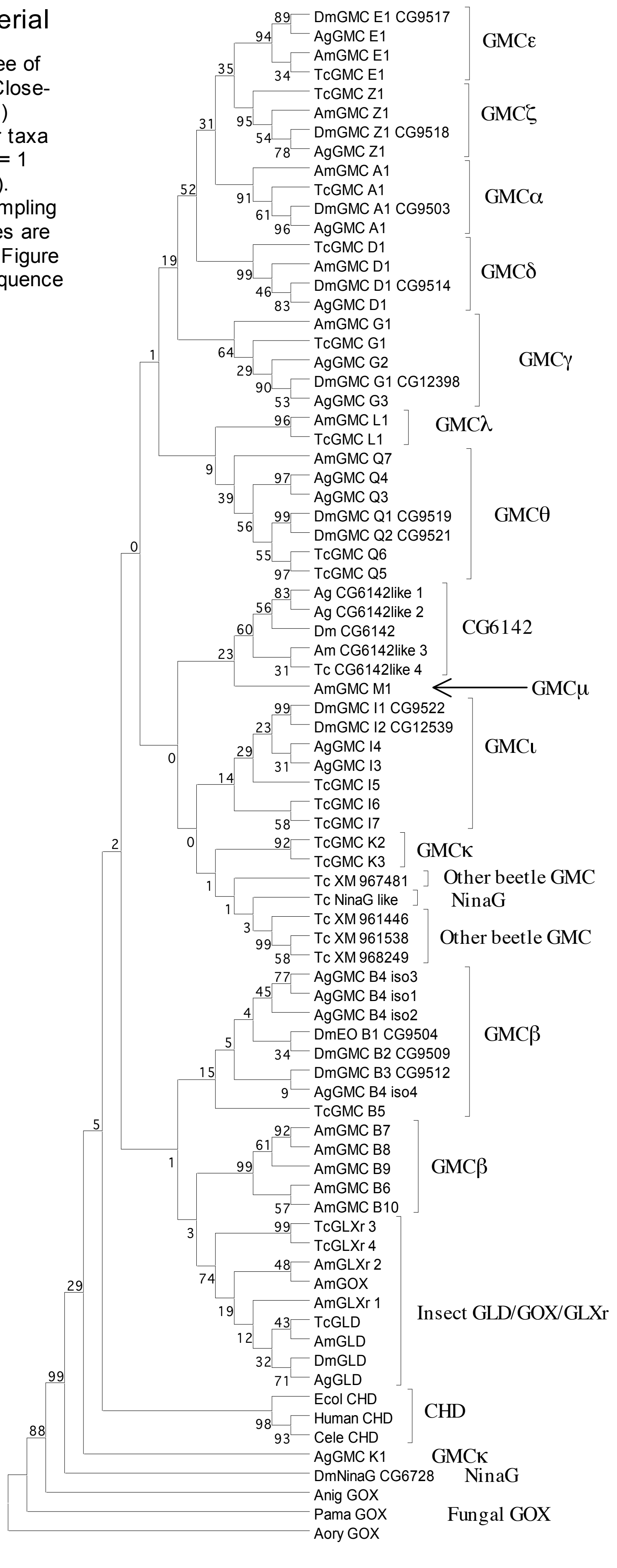

Supplementary material

A neighbor-joining tree of GMC gene subfamilies. Pairwise distances between amino acid sequences were estimated assuming the JTT substitution model. Scores of bootstrap re-sampling analysis with 500 replicates are shown at tree nodes. See Figure 3 for the details of the sequence names.

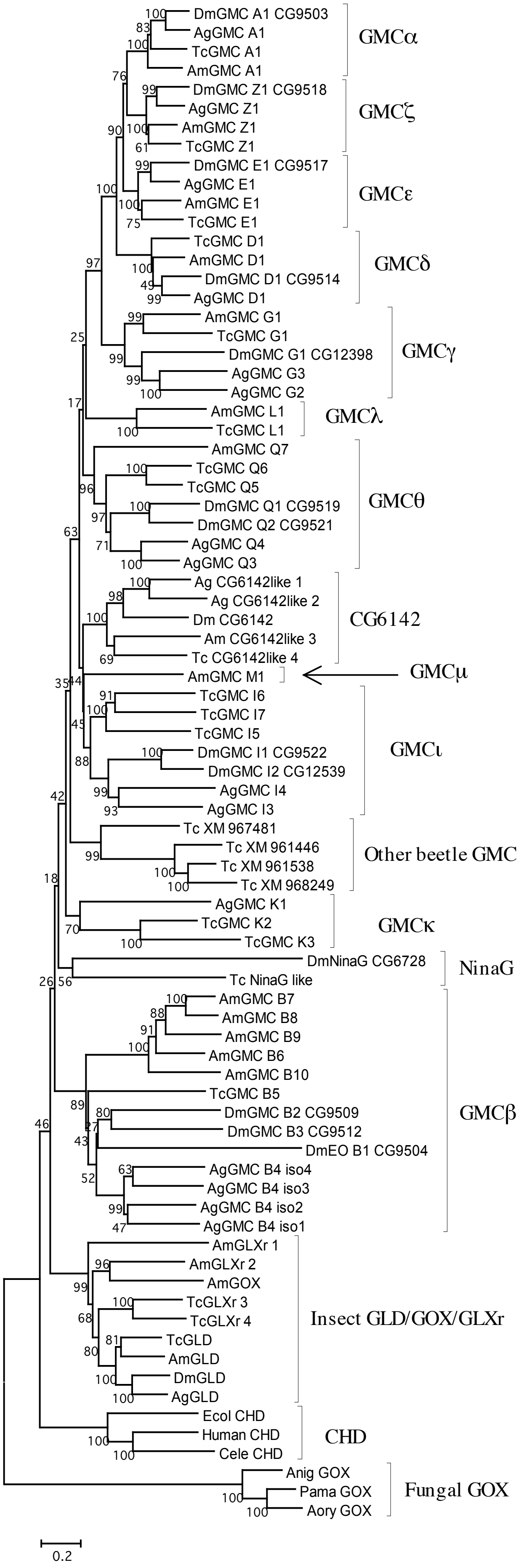

Supplement: Additional File 3 — Phylogeny of GMC genes. Phylogenetic trees of GMC genes based on a different method and substitution model. [file 1471-2148-7-75-S3.pdf]
