# Supplementary material for: Expansion and evolution of insect GMC oxidoreductases
Source: BMC Evol Biol. 2007 May 11;7:75. doi: 10.1186/1471-2148-7-75 (PMC1891103; doi:10.1186/1471-2148-7-75)
Supplement: Additional File 6 — The alternative splicing pattern of A. mellifera GLXr-2. The 3' end exon/intron sequences of A. mellifera GLXr-2 gene. [file 1471-2148-7-75-S6.pdf]

GLXr-2 alternative splicing

The coding sequence of GLXr-2 isoform I terminates in exon 8, whereas isoform II splices out of exon 8 (at #) before reaching the termination codon and adds a unique carboxy-terminus encoded in exon 9 (85bp). See Figure 6. Underlined (bold letter) sequences indicate positions for the primers that amplify the two products: 548 bp for Isoform I and 455 bp for Isoform II.

Exon 7 (371 bp)
GCAGCCTTCGACTGGCGAGCAACGACCCGTTCGCCAAACCAGTGATCCACGGCAATTAT
TTGAGCGACCCGATGGACGAGGCAGTCCTTCTCCACGGGATTCGGATAGCCCTGTCGCT
GAGCAACACGAGCGCGTTGGCCAGGTACAACATGACTCTCGCCAACCTCCCTCTCCCCG
CCTGCTCCCAGCACACGTATCTAAGCGACGACTACTGGAGGTGCGCCATGCGCCAGGAC
AC**CGGCCCGGAGAATCATCAG**GCCGGTTCCTGCAAAATGGGCCCGGTCAGCGACCGGAT
GGCGGTGGTCGACCCGAGGCTGAGGGTGCACGGCGTCGACGGTTTGCGCGTGGCTGACA
CGTCCATCATGCCCAAG

Exon 8 (95 bp)
GTGACGTCCGGGAACACGGCCGCTCCAGCGATCATGATCGGCGAGAGGGCAGCGGCTTT
CGTCAAGTCTGACTGGGGTGGCGCGCCTGCAAAATG#

Exon8 (43 bp)
GTACGGGCATACTTCTTCTTCCCATCTGTGGAGAGAGGCGTGA (stop for Isoform I)

Intron (50 bp)
CGGAGTAGAGAGGAGTACGATAGTCAAATATTTGCCATATATTTCCATAG

Exon9 (85 bp)
TAGCCCCCGTCCCGAGATCGACAACTCGTTGGAACTGTTGCACTGGGGGATCAAATACA
ACGACTGGGACCAGGGCGACTGGTAG (stop for Isoform II)

3′ UTR
TCAACGTCCTCGAGATCTCACTAGATCCGGATAACCGGTTTTATCCCTTCGATCGGGAG
AAAGTATTCCAGATAGTTTCTTCTCCTTTTCAACATCCACTCTTTGTAACAAACGTC**GA
GACCAAAGAAATGTAAATGCGGAT**TCAATAAA (putative poly(A) signal)
