# Supplementary material for: Expansion and evolution of insect GMC oxidoreductases
Source: BMC Evol Biol. 2007 May 11;7:75. doi: 10.1186/1471-2148-7-75 (PMC1891103; doi:10.1186/1471-2148-7-75)
Supplement: Additional File 9 — Coding sequences. CDS of the manually annotated sequences used in this study. [file 1471-2148-7-75-S9.rtf]

>AgGMC_I4ATGAAGCATTTGTGGATCGCGGTGATCCTAATCGCGACACATAGTGCACTGACCGCGAACGGATTTTTTCTACTGCTCAAAACGCTGGCCCATGCTGGACGCTACATTAACGAGCATTATCCAGATGAGGGCATTAACTACCGCCAGTCGGTGCCGGAGTATGATTTCATAATTGTTGGTGCCGGCGCAGCCGGATGTGTGCTGGCCAACCGTCTGTCGGAAAACCCGCAGTGGAAGATCTTGCTGCTAGAGGCAGGACCGGGCGAGAACGACTTGCAGAACATTCCGCTGCTGACTACCTTTCTGCAAAACTCCCAATACAATTGGGCGGACATTGCCGAGGCACAAAACACCTCCTGCTATGGCATGATCGATCAACGGTGCAGTCTGCCGCACGGCAAAGGTCTGGGAGGATCGACCCTTATCGACTACATGCTGTACGGGCGCGGTAATCCCGCCGATTACGACCGGTGGGCCGCACAGGGCAACCCGGGCTGGTCACATGCCGATCTCTTCCCGTACTTTCTCAAATCCGAACGGGCGGAGCTGCGAGGTTTGGAAAATTCCACCTACCACGGTAAGAGCGGTGAGCTGCACGTCGAGTTTCCAACGTTCCGCACCAATCTGGCGCGCACCTTCGTGAATGGGGCCCGAGAAGCCGGCCACCGCAAGCTCGACTACAACGGCAAATCGCAGCTCGGCGTCTCGTACGTCCAGACGACGGGGCTGCGCGGCATGCGCCAAACCGCGTACCGTGCGTTCGTTGAACCGGTGCTCTACAAACGGCCCAACCTGCACGTTCAGCCCTACAGCCAGGTGCTGAAGGTACTGATCAACCCCGACACACAGACGGCCTACGGTGTGACGTACACGCGACACTTCCGCAACTACGAAGTGCGCGCACGCAAGGAGGTGATCGTAACGGCGGGCAACATCAACACCGCCCAGCTGCTGCTGCTGTCCGGCATCGGACCGAGGGAGCATCTGCAAAATTTTAACCTGCCGCTCGTAAGCAATCTCCCGGTGGGTCAATCGTTCGTCGACAGTCCCGTTTTCAACGGGTTGACGTTCGTGCTGAACGAAACCGGGCAAGCGCTGCTGACGGACAGTCGCTTCCAGCTGCGTTCGCTCGGTGATTACTTCCGGGGCGAGGGCCCCCTGACCGTCCCTGGCGGTGTGGAGGCCATCAGTTTCGTGCGCACCGAGAACGCTACCACCGAACCGGGCGTGCCGAATATCGCGATCGTTTTCTCCACCGGGTCGCTCGTCTCCGACGGCGGACTGGGTTTGCGCAAGGGCAAACGCATCAAGACGGCCATCTACAACAAGGTGTACCGCCCGCTGGAAACGCTACGCAACGATCAGTGGACGGCCAGCGTGGTGCTGTTGCATCCGGAATCGCGCGGACACCTGAAGCTGCGCAGCATCAATCCGTACAGTGCGCTCAAGATCTATCCCGGCTACTTCGGCGCGGATCGGGACGTGGAGACGATGCTGGAAGGCATCAAGGAGGCGGTACGCATCTCGAAGTCACCCGCCATGCGGCGGTACGATGCGCGCGTCCTGGGCATCCCGCTGCCGAACTGTGAGCAATGGGACCAGCGGGAGGACGAATACTGGCGCTGCGCGATACGGACGCTGTCCAGCACGGCCTACCAGCAGCTGGGCAGCTGCCGCATGGGACCGGCCGGCGATCCGCTGGCGGTCGTAGCGCCCGACCTTCGCGTGCACGGCGTGCAAGGTTTGCGCGTGGCGGATGTGAGCGTGGTGCCGACCACTATTTCGGCGCAGTCGGCCGCAATCGACTACATGATTGGCGAACGGGCCGCAGACATCATTAAGGACCAGTGGGAGCAAGGGTCCAGTGCGCCCACCTCGTCAAGCGACCGCTAG>AgGMC_K1ATGGCTGCACAGCGCACGTCCGCCAGCACCGTCGTCCCCACCGACGCCGCCACTCAGGAGAGCTATAGGACAGTGTATGATTTCATAGTTGTTGGTGGTGGTACTGCTGGTTCGGTCATCGCTAGCCGGCTTGCCGAATTACAACAGTGGCACATTTTGCTAATTGAAGCTGGTGGTGGCCCAAGCGATAAGGATCTAAGCTGGAATCTTCAGGCCCAGCGGCAGATGGGCTCTTGTCTGGGTGCTCCAGAGCAGCGGTGCGAAATACCGACCGGCAGAGGATTGGGTGGCAATACGCTAACCAATAATATGCTTTACGTCAGAGGCAGTGAAGCAGATTATGATGCGTGGGCAAAACAAACAAATGTGGACTGGTCGTATCGGAACGTGCTTCCGTATTTCTTAAAACTTGAAAATTTTCGCAAAAATGCTTCTTCAACCTCACGGCAGCAACGTGGAAAAGGTGGACCTGTTCCGATAGCGGGACTACGCGAAAAATCACCTTTGGTACGTTCTTTCATTTCTGCCTGCAACCGACTCGGGCTACGTACGACCGATTACAACGCGGAAAGGAACCAAACCGTTGGGTTTGTTCAACTAACACAATACCGCACCAAGCGCATCACTGCAGCTGACGCATATATTCGGCCCGTGAAACAGTTGTTCAACAATCTTCACATCATGTCATCGGCACGGGTCACAAAGGTGCTGATAAACGGTATGAACCGGCAAGCGGTAGGAGTCAAAGTGCTTGTTAACGGCAAACAACGAAAACTACGGGCTACCAAAGAGGTCATCCTTTCGGCGGGACCTATATTTACACCACATCTGTTGCTGTTGTCTGGAATTGGCCCCCGAGCGCAGCTGGACGCATTGCAAATTCCGGTACTTGCAGATCTTCCCGTAGGAGCGACAATGAACCTTCGATTAGTATCGTTTCCGCTGCATCTCGCAACCAATCGAACAGTGCCATACGCAGCGCAGAAAATGATAGAAGCAATAGCATTTTTAAACACGACCAAACAGAACAATACTGATCCTACACACGAGATTCTGTTCCAGTACGAACCACGCGGTACACTGGAATATTTTTCACTCGGGCTAATTCACCTTCGACCAGCGTCGAGAGGATTCGTGCAGTTAAACGCCACAAACCCTTCACGTAATCCCGTGGTTTACACCAATTTTTTCAGCGCCCCAAACGATATGGAGGAAATTTTGAGCGGCATTACCGAATGTCTTAAGATTGTTCACAGCGAAGAGTTTACAAAGCTTGGCCTACAGTCCAGAAAGCTGATCGTTCCCCCTTGTGATAAGCTTCGGTACGGCACGGACGAATATTGGCGCTGCGTCGTACGTCACGTAGGCCATGCAGCCGATCAACCGTACGGAACCTGTCCTATGGGAAGGCAAGATAATAGACAAGCAGTGGTATCACCGGAGCTAAGAGTACACGGCATTGGGAATCTACGAATAGCCGATGCAAGTGTAATGCTTCCTGTTTCCAATGGCCATACCCAGGCGACGGTGTATATGATAGCTGAAAAAGCGTCCGATTTGATCAAAAGCTCTTGGGACTGGGGCAATGAACTAGAACGGCGCCGCTAG>AgGMC_I3ATGGGTGTTTTGCAGGACTTACTACGAGTGCACGATGGCAATGGGCGTTTGTTGTTTCTAGTGTTCTTGTGCTTGTACCTTACGGTACGGTGCTCGGTGTGCCAGTGCCCGGATACTGGTGGGTTAGGTGCGGAAGATCCGGCAAATGTGCGATTGCTGCAGGAAAACAGCATCAAGCAAGCTTCTTTGCTGAAGAAATATGACTTTATCATCGTCGGTGCTAGTCCTTCGGGTTGTTTGCTGGCGAATCGGCTAACTGAGATTCGCGATTGGAATGTGCTGCTCATAGAGGCCGGTGAGCAGGAAAATTTGTTCGTCCAGGTGCCTATTTTTTCGGCGTACCTGCAATCTACCAGCTATAACTGGGGATACCTCGCGGAACCGCAGAACTACTCTTGCTGGGGGATGAAGGATCAGCGCTGTAGCTATCCGCGTGGTAAAGGTCTTGGTGGTTCGACACTGATCAATTACATGATGTACGTGCGCGGAAACAAGTACGACTACGATCAATGGTCAGCGGCCGGGAATGATGGTTGGTCGTTTGACGAGATTCTACCGTACTTTGTCAAGTCGGAAAAATCGTACCTACGCGAGGTGAACCGATATCACGGCATGGACGGCAATCTGGATGTGCGGTACCTTCCCTATCGCACCCGGCTGGCCAAGCTGTTCGTGAATGCGTGGCGTGAGCTAGGTCTGGAAAGTGTGGATTACAACGGCGAGTCGCAGATCGGTGTGTCGTACATACAGTCAAACGTGCGCAACGGCCGCCGGCTGACGGCGTACACCGCTTTCCTTGAACCGATACTCGATCGGCCAAATTTGCACATACTTACCAATGCGCGTGCCACGCGGGTGCTGATCGACGCGACTACCCAGCAAGCGTACGGGGTGGAATTTATTAAGGACCGAAATCGGTACACGGTGTACGCGGATAAGGAAATTCTGATGACCGCGGGTGCTCTGCAGACTCCGCAGCTGCTGATGCTGTCCGGTGTTGGGCCAAAGGAGCACCTGCAGGAGGTAGGCATACCCGTGATAAAGGACCTGCCCGTCGGCCAGACGTTGTACGATCACATCTACTTTACCGGGCTCGCGTTCGTGACCAACACGACGAACCTTTCACTGCACGGTGACAACGTCCTTACGCTGGACGCCTTTCTGTCGTTTCTGCAAGGCCAGGGTCCGATGACGGTGACGGGCGGTGTCGAGGCGGTGGCCTTCATTCGTAACACCACAAATCCTGAAAGTGCGGCCACTCCGACCGTGCTACCTAACATCGAGTATATACTCACCGGCGGTTCACAGGCCGCCGATCACGGCAGCGGCATCCGCAACGGGTTCCGGCTGACGGACACCATTTACAGCATCTATAAACCGCTGGAAGCGAACGAGCGAGACGCAATGACGGTTAATATTGTGCTGCTGCACCCGAAGTCGAAGGGGTACATGCGCCTGAAAAGCTGCAACCCACTGCACTGGCCGCGCTTTTACTCGAACATGCTGAAGGAGCAGGAAGACGTGGAAACGATTCTGCAGGGCATCCGGTCGGCGTTGCCGCTGATGGACACACGGGCTGCCCGCCGTTACGGTGCGAAACTGTACGATGTACCGTTGCCAAACTGTGCCAGCTTTCGGTTCGGTACGGATGATTACTGGCGTTGTGCCATTCGTACGCAGACGACCTCAATTCACCACCAGATAGCGACGTGCAAGATGGGACCGCCGAGTGATCCGGACGCCGTCGTATCATCCAATTTGAAGGTGTACGGTGTGCGGCGGTTACGGGTGGCCGATGTGGGAGTCATACCGTATCCGACCAGTGGACACCCTACCGCTACGGCGTACATGATCGGCGAGAAGCTGTCCGATTTGATAAAAAACGAATGGTTAGGGCAAAACATCCCTACCGGTAGTGGTGCTGGTGGTATCTAA>AgGMC_Q4ATGCAGTACCTACCACTTGCGGCCGGTATTCTGGGGATGGTGAGCTTTAGCAGACCCCAGGATAGTCTGCTGTCGATGCTCAGCTTCCTGCAGGATGGCGGCGAGCGTATGTCGCACGAGCTGCCAAGCCAACCGGTCGTTCGGCCAGAATATGATTTCATCATTGTCGGAGCCGGATCGGCCGGCAGTGTGCTGGCAAATCGGCTAAGTGAAGTGCCGGACTGGTCGGTGCTGCTGATCGAGGCAGGACCTGGTGAGAATTTGCTAATGGACATCCCGATGGCGGCCCACTATCTGCAAAACTTCAACATCAACTGGGACTATCGAACGAAACCGAGCGACCAGTACTGTTTGGCGTTCAAGAACAATCAGTGTCGCTTTCCCCGTGGCAAGGTGATGGGTGGCTCGAGCGTACTAAACTACATGATCTATACGCGGGGCAATCGGCGCGATTTTGATCACTGGGCCGACCTGGGCAATCCGGGCTGGTCGTACAAGGAGGTGTTGCCGTACTTCAAGAAGCTCGAGCACAGCGTCGTACCAGACGCGAACCCGGCGTACGCGGGCAAGGATGGTCCGCTTACCATTTCCTATCCACGGTTCCGCTCGGACACGGCGAAAGCGTTTGTGCAGGGCGCAATAGAGGACGGTGCCCCATACGTCGACTACAACGGGCCGACCCAGATCGGTGTCTCGTACATCCAGAGCACTACGAAGGATGGCAAACGGGACAGTACGAACGTGGCGTATCTGTACGATATGCGCAACCGCTCGAATCTGCACGTGAAGAAAAACAGCCAGGTGACGAGAATCCTGTTCGACCGCAGTGCCAACCAGGCGAATGGTGTCCGATTCTTTCACGCCGGCCGTTTCCACACGGTTCGCGCGCGTCGAGAGGTGATCGTTTCTTCTGGCGCTATTGGTTCGCCCCATCTGCTGATGCTTTCCGGCATTGGACCGGCCGACCATTTGCGCGCAAACGGCATCAAACCGATCGCCGATCTACCGGTGGGGCACAACTTCCAGGATCATACCGCGGCCGGTGGTCTCACCTTTCTGGTGAACAATACCCAAACGCTCACCTACAAGAACGTGTTCCGGCTGGACAACTTCATGAAGTATCAGTACGACAAGCGTGGACCATTCACGTCCACTGGCGGGTGCGAAGCCATCGCATTCTACGACTCTGAGCGTCCGGGTGATCCAGACGGATGGCCCGATTATGAACTGCTGCACATCGGTGGTACGATCGGGGCCGATCCCACCTACGAGGTGAACTTCAACTACAAGCACAAAACGTTCCAAACCCTGTTTGGCGAGATTCAGCGCCGGAACTACGATGGGTTTACGGTTTTCCCATTGATCATGAGACCGCGCAGCAAGGGACGCATCTCGCTGAACGGTTCCAGCCCGTTCCAGTATCCGATCATCGAGCCGAACTACTTTGACGATCCGTACGATCTGGACATTTCGGTGCGTGCCATCCGGAAGGCGATCGAGCTGAGCCGGACTGGCGCGATGCAGCGGTACAATGCCCGCCTGCTAGATATTCCGATGCCGGGCTGCGAACATTATCGCTTCGATTCGGACGACTACTGGAAATGTTTTTCGCGCCACGCCACCTTCACGATCTACCATCACGTGGGTACGTGCAAAATGGGACCGAGAAAGGATCCTACCGCGGTGGTGGATGCGAGACTGCGCGTACACGGTGTGAAGGGTTTGCGAGTGATCGACGCAAGCATCATGCCGGATGTACCGGCGGGCCATACGAATGCGCCCACTATCATGATCGGCGAAAAGGGTGCCGACATGATCAAGCAGGATTGGAACGAGCTTACGTAG>AgGMC_Z1ATGGTATTCAATGTACTGATAGCATCGTCTGTGATTAAAACGGCCACCGTCGTTGGTTCTAGTCTATGGCTCATCCCGTTTTTGCTGGGTGCCATCTCCTACTATCGCTACGATCGCGTCGATCCGGAATCGCGCGTCATCAACCAGGAAGCGTTACTGCCGGAGTATGATTTCATCGTCGTTGGCGGCGGCTCGGCCGGTGCAGTCGTTGCTAACCGGCTGACAGAGATCCACCGCTGGAAGGTACTGTTGCTGGAGGCGGGCCCAGACGAGAACGAGATCTCGGACGTGCCGTCGCTGGCAGCGTACTTGCAGCTGAGCAAGCTCGACTGGGCGTACAAGACGGAGCCAACGAACAAGGCATGCCTGGGCATGGTGAATAATCGGTGTAACTGGCCGCGCGGCAAGGTGCTGGGTGGATCATCCGTGCTCAACTACATGATATACGTGCGCGGCAACCGAAATGACTTTAACCATTGGGAATCCCTGGGCAATCCGGGCTGGGCGTATGACGATGTGCTACAGTTCTTTGTAAAATCCGAAGACAACCGTAATCCATACCTCGCTCGCAATCCGTACCACGGGCAAGGGGGACTGCTCACCGTGCAGGAAGCTCCCTGGCATACACCGCTCGTAGCGGCGTTCGTGGAAGCTGGCACTGAAATTGGGTACGAAAATCGGGACATCAATGGTGAACGACAAACCGGCTTCATGATAGCGCAGGGCACAATCCGACGAGGAAGCCGCTGCTCTACCGCTAAAGCCTTTCTGCGCCCGATACGGCTTCGCAAGAACCTGCACATTGCGATGAACTCACACGTCAGCAAGCTGGTCATCGATCCGGAAACGAAGCATGCGGTTGGCGTTGAATTCTTTCGGGGCGGTAAGCGACACTACGTGCGTGCCCGGAAGGAAATTATCATGTCGGCCGGCTCGATCAACACACCGCAGATATTGATGCTGTCGGGTATTGGACCGCGGGCTCATCTCGAGGATGTGGGCATTACGACTATTCAGGATTTGCCGGTTGGCGAAAATCTGCAGGATCACGTCGGGATGGGTGGCTTAACGTTTCTAGTGGACAAACCCGTAGCGATTCTACAGAACCGTCTCGAGGCGGGCTCTGTGACAATGAACTATGTGATTAACGAACGCGGCCCGATGACCATACTGGGTGGGCTGGAGGGCATCGCGTTCGTTAACACGCCCTTCGCAAATGTCACTGATGACTGGCCGGACATTCAGTTCCACATGGCGCCGGCCTCACTCAACTCCGACGGTGGAGCCCGTGTGAAGAAAGTGCTTGGCCTGCGGGAGGACCTTTACAAGGAGGTATTCCACCCGATCGAAGACACGTACAGCTGGACGATAATGCCACTGTTGCTGCGGCCCCGGTCCCGCGGTTGGGTACGTCTTAAATCCAACAACCCTTTCCACTACCCACTCATGAACCCGAACTACTTTGAGGATCCGTTCGACGCTGCCACCCTCGTTGAGGGTGCGAAGATAGCGTTACGCGTTGGGGACGCCAAGGTGTTCAAGCAGTTTGGTAATCGACTGTACCGCAAACCGCTACCAAACTGCAAGCAACATAAGTTTCTATCGGACGAGTATCTTGACTGTCAGGTGCGTACGATATCAATGACCATCTACCATCCGGTGGGGACGGCCAAAATGGGGCCACATTGGGATCCCGGCGCGGTGGTTGATCCACGGCTGCGCGTTTACGGCATATCTGGACTGCGCGTGATCGATGCTAGCATTATGCCAACTATCGTAAGTGGCAACACGAATGCGGCTGTCATTATGATCGGTGAGAAGGGAGCACACATGATCAAAGAAGACTGGCTAGGACACGATCGATGA>AgGMC_D1ATGGTGGTCTCCTTTGGAACACTCATTCCGCTGTTGGCGGGAGCGGCACTGAAAGCCACACCTGCCGCGGCCGGGCTAACAACTGCCGTTGGGGCGGCCATTAGTGCCGCGACGGCCGTTATCGGCGTCGGCAAGCTGGCGATTGTGCCGATCCTGATCGCTTCGCTCGCCTATTACAACTACGATCTGTTTGATCCGGAGAATCGGCCGTTCAATGTGCCGGAGGTGGACCGTGAGTACGACTTCATCGTGGTAGGCGCGGGTTCGGCTGGTGCTGTTGTTGCCTCGCGCCTTTCGGAGATAGGCGGCTGGAAGGTGCTGCTGCTTGAGGCGGGTGGCCACGAGACCGAGATCTCGGACGTTCCCATCCTGTCACTTTATCTGCACAAAAGCAAGCTGGATTGGAAGTACAGAACCCAGCCACAGAAGACGGCATGTCAAGCGATGAAGGATAACCGATGCTGCTGGACACGAGGCAAGGTCCTCGGGGGCTCTTCGGTGCTTAACACGATGCTGTACATACGCGGTAACAAGCGCGATTTCGACCTGTGGCAGGCGCTAGGAAATCCCGGTTGGGGCTACGAGGACGTCCTGCCGTACTTCCGCAAGTCGGAAGACCAACGGAATCCCTACCTGGCACGCAACAAACGACAGCATGGTACTGGTGGGCTGTTGCAAGTACAAGATGCTCCATATTTGACTCCACTCGGTGTATCCTTCCTGCAAGCTGGCGAGGAGATGGGCTACGACATAGTGGATGTAAACGGTGAACAGCAGACTGGTTTCGCGTTTTTCCAGTTCACGATGCGCCGTGGTACGCGTTGCAGCACTTCCAAGGCTTTCTTGCGCCCGGTACGCAACCGTAAAAATCTACACGTCGCCCTGTTTGCCCACGTCACGCGCGTTATACTAGATCCGGAAACACGACGCGCACTAGGCGTAGAATTTATTCGAAACGGAAAAACGCACAAGGTGTTTGCCACACGTGAGGTGATTCTTTCGGCAGGAGCGATCGGTACACCCCATCTGATGATGTTGTCGGGAATTGGGCCGAGGGAGAACCTAGAGCGCGTAGGTATCCCTGTCTTTCACGATCTACCTGGTGTGGGACAGAATCTGCAGGATCATATCGCTGTTGGTGGTTTGGTATTCCGTATCGATCAACCGATTTCGGTAATTATGAACCGTTTGGTGAATCTCAATTCGGCACTTCGCTACGCTGTCACCGAAGATGGGCCACTGACCAGCAGTATCGGGCTTGAGGCAGTCGGTTTTATCAGTACTAAGTATGCGAACCAGACGGACGACTGGCCGGATATAGAGTTTATGTTGACGAGTGCATCGACACCTTCGGACGGAGGCGATCAGGTGAAAAAAGCGCACGGATTGAAGGATGAATTTTACGAGGATATGTTCAGCTCGATCAACAATCAGGATGTATTTGGCGTGTTTCCGATGATGTTGCGTCCCAAAAGCCGGGGCTTCATTCGGTTGCAGTCTCGCAATCCGTTGCGTTATCCTTTGCTTTACCACAACTATCTCACCCATCCGGATGATGTGGGCGTGTTGCGCGAGGGCGTAAAGGCTGCGATAGCATTCGGCGAAACACAGGCCATGAAACGGTTTGGCGCTCGCTTCCACAGTAAACAGGTGCCGAACTGTCGCCACCTACCGGAGTTTACCGATGAGTATTGGGACTGCGCCATTCGACAGTACACGATGACTATATATCACATGTCCGGCACGGCAAAGATGGGTCCCCCAGATGATCCTTGGGCAGTTGTCGATCCGAAGCTACGCGTCTACGGTATTAAGGGGCTGCGAGTTATAGATGCTAGCATAATGCCGCGCATCACAAGCGGTAACATCAACGCACCGGTCATCATGATTGGAGAGAAGGGTGCCGATATGATCAAAGAGCTGTGGTTGAAGAAGGGTCACTCACGGCGAGGGAAGCGTCAACAGTTTGCGAACGAAACGCTTAGTGCCGCCAACCAAACGGAAGCAATAGAAGCGGTGGGCGATAGTAGTGCGGCACCTTGTGCAAACGAAACCTTTGTTAGCTAA>AgGMC_B4_iso4ATGGAGGCCTTAATGGGAGGCCAGTGCGCCGCACAGAGCGTCGGACCGGCGAATCAGCTGTTTGGGCTGCTGGTGCAAACAATCCTGGCGGCACAGTGTGCGATTTCCCCACCGGATATGTGGCCGAAGGACTACGGCCCGACAGCACTGCAGCGCGGATTGGATGAGTACGATTTCGTGATCGTCGGTGCCGGTTCCGCCGGGTCGGTTGTTGCCAATCGACTGTCCGAAAACCCGGACTGGAAGGTTCTGCTGCTAGAAGCGGGCGGTGATCCACCGATCGAGTCTGAGGTACCATATCTGGCATTCGCTTTGCTTAACGGTTCGCATGTCTGGAATTATTACGCCGAAAGGAGTGACACAGCTAGCAAAGGCTACAAGCGCGGAAGTTACTGGCCGAGGGGTAAAATGTTGGGTGGATCAAGCAGTAACAATATCATGCTGTACGTGCGTGGCAATAGCCGCGATTACGATCGCTGGGAGGAGCAGGGTAATCCGGGCTGGGGTTGGAAGGATGTGCTGGAGTATTTCAAAAAGTCCGAAGATAACGGGGCGCAACATTTGCTGCAGGAGAGAGCCGACTACCACGCACAAGGTGGTTTACTCAAGGTCAATTCTTTCATGTCAAACGACATGACTAAACTAGTGATAACTGAGGCGGCACAAGAGTTGGGTATACCGGAGATTATGGACATCAACAGTGATGAGTATATCGGTTACAATGTAGCGCAAGGCACGGTCCACAAGGGAAGACGCTGGAGTACAGCAAAAGCATTTCTAAACACCGCAGCTGATCGACCCAATCTGCACATCATTAAAAATGCTCACGTGACCAAGATCAACTTCGAGGGGACTGCAGCGACAGGCGTGACGTTCGATGTGCCCTCCCAGACGGGTGTTTCAGCAAGCATACGCAAAGAAGTGATTATATCGGCTGGTGCAATAAATACACCACAAGTTTTGCAGCTTTCCGGACTGGGAGCAAAGGAGCAACTCGATCGGCTAGACATCCCGCTGGTTAAGGAAATACCGTCAGTGGGGGAAAATTTGCAAGATCATTTGATTGTGCCCCTGTTTCTGAGTCTGCACGGTTCACGACCGATCGAACGCAGCATGGATGAACTGCTCGACAGTATCTACAGTTACTTCCGTTACGGGCTTGGTACATTTGGTACCGTCGGTATAACAGATCTTTTAGCGTTCGTCAACACACAAAGCCCGGCCGCCAAATTCCCGGACATACAGTACCATCACTCGCTCATTCTTTGGAAAACGCCCGATATAGCACGACTCACTCAGTGTTTCGGCTGGGAAGACTACATTTCCCATCAAATAATTGAGCAAAACCAAAAGTCCGAAATCCTTATGGTGATGGTAACACTACTCAATCCCAAATCGAAAGGAAATGTGCAGCTTCGCTCGTCCAACCCGTACGATGCACCAATCATCAACGCTAACTACCTCGACGACCAGCGGGATGTAAAAACGATCATTCGTGGCATTCGGTTCTTCCGCAAACTTTTGGATACGGAAAATTTCGGCTATCATGAGCTGAAAGAGTTCCACCTGAAGATTGAGGAATGCGATCGGTTGGAGTACGAATCAGATAGCTACTGGGAGTGTTACGCCCGATACATGTCCTCTACTATCTACCATCCTACGGGTACGGCCAAAATGGGTCCCAATGGAGATCAAGCGTCCGTTGTCGATTCTAGACTGAAGGTGAGAGGGGTACAAAATTTACGCGTAATAGATGCAAGCATTATGCCCGACATTGTGAGTGGCAACACAAATGCACCGACTATCATGATCGGTGAAAAGGGTGCCGATATGATAAAGGAGGATTACGGAGTAGAGAAGAAGGAAGCAGCTACACACACAGAACTCTGA>AgGMC_G2ATGAACGCGGTTGGTAGCTACGAAGTGCGGACGCGGCTGCTCTATACGTCGCGAATCGGAACCGTGTTTTTGCTACTGATTGATGCAAGCATCTGGCTGCAGCGACCCGACATTGTAGACTTCCACCACCGTGTGCAGCCCATTCCCGGCCCGTTTGTGCAGGACATCTACGACTTTGTGGTGGTGGGAGCTGGATCTGCCGGTGCCGTGATGGCCGCACGGCTCTCCGAAATCTGCCACTGGGACGTACTGTTGCTGGAGGCCGGCACGGACGAATCGTTCCTGACCGACATACCGTTCCTGTATCCGACGCTGCAGACGTCGCGCGTCGATTGGAAGTTTCGCACGGAACCGTCCGATCGGTTCTGTCTCGCAATGAAAGATCAGCGGTGCCGGTGGCCACGCGGCAAAGCACTCGGCGGCAGCTCAACGATCAACGCAATGCTGTACGTGCGCGGCAACCCGCGAGACTTTGATGCCTGGCGTGACCTGGGCAATCCGGGCTGGAGCTACGACGATATGCTGCCGTACTTTCTCAAGCTGGAAGATATGCGCGATCCACGGTACGCCAATCTATCGTACCATGGGCGGGGTGGTCCGATCAGTGTGGAACGATTCCGTTACCATACGCCGCTGCGCAACCATCTGCTGGCGGGGCTGGAGGAGATGGGCCTTACCAACCGGTACGGCGAAGTGAATGGGCCGATGCAGAGTGGTTTTGCCGTACCGCACGGTTCCATCCGCAATGGGTTACGGTGCAGCACGGCGAAGGGTTATTTACGGCCGGCAGCGGCGCGTAAGAATCTTCACATATCCACCAAAACGATGGTCGAGCGCGTTTTGATCGATCCAAACGATCGGCGCGCCTACGGTGTACAGTTCGAGAAGGGTGGCCGACGGTACCAGGTGATGGTGTCGAAGGAAGTGATTCTATCAGCCGGTGCGCTTAACAGCCCGCAGCTACTGATGCTGTCCGGAATAGGACCAAGGCAGGAGCTCGAACGCCATGGCATTCGTGTGATCCAAGATCTGCCCGGCGTAGGACAGAACATGCAGGATCACGTGGCAACCGGTGCGGGGGGCTACACGATACGACCACCACCAGGCAGTCCCCCGCTAGCGTACGATTTTGGCGACGCAGTGGGCGTTGATACGCTGAGACGGTTCCTCTTTAACGAGGACGGCATGCTGTACGGTATGTCGCTGTGTGAGGTTATGGGTTTCCTGAACACCAAGTACCAAGATCCGGAACTCGATTGGCCCGATGTAGAGCTGTTTCTCGCCAGCCTGTCCGATCTAACCGATGGCGGACGGTTCGGGAAGCGAGGTTCGGGTATCAGCAACAACTACTACGCGCAGGTGTACGAGGAGCAGGTGTATCAAAACTCGTACATGGTCATACCAATGTTATCGCGCCCGCTCAGCACCGGATGGTTGGAGCTGGCAAGTAAAAATCCACATGATCACATCCGTATCCATCCCAACTATTTTGACAACCCGAAGGATATGATGGTACTGATCGAGGGTCTGAAGTTCGCCGAAGCCCTTGCTAATACGACGGCGATGCGCAACATCAACGCTACCCTGCTTGATTACTCTAGGTCAGCCTGCCGAGCGAGTAATTTTCCCAACAAAGACGATTTCTACACCTGTCTGGTGCGACACTACACCCAGACAATCTATCATCCGTGCGGCACGGCCAAGATGGGCCCGGTCACCGATCCAATGGCAGTGGTTGATCGGTTTCTACGCGTGCACCACATCGGCGGACTACGCGTAGTTGATGCGAGCATCTTTCCTGTTATTACCACGGGAAACACGAACGTGCCTACGATTGCGACCGGAGAGAAAGCGGCGGATCTCGTTAAGGCAGCGTACGCAGCCGATCTACGAGCGCACGCCGACACGCTGCGGGAGTGTAAGACACTGCACACAGACTATTCTGCCAAAGCGATGGAGGAGAGCCAGGCAGTATGA>AgGLDATGTCGTCCTGTGCGTGTCCTATGACGAGCCCCGTCGGGGCCACGCTGGCCGCACTGTGCGGCGGTACGCAGTACATGCTGTTCATGGGACTGCTGGAGGTGTTCATAAGGTCACAGTGCGATCTGGAGGATCCGTGCGGACGGACCAAGGCAAAATCATCCCGAAATGTGGACTACGAGTACGACTTCATTGTGGTTGGCGGTGGATCGGGCGGTTCGGTAATCGCGTCCCGGCTCTCGGAAATCAAGAACTGGAAGGTGCTACTGATTGAAGCGGGACCGGATGAACCGACGGGCGCCCAGATCCCGTCCATGTTTCTGAACTATCTCGGCAGCGACATCGACTGGAAGTTTAACACCGAGCCGGAACAGTACGCCTGCCTGGGGTCGCCCGAGCAGCGCTGCTACTGGCCTCGGGGCAAGGTACTCGGCGGCACGTCCGTGCTGAACGGCATGATGTACATCCGGGGCAACCCGCAGGACTACGACGACTGGGACGCGATGGGCAATCCGGGCTGGAAGTGGAAGGACGTGCTGCCGTACTTCATGAAGTCGGAGGACAACCTGCAGATCAACGAGGTCGACTCGAAGTACCATTCGACGGGCGGCATGCTACCGGTTGGGCGGTTCCCGTACAACCCGCCGTTCTCGTACTCGGTGCTCAAGGGCGGCGAACAGCTCGGCTACCAGGTGCAGGATCTGAACGGTGCCAACACGACCGGCTTCATGATTGCGCAGATGACGAACAAGAACGGCATCCGGTACAGTGCGGCGCGTGCCTTCCTGCGCCCGGCCGTCAACCGGGCCAACCTGCACATCCTGCTCAACACGACCGTCACGAAGGTGCTGGTGCATCCGACCTCGAAGACGGCGCACGGTGTCGAGATTGTCGATGAGGATGGGCATATGCGCAAGATTCTGGTAAAGAAGGAGGTGATCGTGAGCGGTGGAGCGGTCAACTCGCCCCAGATACTGCTGCTGAGCGGCATTGGACCGCGCGAACATCTGGAGAAGGTGGGCGTGCGTCCGATACACGATCTGCCGGGCGTGGGCAAGAACCTGCACAACCACGTCGCGTACTTCATCAACTTCTTCCTGAACGATACGAACACGGCACCGCTGAACTGGGCGACGGCGATGGAGTATCTGCTGTTCCGCGACGGGCTCATGTCGGGGACGGGCGTGTCGGCCGTGACGGCCAAGATCAGCTCGAAGTACGCGGAGCGACCGGATGATCCGGATCTGCAGTTCTACTTCGGCGGGTTCCTGGCTGACTGTGCCAAGACGGGGCAGGTCGGTGAGCTGCTCAGCAACGATTCCCGCTCGGTGCAGATCTTCCCGGCCGTGCTGCACCCGAAGAGCCGCGGGTACATTGAGCTGAAGTCGAACGACCCGCTGGAGCATCCGAAGATTGTCGTGAACTACCTGAAGGAGGATCACGACGTGAAGGTGCTGGTGGAGGGCATCAAGTTTGCGGTGCGGCTCTCGGAAACGGACGCGCTGCAGGCGTACGGCATGGATCTGGACCGAACGCCCGTCAAAGCGTGCCAGGATAAGGACTTTGGAAGTCAGGAATACTGGGAGTGTGCCGTACGACAGAACACCGGTGCGGAGAACCATCAGGCCGGTTCGTGCAAGATGGGCCCGACCAGTGACCCGCTGGCCGTGGTCGATCACGAGCTGCGTGTGCACGGCGTGCGGAATCTGCGAGTCGTCGACGCGTCCGTCATGCCGAAGGTGACCTCGGGCAACACGAACGCACCGATCATCATGATCGCCGAGAAGGGTGCCCATCTGATTCGCCGGGCGTGGGGCGCTCGCTAG>AmGLDATGAGCTGTAACTGTCCATTAAACCCATCGACTGGACCAACACTGGCATCAACATGCGGTGGTTCGTCCTTTATGCTATTCATGGGTCTTTTAGAAGTATTTCTTCGTAGTCAGTGCGACCTCGAAGATCCGTGCAATAGACCTCTACCACCTCCTACCGTTAATTCCAGGTATGATTTCGTAGTTATCGGAGGTGGAAGTGCAGGTGCAACAGTTGCATCACGATTATCAGAGGAACCACGATTTTCGGTGTTATTGTTAGAAGCTGGATTAGATGAACCCACCGGGACGCAGATACCTTCGTTCTTTTTCAATTTCATTGGGACTGACATCGATTGGCAGTATAATACCGAAAGTGAAGATACTGCGTGTCTAAATAAAGATGATCGAAAATGTTATTGGCCCAGGGGGAAAGTATGTGTTCTGGGTGGAACCAGCGTTATGAATGGCATGATGTATATAAGGGGCTCGCGAAAGGATTACGACGACTGGGCGAGGCTAGGTAACATAGGATGGTCTTATCAAGACGTCCTTCCATATTTCATCAGGAGCGAGGATAATCTTCAAGCAAACACCATGGATTATGGTTATCACGGTGTTGGTGGGCCACTCACGGTCACGCAGTTCCCTTATCATCCGCCCTTGAGTTATTCTATTCTCGAAGCTGGAAAAGAACTTGGTTATGGTATCGCGGATCTAAATGGACGAACTCACACTGGATTCGCTATAGCCCAAACGACCTCCAGAAATGGTTCACGACTTTCCACTGCCCGGGCATTTTTACGACCAGCTAAAAATCGTCCAAATCTTCATATAATGCTCAACTCCACGGCCACTAGAATATTGTTTGACAATAACAAAAGGGCAGTTGGTGTAGAATTTGTTCATGATGGAAAGATCCATCGAGTCTCTGTGGCTAAAGAAGTAGTCATAAGTGGAGGTGCCGTTAATTCACCACAAATACTTTTGAACAGTGGTATCGGTCCTCGGGAAGAATTGAATGCTGTTGGTGTTCCGGTTATTCATGATTTACCTGGTGTAGGTAAAAATCTTCACAATCACGTCGCCTATACGCTGGCCTTCACTATCAATGATACTGATACGACTCCCCTTAACTGGGCAACTGCAATGGAATATCTTCTCTTCAGAGATGGATTGATGTCTGGTACAGGTATTTCCGAGGTAACAGCGATGATAAATACGAAATACGCGAATCCAAAGGATGATCATCCAGATGTTCAGTTGATATTCGGTGGATACCTAGCAGACTGCGCAGAAACAGGTATGGTCGGTGAAACGAAGGGTAATAACCGAACCATTTACATCATTCCAACATATCTTCATCCGAAGAGTCGTGGTTACCTTCGTCTACGAAATAACGATCCTCTTTCGAAGCCGTTAATCTACCCGAAGTATTTGAGCCATCCTGATGACGTAGCAGGCCTTATAGAGGCTATTAAATTCAGTATCAGGTTATCTGAAACTGAGGCTCTCAGCAGATATGGATTTCAATTAGATCGTACACCTGTGAAAAATTGTGAACACTTAGAATTCGGTTGCGATGCTTATTGGGAATGCGCTGTAAAACATGATACTGCACCAGAAAACCATCAAGCAGGTTCTTGCAAAATGGGCCCACCGGACGATCCTCTCGCTGTAGTAGACAATCAATTAAGAGTCAGAGGAGTGAGAGGTGTCAGAGTAGCGGATACTAGTATCATGCCAAGGGTTATTTCCGGTAACACAAATGCGCCCGCCATTATGATAGGAGAACGCGCCGCAGACTTCATCAAGAGGACCTGGGTCGGCTGA>Am_CG6142like_3ATGTTAAAATTCTTGATAATCGCCTTGCTTCCGTCCTCCGTGCAATCCATTATTCCTCCCGCGATTCTGAAAACTATTTATTTATTTCTTTTCGGACTTTTGAAAGGACAAGACGACTCGATCCCCGATCAAACGCGCTTCTCCCAGGAATACGATTTCATCGTAATCGGGGCTGGTTCGGCTGGTTCCGTTTTGACGAATCGGTTGACGGAAAACCCGCAGTGGAACGTGTTGCTACTCGAAGAGGGCAAAGACGAGATTTTCCTCACGGATATACCATTGCTTGCTCCGGCTTTGCACGTCACGGACTACGTCCGTCTGCACACAAGCGAGCCGAGGCCCCGAAATACCGACGGAACCGACGGTTACTGTTTATCGATGAAGAACGGCCGTTGCAATTTGCCAGGAGGCAGGGCGGTCGGTGGTAGCTCCGTGGTGAATTTCATGATATATTCGAGAGGATCGCCGAACGATTACGATAATTGGGCAGCGCAAGGCAACCCAGGCTGGAGTTATCAAAACGTCCTCCCCTATTTTATCAAATCGGAAAATTGCAAATTGCTGGATCAAGATATAAGGTTTCATGGGAAAGGAGGATATTTGGACGTGATATCCTCTCCATACGTCTCGCCTTTGAGAGAATGTTTTCTTCGCGGTGGCGAGGAATTAGGATATGACGTAATCGATTACAATGCGGCCAATGTAATTGGTTTTTCGACAGCGCAGGTCCATCTCAGGAACGGACGTAGAGTTAGCGCGAGCAAAGCGTTCTTGAGGCCCATCCGCGAACGAAAGAATTTCCATTTGTCCAAATTGTCCAGAGCGACGAGGATCGTAATCGATCCAAAGAAAAAAGTGGCAGTGGGCGTCGAATTCGTTAAAAATGGCAGAAAACGATTCGTCTCTGCTAGTAAGGAGATAATACTTTCAACGGGCACTTTAAATTCGCCACAATTGTTAATGCTATCTGGAATAGGACCCAAAGATCACCTCGAATCTTTGAATATCGATTCGATCGAGGATCTGCAGGTCGGATATAACCTCCAAGATCATGTCAGCATGTCCATGTTGACTTTTCTCGTAAATGAGAGCGTGACTATCGTCGAGCCCCGGATTGCCTCGAATCTGGCCAATATTTTGGATTATTTTGTCAAAGGAACTGGACCCTTAACGGTACCCGGAGGAGCGGAATGTCTTGCGTTTATCGACACGAAAGAAGATCGATCTATCCGGCTAATGAAAAAATTTCAAGTTAATAATACGAAATTTCAAACGAATGTTCCGGATATTGAACTGGTTCTAGGTATAAGCGCTCTAACCGGAGATATATCGGGTAGTTACAGAGGTCTTCTGGGTTTGACGAACGAATTTTACAAAGAAGTATTCACTGGTTACGAAGGTTACGACGCCTTTTCCATCGTTCCAGTCCTCTTGCAGCCGAAAAGTAGGGGGAGAGTTACTTTGAAAAGCTCCGATCCCTTTGACCGACCTATTTTCGAAACAAATTACTATGATCACGAAGACGATCTCAGAACTATGTTAATTTTTCAGGCCATAGAAGTGGCATCGACCAAAGCGTTTAAACGTTTCAATGCAACGTTACTGCCAGTTGCGTTTCCAGGATGCAAACATGTCCCGTTCGGTACAGATCCGTATTGGGCTTGTGTTGCTCGGCAAGTGACCACGACCCTGGGTCATTTCGTAGGAACGTGTAAGATGGGCCCAAGGAGGAACTCGGGTGTAGTGGATCACAGGTTACGAGTGCACGGGATCAATGGTCTCAGAGTTGTGGATGCCAGTATAATCCCTACCATAGTAACAGGCCATACGAATGCGGTTGCTTATATGATCGCTGAAAAGGCTGCGGATATGATAAAAGAAGATTGGAAAGTATTGAATACTGAATTTGATCGAACATTTAGGAAAAATTAG>AmGMC_B7ATGGAATCGTGTATGAGCAGAACTTGTTCGTCGGTGATCGCACAGCAATCGTCACCAGCCTCCATATTTACTTTCTTGATTCAAACGTTAATAGCGTCCCGTTGTAAACTGAACAACCCGGATGAATATCCCAGAGATCGCGTAAATGACGTTTTAAGATCGAACAAGGAATTCGATTTTGTGATCATCGGTGGTGGAACCGCGGGATCTATTTTGGCTCGCAGATTAACGGAAGTGAAGAATTGGAACGTTCTGTTAATCGAACGAGGAGGATACCCTTTACCCGAAACTGCAGTGCCTGCTTTATTCACCAGCAATTTGGGCTTCCCACAGGATTATGCTTACAAGATCGAATATCAAAAGGAAGCCTGTTTGAGCCAAGTCGACAAACGATGCAGATGGTCGAAGGGTAAAGCCCTTGGTGGAAGTTCCGTCATCAACGCTATGCTGCACATATTTGGCAACAAAAGGGACTACGACACTTGGGAAAATATAGGAAATCCCGGATGGAATTACGAACAAGTACTCCCGTACTTTAGAAAATCGTTGAGCTGTGCACCCGAATTCATAGCCAAATACGGGACCGATTATTGCGGGACAGACGGTCCTATGAGAATCAGACACTACAATTACACTGCGACAGACGCAGAGGACATAATCTTGGAAGCCGCGCACGAGGCGGGATACGATGTTCTCGAGCCATTGAACGGTGATCGGTTTATCGGGTTTGGAAGAGCGATGGGCACCCTGGACAACGGGCAACGGGAAAATTGTGCGAAAGCTTTCCTCTCCCCCGTCAAAGACAGGAAGAACTTGTACGTGATGACGTCGAGCAGAGTGGACAAGATTCTGTTCGAGAGGAAACGGGCGGTCGGTGTACGAATCACTTTGGACAACAATCAATCGGTGCAAGTGAGAGCGACGAAGGAAGTGATTCTGTCGGCGGGGAGTATAGCCAGTCCTCAGGTATTGATGCTCTCGGGAATAGGGCCGAAGAACCATTTGAAAAAAATGGGAATACCCACTCTCGTCGATCTGCCTGTCGGGAAGAATCTTCAAGATCACGCGATATGGTTAGGAATATACTTGGCCTACAATAATGAATCGGTGACGTCCCCTCCTAGCGAGAAGAGTCAGTTAGATGATATTTATGATTATTTGGAATTCAATGCCGGCCCGTTACGCGTTCTTCCACTCGATCTGAACGGTTTCGTGGACGTGAACGATCCCCACTCGAAATATCCGAATGTTCAATTTATGTTCGTACCTTATCAACGTTACACCAACAATTTGTTATCTTTATTACAAGGATACAATATGAATGATGACATTATTCAAGAAATGCAACAAGCTGTAAAAAAGATGAGCTTGATCTCTATTTGTCCCGTATTGATAAGACCTTTGAGCCGAGGTTTTGTCGAGTTGCGTAACACCAATCCGGCCGATCCTGTCAAGATCTACGCGAATTATTTCGCCGAGAAGGAAGATTTCAATAATTTGTTGAAATCCGTGAATATCGTTAAGGCTTTCTTGAACACCGACATTCTGAAGAAGTACAACATGACGTTATATTATCCCAACATTTCCGGATGTCAACACACGGAGCCAGGTACCGATGAATATTGGGAATGCAACCTGGAACACTTGTCCACCACGTTGTTTCATCCGTGTGGGACGGCCATGATGGGACCGGCTAACGATTCCAGAGCCGTTGTGGATTCGAGGTTGAAAGTGCACGGTGTCCAAAATTTGCGAGTGATCGATGCGTCGATCATGCCGGAAGTGACGAGCGGGAATACCAATGCGCCGACTATGATGATCGCGGAGAAGGGAGCGGATATAATTAAACAGGATTGGGGCGTAAAGATACAGATATGA
